# Organocatalytic Enantioselective Addition of Malonates to Cyclic Imines: A Short Total Synthesis of Tetraponerine T‐1

**DOI:** 10.1002/anie.8336123

**Published:** 2026-07-11

**Authors:** Frederic Kölblin, Helma Wennemers

**Affiliations:** ^1^ Laboratory of Organic Chemistry ETH Zürich Zürich Switzerland

**Keywords:** alkaloids, bioinspired synthesis, imines, organocatalysis, stereoselective synthesis

## Abstract

Cyclic imines are common intermediates in the biosynthesis of alkaloids, but their use in enantioselective organic synthesis is limited by their high reactivity and tendency to trimerize. Here, we present a mild organocatalytic method for the stereoselective addition of malonates to cyclic imines. Under carefully optimized conditions that suppress background reactivity, cinchona alkaloid‐derived catalysts enabled access to α‐functionalized pyrrolidines and 5‐6‐5 and 6‐6‐5 tricycles in high yields and stereoselectivity. Mechanistic studies revealed that the catalyst controls the stereochemistry of the initial addition, while subsequent reactions are governed by the substrate. The total synthesis of the alkaloid Tetraponerine T‐1, the shortest route reported to date, highlights the synthetic utility of the methodology.

## Graphical abstract

A stereoselective functionalization method of cyclic imines under mild organocatalytic conditions was developed. Bifunctional cinchona alkaloid catalysts combined with carefully optimized reaction conditions to suppress background reactivity enabled highly enantio‐ and diastereoselective addition reactions of malonates to cyclic imines. The utility of the method is highlighted with a straightforward total synthesis of tetraponerine T‐1.

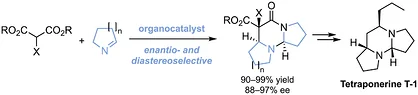

## 1 Introduction

**SCHEME 1:**
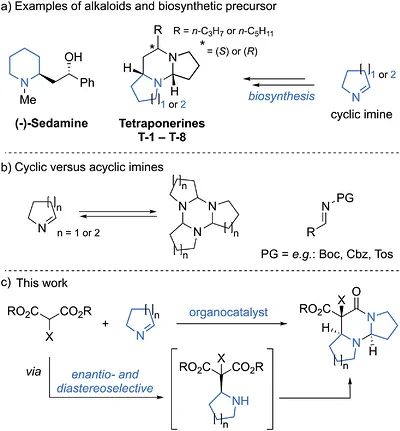
(a) Examples of natural alkaloids biosynthetically made from cyclic imines. (b) Equilibrium between mono‐ and trimeric cyclic imines and in organic synthesis, commonly used acyclic imines. (c) Organocatalytic, stereoselective addition of malonates to cyclic imines.

Cyclic amines are widespread among natural products and bioactive substances. Alkaloids with one or more rings are prominent examples (Scheme 1a) [1, 2, 3]. Nature uses cyclic five‐ and six‐membered imines as building blocks for their synthesis. In organic synthesis, however, the reactivity of these electrophiles is difficult to control [4, 5, 6]. Aside from their high reactivity towards nucleophiles, cyclic imines also react with one another to form cyclic trimers. Reactions with cyclic imines are, therefore, challenging, especially in asymmetric catalysis where the reactivity and stereoselectivity must be carefully controlled. As a result, only a few enantioselective reactions with cyclic imines have been reported to date [7, 8, 9, 10, 11]. In contrast, acyclic imines bearing carbonyl or sulfonyl protecting groups are less basic and commonly used in stereoselective catalysis (Scheme 1b) [12, 13, 14, 15].

Inspired by nature's alkaloid biosynthesis, we asked whether hydrogen bond donor catalysts can facilitate stereoselective addition reactions to cyclic imines under mild organocatalytic conditions (Scheme 1c). We envisioned that such reactions would enable the straightforward, protecting group‐free synthesis of tetraponerines. These alkaloids are a class of eight closely related compounds that consist of either 5‐6‐5 or 6‐6‐5 annulated ring systems with a propyl or pentyl sidechain (Scheme 1a). Tetraponerines are contact poisons used by the *Tetraponera* ant that paralyze their enemy by non‐competitively inhibiting the nicotinic acetylcholine receptor [16, 17, 18]. Activity has also been observed against colon cancer and insects [19]. The biosynthesis of tetraponerines involves reactions of cyclic imines derived from ornithine with ß‐ketoacid building blocks to yield the 5‐ and 6‐membered tricycle [20, 21]. Their tricyclic scaffold has made tetraponerins interesting targets for total synthesis. The first enantioselective synthesis utilized a dipolar cycloaddition with Ellman's auxiliary for chiral induction [22]. Chiral auxiliaries were also used in several other total syntheses [23, 24, 25, 26, 27, 28, 29]. Catalytic enantioselective syntheses of tetraponerines **T‐6**, **T‐7,** and **T‐8** have relied on a Pd‐catalyzed asymmetric allylic alkylation followed by a Ru‐catalyzed ring rearrangement [30]. Stereoselective Sharpless dihydroxylation and organocatalyzed *aza*‐Michael addition have also been used as entry points into enantioselective syntheses [31, 32]. Starting from cyclic imines, only the synthesis of racemates has been accomplished, with comparatively low yield, highlighting how challenging controlled nucleophilic additions to cyclic imines are [19, 33].

Here, we present the enantioselective addition of various malonates to cyclic imines catalyzed by cinchona alkaloid‐derived hydrogen bond donor organocatalysts. Chiral tricyclic amines with up to three stereogenic centers were obtained in one step with high yields and stereoselectivities. Mechanistic studies elucidated that the stereoselectivity is established by the initial, catalyst‐controlled addition step. The synthetic methodology allowed straightforward access to 5‐6‐5 and 6‐6‐5 tricycles and to the shortest total synthesis of the natural product Tetraponerine T‐1.

## 2 Results and Discussion

### 2.1 Reactivity of Pyrroline and Reaction Development

**SCHEME 2:**
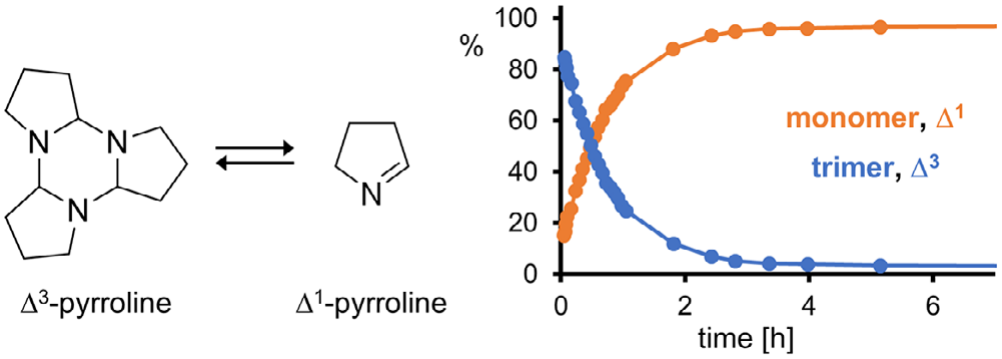
Monitoring of trimer disassembly of **1a** over time in CD_2_Cl_2_ (100 mM).

We started by exploring the reactivity of pyrroline (**1a**), a cyclic 5‐membered imine. NMR spectroscopic analyses showed that monomeric and trimeric pyrroline are in equilibrium with each other. In dichloromethane (CD_2_Cl_2_) and chloroform (CDCl_3_), trimeric pyrroline converts into monomeric pyrroline within a few hours, whereas this disassembly is significantly slower in tetrahydrofuran (THF; Scheme 2 and S1).

Next, we probed the reactivity of pyrroline toward malonate derivatives, analogs of malonyl‐CoA, a common natural building block in alkaloid syntheses [1]. Upon addition of monothiomalonates (MTMs) to **1a**, the 5‐6‐5 core of tetraponerines formed, even at low temperature and in the absence of a catalyst (Table 1, entry 1 and Table S1). This result shows that uncatalyzed background reactivity is rapid and must be overcome when designing a stereoselective catalytic reaction. The result also showed that the initial addition product is prone to further reaction to form the tricycle.

**TABLE 1:** Optimization of the reaction conditions.

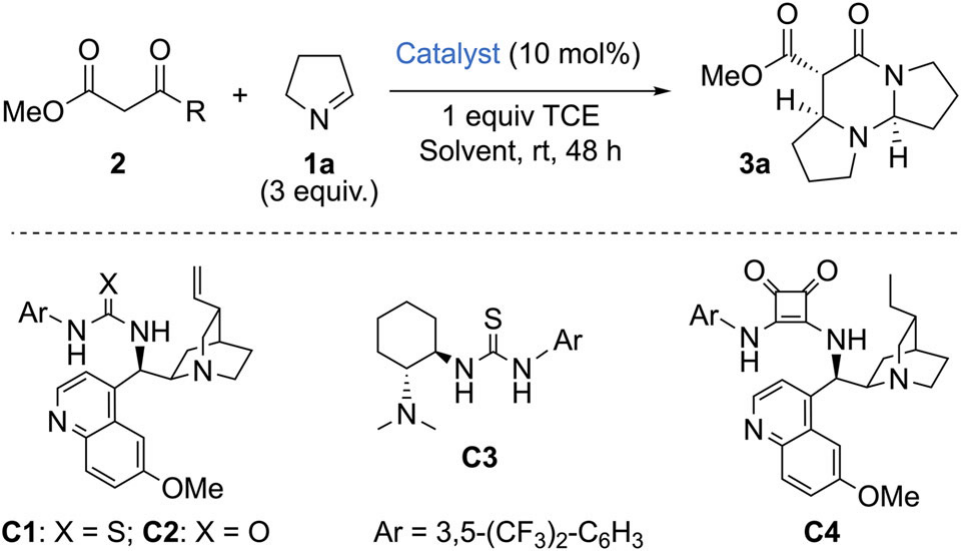					
Entry	Cat.	R	Solvent	Conv.^a^	ee^b^
1	none	SPMP^c^	CH_2_Cl_2_	>95%	—
2	**C1**	OMe	CH_2_Cl_2_	30%	90%
3	**C2**	OMe	CH_2_Cl_2_	15%	62%
4	**C3**	OMe	CH_2_Cl_2_	30%	84%
5	**C4**	OMe	CH_2_Cl_2_	47%	93%
6	**C4**	OMe	Toluene	58%	66%
7	**C4**	OMe	CH_2_Cl_2_ (85 mM)	32%	93%
8^d^	**C4**	OMe	CH_2_Cl_2_	81%	91%
9^d^, ^e^	**C4**	OMe	CH_2_Cl_2_	>95% (97%)	92%
10	**C4**, no TCE	OMe	CH_2_Cl_2_	51%	77%

*Note*: The reactions were carried out at a 0.1 mmol scale at a concentration of 170 mM unless otherwise noted. TCE = 1,1,2,2‐tetrachloroethane. With the exception of the monothiomalonate (entry 1), **3a** formed as a single diastereoisomer.

^a^ Determined by ^1^H NMR spectroscopic analysis. Yield of isolated product in brackets.

^b^ Determined by chiral stationary phase HPLC analysis.

^c^ PMP = *p*‐methoxyphenyl.

^d^ 4 equiv. of imine.

^e^ The cyclic imine was purified by distillation before use.

We reasoned that uncatalyzed background reactivity could be reduced by using a less acidic malonate derivative. Indeed, dimethyl malonate did not react with pyrroline in the absence of a catalyst. (For a reactivity test with other malonate derivatives, see Scheme S2). We therefore used dimethyl malonate to explore catalytic, stereoselective addition reactions with cyclic imines. As catalysts, we tested H‐bond donor catalysts **C1**–**C4** (Table 1) as well as **C5**–**C12** (Scheme S3 and Table S2), which have been used in related reactions with linear imines [34, 35, 36, 37, 38, 39, 40, 41]. In addition, we varied common reaction parameters such as the solvent (e.g., toluene, THF, MeCN, acetone, CH_2_Cl_2_, and CHCl_3_) and the concentration (Table S3). In all cases, the reaction proceeded to tricycle **3a**. Tetrachloroethane (TCE, 1 equiv.) served as an internal standard to determine the conversion by NMR spectroscopy. The highest enantioselectivity was obtained with squaramide catalyst **C4** (10 mol%) in CH_2_Cl_2_ (Table 1, entry 5). The use of 4 equivalents of freshly distilled imine **1a** improved the conversion and enabled the formation of tricycle **3a** with a yield of 95% and an enantioselectivity of 92% ee (Table 1, entries 8 and 9). Under these conditions, **3a** formed as a single diastereoisomer. Of note, TCE, the internal standard used for NMR‐spectroscopic monitoring, proved to be important for both enantioselectivity and yield (Table 1, entry 10). More equivalents or the use of TCE as a solvent led to even higher enantioselectivity but at the expense of lower conversion (Table S4). This finding suggests that TCE helps to suppress the background reaction.

**SCHEME 3:**
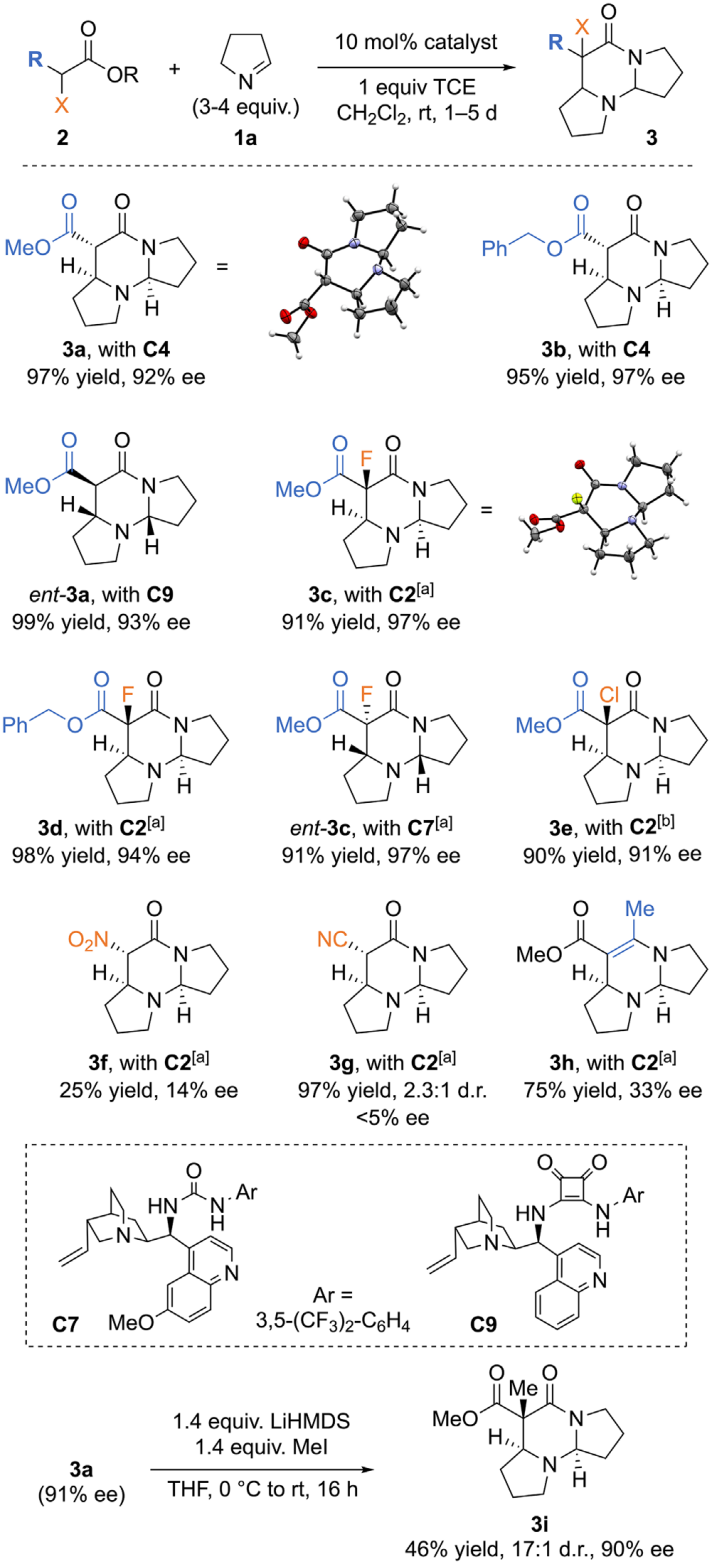
Enantioselective synthesis of 5‐6‐5 ring system by malonate addition to pyrroline. Except for **3g**, single diastereoisomers were observed. (a) Reaction at –15°C. (b) reaction in TCE. Bottom: Methylation of tricycle **3a**.

### 2.2 Scope of Stereoselective Additions to Form Tricycles

With optimized conditions identified, we examined the reaction scope (Scheme 3). The tricyclic addition products of dimethyl and dibenzyl malonate with pyrroline (**3a** and **3b**) were isolated in very good yields (97 and 95%) and with very good enantioselectivity (92 and 97% ee). Bulkier esters, including diethyl malonate (Scheme S4), were not well tolerated (<25% conversion). Fluoromalonates are more reactive and require a lower temperature (–15°C) to avoid uncatalyzed background reaction. With these substrates, catalyst **C2** proved to be the best and enabled access to products **3c** and **3d** in high yields (91% and 98%) and enantioselectivities (97% and 94% ee; Scheme 3 and Table S6). Also, dimethyl chloromalonate reacted well and gave product **3e** in 90% yield and 91% ee. Due to the higher reactivity of the chloromalonate and, thus, uncatalyzed background reactivity, this reaction was carried out in TCE as solvent. Other acidic substrates, such as the methyl esters of nitroacetate and cyanoacetate, as well as a β‐ketoester, provided the respective products **3f**, **3g**, and **3h**, but with significant uncatalyzed background reactions and, in the case of nitroacetate, unidentified side products. Notably, with the β‐ketoester ring closure to tricycle **3h** proceeded by reaction of the amine with the ketone rather than the ester moiety. This pathway is reminiscent of the proposed biosynthesis of the 5‐6‐5 tetraponerines [20, 21]. More electron‐rich and therefore less reactive malonates, such as methylmalonate, yielded only trace amounts of product, even after 5 days. Yet, deprotonation of **3a** with LiHMDS followed by alkylation with MeI provided straightforward access to methylated tricycle **3i** in 46% yield and with very good stereoselectivity (17:1 d.r., 90% ee, Scheme 3, bottom).

For many cinchona alkaloids, a pseudoenantiomer is available. Reassuringly, use of catalyst **C9**, a pseudo‐enantiomer of cinchonine‐based catalyst **C4**, which lacks the methoxy group at the quinoline moiety and bears a vinyl instead of an ethyl group, yielded the enantiomeric product *ent*‐**3a** with the same high enantioselectivity (Scheme 3 and Tables S5). Similarly, the pseudo‐enantiomer of **C2** (catalyst **C7**) yielded *ent*‐**3c** with excellent yield and enantioselectivity.

Single crystals of **3a** and **3c** revealed the absolute configuration of the 5‐6‐5 ring system, and all other compounds were assigned accordingly [42]. Within this tricyclic structure, the substituents of the central 6‐membered ring are in equatorial positions, with the α‐malonyl substituent in an axial position. The crystal structures are in good agreement with the conformation in solution, as indicated by the coupling constant of ∼10 Hz observed by NMR spectroscopy between the α‐proton of the malonate portion of **3a** and **3b** and the vicinal proton. For the fluorinated tricycles, large coupling constants of ∼24 Hz seen in the ^19^F‐NMR spectra are in agreement with the stereochemistry of the crystal structure of **3c**. Of note, the addition between the malonate and the cyclic imine proceeds with opposite stereochemistry compared to reactions that use carbamate‐protected acyclic imines [14], implying different coordination geometries to the catalyst [43].

### 2.3 Reactivity of Piperideine—Access to Monoaddition Products and 6‐6‐5 Tricycles

**SCHEME 4:**
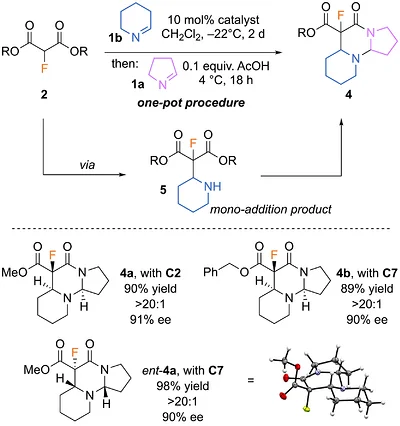
Stereoselective one‐pot synthesis of 6‐6‐5 tricycles.

Next, we investigated reactions with piperideine (**1b**), the 6‐membered cyclic imine (Scheme 4). Prior work has shown that the addition of stoichiometric amounts of NaOH to piperideine yields the 6‐6‐6 ring system [19]. Yet, piperideine is less reactive than pyrroline. We, therefore, envisioned that this lower reactivity offers opportunities for the one‐pot synthesis of tricycles with two six‐ and one five‐membered ring. Natural biosyntheses prepare such 6‐6‐5 ring systems, but organic syntheses starting from cyclic imines have so far not been achieved, not even racemically. We were pleased to observe that the cinchona alkaloid‐urea (**C2**) catalyzed reaction between fluoromalonate and piperideine (2.5 equiv.) stopped after the first addition step, without tricycle formation. Subsequent addition of pyrroline (**1a**) provided the 6‐6‐5 tricycle **4a** in 90% yield, a diastereoisomeric ratio of >20:1, and 91% ee (Scheme 4). This sequential addition of cyclic imines allowed access to 6‐6‐5 tricycles in one pot. Acetic acid was beneficial as an additive (10 mol%) to avoid back reaction of the initial mono‐addition product **5** and formation of the 5‐6‐5 ring system **3a** as a side product (Tables S7 and S8). Reactions with imines derived from 7‐membered azepane or morpholine were attempted, but purification of the imines proved difficult, and addition reactions proceeded with numerous side products (Scheme S8). The effect of acetic acid likely arises from protonation and thereby deactivation of the catalyst (Schemes S6 and S7). Using the pseudo‐enantiomeric cinchona alkaloid‐based catalyst **C7** allowed access to the enantiomers with very good enantioselectivity and yield (Scheme 4). These sequential one‐pot additions worked best with the fluoromalonates. With unsubstituted or chloromalonate, only partial conversion of piperideine took place.

**SCHEME 5:**
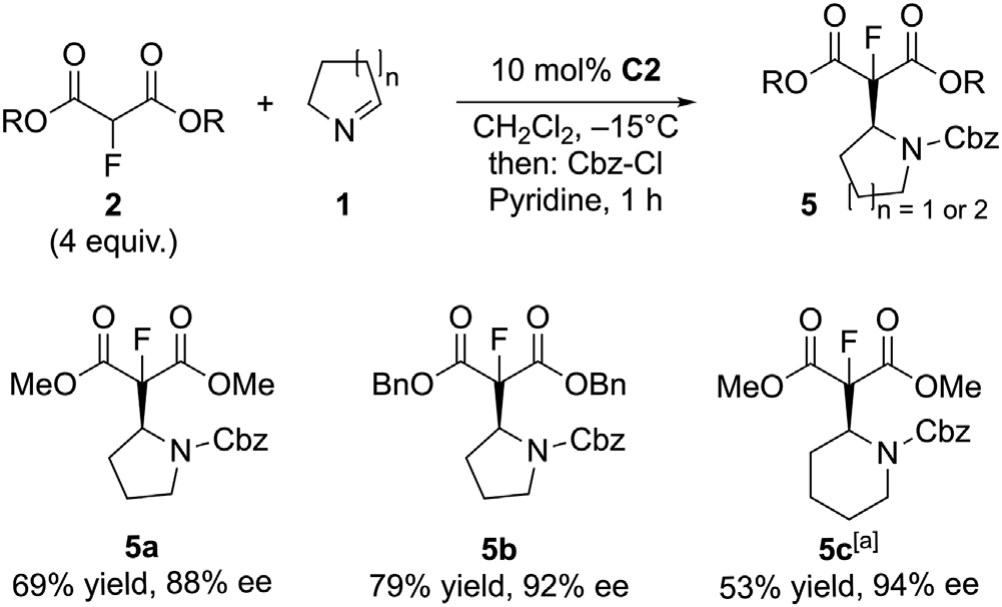
Mono‐addition products. (a) reaction at –22°C.

We then investigated whether the reaction conditions can be tuned to enable the isolation of the mono‐addition product. In the case of pyrroline, an excess of fluoromalonate (4 equiv.) suppressed the incorporation of a second imine (Scheme 5 and Table S9). Protection with Cbz facilitated isolation and prevented retro‐Mannich reaction. This procedure enabled access to mono‐addition products of fluoromalonate **5a**–**5c** with either pyrroline or piperideine in yields of 53%–75% and enantioselectivities of 87%–94% ee. In the case of piperideine, due to its lower reactivity, even an excess of the cyclic imine allowed isolation of the mono‐addition product.

Reactions between pyrroline and other malonates did not stop at the mono‐addition product, even when a greater excess of the malonate (6 equiv.) was employed. These reactions provided the tricycles in comparably high yields and stereoselectivities as observed with an excess of the imine (Scheme 3). This finding corroborates the high reactivity of pyrroline towards the initially formed mono‐addition product that contains a nucleophilic amino group.

### 2.4 Mechanistic Insights

With the mono‐addition products in hand, we asked whether the catalyst is involved in the second addition step to the tricycle or whether this step is controlled by the substrate's stereochemistry. We therefore subjected enantioenriched mono‐addition product **6** to pyrroline in the presence of pseudo‐enantiomeric—matching and mismatching—catalysts **C2** and **C7**, an achiral urea‐based catalyst (**C11**; Scheme S3), and in the absence of a catalyst (Table 2). Acetic acid (10 mol%) was added to suppress retro‐Mannich reaction back to the malonate and piperideine (*vide supra*).

**TABLE 2:** Origin of diastereoselectivity through substrate control.

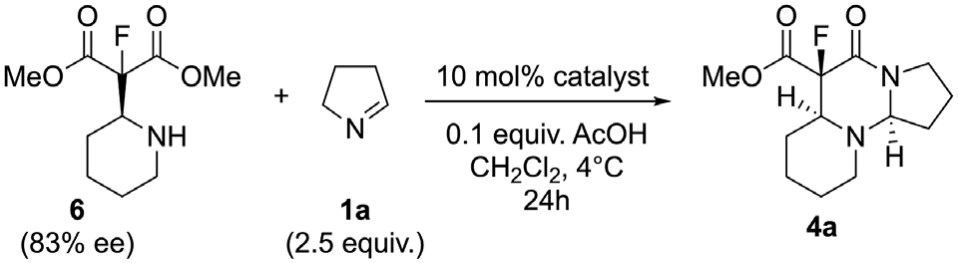			
Entry	Catalyst	Conversion^a^	ee^b^
1	**C2** (match)	96%	82%
2	**C7** (mismatch)	95%	77%
3	**C11** (achiral)	93%	77%
4	—	92%	73%

^a^ Determined by ^19^F NMR spectroscopic analysis of the reaction mixture using trifluorotoluene as internal standard.

^b^ Determined by chiral stationary phase SFC analysis.

Regardless of the presence or absence of a catalyst, tricycle **4a** formed with high stereoselectivity, yet a small loss of enantioselectivity. The lowest drop in ee (Δ = 1% ee) was observed in the presence of catalyst **C2**, the catalyst used to prepare the mono‐addition product **6**. This finding implies that the loss in enantioselectivity is most likely due to retro‐Mannich reaction followed by re‐formation of the mono‐addition product. Overall, these data show that the cinchona alkaloid‐urea catalyst controls the stereochemistry of the first addition step, and the stereochemistry of the mono‐addition product controls the second addition. These findings show that the catalyst‐controlled initial addition is key for high stereoselectivity. In addition, a fast second addition is beneficial to avoid back reaction and uncatalyzed background reactions.

**SCHEME 6:**
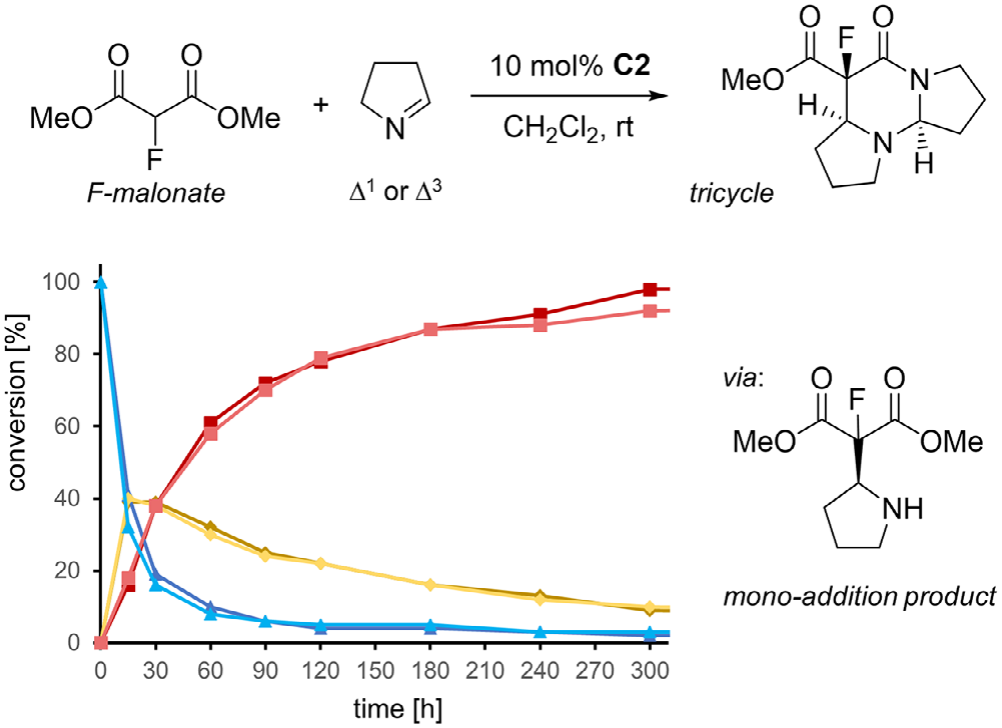
Reaction progress monitored by ^19^F NMR spectroscopy. Blue: F‐malonate, Red: tricycle, Yellow: mono‐addition product; pyrroline was either freshly dissolved (light colors) or dissolved 18 h prior to reaction with F‐malonate (dark colors).

For insight into the rates of the first and second addition, we monitored the addition of dimethyl fluoromalonate to pyrroline in the presence of 10 mol% **C2** by ^19^F NMR spectroscopy (Scheme 6). After 20 min, 40% of the mono‐addition product, 40% of unreacted malonate, and 20% of the tricycle had formed.

Afterwards, the ratio shifts increasingly to the tricyclic double addition product. Interestingly, the reaction progress is essentially identical when using a solution of pyrroline prepared 18 h prior to the reaction with the malonate, or when dissolving pyrroline freshly. This result shows that the equilibrium between monomeric (Δ^1^) and trimeric (Δ^3^) pyrroline does not affect the reaction rate (Scheme 6).

### 2.5 Total Synthesis of Tetraponerine T‐1

**SCHEME 7:**
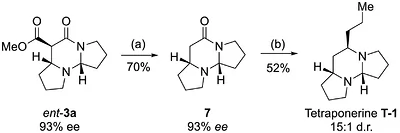
Total synthesis of tetraponerine‐T‐1. (a) NaOH (1 M, aq.), THF, 1 h, rt then HCl (1 M, aq.), 1 h, 80°C, 70% over two steps (b) Vaska's catalyst (IrCl(CO)[PPh_3_]_2_, 1 mol%), TMDS, CH_2_Cl_2_, 20 min, rt then −78°C; *n*PrMgBr, 4 h, −78°C to rt, 52%.

Finally, we explored the total synthesis of Tetraponerine **T‐1** (Scheme 7). Saponification of tricycle *ent*‐**3a**, followed by decarboxylation, gave tricycle **7** in 70% yield and with retained enantiopurity. Vaska's catalyst facilitated the reductive alkylation to the respective hemi‐aminal, which was reacted in‐situ with *n*PrMgBr [44]. This procedure installed the propyl side chain directly and provided **T‐1** along with small amounts of diastereoisomeric **T‐2** (15:1 d.r.) in 52% yield. Optical rotation confirmed the formation of **T‐1** (α_D_ = 15.4 ± 0.5 at 25°C, c = 0.498, Reference (**T‐1**): α_D_ = 14) [26]. The enantiomer of Tetraponerine‐1, *ent*‐**T‐1**, was obtained in similarly high yields and optical purity starting from tricycle **3a** (Scheme S9). Starting from pyrroline, this route enabled stereoselective access to both enantiomers of Tetraponerine **T‐1** in three steps. To the best of our knowledge, this is the shortest enantioselective route reported so far.

## 3 Conclusions

Herein, we have established enantioselective additions of malonates to elusive cyclic imines catalyzed by cinchona alkaloid‐derived hydrogen‐bond donor organocatalysts. This method successfully overcomes the background reactivity of cyclic imines and gives rise to tricyclic scaffolds bearing three non‐adjacent stereogenic centers with high enantio‐ and diastereoselectivity. Reactions with fluorinated malonates enabled access to α‐functionalized cyclic amines in high yields and stereoselectivities. Mechanistic studies revealed the stepwise reaction progress with stereoselectivity established in the initial, catalyst‐controlled addition step. The total synthesis of the natural product Tetraponerine **T‐1**, via the shortest route reported so far, showcases the value of the synthetic methodology. Overall, the study highlights the untapped potential of utilizing cyclic imines in asymmetric catalysis for the synthesis of enantioenriched cyclic amines.

## Author Contributions

**Frederic Kölblin**: investigation, writing – original draft, conceptualization, validation, visualization, formal analysis. **Helma Wennemers**: conceptualization, investigation, funding acquisition, writing – review and editing, validation, visualization, formal analysis, project administration, supervision, resources.

## Conflicts of Interest

The authors declare no conflicts of interest.

## Supporting information




**Supporting File**: The authors have cited additional references within the Supporting Information [45, 46, 47, 48, 49, 50].

## Data Availability

The data that supports the findings of this study are available in the supplementary material of this article
