# Supplementary material for: Organocatalytic Enantioselective Addition of Malonates to Cyclic Imines: A Short Total Synthesis of Tetraponerine T‐1
Source: Angew Chem Int Ed Engl. 2026 Jul 11;65(38):e8336123. doi: 10.1002/anie.8336123 (PMC13573027; doi:10.1002/anie.8336123)
Supplement: Supplementary file 1 [file ANIE-65-e8336123-s001.pdf]

Supporting Information

Organocatalytic Enantioselective Addition of Malonates to Cyclic Imines: A Short Total Synthesis of Tetraponerine T1

Frederic Kölblin, Helma Wennemers*

Laboratory of Organic Chemistry, ETH Zurich, Vladimir-Prelog-Weg 3, CH-8093 Zurich, Switzerland.

email: helma.wennemers@org.chem.ethz.ch

Table of Contents

1 GENERAL INFORMATION **3**

2 SCHEMES MENTIONED IN THE PAPER **4**

2.1 OPTIMIZATION OF CATALYTIC CONDITIONS 6

3 SYNTHESIS OF STARTING MATERIALS **17**

3.1 SYNTHESIS OF PYRROLINE 17

3.2 SYNTHESIS OF PIPERINE FROM CHLORAMINES 18

3.3 SYNTHESIS OF MALONATES 20

4 SUBSTRATE SCOPE **22**

4.1 GENERAL PROCEDURE FOR REACTION WITH PYRROLINE 22

4.2 ANALYTICAL DATA OF 5-6-5 PRODUCTS 23

4.3 GENERAL PROCEDURE OF SEQUENTIAL REACTION WITH PIPERIDEINE AND THEN PYRROLINE 30

ANALYTICAL DATA OF 6-6-5 PRODUCTS 31

4.4 GENERAL PROCEDURE FOR MONO-ADDITION REACTIONS FOLLOWED BY CBZ PROTECTION 33

4.5 ANALYTICAL DATA OF MONO-ADDITION REACTIONS 34

4.6 SYNTHESIS OF UNPROTECTED MONO-ADDITION PRODUCT 36

5 TOTAL SYNTHESIS OF TETRAPONERINE T-1 AND *ENT*-T1 .... **37**

6 X-RAY CRYSTALLOGRAPHIC DATA **39**

7 REFERENCES **42**

8 NMR SPECTRA OF STARTING MATERIALS **43**

8.1 CYCLIC IMINES 43

8.2 NMR SPECTRA OF MALONATES 46

9 NMR SPECTRA AND CHIRAL STATIONARY PHASE HPLC CHROMATOGRAMS OF PRODUCTS **51**

9.1 NMR AND CHIRAL STATIONARY PHASE OF 5-6-5 PRODUCTS 51

9.2 NMR AND CHIRAL STATIONARY PHASE HPLC CHROMATOGRAMS 6-6-5 RING SYSTEMS 73

9.3 NMR SPECTRA AND CHIRAL STATIONARY PHASE HPLC CHROMATOGRAMS OF MONO ADDITION PRODUCTS 80

10 NMR SPECTRA AND CHIRAL STATIONARY PHASE HPLC CHROMATOGRAMS OF COMPOUNDS IN TETRAPONERINE TOTAL SYNTHESIS **92**

1 General Information

Unless otherwise noted, all reactions were carried out under an ambient atmosphere without the exclusion of air or moisture. All reagents were purchased from commercial suppliers in the highest available grade and used without further purification unless otherwise stated.

Reactions were monitored by thin-layer chromatography using Merck silica gel 60 F254 glass plates and visualized with 254 nm light or potassium permanganate staining solutions followed by heating. Automated flash column chromatography was performed using a Teledyne Isco CombiFlash system with prepacked silica columns. Flash column chromatography was performed using silica gel column chromatography (SiliaFlash® P60, 40-63 µm, 230-400 mesh). Reactions at lowered temperature were cooled either using a Cryostat or when performed at -22°C in the freezer or at 4°C in the fridge.

^{1}H, ^{13}C and ^{19}F NMR spectra were recorded on a Bruker Neo 500 MHz, a Bruker Avance III 400 MHz, a Bruker Neo 400 MHz and Bruker Avance III 600 MHz spectrometers. Chemical shifts (δ) reported in parts per million (ppm) with the solvent resonance as the reference and are reported as following: s = singlet, d = doublet, t = triplet, q = quartet, m = multiplet or unresolved, br = broad signal.

High-resolution mass spectra were measured by the Molecular and Biomolecular Analysis Service in the Laboratory of Organic Chemistry at ETH Zürich on a Bruker maXis - ESI-QTOF (high-resolution ESI) spectrometers. IR spectra were recorded neat on an Agilent Cary 630 FTIR and are reported as absorption maxima (cm^{-1}). Crystals suitable for X-Ray analysis were selected and measured on a XtaLAB Synergy, Dualflex, Pilatus 300K diffractometer. Optical rotation was measured on a Jasco P-2000 Polarimeter with a 10 cm cuvette (1.5 mL).

High-performance liquid chromatography (HPLC) analyses were performed on an analytical Ultimate 3000 HPLC system with a diode array detector and chiral stationary phase columns from Daicel (AD-H, AS-H, OJ-H, OD-H, IC, IA, IF, IBN). Supercritical fluid chromatography (SFC) analyses were performed on an analytical Waters ACQUITY-UPLC with a diode array detector using chiral stationary phase columns from Daicel or Waters (Whelk, 150 mm x 30 mm). Samples for HPLC or SFC analysis were prepared by dissolving the sample in *n*-hexane/*i*-PrOH (4:1) and filtering through an Acrodisc® CR 4 mm syringe filter with a 0.45 µm PTFE membrane. The separation conditions used for each compound are reported below.

Catalysts were synthesized according to reported procedures.[34-38] Dimethyl malonate, dibenzyl malonate and diemthyl fluoromalonate (Sigma-Aldrich) were used as received.

2 Schemes mentioned in the paper

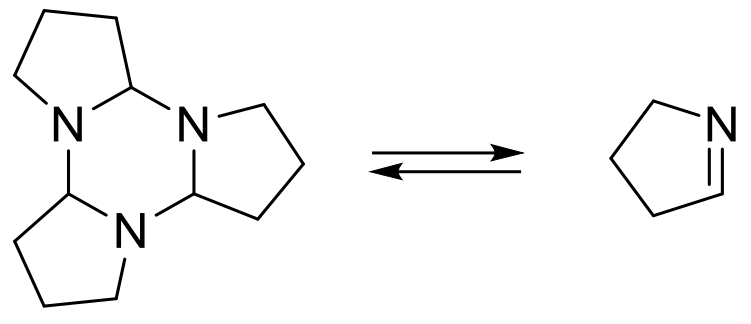

Δ^3-pyrroline Δ^1-pyrroline

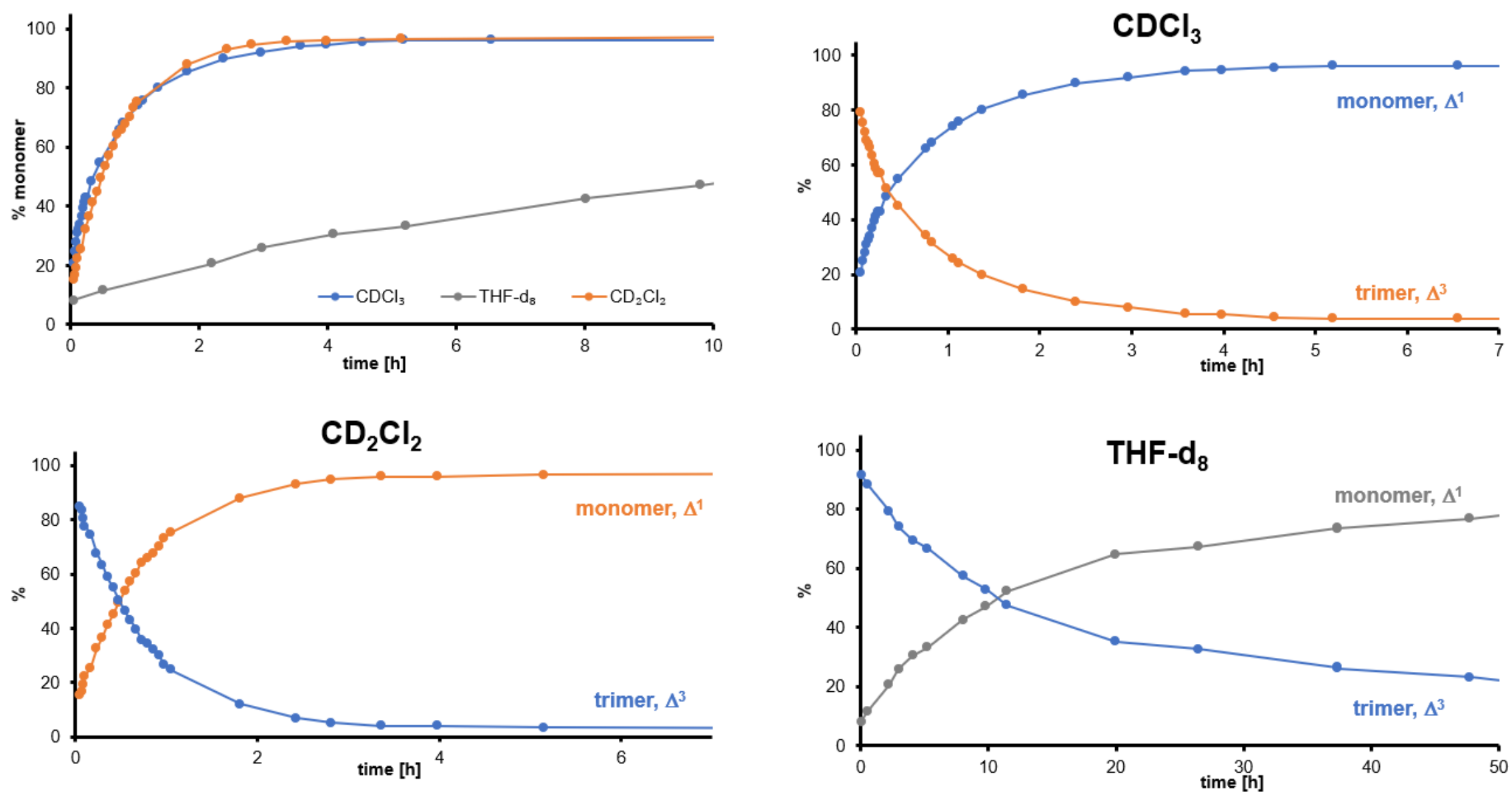

Scheme S1: Trimer disassembly of 1a monitored by ^{1}H NMR spectroscopy over time in $CDCl_3$, CD_2Cl_2 and THF-d_8 (100 mM)**.**

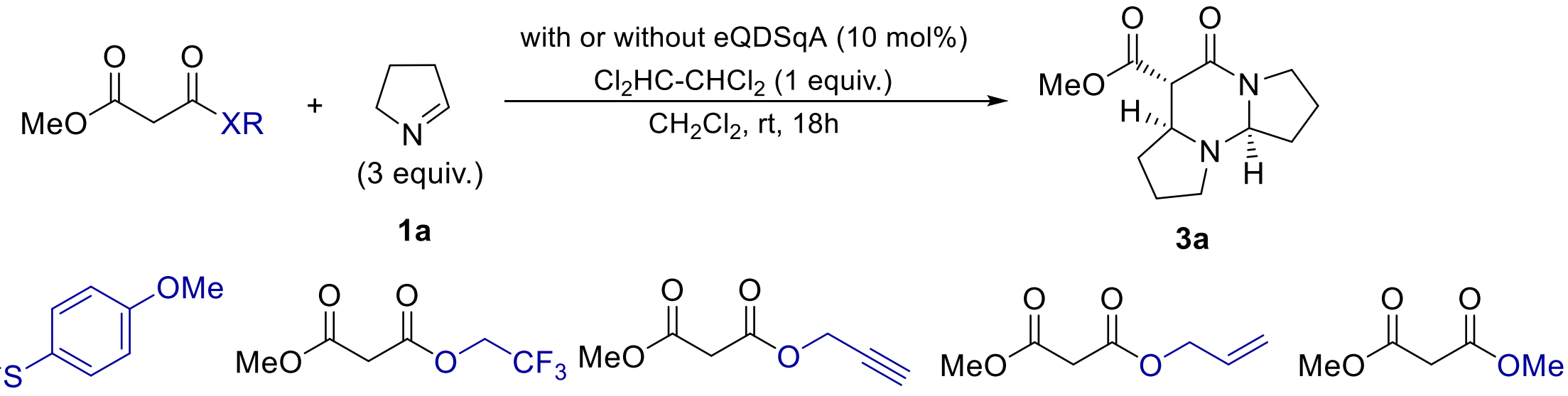

	**SI-2a**	**SI-2b**	**SI-2c**	**SI-2d**	**2a**
**with catalyst:**	8% ee<br>>95% conversion	20% ee<br>90% conversion	20% ee<br>85% conversion	88% ee<br>32% conversion[c)]	93% ee<br>47% conversion
**without catalyst:**	>95% conversion	>95% conversion	85% conversion	0% conversion	0% conversion

Scheme S2: Use of different esters in the addition reaction.

a) Conversion was determined by ^{1}H NMR using TCE as internal standard b) ee determined by chiral HPLC c) mixture of both possible ester products, the listed ee is for product **3a**.

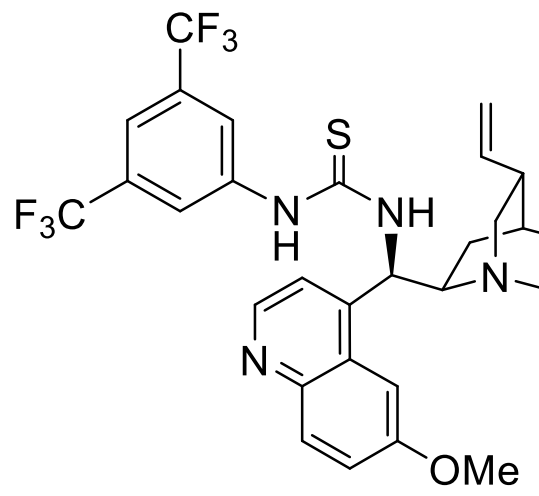

C1
eQDTU

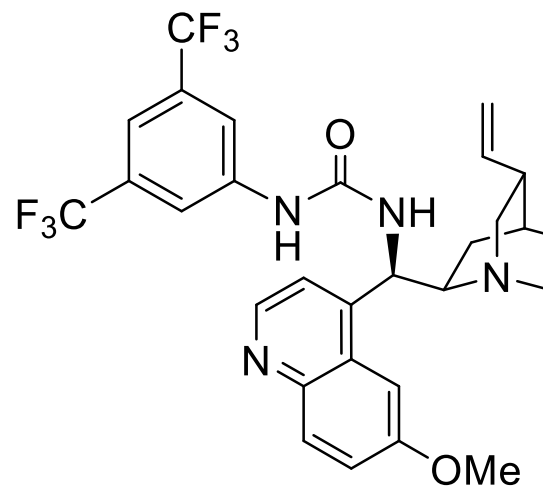

C2
eQDU

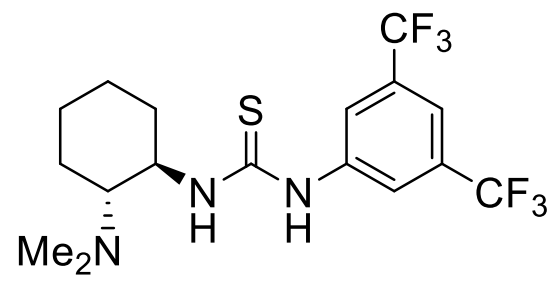

C3

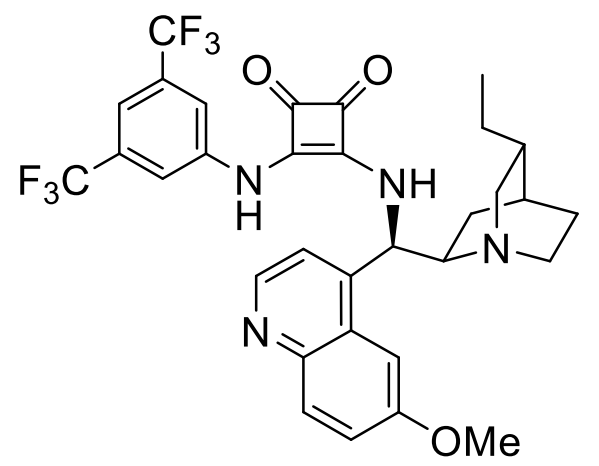

C4

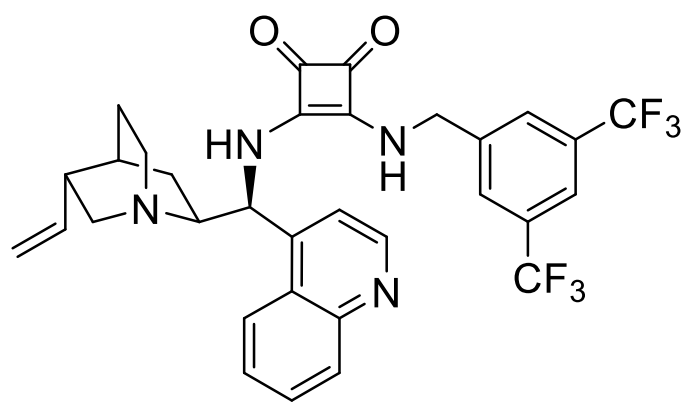

C5

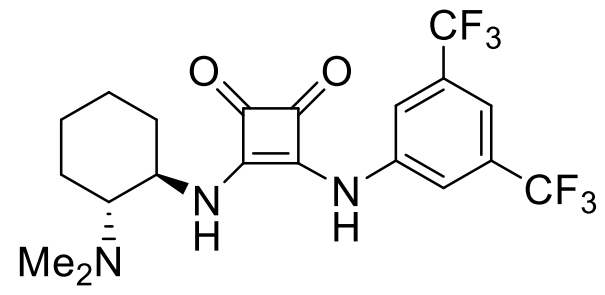

C6

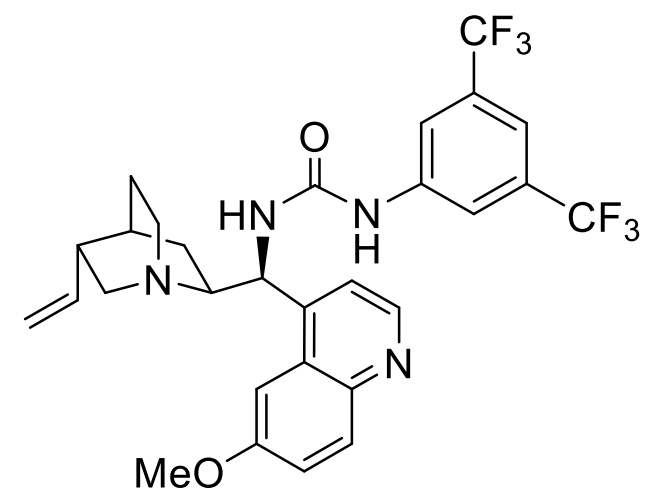

C7
eQNU

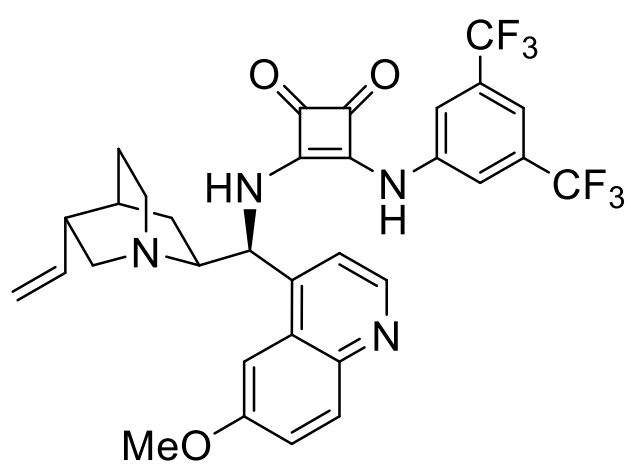

C8
***e*QNSqA**

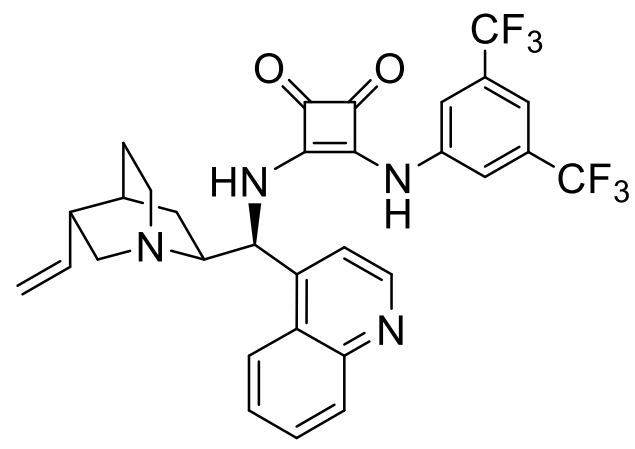

C9
***e*CDSqA**

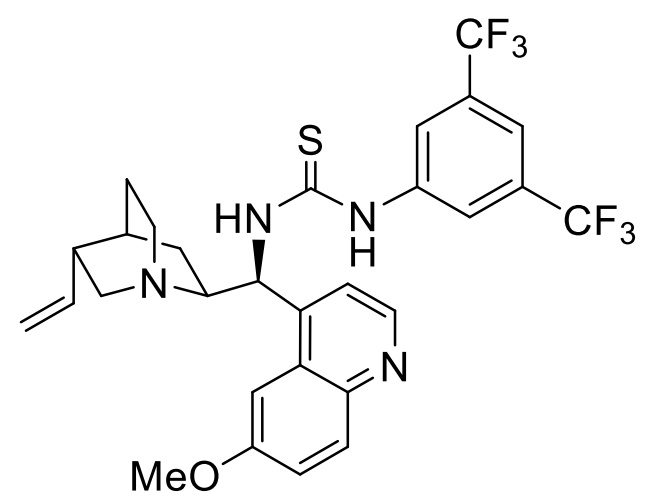

C10
***e*QNTU**

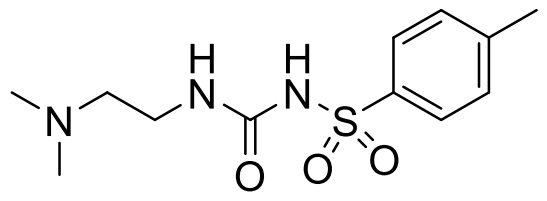

C11
achiral catalyst

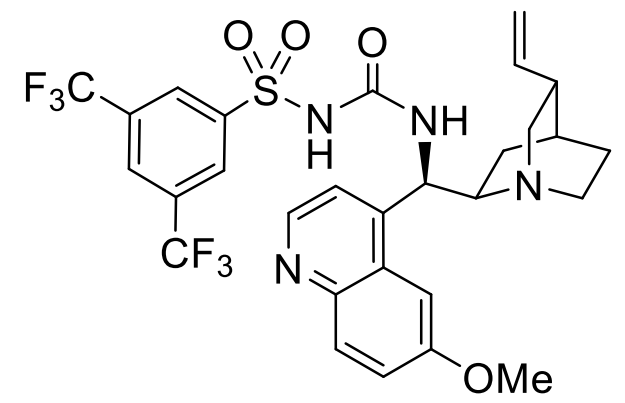

C12

Scheme S3: Catalysts used in this study.

2.1 Optimization of catalytic conditions

General Procedure I: **Optimization of Reaction Conditions**

All screening reactions were conducted on a 0.1 mmol scale with respect to the limiting substrate. An HPLC or a 5 mL Biotage microwave reaction vial was charged with the catalyst, dissolved in the solvent and the malonate was added using an Eppendorf pipette. The cyclic imine was added last. The reaction mixture was stirred for the given time at the given temperature. A sample (100 μL) was taken from the reaction mixture, dissolved in $CDCl_3$ (450 μL), and the conversion was determined by ^1H and ^{19}F NMR. The enantiomeric excess was determined by chiral stationary phase HPLC after a preparative TLC purification.

Table S1: Catalyst screening with H-MTM SI-2a.

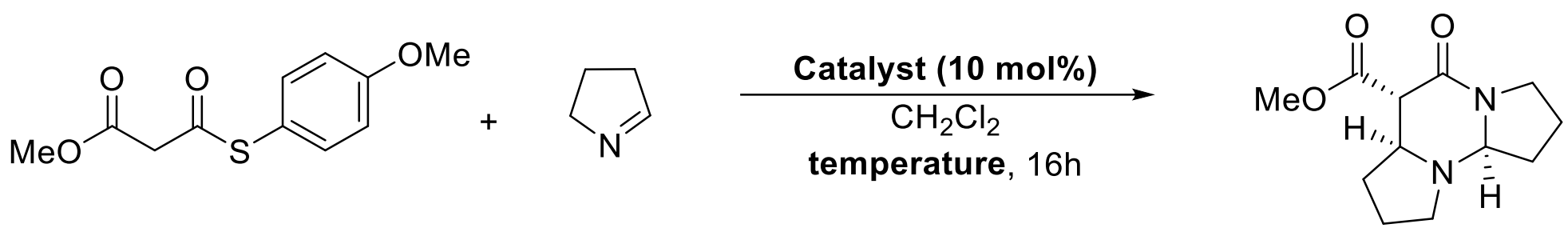

SI-2a **1a** (3 equiv.) **3a**

Entry	Catalyst	Temperature	Conversion to 3a[a]	*ee*[b]
**1**	**C1**	rt	>95%	<5%
**2**	**C2**	rt	>95%	0%
**3**	**C3**	rt	>95%	6%
**4**	**C4**	rt	>95%	8%
**5**	**C12**	rt	>95%	0%
**6**	**C4**	-50°C	49%	5%
**7**	-	rt	>95%	-
**8**	-	0°C	64%	-
**9**	-	-50°C	34%	-

a) Conversion was determined by ^1H NMR spectroscopy using TCE as an internal standard
b) determined by chiral stationary phase HPLC

Table S2: Catalyst optimization with dimethyl malonate.

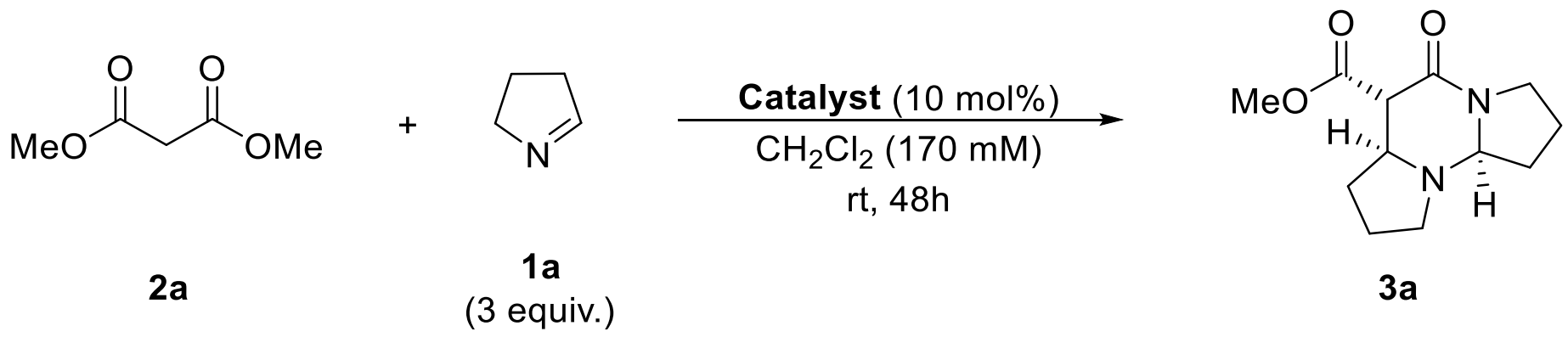

Entry	Catalyst	Conversion to 3a[a)]	*ee* [b)]
**1**	**C1**	30%	90%
**2**	**C2**	15%	62%
**3**	**C3**	30%	84%
**4**	**C4**	47%	93%
**5**	**C5**	35%	39%
**6**	**C6**	55%	66%
**7**	**C12**	7%	<5%

a) Conversion was determined by ^{1}H NMR spectroscopy using TCE as internal standard
b) determined with chiral stationary phase HPLC.

Table S3: Optimization of solvent, concentration and catalyst loading with C4.

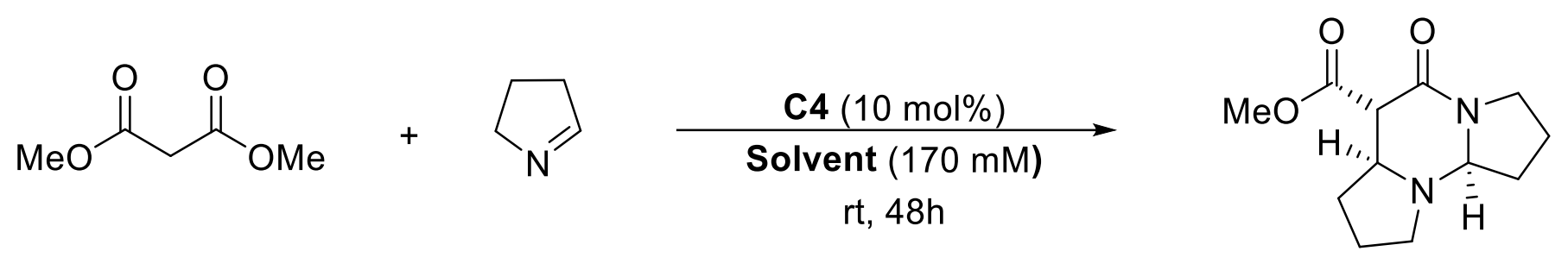

Entry	Solvent (conc.)	Deviation	Conversion to 3a [a)]	*ee* [b)]
**1**	CH_2Cl_2	-	47%	93%
**2**	Toluene	-	58%	66%
**3**	THF	-	50%	71%
**4**	MeCN	-	42%	82%
**5**	Acetone	-	<5%	n.d.
**6**	MeOH	-	64%	5%
**7**	$CHCl_3$	-	56%	78%
**8**	DCE	-	35%	68%
**9**	CH_2Cl_2	concentration 85 mM	47%	93%
**10**	CH_2Cl_2	20 mol% catalyst	76%	80%
**11**	CH_2Cl_2	4 equiv Imine	81%	91%
**12**	**CH_2Cl_2**	**4 equiv. distilled Imine**	**>95%**	**92%**

a) Conversion was determined by ^{1}H NMR spectroscopy using TCE as internal standard.
b) determined with chiral stationary phase HPLC.

Table S4: Additive testing

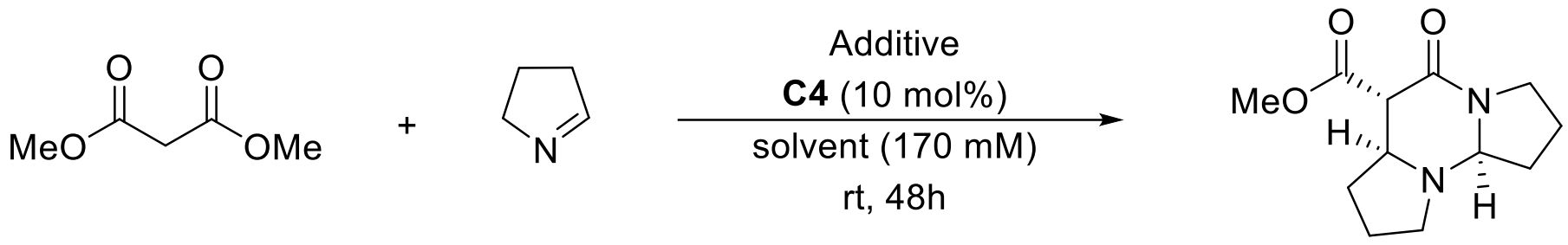

2a 1a (3 equiv.) 3a

Entry	Additive	Solvent	Conversion to 3a [a)]	*ee* [b)]
**1**	4Å Molecular sieves	CH_2Cl_2	64%	54%
**2**	1 equiv. Trimethoxybenzene	CH_2Cl_2	56%	76%
**3**	no additive	CH_2Cl_2	51%	77%
**4**	0.2 equiv. TCE	CH_2Cl_2	49%	88%
**5**	**1 equiv.TCE**	**CH_2Cl_2**	**50%**	**92%**
**6**	5 equiv. TCE	CH_2Cl_2	37%	95%
**7**	-	TCE	17%	96%

a) Conversion was determined by ^{1}H NMR spectroscopy. b) determined with chiral stationary phase HPLC.

Table S5: Catalyst optimization for the opposite enantiomer.

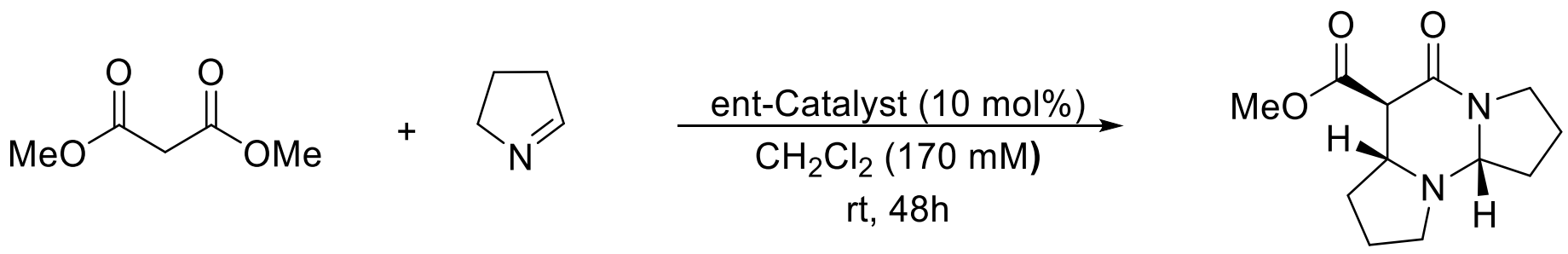

2a 1a (4 equiv.) **single diastereoisomer** *ent*-**3a**

Entry	Catalyst	Conversion to *ent*-3a [a)]	*ee* [b)]
**1**	**C8**	87%	89%
**2**	**C9**	**82%**	**93%**
**3**	**C10**	58%	82%

a) Conversion was determined by ^{1}H NMR spectroscopy using TCE as internal standard. b) determined with chiral stationary phase HPLC.

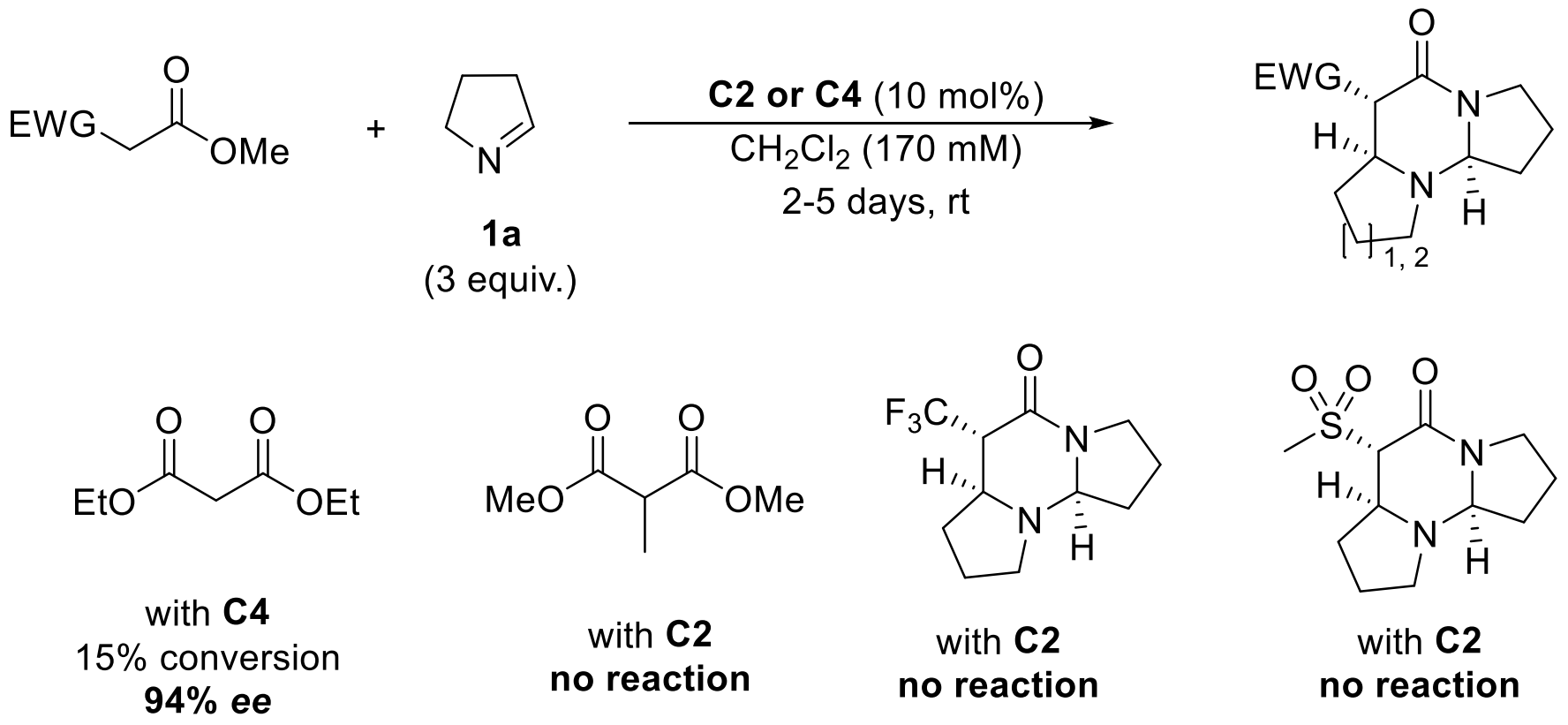

Scheme S4. Examples of C-H acidic substrates that did not react or only poorly with pyrroline

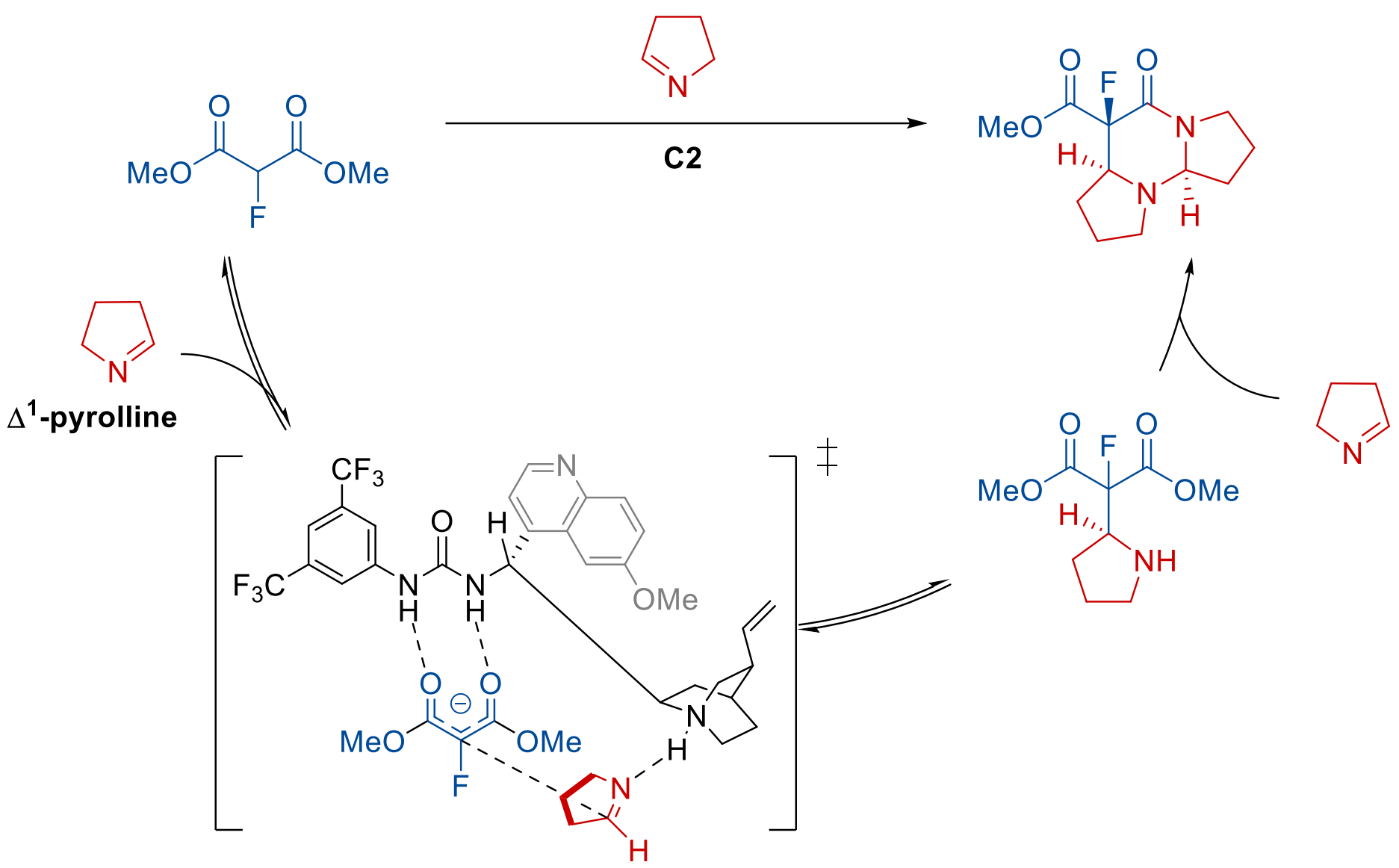

Scheme S5. Stereochemical model of the addition reaction.

Table S6: Catalyst and temperature optimization for dimethyl fluoromalonate

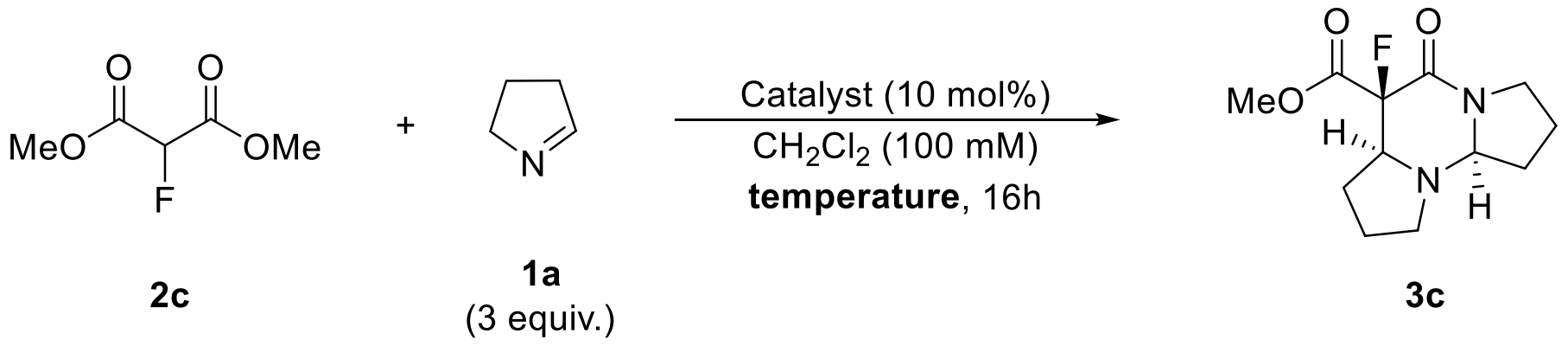

Entry	Catalyst	Temperature	Conversion to 3c [a]	*ee*
**1**	**C1**	rt	>95%	86%
**2**	**C2**	rt	>95%	88%
**3**	**C3**	rt	>95%	89%
**4**	**C4**	rt	>95%	25%
**5**	**C12**	rt	>95%	7%
**6**	**C1**	0°C	>95%	84%
**7**	**C3**	0°C	>95%	82%
**8**	**C2**	0°C	>95%	90%
**9**	**C2**	-5°C	91%	95%
**10**	**C2**	**-15°C**	**83%**	**97%**
**11**	**C2**	-30°C	64%	98%

a) Determined by ^{19}F NMR spectroscopy using Trifluorotoluene as internal standard. b) determined with chiral stationary phase HPLC.

Table S7: Catalyst and temperature optimization for the 6-6-5 ring system.

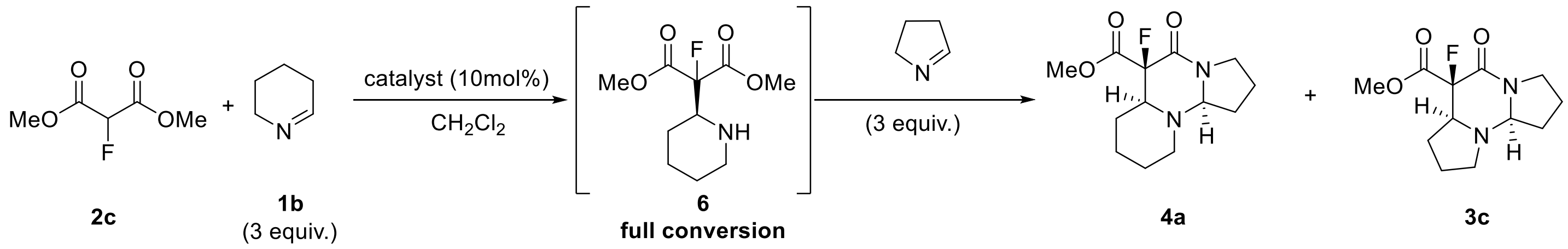

Entry	Catalyst (10 mol%)	Temperature 1st step	Temperature 2nd step	Conversion to 4a [a)]	ee [b)]	Ratio 4a:3c [a)]
**1**	**C1**	rt	rt	81%	32%	4.8:1
**2**	**C2**	rt	rt	70%	53%	3.7:1
**3**	**C3**	rt	rt	79%	30%	4.5:1
**4**	**C4**	rt	rt	80%	13%	5:1
**5**	**C12**	rt	rt	75%	<5%	5.8:1
**6**	**C2**	**-22°C**	**-20°C**	**29%**	**81%**	**1.2:1**
**7**	**C2**, 4eq. Imine	-22°C	rt	60%	86%	5:1

a) Determined by ^{19}F NMR spectroscopy using Trifluorotoluene as internal standard. b) determined with chiral stationary phase SFC.

Table S8: Additive testing for the second step forming the 6-6-5 ring system.

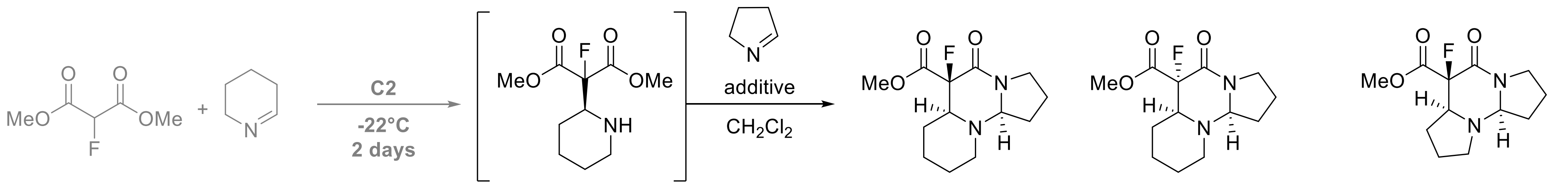

Entry	Temperature	Imine equiv.	Additive	Time	Conversion to 4a + *dia*-4a [a)]	*d.r.* (ratio 4a:*dia*-4a) [a)]	Ratio 4a:3c	*ee of 4a* [b)]
**1**	rt	4	-	2 days	65%	only 2	2:1	89%
**2**	-20°C	2	0.1 equiv. AcOH	2 days	48%	> 20:1	only 2+3	94%
**3**	-20°C	2	1 equiv. AcOH	3 days	35%	4.2:1	only 2+3	84%
**4**	rt	3	0.2 equiv. AcOH	18 h	> 90%	3.4:1	only 2+3	90%
**5**	rt	3	1 equiv. TFA	18h	65%	only 2	5.7:1	n.d.
**6**	**4°C**	**3**	**0.1 equiv. AcOH**	**18h**	**> 90%**	**10:1**	**only 2+3**	**92%**

a) Determined by ^{19}F NMR spectroscopy using Trifluorotoluene as internal standard. b) determined with chiral **stationary phase** SFC.

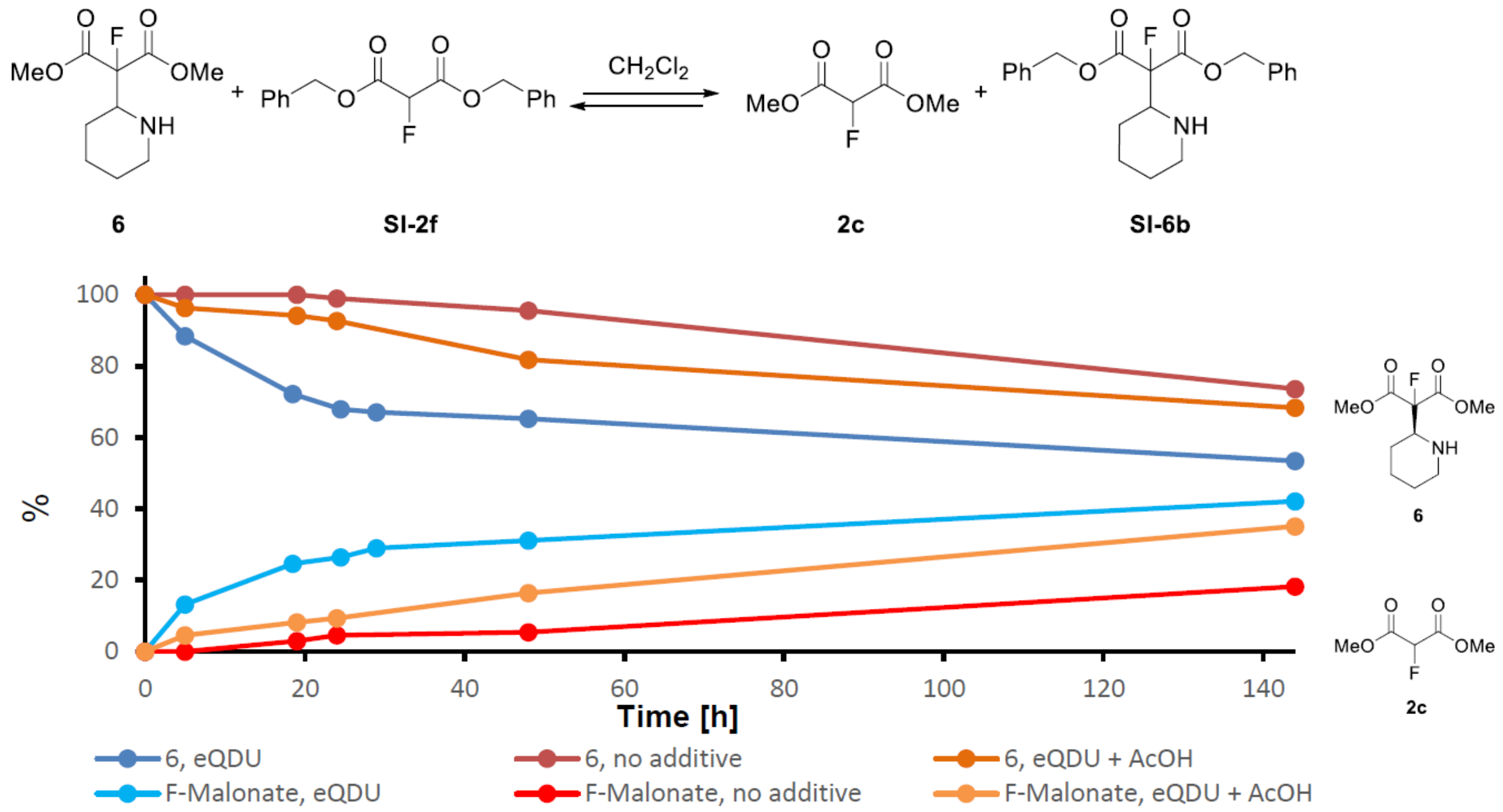

Scheme S6. Stability of the intermediate 6 in the presence of eQDU (blue), eQDU + AcOH (orange) and absence of catalyst and acid (red). The upper curves monitor the decay of 6, the lower curves the formation of 2c.

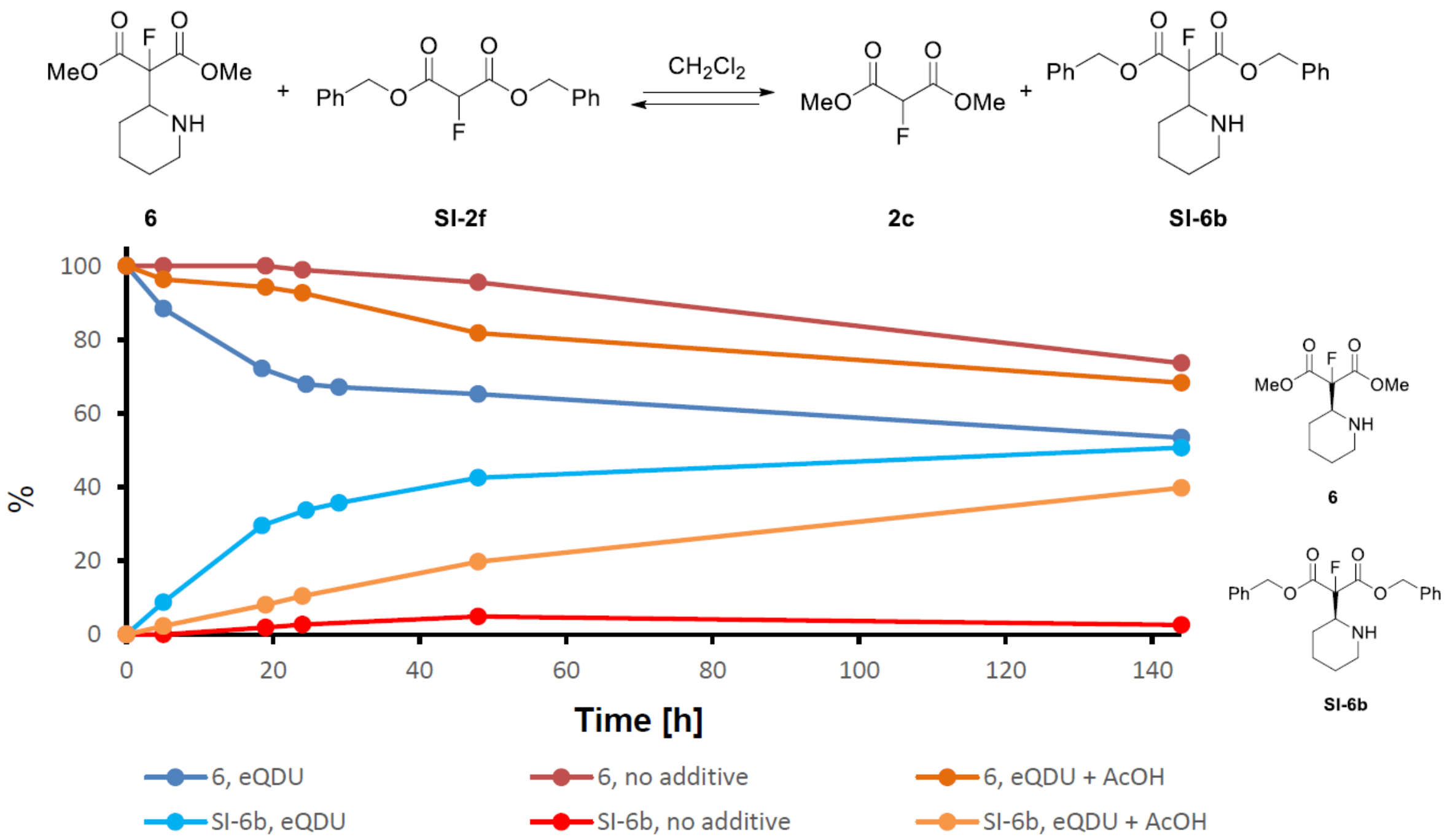

Scheme S7. Stability of the intermediate 6 in the presence of eQDU (blue), eQDU + AcOH (orange) and absence of catalyst and acid (red). The upper curves monitor the decay of 6, the lower curves the formation of SI-6b.

Table S9: Optimization of F-Malonate mono addition.

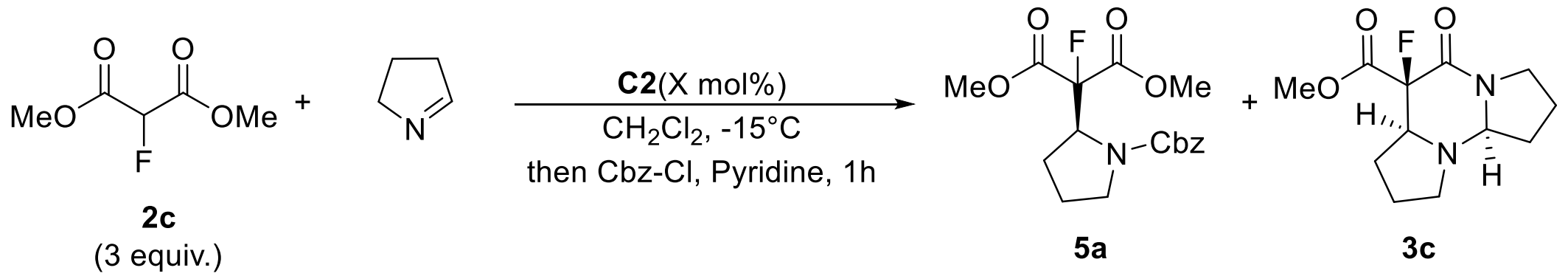

Entry	Conc.	Cat. loading	Conv. to 5a	Conv. to 3c	Ratio 5a / 3c	*ee of 5a*
**1**	100 mM	10 mol%	59%	16%	3.7	85%
**2**	**50 mM**	**10 mol%**	**63%**	**16%**	**3.9**	**89%**
**3**	200 mM	10 mol%	58%	20%	2.9	82%
**4**	200 mM	5 mol%	56%	19%	2.9	77%

a) Determined by ^{19}F NMR spectroscopy using Trifluorotoluene as internal standard. b) determined with chiral stationary phase SFC.

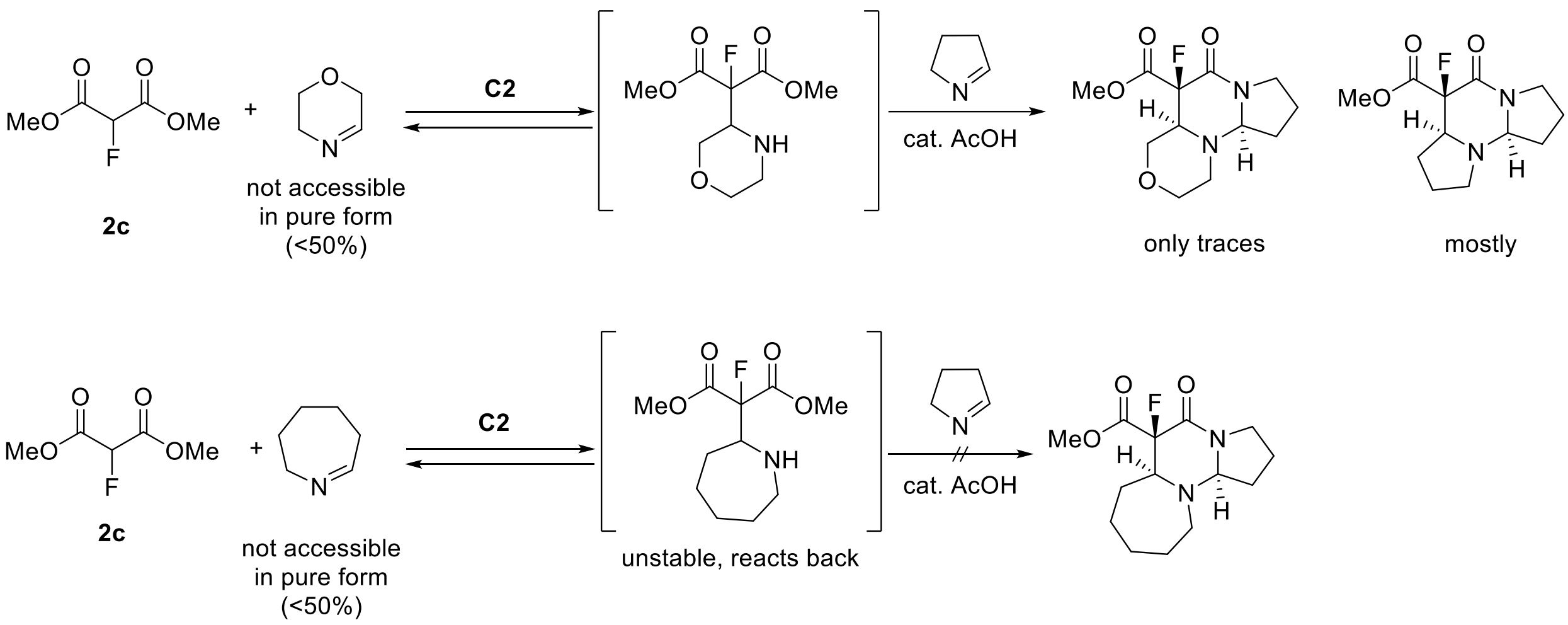

Scheme S8. Imines that did not yield addition products in high yields and enantioselectivity**.** Of note, imines derived from azepane and morpholine are known to be difficult to access pure (ref. 6).

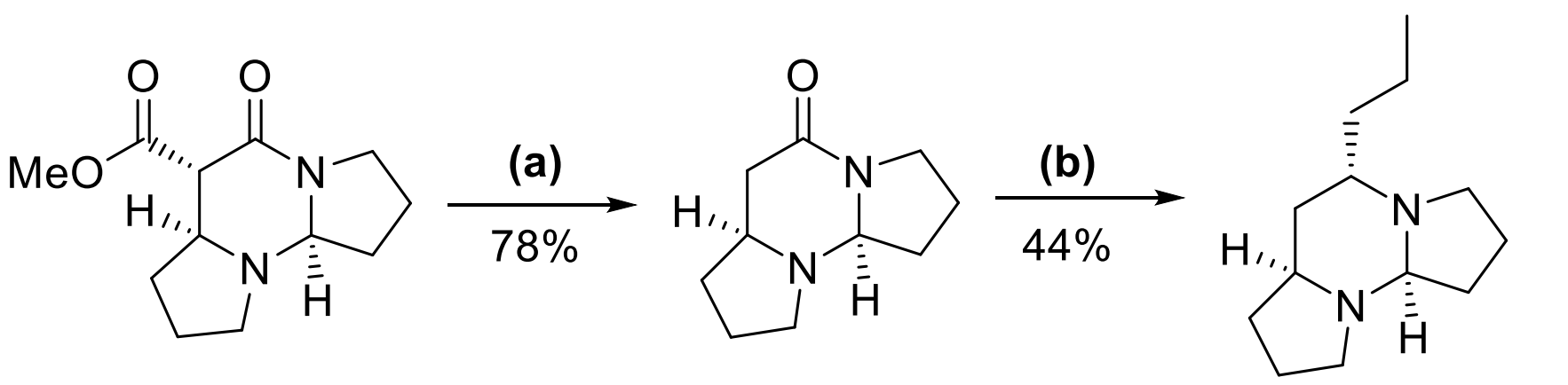

3a 91% *ee* | **ent-7** 91% *ee* | ***ent*-Tetraponerine T1** 15:1 d.r.

Scheme S9. Total synthesis of Tetraponerine **T-1**. (a) NaOH (aq.,1 M), THF, 1 h, rt then HCl (1 M, aq.), 1 h, 80 °C, 78% over two steps (b) Vaska's catalyst ($IrCl(CO)[PPh_3]_2$, 1 mol%), TMDS, CH_2Cl_2, 20 min, rt then then -78°C; *n*PrMgBr, 4 h, -78 °C to rt, 44%.

3 Synthesis of starting materials

3.1 Synthesis of pyrroline 1a [45]

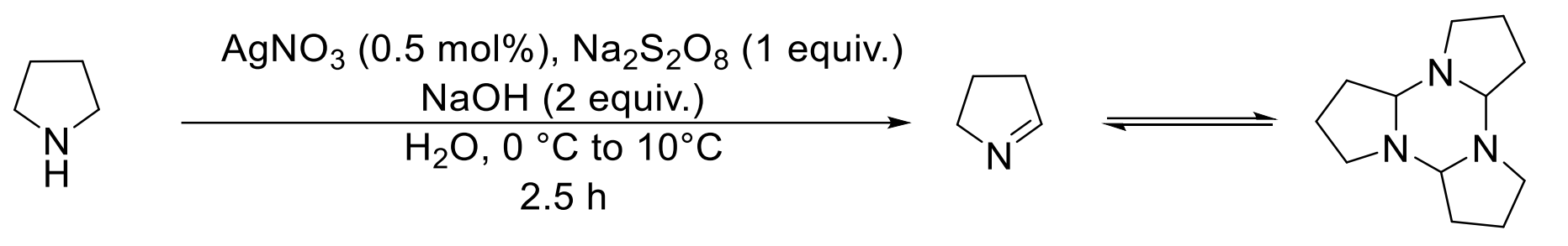

Δ1-pyrroline Δ3-pyrroline

Pyrrolidine (12.0 mL, 146 mmol, 1.0 equiv.) was dissolved in water (140 mL) and then NaOH (12.0 g, 300 mmol, 2.05 equiv.) was added. After complete dissolution, an aqueous solution of $AgNO_3$ (127.8 mg, 0.75 mmol, 1 mol% in 10 mL of water) was added dropwise via syringe. The reaction mixture was cooled with a water/ice bath and an aqueous solution of $Na_2S_2O_8$ (37.6 g, 158 mmol, 1.08 equiv. in 150 mL of water) was added dropwise over 15 min. The reaction was stirred for additional 2h in the water/ice bath. Afterwards, NaCl (5 g) was added to precipitate AgCl. The reaction mixture was filtered through a paper filter. The filtrate was transferred into a separatory funnel, extracted with CH_2Cl_2 (3 x 125 mL) and dried over Na_2SO_4. The solvent was evaporated at room temperature.

Neutral aluminum oxide (4 g) and 50 mL Et_2O (50 mL) were added, and the mixture was filtered through a fritted funnel packed with neutral aluminum oxide (1 cm diameter, circa 4 cm high). The solvent was removed at room temperature (down to 20 mbar). Pyrroline (5.4 g, 78.1 mmol, 54 %) was obtained as a pale yellow oil.

Pyrroline can be further purified by distillation under atmospheric pressure. Afterwards it contained again pyrrolidine, which can be removed through filtration through neutral aluminum oxide with Et_2O.

^{1}H NMR (400 MHz, $CDCl_3$) δ = 3.07 – 2.94 (m, 6H), 2.31 (m, 3H), 1.87 (m, 6H), 1.79 – 1.62 (m, 6H).

^{13}C NMR (101 MHz, $CDCl_3$) δ = 82.2, 46.1, 28.1, 20.5.

3.2 Synthesis of piperine from chloramines

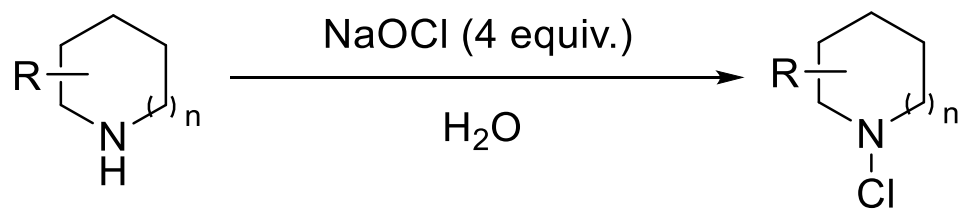

General procedure 1: Bleach (14% active Cl_2, 42.8 g, 80.4 mmol, 4.0 equiv.) was diluted with water (20 mL). Then the respective amine (20.1 mmol) was added slowly dropwise within 15 min. The reaction mixture was stirred for 15 min and then transferred to a separatory funnel. The aqueous phase was extracted with Et_2O (3 x 40 mL) and the solvent was evaporated at room temperature.

The obtained compounds are unstable and may decompose upon storage. Therefore, they were used directly in the next step.

1-Chloropiperidine (SI-1a):

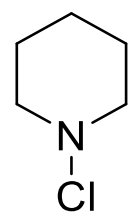

Compound **SI-1a** was prepared according to **General Procedure 1** starting from piperidine (10 mL, 101.2 mmol, 1.0 equiv.) and bleach (215 g, 243.5 mmol, 4.0 equiv.). The product was obtained as a colorless liquid (10.41, 87.1 mmol, 86 yield). The analytical data is in agreement with reported data. [46]

^{1}H NMR (400 MHz, $CDCl_3$) δ = 3.63 – 2.73 (br. s, 4H), 1.74 (m, 4H), 1.47 (br. s, 2H).

^{13}C NMR (101 MHz, $CDCl_3$) δ = 63.9, 27.6, 22.9.

1-Chloromorpholine (SI-1b):

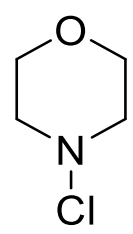

Compound **SI-1b** was prepared according to **General Procedure 1** starting from morpholine (9.5 mL, 110 mmol, 1.0 equiv.) and bleach (234 g, 440 mmol, 4.0 equiv.). The product was obtained as a colorless liquid (7.64 g, 62.9 mmol, 57% yield). The analytical data is in agreement with reported data. [46]

^{1}H NMR (400 MHz, $CDCl_3$) δ = 3.78 (s, 2H), 3.21 (s, 2H).

^{13}C NMR (101 MHz, $CDCl_3$) δ = 67.6, 62.9.

1-Chloroazepane (SI-1c):

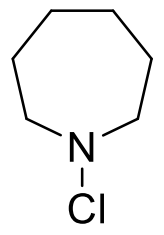

Compound **SI-1c** was prepared according to **General Procedure 1** starting from azepane (4.5 mL, 39.9 mmol, 1.0 equiv.) and bleach (91.4 g, 159.6 mmol, 4.0 equiv.). The product was obtained as a colorless liquid (4.91, 36.8 mmol, 92% yield). The analytical data is in agreement with reported data. [46]

^{1}H NMR (400 MHz, $CDCl_3$) δ = 3.37 – 3.22 (m, 4H), 1.76 (p, *J* = 5.2, 4.3 Hz, 4H), 1.70 – 1.60 (m, 4H).

^{13}C NMR (101 MHz, $CDCl_3$) δ = 65.7, 26.8, 25.8.

Synthesis of piperideine 1b:

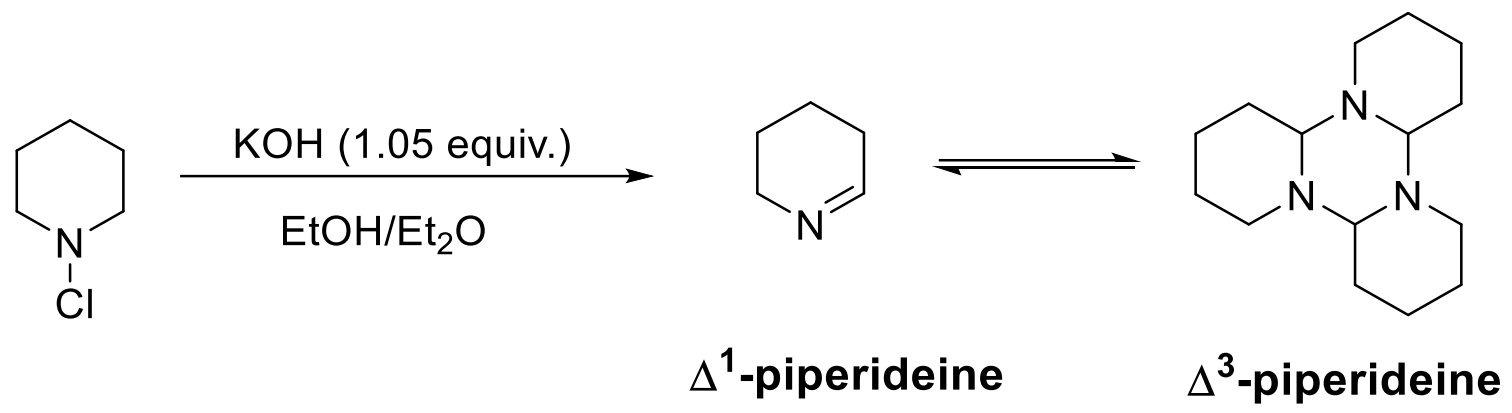

KOH (985 mg, 17.6 mmol, 1.05 equiv.) was dissolved in absolute EtOH (34 mL, 0.5 M). In a separate flask, Cl-Piperidine (2.0 g, 16.7 mmol, 1.0 equiv.) was dissolved in Et_2O (36 mL, 0.46 M). The chloramine solution was added dropwise to the ethanolic KOH solution which was cooled to room temperature with a water bath. The reaction was stirred at rt for 24 h.

The resulting precipitate (KCl) was filtered off and the filtrate was concentrated under reduced pressure at room temperature. The residue was dissolved in water (30 mL) and the aqueous phase was extracted with Et_2O (3 x 125 mL). The combined organic layers were dried over Na_2SO_4 and concentrated under reduced pressure. Upon standing, the compound slowly crystallized to afford piperideine (866 mg, 10.4 mmol, 62%) as a light yellow solid.

The analytical data is in agreement with reported data. [7]

Δ^3-piperideine:

^{1}H NMR (400 MHz, $CDCl_3$) δ = 3.19 – 3.09 (m, 3H), 2.85 – 2.78 (m, 3H), 2.08 – 1.97 (m, 3H), 1.82 – 1.63 (m, 9H), 1.63 – 1.51 (m, 6H), 1.39 – 1.22 (m, 3H).

^{13}C NMR (101 MHz, $CDCl_3$) δ = 81.9, 46.4, 29.2, 25.8, 22.3.

Visible peaks of **Δ^1-piperideine** after 6h, other peaks overlap with the trimer:

^{1}H NMR (400 MHz, $CDCl_3$) δ = 7.82 (m, 1H), 3.67 – 3.52 (m, 2H), 2.18 (m, 2H).

^{13}C NMR (101 MHz, $CDCl_3$) δ = 163.2, 49.3, 28.8, 22.4, 18.7.

3.3 Synthesis of Malonates

General Procedure 2: To a solution of DMAP (0.1 equiv.) in CH_2Cl_2 (0.5 M) were added methyl malonyl chloride (1.05 equiv.) and the respective alcohol or thiol (1.0 equiv.). Pyridine (1.2 equiv.) was added dropwise and cooling was continued for 30 min. The ice-bath was removed and the reaction was stirred overnight at room temperature.

The reaction was quenched by addition of 0.5 M HCl and extracted with CH_2Cl_2 (2 x). The combined organic phases were washed with semi-saturated $NaHCO_3$ (1x) and dried over Na_2SO_4. The crude product was used without further purification or purified by column chromatography as indicated.

Methyl 3-((4-methoxyphenyl)thio)-3-oxopropanoate (SI-2a):

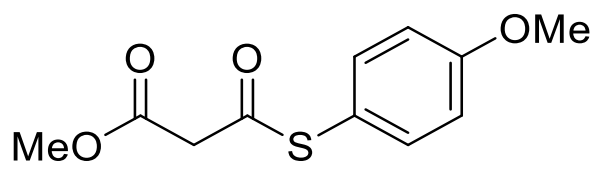

Compound **SI-2a** was prepared according to **General Procedure 2** starting from *p*-methoxythiophenol (15.6 mL, 126.5 mmol, 1.0 equiv.) and methyl malonyl chloride (15 mL, 139 mmol, 1.1 equiv.). The product was purified by column chromatography (silica, gradient elution from hexane to hexanes:EtOAc 1:1) and isolated as a colorless oil (20.1 g, 83.7 mmol, 66% yield).

^{1}H NMR (400 MHz, $CDCl_3$): δ = 7.39 – 7.30 (m, 2H), 7.00 – 6.90 (m, 2H), 3.82 (s, 3H), 3.76 (s, 3H), 3.65 (s, 2H).

^{13}C NMR (101 MHz, $CDCl_3$): δ = 190.6, 166.4, 161.0, 136.1, 117.5, 115.0, 55.4, 52.7, 48.6.

HRMS (ESI): *m/z* calc. for $C_{11}H_{12}NaO_4S$ $[M+Na]^+$: 263.0349, found: 263.0346.

IR (neat) v_{max}(cm^{-1}): 2951, 1736, 1696, 1591, 1493, 1289, 1245, 1172, 1024, 973, 825, 665.

Methyl (2,2,2-trifluoroethyl) malonate (SI-2b):

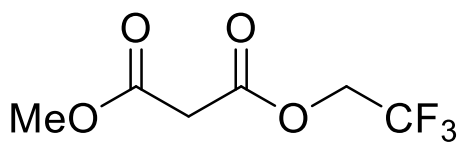

Compound **SI-2b** was prepared according to **General Procedure 2** starting from trifluoroethanol (360 µL, 5.0 mmol, 1.0 equiv.) and methyl malonyl chloride (564 µL, 5.25 mmol, 1.05 equiv.). The product was pure as judged by NMR analysis after aqueous extraction and obtained as a colorless oil (0.98 g, 4.9 mmol, 98% yield).

^{1}H NMR (400 MHz, $CDCl_3$) δ = 4.56 (q, *J* = 8.3 Hz, 2H), 3.79 (s, 3H), 3.52 (s, 2H).

^{19}F NMR {^{1}H} (377 MHz, $CDCl_3$) δ = -73.80.

^{19}F NMR (377 MHz, $CDCl_3$) δ = -73.80 (t, *J* = 8.2 Hz).

^{13}C NMR (101 MHz, $CDCl_3$) δ = 166.0, 165.0, 122.6 (q, *J* = 279.6 Hz), 61.0 (q, *J* = 37.0 Hz), 52.8, 40.6.

HRMS (ESI): *m/z* calc. for $C_6H_7F_3NaO_4$ $[M+Na]^+$ 223.0189, found 223.0191.

IR (neat) v_{max}(cm^{-1}): 2961, 2359, 1740, 1277, 1134, 1055, 979, 842.

Methyl prop-2-yn-1-yl malonate (SI-2c):

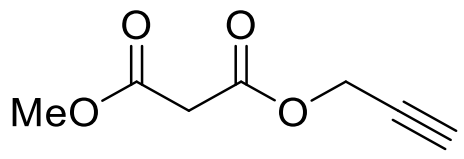

Compound **SI-2c** was prepared according to **General Procedure 2** starting from propargyl alcohol (288 μL, 5.0 mmol, 1.0 equiv.) and methyl malonyl chloride (564 μL, 5.25 mmol, 1.05 equiv.). The product was pure as judged by NMR analysis after aqueous extraction and obtained as a colorless oil (0.64 g, 4.1 mmol, 82% yield). The analytical data is in agreement with reported data. [47]

^{1}H NMR (400 MHz, $CDCl_3$) δ = 4.77 (d, *J* = 2.5 Hz, 2H), 3.78 (s, 3H), 3.46 (s, 2H), 2.53 (t, *J* = 2.5 Hz, 1H).

^{13}C NMR (101 MHz, $CDCl_3$) δ = 166.5, 165.7, 76.9, 75.5, 52.9, 52.6, 41.0.

HRMS (ESI): *m/z* calc. for $C_7H_8NaO_4$ $[M+Na]^+$ 179.0315, found 179.0313.

IR (neat) ν_{max}(cm^{-1}): 3282, 2955, 2129, 1732, 1437, 1334, 1270, 1202, 1142, 1022, 679.

Allyl methyl malonate (SI-2d):

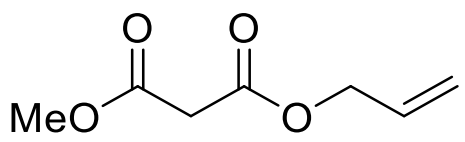

Compound **SI-2d** was prepared according to **General Procedure 2** starting from allyl alcohol (1.0 mL, 14.6 mmol, 1.0 equiv.) and methyl malonyl chloride (1.65 mL, 15.4 mmol, 1.05 equiv.). The product was pure as judged by NMR analysis after aqueous extraction and obtained as a colorless oil (1.6 g, 10.1 mmol, 69% yield).

^{1}H NMR (400 MHz, $CDCl_3$) δ = 5.99 – 5.84 (m, 1H), 5.34 (dt, *J* = 17.2, 1.7 Hz, 1H), 5.26 (dt, *J* = 10.4, 1.3 Hz, 1H), 4.65 (ddd, *J* = 5.4, 1.9, 1.7 Hz, 2H), 3.75 (s, 3H), 3.42 (s, 2H).

^{13}C NMR (101 MHz, $CDCl_3$) δ = 166.9, 166.1, 131.5, 118.7, 66.1, 52.5, 41.3.

HRMS (ESI): *m/z* calc. for $C_7H_{10}NaO_4$ $[M+Na]^+$ 181.0471, found 181.0469.

IR (neat) ν_{max}(cm^{-1}): 2955, 1730, 1437, 1332, 1270, 1197, 1142, 990, 931, 849.

4 Substrate Scope

4.1 General Procedure for reaction with pyrroline

General Procedure 3: Synthesis of Racemates

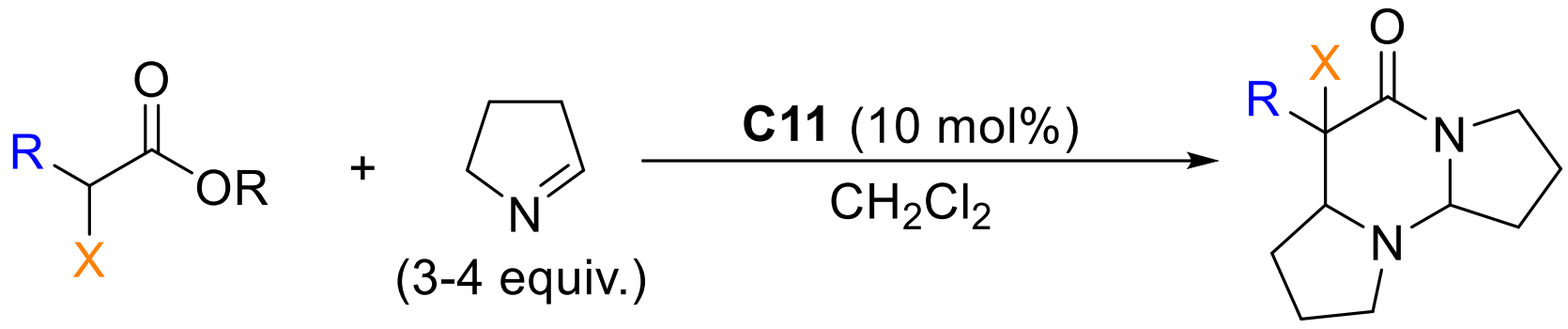

2 1a 3

Racemates were prepared on a 0.2 mmol scale. A glass vial was charged with achiral catalyst **C11** (10 mol%, 5.7 mg, 0.02 mmol) and CH_2Cl_2 (2 mL). The respective malonate **2** (1 equiv., 0.2 mmol) was added, followed by pyrroline **1a** (3-4 equiv.). The reaction mixture was stirred for 16-120 h at room temperature. The solvent was removed under reduced pressure and the crude mixture was purified by automated flash column chromatography (elution gradient from CH_2Cl_2 to CH_2Cl_2/MeOH 10:1) to yield the racemic product after evaporation of the solvents.

General Procedure 4: Enantioselective addition reactions to pyrroline

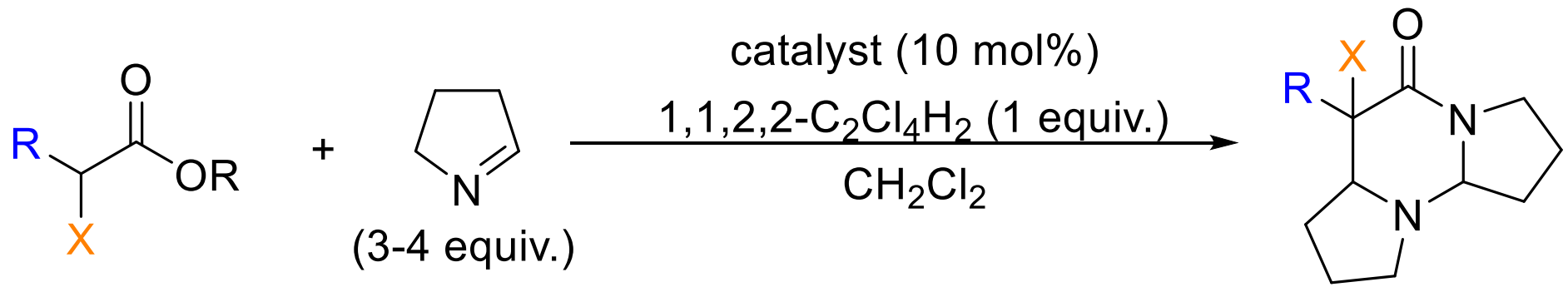

2 1a 3

A small glass vial (3 to 5 mL) was charged with catalyst **C4** (10 mol%) and CH_2Cl_2 (c = 0.17 M). Then the respective malonate **2** was added (1 equiv.), followed by pyrroline **1a** (50.3 µL, 0.8 mmol, 4 equiv.). After 48h, the solvent was removed under reduced pressure and the crude mixture was purified by automated flash column chromatography to yield the product **3a** after evaporation of the solvents. The enantiomeric excess was determined by chiral stationary phase HPLC or SFC.

4.2 Analytical Data of 5-6-5 products

Methyl (6*S*,6*aS*,10*aS*)-5-oxodecahydrodipyrrolo[1,2-*a*:1',2'-*c*]pyrimidine-6-carboxylate (3a)

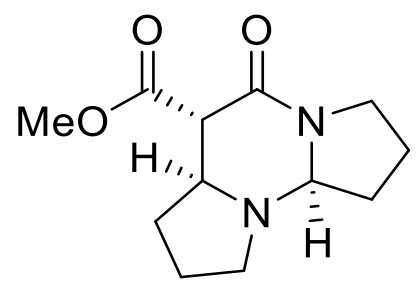

Compound **3a** was prepared according to **General Procedure 4** starting from dimethyl malonate **2a** (46 µL, 0.4 mmol, 1.0 equiv.) and pyrroline **1a** (100 µL, 1.60 mmol, 4.0 equiv.) with a reaction time of 48 h at room temperature. **C4** (25.3 mg, 0.04 mmol, 10 mol%) was used as catalyst. The product was purified by column chromatography (silica, elution gradient from CH_2Cl_2 to CH_2Cl_2:MeOH 90:10) and isolated as a colorless oil (92.5 mg, 0.388 mmol, 97% yield, 92% *ee*).

^{1}H NMR (500 MHz, $CDCl_3$) δ = 3.91 (dd, *J* = 9.7, 5.1 Hz, 1H), 3.76 (s, 3H), 3.67 – 3.59 (m, 1H), 3.55 – 3.49 (m, 1H), 3.25 (dq, *J* = 10.1, 0.8 Hz, 1H), 3.06 (td, *J* = 8.7, 3.0 Hz, 1H), 2.87 (ddd, *J* = 10.1, 8.8, 6.7 Hz, 1H), 2.33 (q, *J* = 8.9 Hz, 1H), 2.13 (ddddd, *J* = 11.0, 6.7, 5.1, 1.4, 0.7 Hz, 1H), 2.10 – 1.79 (m, 5H), 1.72 – 1.63 (m, 1H), 1.63 – 1.56 (m, 1H).

^{13}C NMR (126 MHz, $CDCl_3$) δ = 170.2, 163.9, 78.1, 64.3, 55.6, 52.6, 49.2, 45.2, 32.5, 30.1, 22.1, 21.1.

HRMS (ESI): *m/z* calc. for $C_{12}H_{19}N_2O_3$ [M+Na]$^+$ 239.1390, found 239.1394.

IR (neat) ν_{max}(cm^{-1}): 2952, 1738, 1645, 1435, 1261, 1197, 1155, 1019.

R*f* = 0.4 (5% MeOH in CH_2Cl_2).

Chiral stationary phase HPLC: IBN-5 column, hexane/*i*-PrOH 70:30, 0.5 mL/min, 25 °C, 210 nm; 18.3 min (minor), 22.2 min (major).

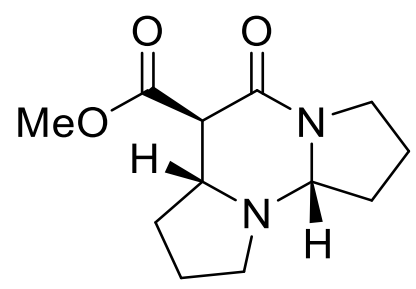

Compound *ent*-**3a** was prepared according to **General Procedure 4** starting from dimethyl malonate **2a** (57.4 µL, 0.5 mmol, 1.0 equiv.) and pyrroline **1a** (127 µL, 2.0 mmol, 4.0 equiv.) with a reaction time of 48 h at room temperature. **C9** (30 mg, 0.05 mmol, 10 mol%) was used as catalyst. The product was purified by column chromatography (silica, gradient elution from CH_2Cl_2 to CH_2Cl_2:MeOH 90:10) and isolated as a colorless oil (118.5 mg, 0.497 mmol, 99% yield, 93% *ee*).

The NMR, HRMS and IR data are the same as for **3a**.

Chiral stationary phase HPLC: IBN-5 column, hexane/*i*-PrOH 70:30, 0.5 mL/min, 25 °C, 210 nm; 17.1 min (major), 21.8 min (minor).

Benzyl (6*S*,6a*S*,10a*S*)-5-oxodecahydrodipyrrolo[1,2-*a*:1',2'-*c*]pyrimidine-6-carboxylate (3b)

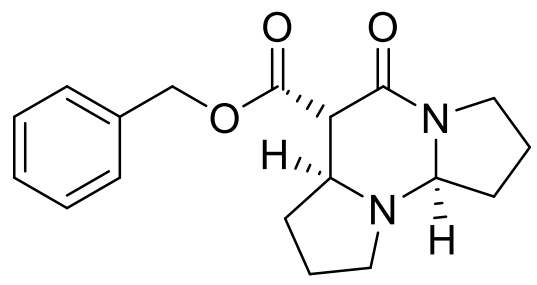

Compound **3b** was prepared according to **General Procedure 4** starting from dibenzyl malonate **2b** (50 μL, 0.2 mmol, 1.0 equiv.) and pyrroline **1a** (50.3 μL, 0.80 mmol, 4.0 equiv.) with a reaction time of 5 days at room temperature. **C4** (12.7 mg, 0.02 mmol, 10 mol%) was used as catalyst in this reaction. The product was purified by column chromatography (silica, gradient elution from CH_2Cl_2 to CH_2Cl_2:MeOH 90:10) and isolated as a colorless wax (59.7 mg, 0.190 mmol, 95% yield, 97% *ee*).

^{1}H NMR (400 MHz, $CDCl_3$) δ = 7.45 – 7.30 (m, 5H), 5.24 (s, 2H), 3.92 (dd, *J* = 9.8, 5.0 Hz, 1H), 3.72 – 3.60 (m, 1H), 3.60 – 3.47 (m, 1H), 3.32 (d, *J* = 10.2 Hz, 1H), 3.07 (td, *J* = 8.5, 3.1 Hz, 1H), 2.89 (ddd, *J* = 10.1, 8.7, 6.6 Hz, 1H), 2.34 (q, *J* = 8.7 Hz, 1H), 2.21 – 2.10 (m, 1H), 2.09 – 1.77 (m, 5H), 1.76 – 1.53 (m, 2H).

^{13}C NMR (101 MHz, $CDCl_3$) δ = 169.5, 163.7, 135.7, 128.5, 128.2, 128.1, 78.0, 67.0, 64.2, 55.6, 49.1, 45.0, 32.4, 29.9, 22.0, 21.0.

HRMS (ESI): *m/z* calc. for $C_{18}H_{23}N_2O_3$ $[M+Na]^+$ 315.1703, found 315.1703.

IR (neat) ν_{max}(cm^{-1}): 2951, 2807, 1733, 1646, 1439, 1236, 1143, 1015, 748, 698.

Chiral stationary phase HPLC: IBN-5 column, hexane/*i*-PrOH 70:30, 0.5 mL/min, 25 °C, 210 nm; 18.3 min (minor), 22.2 min (major).

Methyl (6*R*,6a*S*,10a*S*)-6-fluoro-5-oxodecahydrodipyrrolo[1,2-*a*:1',2'-c]pyrimidine-6-carboxylate (3c)

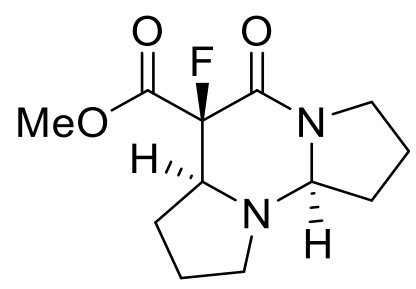

Compound **3c** was prepared according to **General Procedure 4** starting from dimethyl fluoromalonate **2c** (34.6 μL, 0.3 mmol, 1.0 equiv.) and pyrroline **1a** (56.5 μL, 0.90 mmol, 3.0 equiv.) with a reaction time of 24 h at –15°C. **C2** (17.4 mg, 0.03 mmol, 10 mol%) was used as catalyst. The product was purified by column chromatography (silica, gradient elution from CH_2Cl_2 to CH_2Cl_2:MeOH 90:10) and isolated as a white solid (70.3 mg, 0.274 mmol, 91% yield, 97% *ee*).

^{1}H NMR (500 MHz, $CDCl_3$) δ = 3.92 (ddd, *J* = 9.2, 5.2, 3.9 Hz, 1H), 3.83 (s, 3H), 3.73 – 3.64 (m, 1H), 3.57 – 3.49 (m, 1H), 3.13 (td, *J* = 8.3, 2.7 Hz, 1H), 3.03 (dt, *J* = 24.0, 8.1 Hz, 1H), 2.33 (qd, *J* = 8.6, 1.1 Hz, 1H), 2.20 – 2.12 (m, 1H), 2.11 – 2.02 (m, 2H), 2.00 – 1.74 (m, 4H).

^{19}F NMR {^{1}H} (471 MHz, $CDCl_3$) δ = -169.5.

^{19}F NMR (471 MHz, $CDCl_3$) δ = -169.5 (dd, *J* = 23.8, 3.5 Hz).

^{13}C NMR (126 MHz, $CDCl_3$) δ = 167.7 (d, *J* = 26.7 Hz), 161.3 (d, *J* = 24.2 Hz), 90.6 (d, *J* = 198.9 Hz), 78.5, 67.3 (d, *J* = 20.5 Hz), 53.2 (d, *J* = 1.0 Hz), 49.5, 45.1, 32.2, 22.3 (d, *J* = 7.1 Hz), 22.1, 20.9.

HRMS (ESI): *m/z* calc. for $C_{12}H_{18}FN_2O_3$ $[M+Na]^+$ 257.1296, found 257.1296.

IR (neat) ν_{max}(cm^{-1}): 2956, 1765, 1660, 1439, 1268, 1086, 786.

Chiral stationary phase HPLC: AD column, hexane/*i*-PrOH 85:15, 0.5 mL/min, 25 °C, 210 nm; 20.3 min (major), 21.5 min (minor).

Methyl (6*S*,6*aR*,10*aR*)-6-fluoro-5-oxodecahydrodipyrrolo[1,2-*a*:1',2'-*c*]pyrimidine-6-carboxylate (*ent*-**3c**)

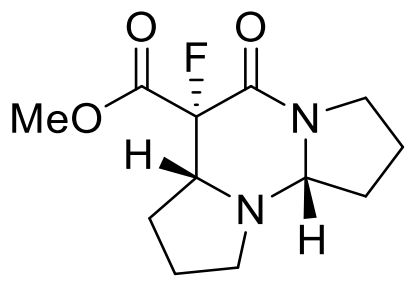

Compound *ent*-**3c** was prepared according to **General Procedure 4** starting from dimethyl fluoromalonate **2c** (34.6 µL, 0.3 mmol, 1.0 equiv.) and pyrroline **1a** (56.5 µL, 0.90 mmol, 3.0 equiv.) with a reaction time of 24 h at –15°C. **C7** (17.4 mg, 0.03 mmol, 10 mol%) was used as catalyst. The product was purified by column chromatography (silica, gradient elution from CH_2Cl_2 to CH_2Cl_2:MeOH 90:10) and isolated as a white solid (70.1 mg, 0.274 mmol, 91% yield, 95% *ee*).

The NMR, HRMS and IR data are the same as for *ent*-**3c**.

Chiral stationary phase HPLC: AD column, hexane/*i*-PrOH 85:15, 0.5 mL/min, 25 °C, 210 nm; 20.8 min (minor), 21.7 min (major).

Benzyl (6*R*,6*aS*,10*aS*)-6-fluoro-5-oxodecahydrodipyrrolo[1,2-*a*:1',2'-*c*]pyrimidine-6-carboxylate (**3d**)

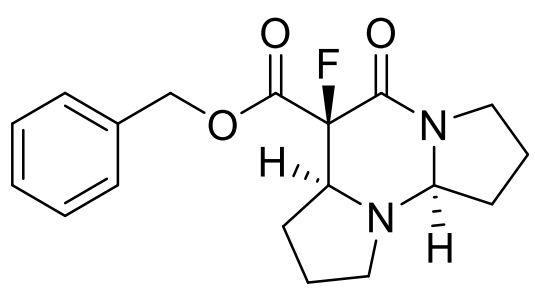

Compound **3d** was prepared according to **General Procedure 4** starting from dibenzyl fluoromalonate **2d** (90.7 mg, 0.3 mmol, 1.0 equiv.) and pyrroline **1a** (56.5 µL, 0.90 mmol, 3.0 equiv.) with a reaction time of 4 days at –15°C. **C2** (17.4 mg, 0.03 mmol, 10 mol%) was used as catalyst. The product was purified by column chromatography (silica, gradient elution from CH_2Cl_2 to CH_2Cl_2:MeOH 90:10) and isolated as a white wax (97.2 mg, 0.292 mmol, 98% yield, 94% *ee*).

^{1}H NMR (400 MHz, $CDCl_3$) δ = 7.46 – 7.30 (m, 5H), 5.39 – 5.26 (m, 2H), 4.00 – 3.88 (m, 1H), 3.80 – 3.67 (m, 1H), 3.61 – 3.50 (m, 1H), 3.15 (td, *J* = 8.4, 2.6 Hz, 1H), 3.04 (dt, *J* = 23.8, 8.1 Hz, 1H), 2.34 (qd, *J* = 8.6, 1.1 Hz, 1H), 2.25 – 2.14 (m, 1H), 2.14 – 1.63 (m, 6H).

^{19}F NMR {^{1}H} (471 MHz, $CDCl_3$) δ -169.5.

^{19}F NMR (377 MHz, $CDCl_3$) δ = -169.30 (dd, *J* = 23.9, 3.6 Hz).

^{13}C NMR (101 MHz, $CDCl_3$) δ = 167.1 (d, *J* = 26.8 Hz), 161.3 (d, *J* = 24.2 Hz), 135.0, 128.6, 128.5, 128.2, 90.6 (d, *J* = 199.3 Hz), 78.5, 67.8, 67.3 (d, *J* = 20.6 Hz), 49.5, 45.1, 32.2, 22.2 (d, *J* = 6.9 Hz), 22.1, 20.9.

HRMS (ESI): *m/z* calc. for $C_{18}H_{22}FN_2O_3$ $[M+Na]^+$ 333.1609, found 333.1602.

IR (neat) v_{max}(cm^{-1}): 2954, 2812, 1763, 1659, 1443, 1277, 1084, 915, 730, 697.

Chiral stationary phase HPLC: AD column, hexane/*i*-PrOH 80:20, 0.5 mL/min, 25 °C, 210 nm; 23.1 min (major), 28.8 min (minor).

Methyl (6*R*,6*aS*,10*aS*)-6-chloro-5-oxodecahydrodipyrrolo[1,2-*a*:1',2'-*c*]pyrimidine-6-carboxylate (3e)

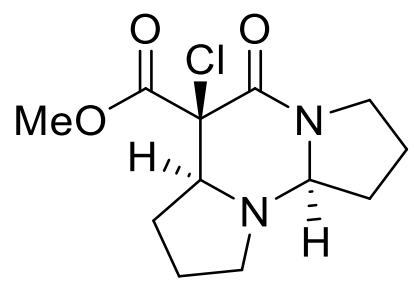

Compound **3e** was prepared according to **General Procedure 4** starting from dimethyl chloromalonate **2e** (38.4 μL, 0.3 mmol, 1.0 equiv.) and pyrroline **1a** (56.5 μL, 0.90 mmol, 3.0 equiv.) using pure tetrachloroethane as solvent with a reaction time of 40 h at room temperature. **C2** (17.4 mg, 0.03 mmol, 10 mol%) was used as catalyst. The product was purified by column chromatography (silica, gradient elution from CH_2Cl_2 to CH_2Cl_2:MeOH 90:10) and isolated as a white wax (73.7 mg, 0.270 mmol, 90% yield, 91% *ee*).

^{1}H NMR (500 MHz, $CDCl_3$) δ = 3.9 (s, 4H), 3.8 – 3.7 (m, 1H), 3.6 – 3.5 (m, 1H), 3.3 – 3.2 (m, 1H), 3.2 (t, *J* = 8.0 Hz, 1H), 2.3 (ddd, *J* = 9.6, 8.7, 7.9 Hz, 1H), 2.2 – 2.0 (m, 3H), 2.0 – 1.8 (m, 5H).

^{13}C NMR (126 MHz, $CDCl_3$) δ = 167.3, 162.5, 79.1, 72.0, 68.3, 53.9, 50.6, 45.4, 32.5, 25.1, 22.5, 20.7.

HRMS (ESI): *m/z* calc. for $C_{12}H_{18}ClN_2O_3$ $[M+Na]^+$ 273.1000, found 273.1005.

IR (neat) v_{max}(cm^{-1}): 2953, 1757, 1656, 1435, 1235, 1074, 1022, 694.

Chiral stationary phase HPLC: AD column, hexane/*i*-PrOH 80:20, 0.5 mL/min, 25 °C, 210 nm; 14.8 min (major), 16.0 min (minor).

(6*S*,6*aS*,10*aS*)-6-Nitrooctahydrodipyrrolo[1,2-*a*:1',2'-*c*]pyrimidin-5(6H)-one (3f)

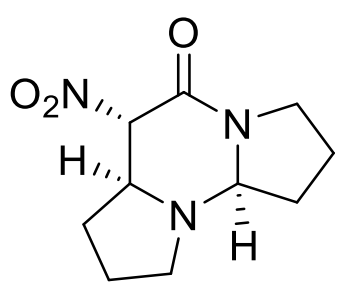

Compound **3f** was prepared according to **General Procedure 4** starting from methyl nitroacetate **2f** (18.5 μL, 0.2 mmol, 1.0 equiv.) and pyrroline **1a** (37.7 μL, 0.60 mmol, 3.0 equiv.) with a reaction time of 24 h at –15°C. **C2** (11.6 mg, 0.02 mmol, 10 mol%) was used as catalyst. The product was purified by column chromatography (silica, gradient elution from CH_2Cl_2 to CH_2Cl_2:MeOH 90:10) and isolated as a brown oil (11.2 mg, 0.05 mmol, 25% yield, 14% *ee*).

^{1}**H NMR** (400 MHz, $CDCl_3$) δ = 4.96 (d, *J* = 9.6, 0.8 Hz, 1H), 4.07 (dd, *J* = 10.0, 5.0 Hz, 1H), 3.73 – 3.66 (m, 1H), 3.66 – 3.56 (m, 1H), 3.21 (m, *J* = 9.7, 8.2, 7.0 Hz, 1H), 3.12 (td, *J* = 8.5, 3.2 Hz, 1H), 2.47 (q, *J* = 8.7 Hz, 1H), 2.27 – 2.15 (m, 2H), 2.12 – 2.05 (m, 1H), 2.04 – 1.88 (m, 2H), 1.85 – 1.77 (m, 1H), 1.75 – 1.62 (m, 1H).

^{13}**C NMR** (101 MHz, $CDCl_3$) δ = 158.9, 89.7, 78.0, 65.2, 49.2, 45.2, 32.2, 29.23, 22.1, 21.0.

HRMS (ESI): *m/z* calc. for $C_{10}H_{15}N_3NaO_3$ $[M+Na]^+$ 248.1006, found 248.1005.

IR (neat) $\nu_{max}(cm^{-1})$: 2955, 2823, 1665, 1552, 1450, 1357, 1299, 745.

Chiral stationary phase SFC: AS3 column, CO_2/MeOH 90:10, 2.0 mL/min, 214 nm; 1.4 min (major), 1.7 min (minor).

5-Oxodecahydrodipyrrolo[1,2-*a*:1',2'-*c*]pyrimidine-6-carbonitrile (**3g**)

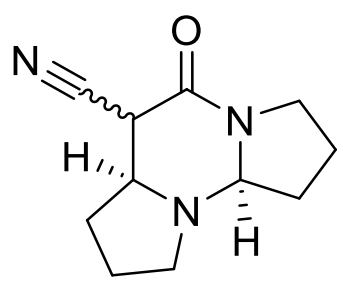

2.3:1 *d.r.*

Compound **3g** was prepared according to **General Procedure 4** starting from methyl cyanoacetate **2g** (17.7 μL, 0.2 mmol, 1.0 equiv.) and pyrroline **1a** (37.7 μL, 0.60 mmol, 3.0 equiv.) with a reaction time of 24 h at –15°C. **C2** (11.6 mg, 0.02 mmol, 10 mol%) was used as catalyst. The product was purified by column chromatography (silica, gradient elution from CH_2Cl_2 to CH_2Cl_2:MeOH 90:10) and isolated as a colorless oil (40 mg, 0.195 mmol, 97% yield, <5% *ee*).

^{1}**H NMR** (400 MHz, $CDCl_3$) δ = 3.86 (dd, *J* = 10.0, 4.9 Hz, 1H), 3.79 – 3.39 (m, 4H), 3.31 (dd, *J* = 10.6, 1.0 Hz, 1H), 3.13 (dtd, *J* = 17.2, 8.9, 3.1 Hz, 1H), 2.89 – 2.72 (m, 1H), 2.44 – 2.24 (m, 2H), 2.19 – 1.56 (m, 11H). (mixture of diastereoisomers)

Major diastereoisomer:

^{13}**C NMR** (101 MHz, $CDCl_3$) δ = 159.6, 116.1, 78.0, 63.9, 49.4, 45.3, 41.0, 32.3, 30.1, 21.8, 20.9.

Minor diastereoisomer:

^{13}**C NMR** (101 MHz, $CDCl_3$) δ = 159.9, 115.8, 78.4, 61.4, 49.9, 45.3, 40.3, 32.2, 28.1, 22.4, 20.8.

HRMS (ESI): *m/z* calc. for $C_{11}H_{15}N_3NaO$ $[M+Na]^+$ 228.1107, found 228.1108.

IR (neat) $\nu_{max}(cm^{-1})$: 2968, 2812, 2247, 1654, 1440, 1382, 1299, 1018, 926.

Chiral stationary phase SFC: AS3 column, CO_2/MeOH 90:10, 2.0 mL/min, 214 nm; major diastereoisomer: 1.1 min (major), 1.8 min (minor); minor diastereoisomer: 1.5 min (minor), 2.9 min (major).

Methyl (6*aS*,10*aS*)-5-methyl-1,2,3,6a,7,8,9,10a-octahydrodipyrrolo[1,2-*a*:1',2'-*c*]pyrimidine-6-carboxylate (3h)

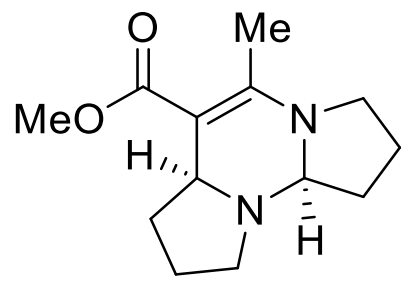

Compound **3h** was prepared according to **General Procedure 4** starting from methyl acetoacetate (21.6 μL, 0.2 mmol, 1.0 equiv.) and pyrroline **1a** (37.7 μL, 0.60 mmol, 3.0 equiv.) with a reaction time of 24 h at –15°C. **C2** (11.6 mg, 0.02 mmol, 10 mol%) was used as catalyst. The product was purified by column chromatography (silica, gradient elution from CH_2Cl_2 to CH_2Cl_2:MeOH 90:10) and isolated as a colorless oil (35.6 mg, 0.15 mmol, 75% yield, 33% *ee*).

^{1}H NMR (500 MHz, $CDCl_3$) δ = 4.32 (dd, *J* = 8.1, 6.3 Hz, 1H), 3.94 (t, *J* = 6.1 Hz, 1H), 3.65 (s, 3H), 3.59 – 3.47 (m, 2H), 2.72 – 2.63 (m, 2H), 2.39 (d, *J* = 1.0 Hz, 3H), 2.31 (dddd, *J* = 13.0, 9.7, 7.0, 5.2 Hz, 1H), 2.19 – 2.10 (m, 1H), 2.05 – 1.97 (m, 1H), 1.90 – 1.77 (m, 4H), 1.70 – 1.61 (m, 1H).

^{13}C NMR (126 MHz, $CDCl_3$) δ = 168.6, 154.0, 96.5, 74.6, 61.0, 49.9, 47.3, 45.1, 31.9, 29.9, 22.7, 22.6, 17.6.

HRMS (ESI): *m/z* calc. for $C_{13}H_{20}N_2NaO_2$ $[M+H]^+$ 237.1598, found 237.1595.

IR (neat) ν_{max}(cm^{-1}): 2945, 2873, 1673, 1553, 1424, 1250, 1155, 1116, 1062, 1012, 775.

Chiral stationary phase SFC: Trefoil column, CO_2/MeOH 90:10, 2.0 mL/min, 254 nm; 2.6 min (major), 4.1 min (minor).

Methyl (6*S*,6*aS*,10*aS*)-6-methyl-5-oxodecahydrodipyrrolo[1,2-*a*:1',2'-*c*]pyrimidine-6-carboxylate (**3i**)

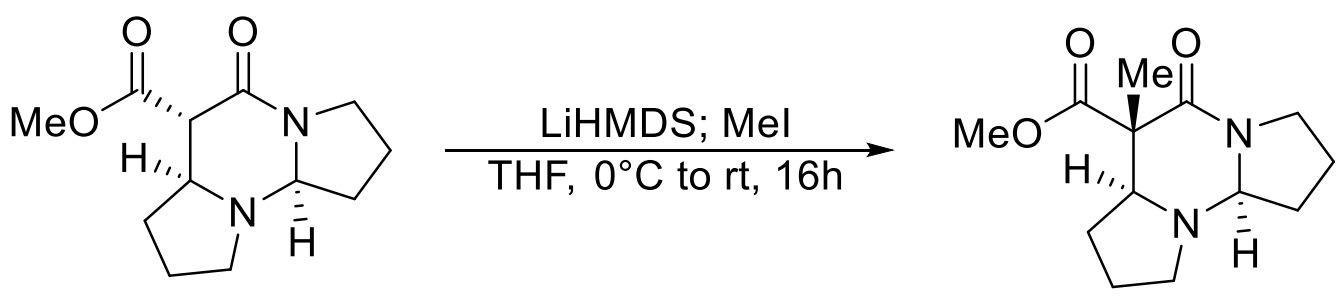

A solution of **3a** (125 mg, 0.525 mmol, 1.0 equiv.) in THF (5.3 mL) was cooled to 0°C. LiHMDS (1.0 M in THF, 0.74 mL, 0.74 mmol, 1.4 equiv.) was added dropwise at this temperature. The reaction mixture was stirred for 5 min and then methyl iodide (45.7 uL, 0.74 mmol, 1.4 equiv.) was added in one portion. The reaction was stirred for 30 min at 0°C and then overnight at room temperature. The reaction was quenched with water (5 mL) and extracted with CH_2Cl_2 (3 x 30 mL). The crude was purified by column chromatography to afford **3h** (61 mg, 0.242 mmol, 46%, 17:1 d.r., 90% ee) as a colorless wax.

^{1}H NMR (400 MHz, $CDCl_3$) δ = 3.84 – 3.72 (m, 4H), 3.72 – 3.61 (m, 1H), 3.54 – 3.45 (m, 1H), 3.21 – 3.09 (m, 1H), 3.03 – 2.91 (m, 1H), 2.26 (q, *J* = 8.8 Hz, 1H), 2.12 (dddd, *J* = 11.5, 6.5, 4.8, 1.3 Hz, 1H), 2.07 – 1.96 (m, 1H), 1.96 – 1.57 (m, 5H), 1.47 (s, 3H).

^{13}C NMR (101 MHz, $CDCl_3$) δ = 172.7, 168.8, 79.2, 67.3, 54.9, 52.5, 50.9, 44.9, 32.8, 24.5, 22.6, 20.8, 16.4.

HRMS (ESI): *m/z* calc. for $C_{13}H_{20}N_2NaO_3$ $[M+Na]^+$ 275.1366, found 275.1360.

IR (neat) ν_{max}(cm^{-1}): 2950, 1738, 1643, 1434, 1256, 1114.

Chiral stationary phase SFC: Trefoil column, CO_2/MeOH 85:15, 2.0 mL/min, 214 nm; 1.4 min (minor), 2.4 min (major).

4.3 General Procedure of sequential reaction with piperideine and then pyrroline

General Procedure 5: Synthesis of Racemates

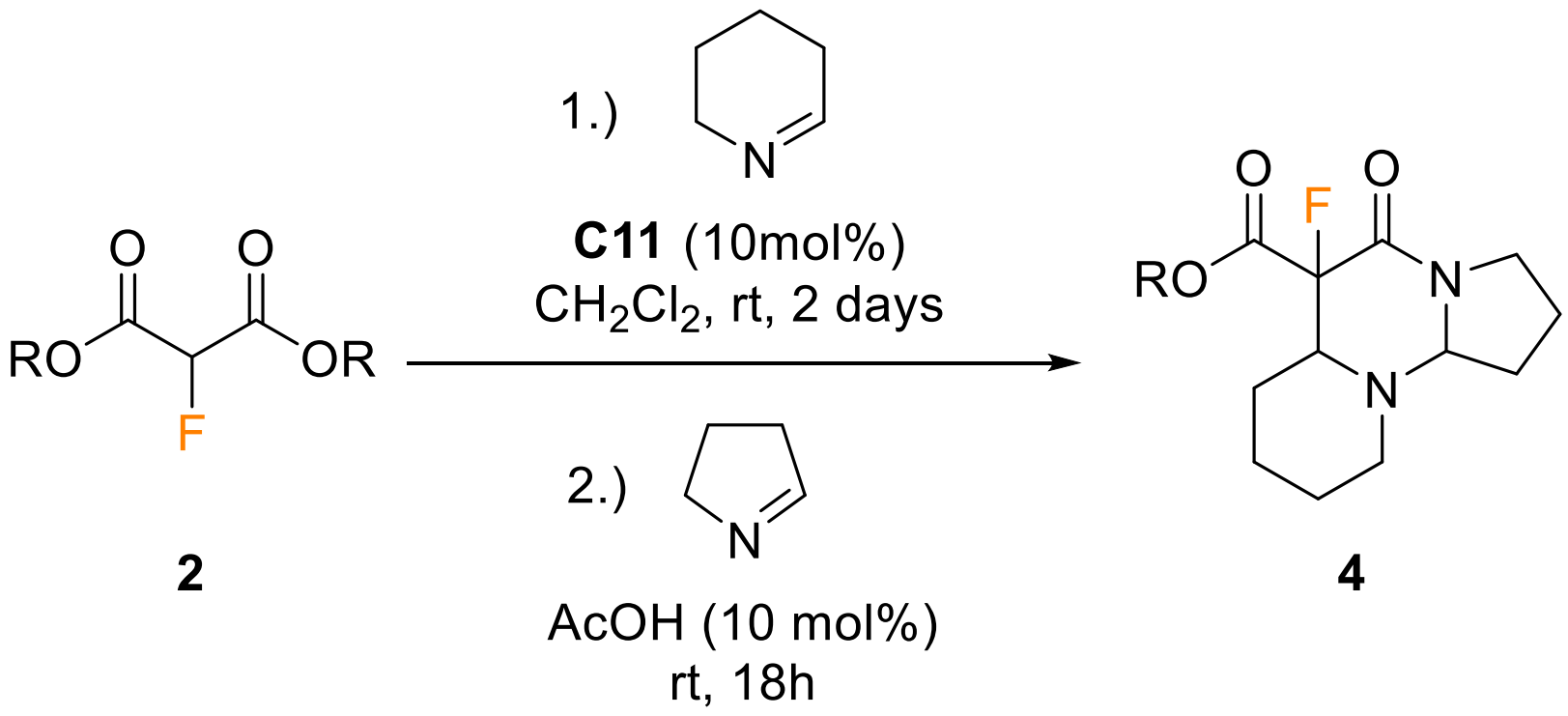

A glass vial was charged with achiral catalyst **C11** (10 mol%, 5.8 mg, 0.02 mmol) and CH_2Cl_2 (2 mL) and the respective fluoromalonate (1 equiv., 0.2 mmol) followed by Δ^3-piperideine (50 mg, 0.6 mmol, 3.0 equiv.) in this order. The reaction mixture was stirred for 2-3 days at room temperature. Then, acetic acid (1.14 µL, 0.2 equiv.) and pyrroline (25.2 µL, 0.4 mmol, 2.0 equiv.) were added and the mixture was stirred for 18 h. The solvent was removed under reduced pressure, and the crude mixture was purified by automated flash column chromatography (gradient elution from CH_2Cl_2 to CH_2Cl_2/MeOH 10:1) to yield the racemic product as a mixture of diastereoisomers after evaporation of the solvents.

General Procedure 6: Substrate Scope

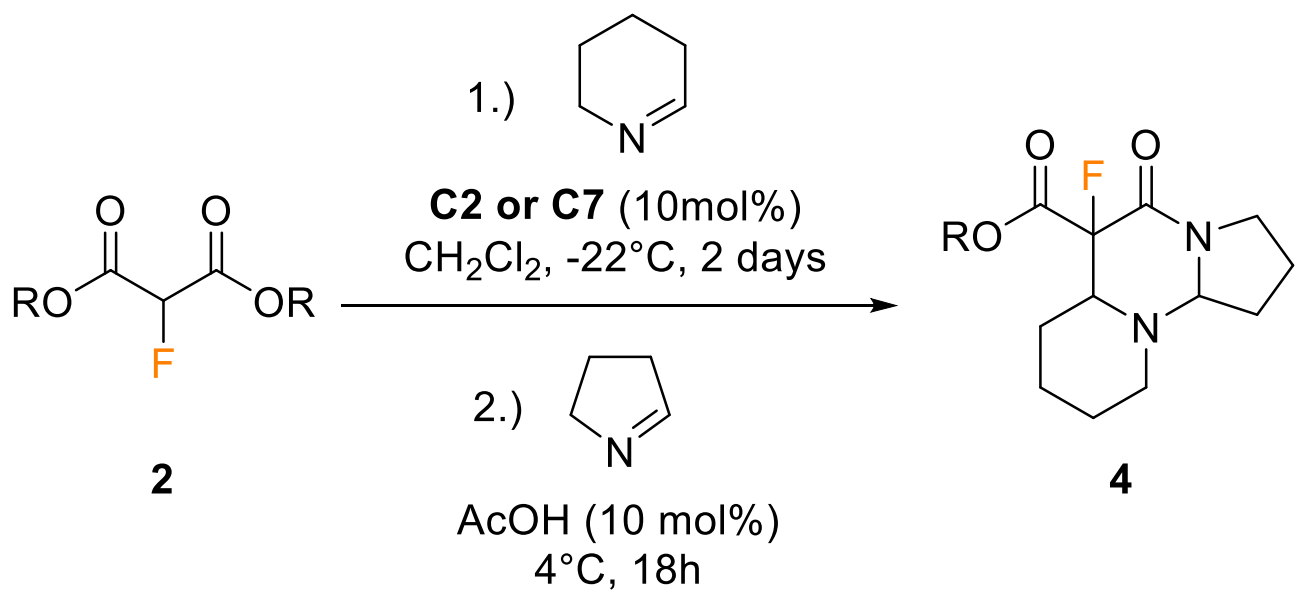

A glass vial (3 to 5 mL) was charged with catalyst (**C2** or **C7**, 12.2 mg, 0.02 mmol, 10 mol%) and CH_2Cl_2 (2 mL, *c* = 0.1 M). Then, the respective fluoromalonate (0.2 mmol, 1 equiv.) was added, followed by Δ-piperideine (49.9 mg, 0.6 mmol, 3.0 equiv.). After 2 days, acetic acid (1.14 µL, 0.02 mmol, 0.01 equiv.) and pyrroline (31.4 µL, 0.5 mmol, 2.5 equiv.) were added and the mixture was stirred for 18 h at 4°C. The solvent was removed under reduced pressure, and the crude mixture was purified by automated flash column chromatography to yield product **4** after evaporation of the solvents.

Analytical Data of 6-6-5 products

Methyl (6*R*,6*aS*,11*aS*)-6-fluoro-5-oxodecahydro-5H-pyrido[1,2-*c*]pyrrolo[1,2-*a*]pyrimidine-6-carboxylate (**4a**)

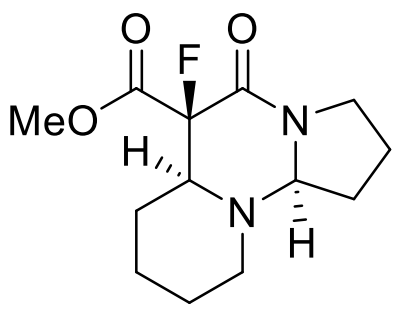

Compound **4a** was prepared according to **General Procedure 6** starting from dimethyl fluoromalonate **2c** (23.1 μL, 0.2 mmol, 1.0 equiv.) using **C2**. The product was purified by column chromatography (silica, gradient elution from CH_2Cl_2 to CH_2Cl_2:MeOH 90:10) and was isolated as a colorless wax (48.5 mg, 0.179 mmol, 90% yield, 91% *ee*).

^{1}H NMR (500 MHz, $CDCl_3$) δ = 3.88 (s, 3H), 3.85 – 3.80 (m, 1H), 3.76 – 3.67 (m, 1H), 3.66 – 3.59 (m, 1H), 3.19 – 3.07 (m, 1H), 2.84 (ddd, *J* = 22.4, 11.1, 3.2 Hz, 1H), 2.33 – 2.23 (m, 1H), 2.16 – 2.05 (m, 2H), 1.91 – 1.71 (m, 5H), 1.67 – 1.55 (m, 2H), 1.38 – 1.23 (m, 1H).

^{19}F NMR {^{1}H} (471 MHz, $CDCl_3$) δ = -165.7.

^{19}F NMR (471 MHz, $CDCl_3$) δ = -165.7 (d, *J* = 22.1 Hz).

^{13}C NMR (126 MHz, $CDCl_3$) δ = 167.8 (d, *J* = 26.6 Hz), 160.3 (d, *J* = 24.0 Hz), 91.5 (d, *J* = 197.8 Hz), 80.0, 65.3 (d, *J* = 20.1 Hz), 53.3 (d, *J* = 1.0 Hz), 51.0, 45.2, 32.1, 24.5, 24.1 (d, *J* = 10.0 Hz), 23.0 (d, *J* = 1.2 Hz), 20.5.

Visible peaks of minor diastereisomer:

^{19}F NMR (471 MHz, $CDCl_3$) δ = -164.3 (d, *J* = 17.8 Hz).

HRMS (ESI): *m/z* calc. for $C_{13}H_{19}FN_2NaO_3$ $[M+Na]^+$ 293.1272, found 293.1276.

IR (neat) ν_{max}(cm^{-1}): 2947, 1766, 1660, 1440, 1269, 1159, 1111, 1040, 778, 725.

Chiral stationary phase SFC: IC3 column, CO_2/MeOH 85:15, 2.0 mL/min, 214 nm; 4.2 min (major), 6.2 min (minor).

Methyl (6*S*,6*aR*,11*aR*)-6-fluoro-5-oxodecahydro-5H-pyrido[1,2-*c*]pyrrolo[1,2-*a*]pyrimidine-6-carboxylate (*ent*-**4a**)

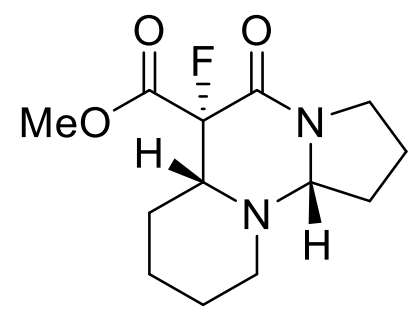

Compound *ent*-**4a** was prepared according to **General Procedure 6** starting from dimethyl fluoromalonate **2c** (23.1 µL, 0.2 mmol, 1.0 equiv.) using **C7**. The product was purified by column chromatography (silica, gradient elution from CH_2Cl_2 to CH_2Cl_2:MeOH 90:10) and isolated as a colorless wax (53.3 mg, 0.197 mmol, 98% yield, 91% *ee*).

The NMR, HRMS and IR data are the same as for **4a**.

Chiral stationary phase SFC: IC3 column, CO_2/MeOH 85:15, 2.0 mL/min, 214 nm; 4.3 min (minor), 6.3 min (major).

Benzyl (6*R*,6*aS*,11*aS*)-6-fluoro-5-oxodecahydro-5H-pyrido[1,2-*c*]pyrrolo[1,2-*a*]pyrimidine-6-carboxylate (**4b**)

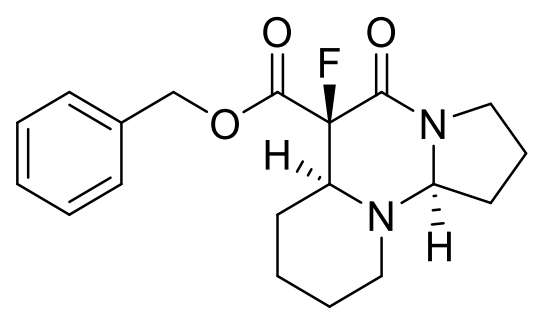

Compound **4b** was prepared according to **General Procedure 6** starting from dibenzyl fluoromalonate **2d** (60.5 mg, 0.2 mmol, 1.0 equiv.) using **C2**. The product was purified by column chromatography (silica, gradient elution from CH_2Cl_2 to CH_2Cl_2:MeOH 90:10) and was isolated as a colorless wax (61.7 mg, 0.178 mmol, 89% yield, 90% *ee*).

^{1}H NMR (500 MHz, $CDCl_3$) δ = 7.45 – 7.31 (m, 5H), 5.38 – 5.27 (m, 2H), 3.85 – 3.76 (m, 1H), 3.75 – 3.58 (m, 2H), 3.14 – 3.06 (m, 1H), 2.81 (ddd, *J* = 22.3, 11.2, 3.2 Hz, 1H), 2.31 – 2.21 (m, 1H), 2.12 – 1.99 (m, 2H), 1.95 – 1.67 (m, 5H), 1.66 – 1.54 (m, 1H), 1.54 – 1.43 (m, 1H), 1.29 – 1.16 (m, 1H).

^{19}F NMR {^{1}H} (471 MHz, $CDCl_3$) δ = -165.6.

^{19}F NMR (471 MHz, $CDCl_3$) δ = -165.6 (d, *J* = 22.3 Hz).

^{13}C NMR (126 MHz, $CDCl_3$) δ = 167.2 (d, *J* = 26.7 Hz), 160.3 (d, *J* = 24.0 Hz), 135.0, 128.6, 128.5, 128.2, 91.4 (d, *J* = 198.0 Hz), 80.0, 67.9 (d, *J* = 0.8 Hz), 65.3 (d, *J* = 20.1 Hz), 51.0, 45.2, 32.1, 24.4, 24.0 (d, *J* = 9.9 Hz), 23.0 (d, *J* = 1.2 Hz), 20.5.

HRMS (ESI): *m/z* calc. for $C_{19}H_{24}FN_2O_3$ $[M+Na]^+$ 347.1765, found 347.1762.

IR (neat) ν_{max}(cm^{-1}): 2946, 1766, 1669, 1456, 1260, 1111, 1040, 906, 730, 698.

Chiral stationary phase SFC: Whelk column, CO_2/MeOH 85:15, 2.0 mL/min, 214 nm; 10.3 min (major), 12.9 min (minor).

4.4 General Procedure for mono-addition reactions followed by Cbz protection

General Procedure 7: Synthesis of Racemates

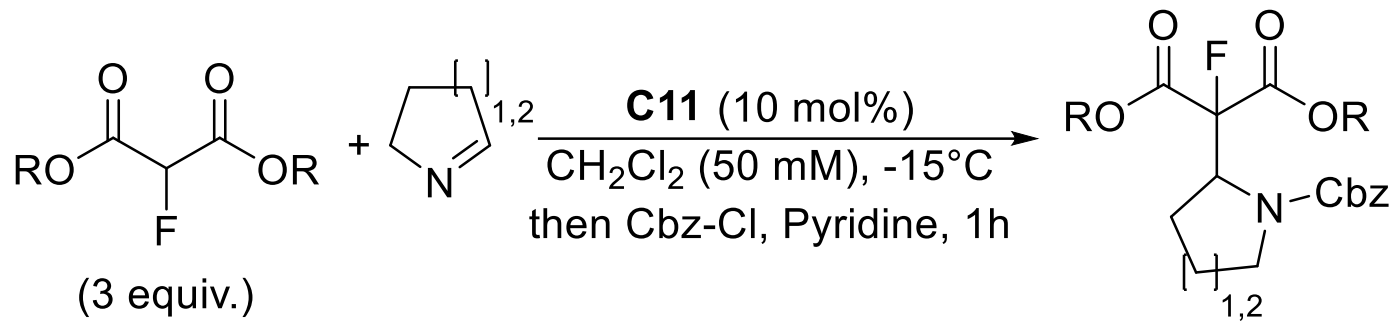

2 **1** **5**

Racemates were prepared on a 0.2 mmol scale. A glass vial was charged with achiral catalyst **C11** (10 mol%, 5.8 mg, 0.02 mmol) and CH_2Cl_2 (2 mL) and then the respective fluoromalonate **2** (3.0 equiv., 0.6 mmol) followed by the imine **1** (1.0 equiv.) were added. The reaction mixture was stirred for 16-120 h at room temperature. Then, benzyl chloroformate (1.5 equiv.) and pyridine (1.5 equiv.) were added at 0 °C. The reaction was stirred for 2 h at room temperature. The solvent was removed under reduced pressure, and the crude mixture was subjected to automated flash column chromatography (gradient elution from hexanes to hexanes/EtOAc 1:1) to yield the racemic product after evaporation of the solvents.

General Procedure 8: Mono addition followed by Cbz protection

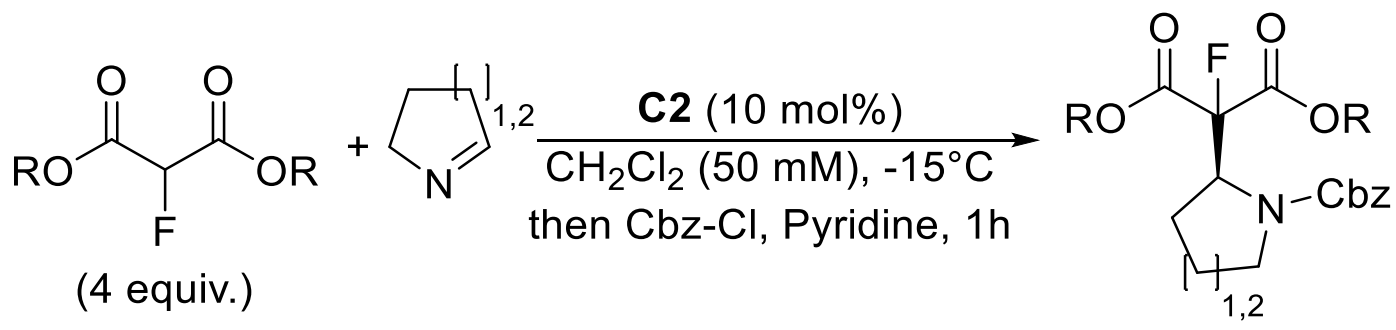

2 **1** **5**

A glass vial was charged with the chiral catalyst **C2** (12.2 mg, 0.02 mmol, 10 mol%), dissolved in CH_2Cl_2 (2 mL) and the respective malonate (4.0 equiv., 0.8 mmol) followed by the imine (0.2 mmol, 1.0 equiv.) were added. The reaction mixture was stirred for 2 days at -15 or -22°C. Then, benzyl chloroformate (42.7 μL, 0.3 mmol, 1.5 equiv.) and pyridine (24.2 μL, 0.3 mmol, 1.5 equiv.) were added at 0°C. The reaction was stirred for 1h at room temperature. The solvent was removed under reduced pressure and the crude mixture was subjected to automated flash column chromatography (elution gradient from hexanes to hexanes/EtOAc 1:1) to yield the product **5** after evaporation of the solvents.

4.5 Analytical Data of mono-addition reactions

Dimethyl (*S*)-2-(1-((benzyloxy)carbonyl)pyrrolidin-2-yl)-2-fluoromalonate (5a):

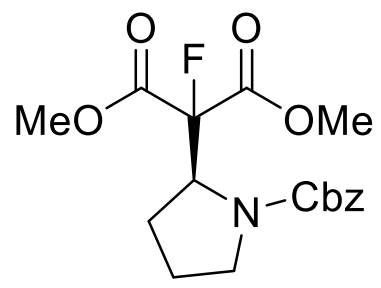

Compound **5a** was prepared according to **General Procedure 8** starting from dimethyl fluoromalonate **2c** (92.4 μL, 0.8 mmol, 1.0 equiv.) and pyrroline **1a** (12.6 μL, 0.2 mmol, 1.0 equiv.). The product was purified by column chromatography (silica, gradient elution from hexanes to hexanes:EtOAc 1:1) and isolated as a colorless oil (48.4 mg, 0.134 mmol, 69% yield, 88% *ee*).

^{1}H NMR (400 MHz, $CDCl_3$) δ = 7.42 – 7.29 (m, 5H), 5.27 – 4.99 (m, 2H), 4.87 (ddd, *J* = 28.2, 8.5, 2.7 Hz, 1H), 3.87 (s, 3H), 3.86 – 3.66 (m, 3H), 3.65 – 3.43 (m, 2H), 2.29 – 2.12 (m, 1H), 2.13 – 1.96 (m, 1H), 1.95 – 1.78 (m, 2H).

^{19}F NMR {^{1}H} (377 MHz, $CDCl_3$) δ = -179.4.

^{19}F NMR (377 MHz, $CDCl_3$) δ = -179.4 (d, *J* = 28.4 Hz).

^{13}C NMR (101 MHz, $CDCl_3$) δ = 165.8 (d, *J* = 24.2 Hz), 164.9 (d, *J* = 27.2 Hz), 155.3, 136.5, 128.5, 128.0, 127.8, 95.9 (d, *J* = 206.2 Hz), 67.2, 60.5, 53.4, 47.4, 28.1, 24.0.

HRMS (ESI): *m/z* calc. for $C_{17}H_{21}FNO_6$ $[M+Na]^+$ 354.1347, found 354.1346.

IR (neat) $\nu_{max}(cm^{-1})$: 2956, 1750, 1700, 1406, 1249, 1113, 732, 698.

Chiral stationary phase SFC: IC3 column, CO_2/MeOH 93:7, 2.0 mL/min, 214 nm; 1.9 min (minor), 2.2 min (major).

Dibenzyl (*S*)-2-(1-((benzyloxy)carbonyl)pyrrolidin-2-yl)-2-fluoromalonate (5b):

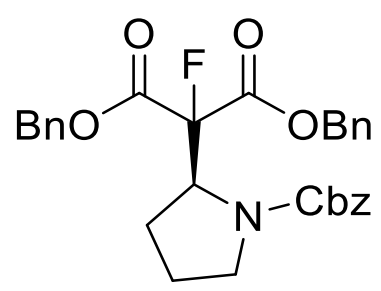

Compound **5b** was prepared according to **General Procedure 8** starting from dibenzyl fluoromalonate (74.3 mg, 0.8 mmol, 1.0 equiv.) and pyrroline (12.6 μL, 0.2 mmol, 1.0 equiv.). The product was purified by column chromatography (silica, gradient elution from hexanes to hexanes:EtOAc 1:1) and isolated as a colorless wax (80.3 mg, 0.159 mmol, 79% yield, 92% *ee*).

^{1}H NMR (500 MHz, $CDCl_3$) δ 7.36 – 7.25 (m, 15H), 5.27 – 5.01 (m, 6H), 4.91 (ddd, *J* = 27.9, 8.4, 2.8 Hz, 1H), 3.53 (d, *J* = 38.2 Hz, 2H), 2.18 – 1.95 (m, 2H), 1.89 – 1.75 (m, 2H).

^{19}F NMR {^{1}H} (471 MHz, $CDCl_3$) δ = -178.9.

^{19}F NMR (471 MHz, $CDCl_3$) δ = -178.9 (d, *J* = 28.1 Hz).

^{13}C NMR (126 MHz, $CDCl_3$) δ = 165.1 (d, *J* = 24.8 Hz), 164.3 (d, *J* = 27.9 Hz), 155.4, 136.6, 135.0, 134.4, 128.6, 128.6, 128.5, 128.5, 128.4, 128.3, 128.0, 127.8, 127.0, 95.9 (d, *J* = 206.9 Hz), 68.4, 68.2, 67.2, 65.4, 60.6 (d, *J* = 20.2 Hz), 47.4, 28.0, 24.0.

HRMS (ESI): *m/z* calc. for $C_{29}H_{28}FNNaO_6$ $[M+Na]^+$ 528.1793, found 528.1783.

IR (neat) $v_{max}(cm^{-1})$: 2956, 1749, 1700, 1407, 1278, 1194, 1113, 739, 697.

Chiral stationary phase SFC: IC3 column, CO_2/MeOH 85:15, 2.0 mL/min, 214 nm; 2.0 min (major), 2.3 min (minor).

Dimethyl (*S*)-2-(1-((benzyloxy)carbonyl)piperidin-2-yl)-2-fluoromalonate (5c):

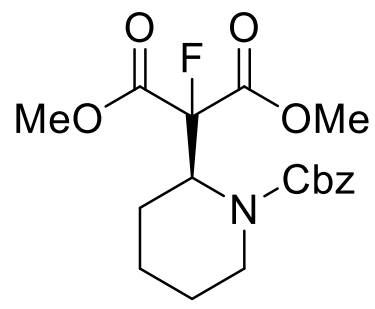

Compound **5c** was prepared according to **General Procedure 8** on 0.3 mmol scale. Note: here, no excess of imine was needed since the reaction stops after the first addition. Starting from dimethyl fluoromalonate (34.6 μL, 0.3 mmol, 1.0 equiv.) and piperideine (49.9 μL, 0.6 mmol, 1.0 equiv.), the product was purified by column chromatography (silica, gradient elution from hexanes to hexanes:EtOAc 1:1) and isolated as a colourless oil (58.9 mg, 0.160 mmol, 53% yield, 94% *ee*).

^{1}H NMR (500 MHz, $CDCl_3$) (mixture of two isomers due to hindered rotation) δ = 7.47 – 7.26 (m, 5H), 5.29 – 5.02 (m, 3H), 4.24 – 4.02 (m, 1H), 3.85 (s, 3H), 3.54 (d, *J* = 30.4 Hz, 3H), 3.27 – 3.01 (m, 1H), 1.79 (d, *J* = 13.6 Hz, 3H), 1.62 (t, *J* = 12.5 Hz, 2H), 1.50 – 1.32 (m, 1H).

^{19}F NMR {^{1}H} (471 MHz, $CDCl_3$) δ = -169.5 – -169.7 (m).

^{19}F NMR (471 MHz, $CDCl_3$) δ = -169.6 (d, *J* = 33.9 Hz).

^{13}C NMR (126 MHz, $CDCl_3$) δ = 165.4 (d, *J* = 25.2 Hz), 165.0 (d, *J* = 28.1 Hz), 155.6, 155.2, 136.7, 136.5, 128.5, 128.0, 127.8, 100.2 (d, *J* = 209.3 Hz), 99.0 (d, *J* = 206.1 Hz), 67.4, 53.5, 53.1, 52.7 (d, *J* = 18.2 Hz), 41.5, 25.2, 24.5, 20.3.

HRMS (ESI): *m/z* calc. for $C_{18}H_{22}FNNaO_6$ $[M+Na]^+$ 390.1323, found 390.1326.

IR (neat) $v_{max}(cm^{-1})$: 2954, 1751, 1696, 1416, 1241, 1154, 1065, 1027, 945, 699.

Chiral stationary phase HPLC: IA column, hexane/*i*-PrOH 80:20, 0.5 mL/min, 25 °C, 210 nm; 19.5 min (major), 21.9 min (minor).

4.6 Synthesis of unprotected mono-addition product

Dimethyl (*S*)-2-fluoro-2-(piperidin-2-yl)malonate (6):

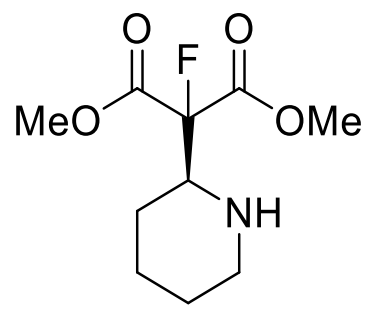

Compound **6** was prepared according to **General Procedure 8** starting from dimethyl fluoromalonate (69.3 μL, 0.6 mmol, 1.0 equiv.) and piperideine (124.7 mg, 1.5 mmol, 2.5 equiv.). Catalyst **C2** (34.7 mg, 0.06 mmol, 10 mol%) was used and the reaction was conducted at -22°C for 2 days. The product was directly purified by column chromatography (silica, gradient elution from CH_2Cl_2 to CH_2Cl_2:MeOH 90:10) and isolated as a colorless oil (127.7 mg, 0.548 mmol, 91% yield, 83% *ee*).

^{1}H NMR (400 MHz, $CDCl_3$) δ = 3.87 (s, 3H), 3.86 (s, 3H), 3.46 (ddd, *J* = 24.4, 11.4, 2.6 Hz, 1H), 3.17 – 3.07 (m, 1H), 2.73 – 2.57 (m, 1H), 1.99 – 1.88 (m, 1H), 1.66 – 1.55 (m, 2H), 1.55 – 1.46 (m, 1H), 1.44 – 1.36 (m, 1H), 1.35 – 1.16 (m, 2H).

^{19}F NMR {^{1}H} (471 MHz, $CDCl_3$) δ = -180.0.

^{19}F NMR (471 MHz, $CDCl_3$) δ = -180.0 (d, *J* = 24.4 Hz).

^{13}C NMR (101 MHz, $CDCl_3$) δ = 166.0 (d, *J* = 26.5 Hz), 165.6 (d, *J* = 25.2 Hz), 97.4 (d, *J* = 203.5 Hz), 60.2 (d, *J* = 20.2 Hz), 53.5, 47.2, 26.9, 26.2 (d, *J* = 2.9 Hz), 24.4.

HRMS (ESI): *m/z* calc. for $C_{10}H_{17}FNO_4$ $[M+Na]^+$ 234.1136, found 234.1142.

IR (neat) ν_{max}(cm^{-1}): 3308, 2934, 1748, 1656, 1436, 1245, 1172, 1031, 780.

Chiral stationary phase SFC: Trefoil column, CO_2/MeOH 95:5, 2.0 mL/min, 214 nm; 0.9 min (major), 1.6 min (minor).

5 Total synthesis of Tetraponerine T-1 and *ent*-T1

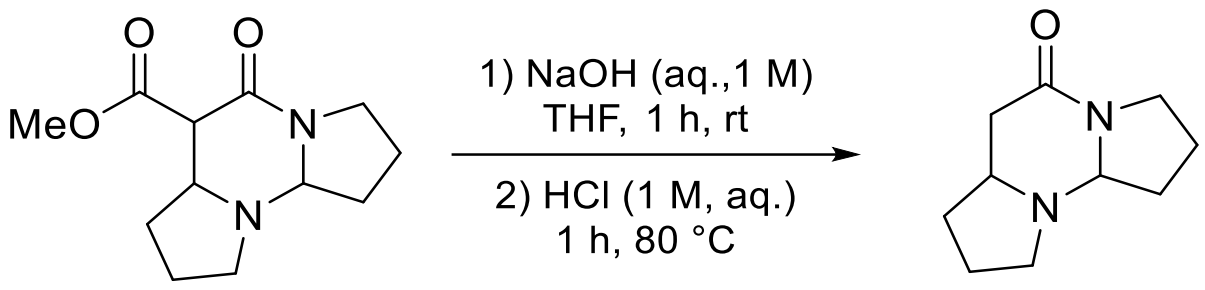

General procedure 9: The ester (117 mg, 491 mmol, 1.0 equiv.) was dissolved in THF (490 µL, 1M). A solution of 1 M NaOH (aq., 980 µL, 980 µmol, 2.0 equiv.) was added dropwise over 1 min and the mixture was stirred at rt for 1.5 h. Then, aqueous 1 M HCl (2.5 mL, 2.5 mmol, 5.0 equiv.) was added and the mixture was heated to 80 °C for 2 h. The mixture was diluted with CH_2Cl_2 and the aqueous phase was washed with CH_2Cl_2 (10 mL). The phases were separated and the aqueous layer containing the product was basified with NaOH (1M, 1.6 mL). The aqueous layer was extracted with CH_2Cl_2 (3 x 30 mL). The combined organic layers were dried over Na_2SO_4 and the solvent was removed under reduced pressure to yield the pure lactam **7** (62.2 mg, 0.35 mmol, 70%) as a colorless oil.

(6*aR*,10*aR*)-Octahydrodipyrrolo[1,2-*a*:1',2'-*c*]pyrimidin-5(6H)-one (**7**)**:**

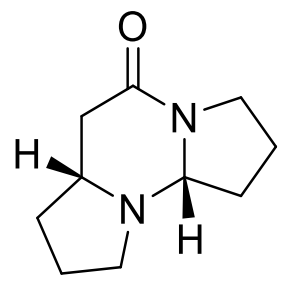

Prepared according to **General procedure 10**. The data matches reported data.[11]

^{1}H NMR (500 MHz, $CDCl_3$) δ = 3.73 (dd, *J* = 9.8, 5.0 Hz, 1H), 3.68 – 3.58 (m, 1H), 3.53 – 3.43 (m, 1H), 3.11 (td, *J* = 8.5, 2.8 Hz, 1H), 2.62 (dd, *J* = 17.0, 4.8 Hz, 1H), 2.56 – 2.47 (m, 1H), 2.33 – 2.19 (m, 2H), 2.17 – 2.09 (m, 1H), 2.11 – 1.90 (m, 3H), 1.92 – 1.77 (m, 2H), 1.75 – 1.63 (m, 1H), 1.55 – 1.43 (m, 1H).

^{13}C NMR (126 MHz, $CDCl_3$) δ = 167.8, 78.4, 61.2, 49.7, 44.6, 38.0, 32.5, 30.8, 22.1, 21.0.

HRMS (ESI): *m/z* calc. for $C_{10}H_{17}N_2O$ [M+Na]$^+$ 181.1335, found 181.1339.

IR (neat) ν_{max}(cm^{-1}): 2956, 2803, 1629, 1457, 1299, 1024.

R*f* = 0.4 (10% MeOH in CH_2Cl_2).

Chiral stationary phase HPLC: OD column, hexane/*i*-PrOH 85:15, 0.5 mL/min, 25 °C, 210 nm; 15.2 min (major), 17.3 min (minor).

(6*aS*,10*aS*)-octahydrodipyrrolo[1,2-*a*:1',2'-*c*]pyrimidin-5(6H)-one (*ent*-7):

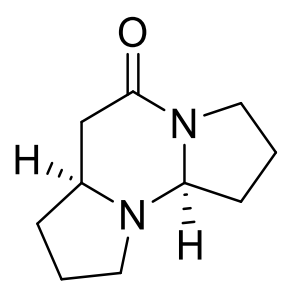

Prepared according to **General procedure 9**. The NMR, HRMS and IR data are identical to **7**.

Chiral stationary phase HPLC: OD column, hexane/*i*-PrOH 85:15, 0.5 mL/min, 25 °C, 210 nm; 15.3 min (minor), 17.1 min (major).

Synthesis of Tetraponerine T1:

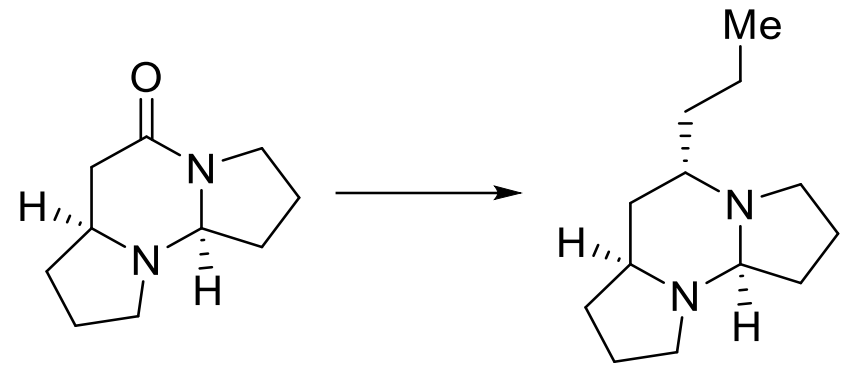

General procedure 10: A dried and nitrogen filled *Schlenk*-tube was charged with $[IrCl(CO)[P(C_6H_5)_3]_2]$ (*Vaska's* complex, 2 mg, 2.5 μmol, 1 mol%), evacuated and backfilled with nitrogen. Then, a solution of lactam **7** (45.6 mg, 0.25 mmol, 1.0 equiv.) in CH_2Cl_2 (2.5 mL) was added followed by tetramethyldisiloxane (89 μL, 0.56 mmol, 2.0 equiv.). The mixture was stirred at rt for 20 min and then cooled to –78 °C. Then, *n*-PrMgBr (1.8 M in THF, 0.6 mL, 1.0 mmol, 4.0 equiv.) was added dropwise to the solution and the mixture was stirred for 60 min at –78 °C and then allowed to warm to rt where it was stirred for 4 h. The mixture was quenched with saturated aqueous NH_4Cl (5 mL). The mixture was transferred to a separatory funnel and the aqueous layer was acidified with 1 M HCl (2 x 5 mL) to pH 1. The phases were separated and the aqueous layer was basified with NaOH to pH 14. The product was extracted with CH_2Cl_2 and the combined organic layers were dried over Na_2SO_4 and the solvent was removed under reduced pressure. The crude was purified by flash column chromatography (0→30% MeOH/CH_2Cl_2) to yield the product (6a*R*,10a*S*)-5-Propyldecahydrodipyrrolo[1,2-*a*:1',2'-*c*]pyrimidine **T-1** (27.3 mg, 253 μmol, 52%, 15:1 d.r.) as a colorless oil.

^{1}H-NMR (500 MHz, $CDCl_3$) δ = 3.66 – 3.47 (m, 1H), 3.25 – 3.10 (m, 1H), 3.09 – 2.96 (m, 2H), 2.93 – 2.85 (m, 1H), 2.31 – 2.09 (m, 1H), 2.08 – 1.73 (m, 8H), 1.68 – 1.54 (m, 3H), 1.51 – 1.40 (m, 2H), 1.38 – 1.29 (m, 2H), 0.90 (t, J = 7.3 Hz, 3H). In agreement with prior reports,[33] the molecule adopts two conformations in $CDCl_3$.

^{13}C-NMR (126 MHz, $CDCl_3$) δ = 76.9, 58.3, 53.6, 50.3, 49.5, 33.5, 30.6, 29.3, 29.0, 21.1, 20.4, 20.0, 14.1. The molecule is present as a mixture of two conformers in $CDCl_3$ which is in agreement with literature [33].

HRMS (ESI): *m/z* calc. for $C_{13}H_{25}N_2$ $[M+Na]^+$ 209.2012, found 209.2013.

R_f = 0.3 (20% MeOH in CH_2Cl_2).

$[\alpha]_D$: +15.4 (c = 0.498, $CHCl_3$, 25 °C). In agreement with prior reports.[33]

Synthesis of Tetraponerine *ent*-T1:

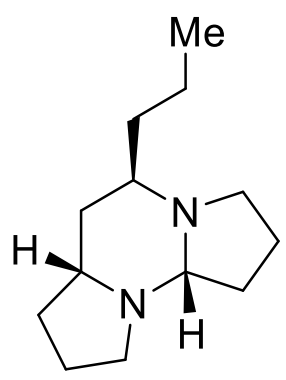

Prepared according to **General procedure 10**. The NMR, HRMS and IR data are identical to **T1**.

$[\alpha]_D$: -14.5 (c = 0.498, $CHCl_3$, 25 °C).

6 X-Ray Crystallographic Data

Crystal Structure of 3a:

Single crystals of **3a** were obtained from the neat compound. A suitable crystal was selected and measured on a XtaLAB Synergy, Dualflex, Pilatus 300K diffractometer. The crystal was kept at 100.0(1) K during data collection. Using Olex2,[48] the structure was solved with the SHELXT[49] structure solution program using Intrinsic Phasing and refined with the SHELXL[50] refinement package using Least Squares minimization.

Crystal Data for $C_{12}H_{18}N_2O_3$ (M = 238.28 g/mol): crystal size: 0.185 x 0.151 x 0.09 mm, orthorhombic, space group $P2_12_12_1$ (no. 19), a = 8.2351(1) Å, b = 10.4499(1) Å, c = 14.0030(2) Å, V = 1205.04(3) Å^3, Z = 4, T = 100.0(1) K, μ(Cu Kα) = 0.780 mm^{-1}, $Dcalc$ = 1.313 g/cm^3, 21902 reflections collected (10.564° ≤ 2Θ ≤ 149.82°), 2393 unique (R_{int} = 0.0244, R_{sigma} = 0.0143) which were used in all calculations. The final R_1 was 0.0315 (I > 2σ(I)) and wR_2 was 0.0793 (all data).

The crystal structure is deposited in the Cambridge Crystallographic Data Centre (CCDC Code: 2542707).

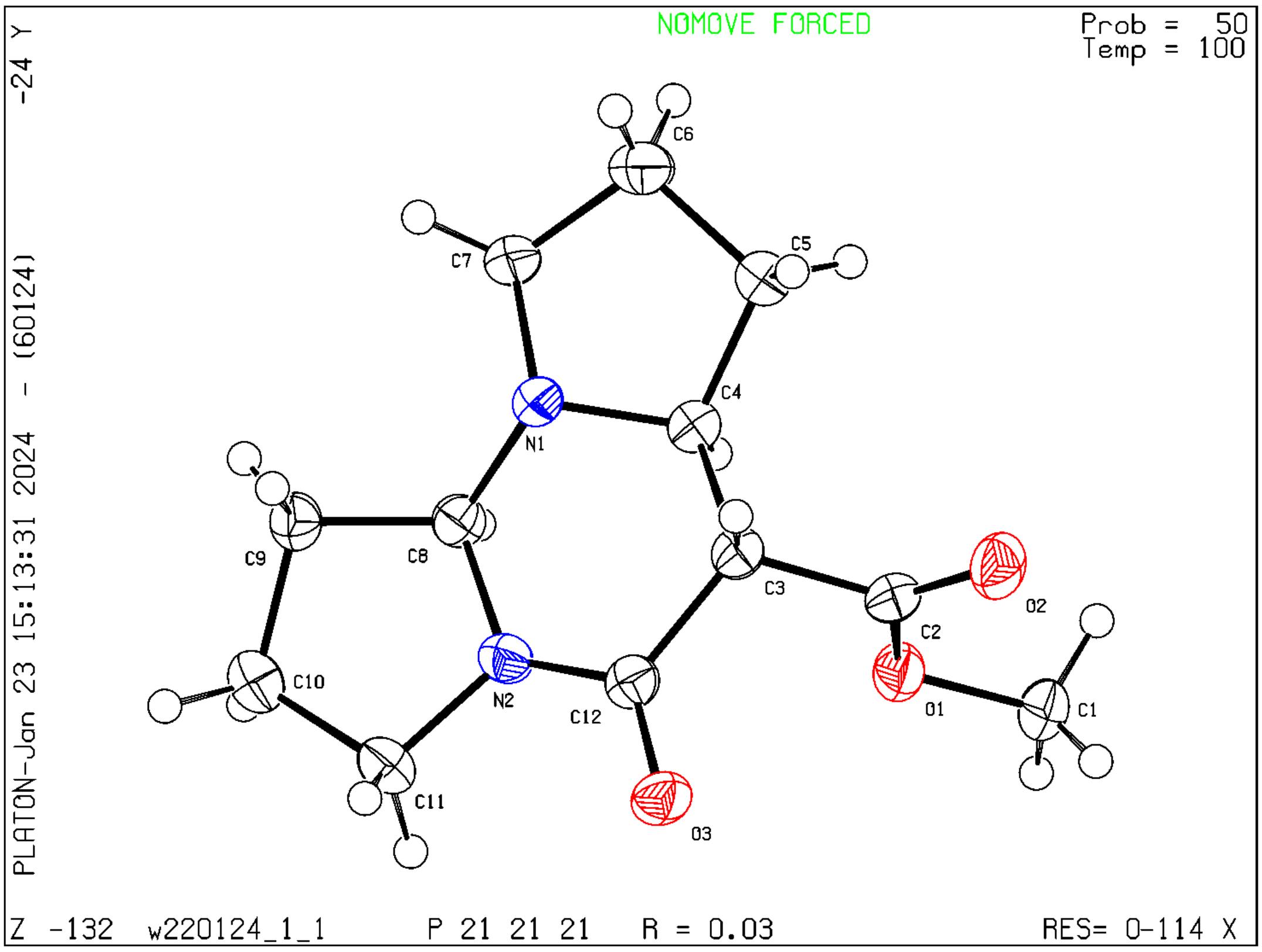

Figure S1: **Crystal structure of** 3a**.**

Crystal Structure of 3c:

Single crystals of **3c** were obtained by slow evaporation of $CHCl_3$. A suitable crystal was selected and measured on a XtaLAB Synergy, Dualflex, Pilatus 300K diffractometer. The crystal was kept at 100.0(1) K during data collection. Using Olex2,[48] the structure was solved with the SHELXT[49] structure solution program using Intrinsic Phasing and refined with the SHELXL[50] refinement package using Least Squares minimization.

Crystal Data for $C_{12}H_{17}N_2O_3F$ (M = 256.27 g/mol): crystal size: 0.138 x 0.107 x 0.033 mm, orthorhombic, space group $P2_12_12_1$ (no. 19), a = 9.35130(10) Å, b = 9.80580(10) Å, c = 13.6026(2) Å, V = 1247.32(3) Å^3, Z = 4, T = 100.0(1) K, μ(Cu Kα) = 0.913 mm^{-1}, $Dcalc$ = 1.365 g/cm^3, 33218 reflections collected (11.124° ≤ 2Θ ≤ 158.9°), 2591 unique (R_{int} = 0.0447, R_{sigma} = 0.0182) which were used in all calculations. The final R_1 was 0.0274 (I > 2σ(I)) and wR_2 was 0.0714 (all data).

The crystal structure is deposited in the Cambridge Crystallographic Data Centre (CCDC Code: 2542708).

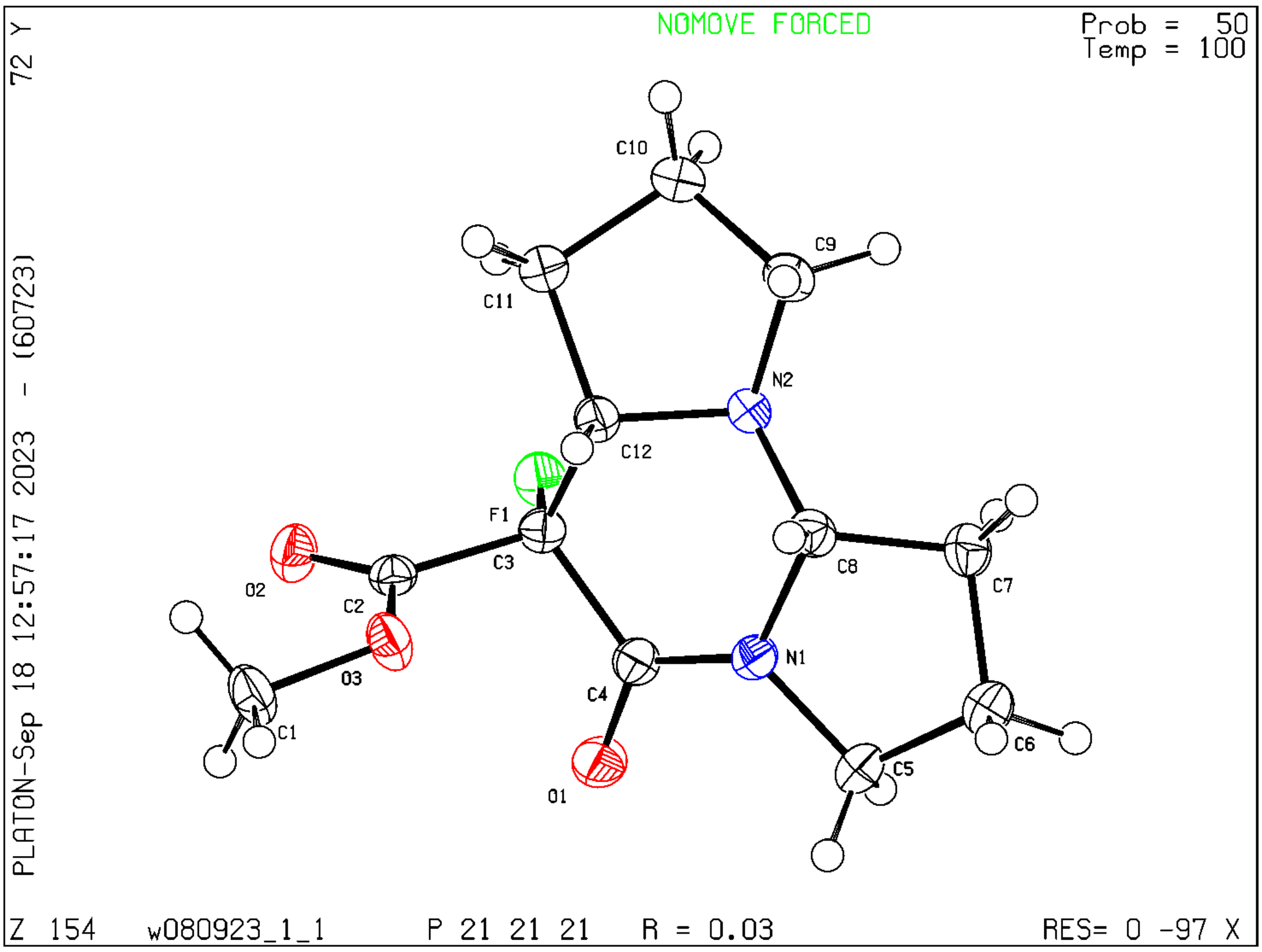

Figure S2: **Crystal structure of** 3c**.**

Crystal Structure of ***ent*****-4a:**

Single crystals of *ent*-**4a** were obtained by slow evaporation of THF. A suitable crystal was selected and measured on a XtaLAB Synergy, Dualflex, Pilatus 300K diffractometer. The crystal was kept at 100.0(1) K during data collection. Using Olex2,[48] the structure was solved with the SHELXT[49] structure solution program using Intrinsic Phasing and refined with the SHELXL[50] refinement package using Least Squares minimization.

Crystal Data for $C_{13}H_{19}N_2O_3F$ (M = 270.30 g/mol): crystal size: 0.166 x 0.134 x 0.083 mm, orthorhombic, space group $P2_12_12_1$ (no. 19), a = 8.09139(5) Å, b = 9.89738 Å, c = 15.86201(10) Å, V = 1270.286(14) Å^3, Z = 4, T = 100.0(1) K, μ(Cu Kα) = 0.925 mm^{-1}, $Dcalc$ = 1.413 g/cm^3, 45150 reflections collected (10.536° ≤ 2Θ ≤ 149.776°), 2504 unique (R_{int} = 0.0252, R_{sigma} = 0.0100) which were used in all calculations. The final R_1 was 0.0243 (I > 2σ(I)) and wR_2 was 0.0619 (all data).

The crystal structure is deposited in the Cambridge Crystallographic Data Centre (CCDC Code: 2542709).

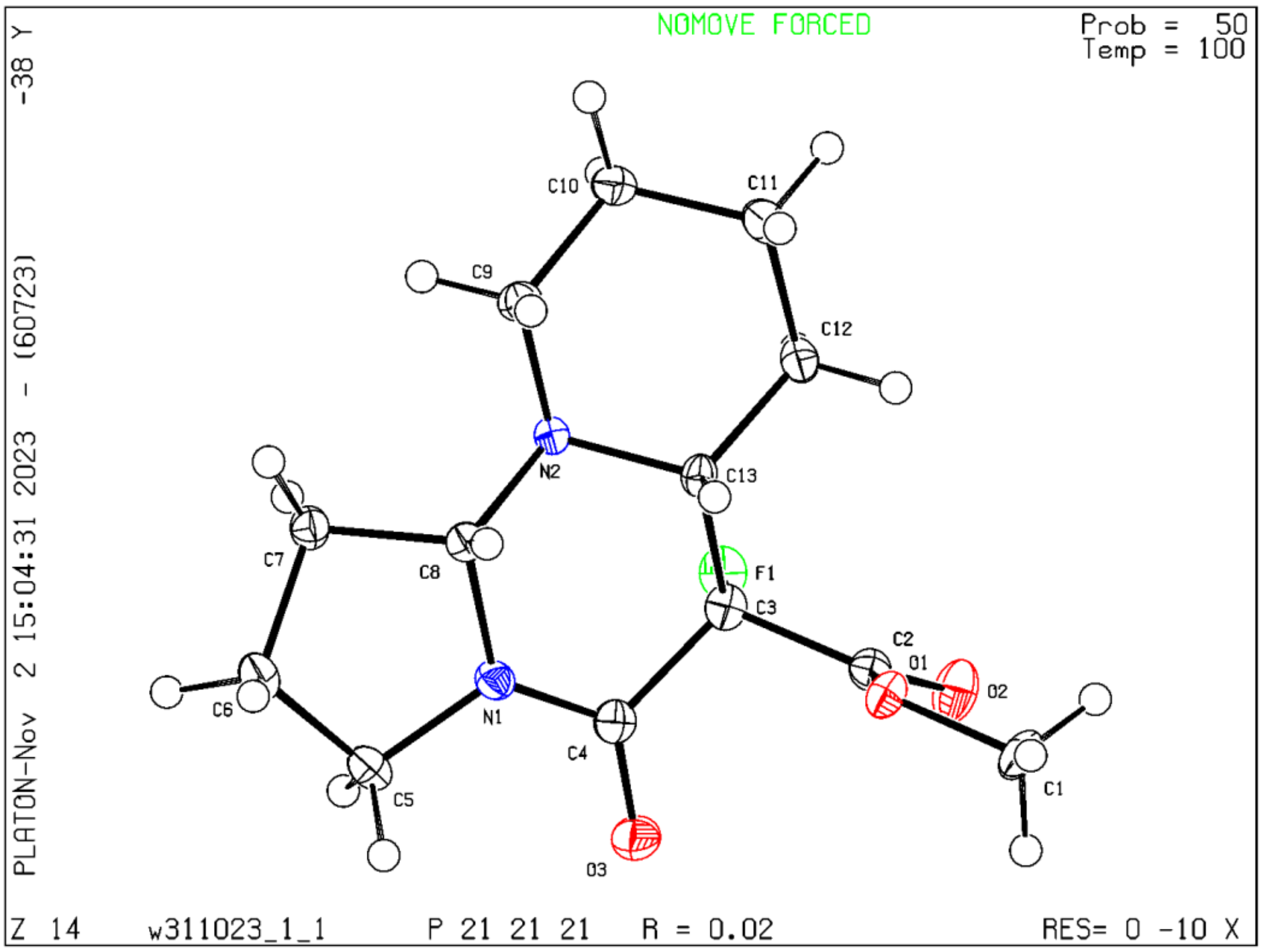

Figure S3: **Crystal structure of** ***ent*****-4a.**

7 References

[6] S. Dutta, J. H. Kim, K. Bhatt, D. R. L. Rickertsen, K. A. Abboud, I. Ghiviriga, D. Seidel, *Angew. Chem. Int. Ed.* **2024**, *63*, e202313247.

[7] M. R. Monaco, P. Renzi, D. M. Scarpino Schietroma, M. Bella, *Org. Lett.* **2011**, *13*, 4546.

[26] I. Bosque, J. C. Gonzalez-gomez, J. Brea, *J. Org. Chem.* **2014**, *79*, 3982.

[33] M. Plehiers, S. Heilporn, D. Ekelmans, S. Leclercq, M. Sangermano, J. C. Braekman, D. Daloze, *Can. J. Chem.* **2000**, *78*, 1030.

[34] S. H. McCooey, S. J. Connon, *Angew. Chem.* 2005, 117, 6525.

[35] J. Ye, D. J Dixon, P. Hynes, *Chem. Commun.* **2005**, 4481.

[36] B. Vakulya, S. Varga, A. Csámpai, T. Soós, *Org. Lett.* 2005, 7, 1967.

[37] T. Okino, Y. Hoashi, T. Furukawa, X. Xu, Y. Takemoto *J. Am. Chem. Soc.* **2005**, *127*, 119.

[38] J. P. Malerich, K. Hagihara, V. H. Rawal, *J. Am. Chem. Soc.* **2008**, *130*, 14416.

[45] U. Gellrich, J. R. Khusnutdinova, G. M. Leitus, D. Milstein, *J. Am. Chem. Soc.* **2015**, *137*, 4851.

[46] T. J. Barker, E. R. Jarvo, *J. Am. Chem. Soc.* **2009**, *131*, 15598.

[47] T. Wei, M. E. Pérez-Ojeda, A. Hirsch, *Chem. Commun.* **2017**, 53, 7886.

[48] O. V. Dolomanov, L. J. Bourhis, R. J. Gildea, J. A. K. Howard, H. Puschmann, *J. Appl. Cryst.* **2009**, *42*, 339.

[49] G. M. Sheldrick, *Acta Cryst*. **2015**, *A71*, 3.

[50] G. M. Sheldrick, *Acta Cryst*. **2015**, *C71*, 3.

8 NMR Spectra of starting materials

8.1 Cyclic imines

^{1}H NMR (400 MHz, $CDCl_3$) of pyrroline **1a**:

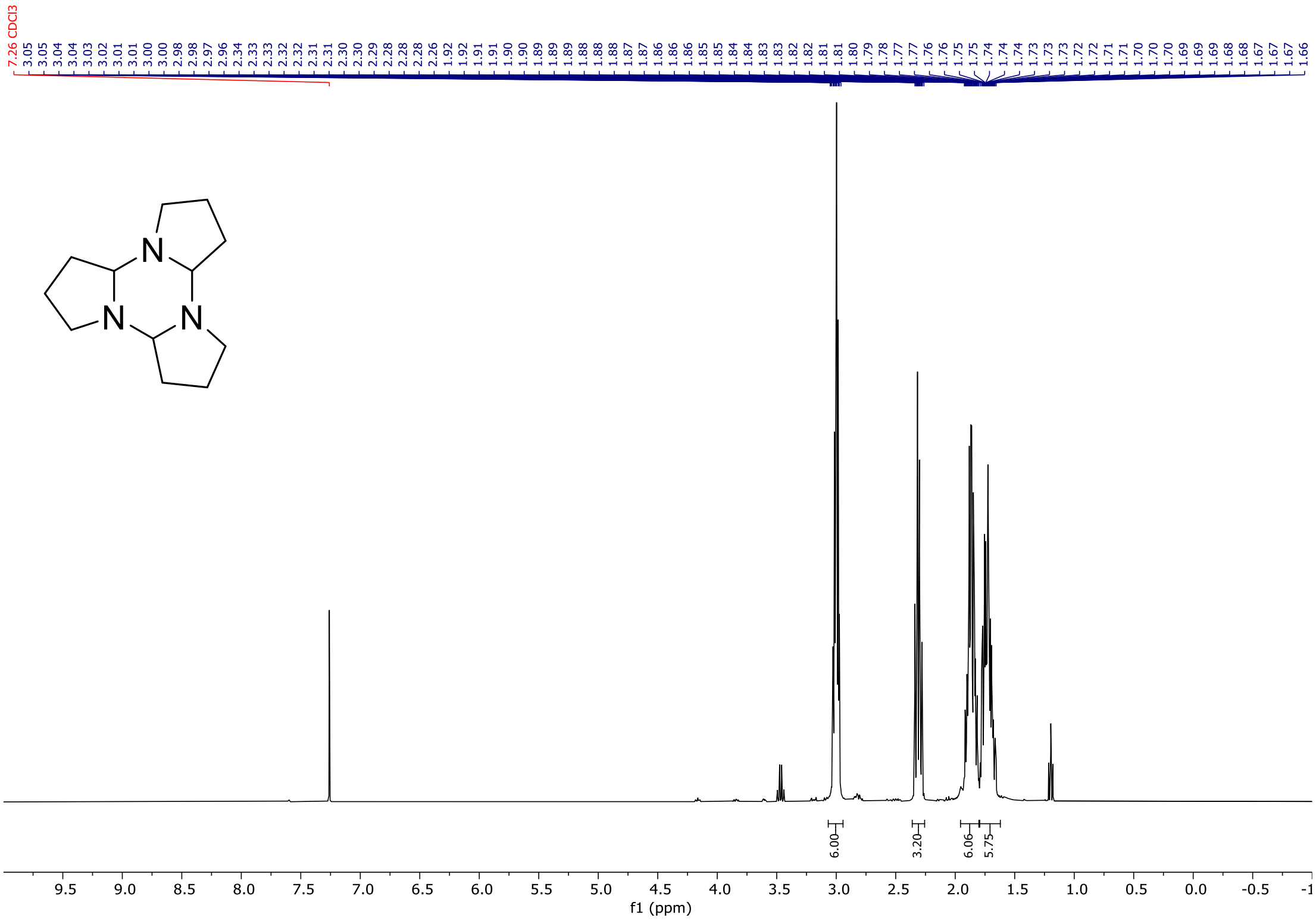

^{13}C NMR (101 MHz, $CDCl_3$) of pyrroline **1a**:

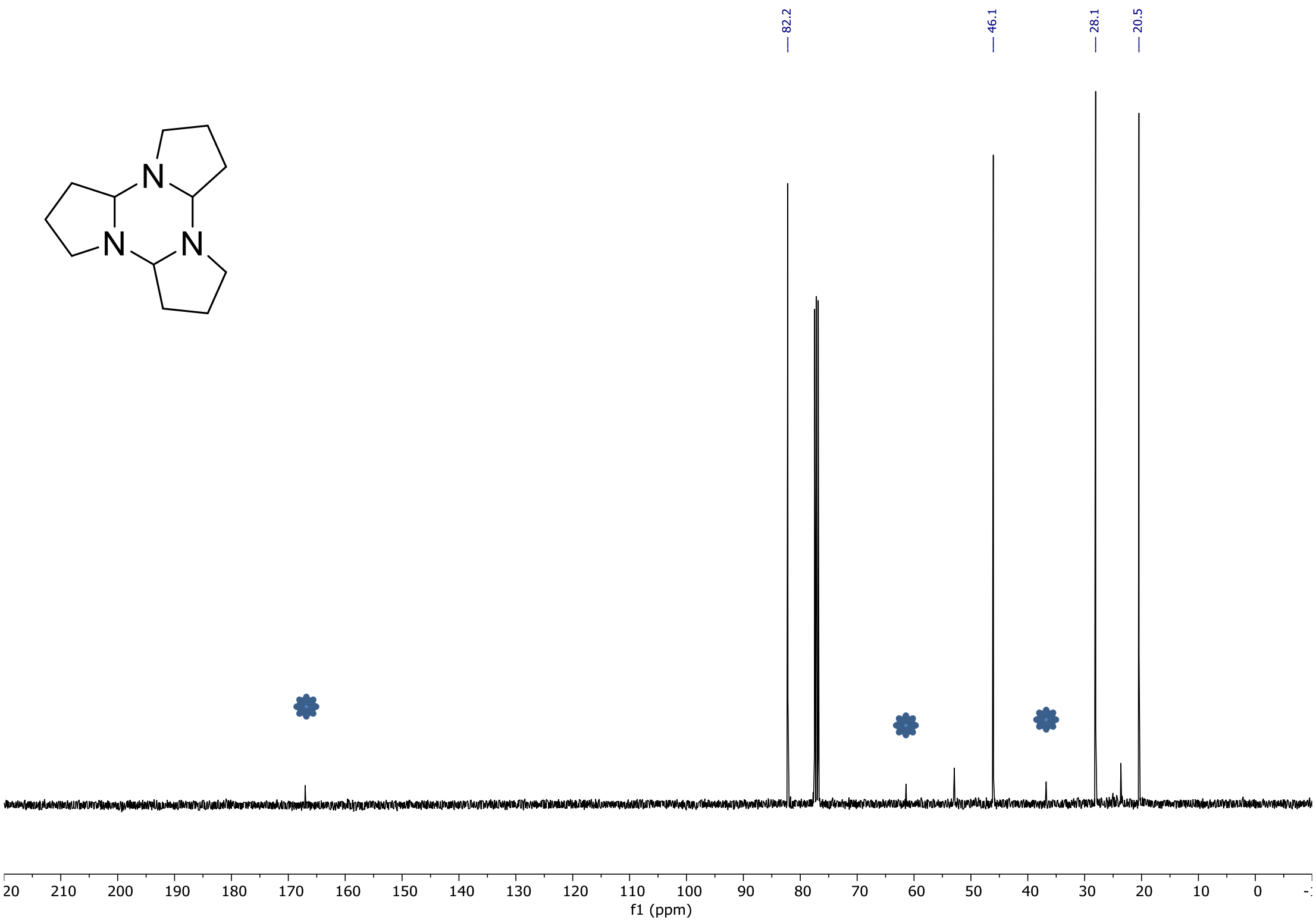

✽ = signals of Δ^1-pyrroline

^{1}H NMR (400 MHz, $CDCl_3$) of piperideine **1b**:

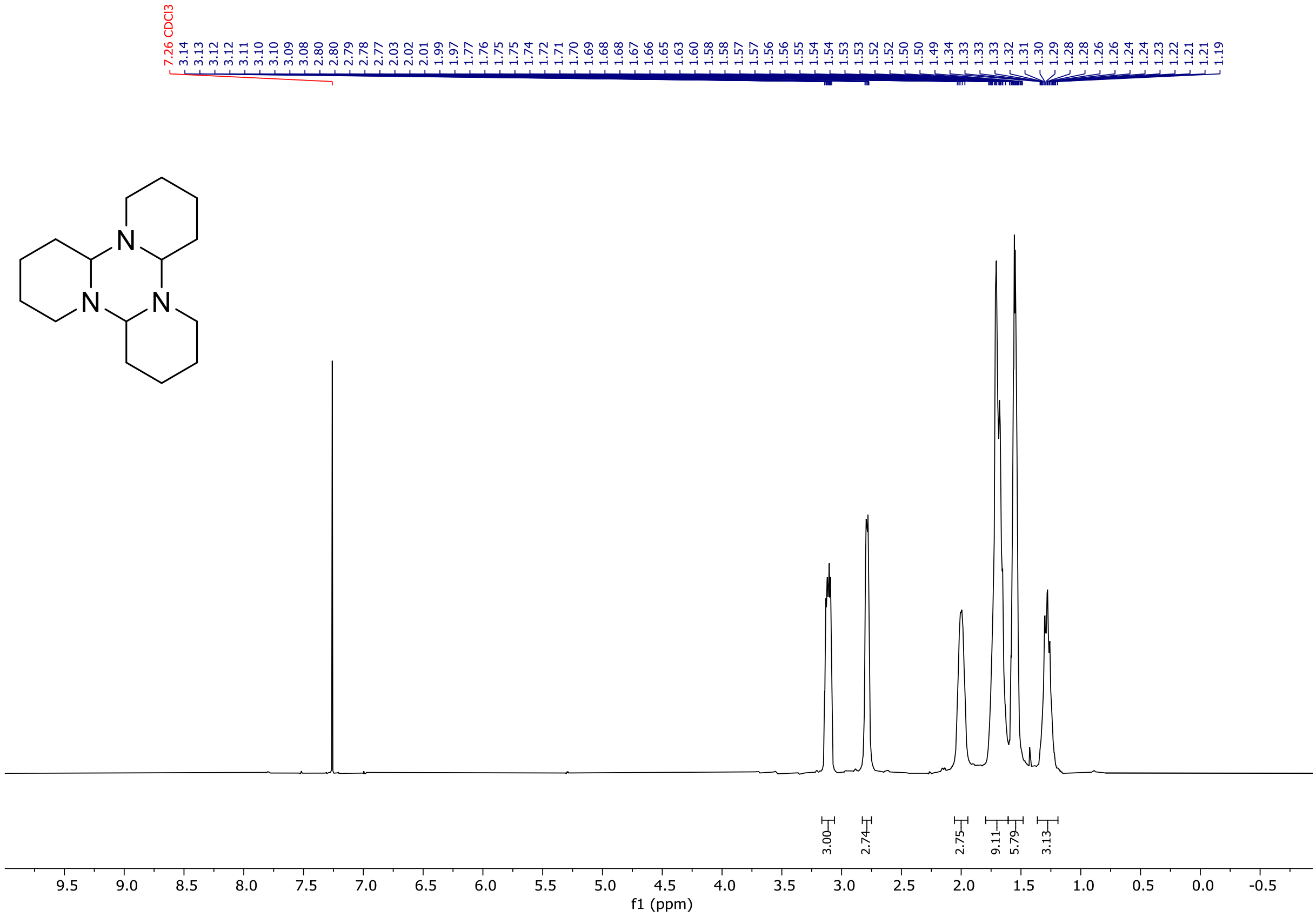

^{1}H NMR (400 MHz, $CDCl_3$) of piperideine **1b** after 6h:

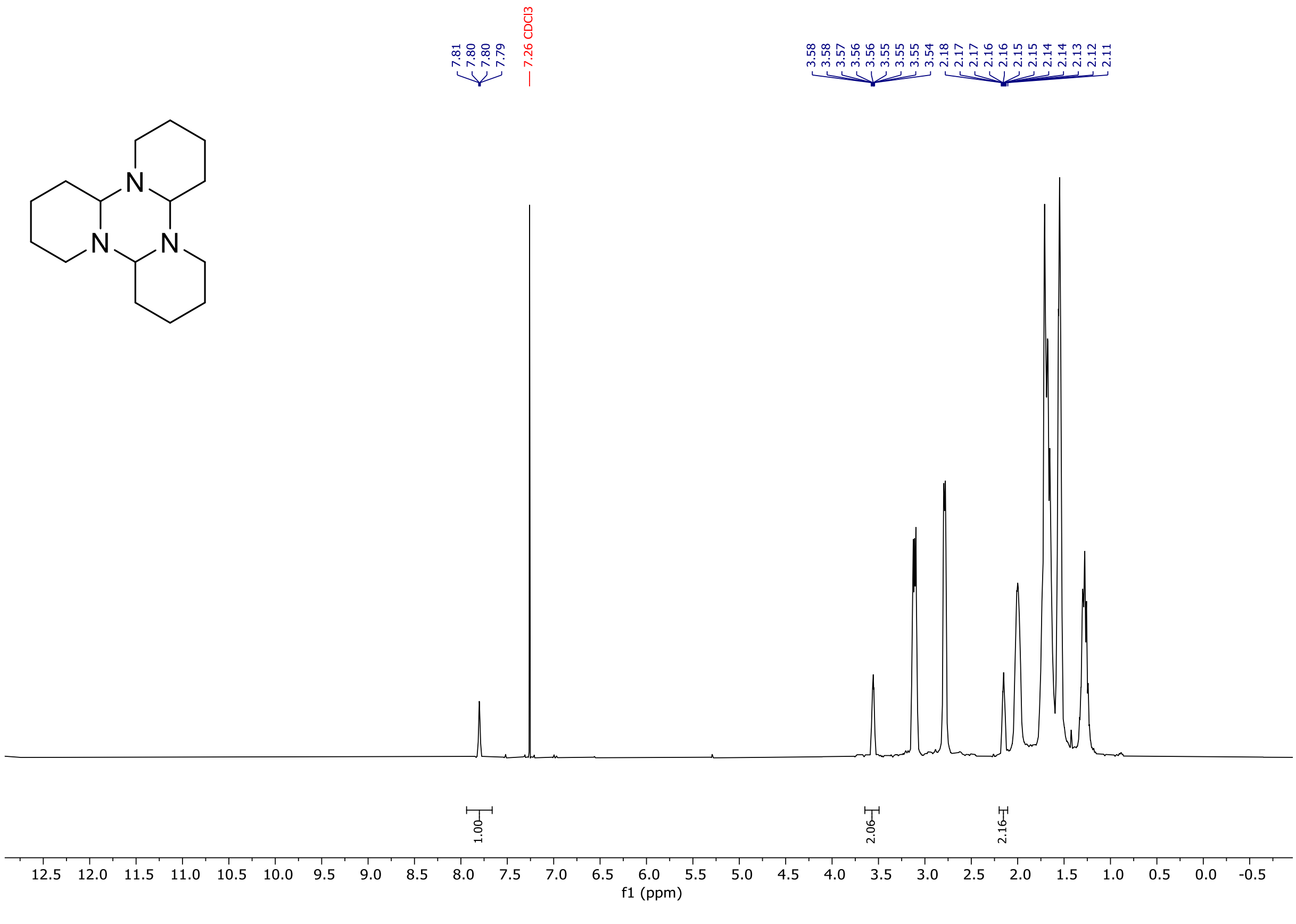

^{13}C NMR (101 MHz, CDCl3$_3$) of piperideine **1b**:

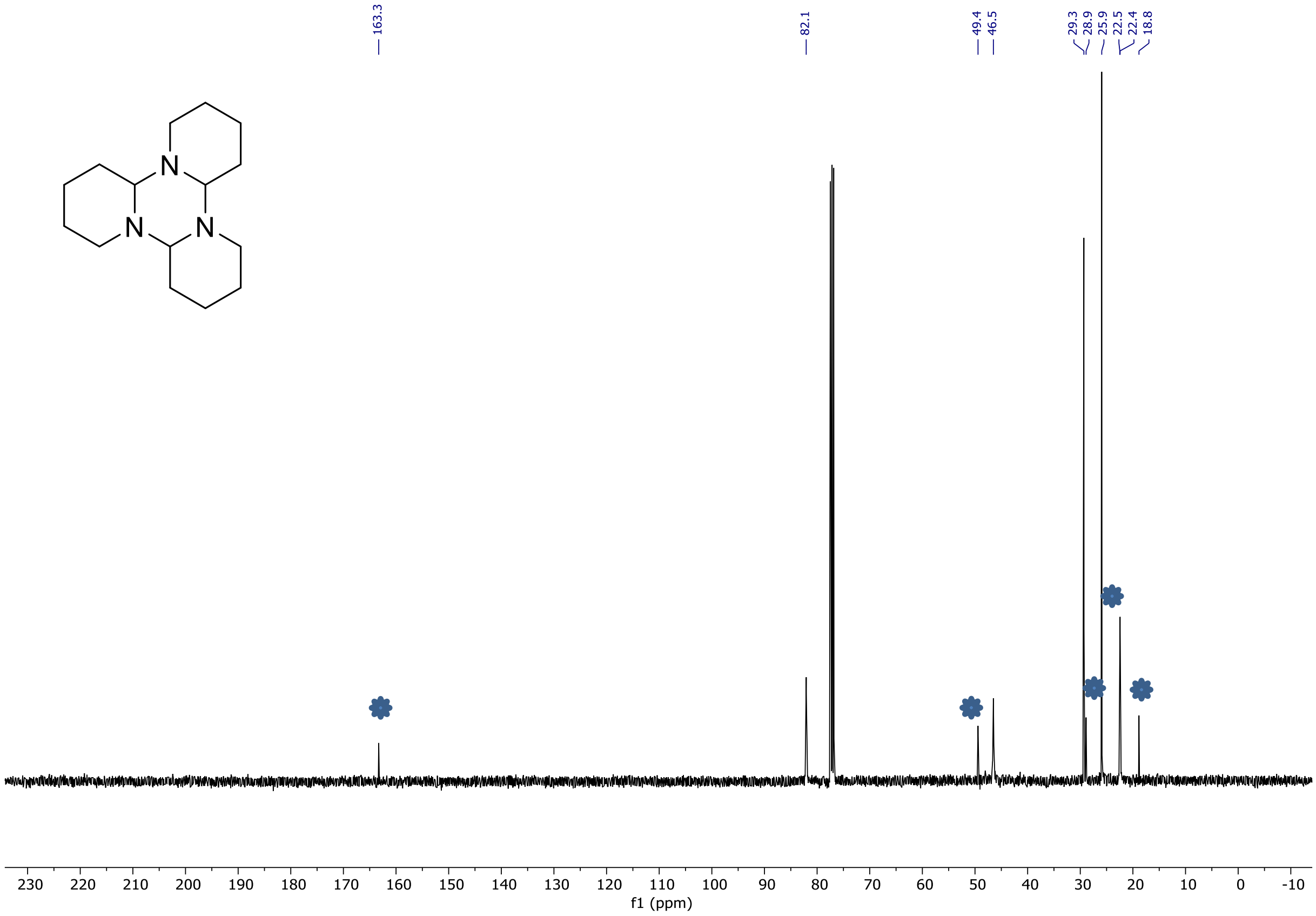

= signals of Δ^1-piperideine

8.2 NMR Spectra of Malonates

^{1}H NMR (400 MHz, $CDCl_3$) of **SI-2a**:

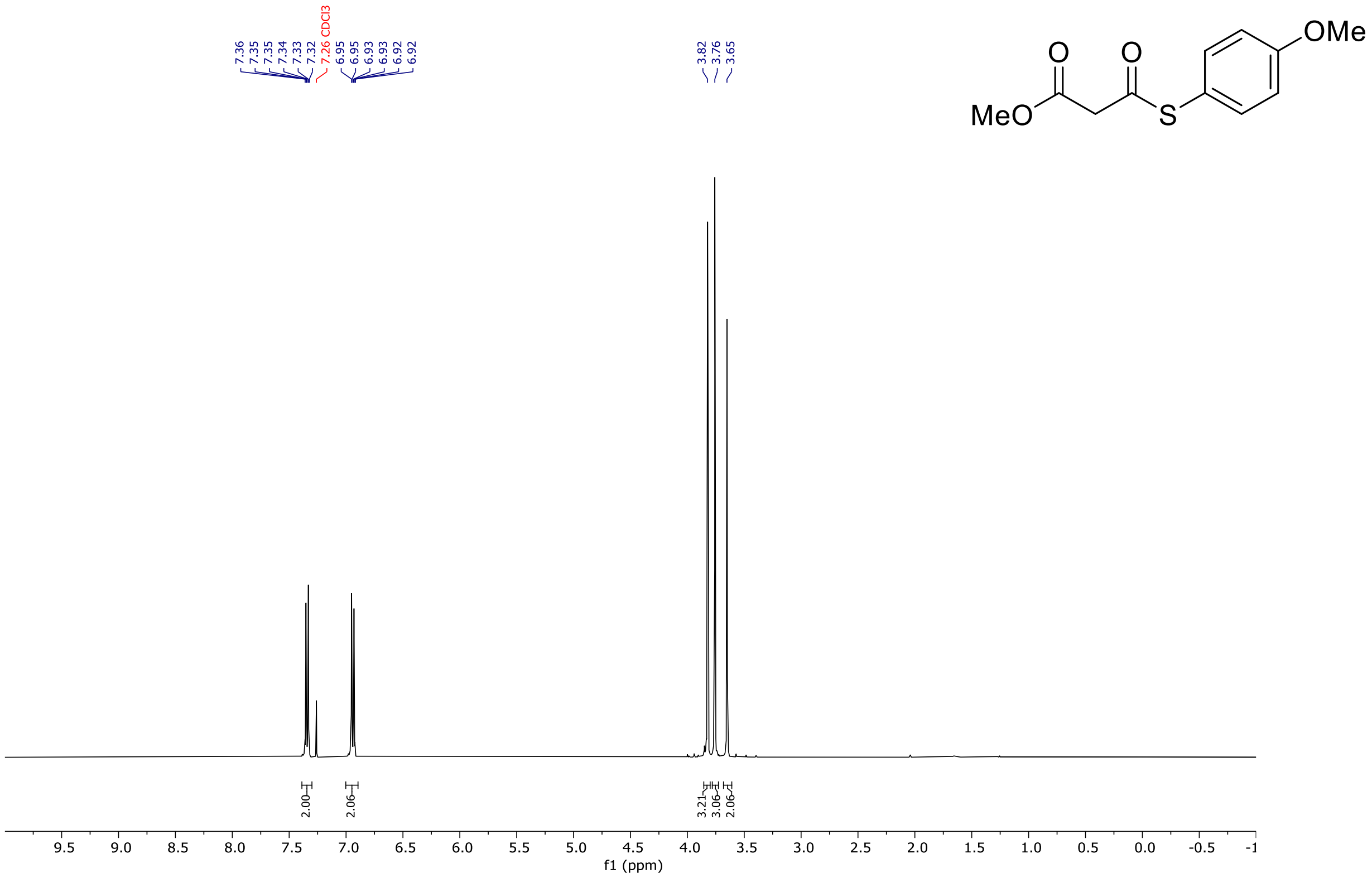

^{13}C NMR (101 MHz, $CDCl_3$) of **SI-2a**:

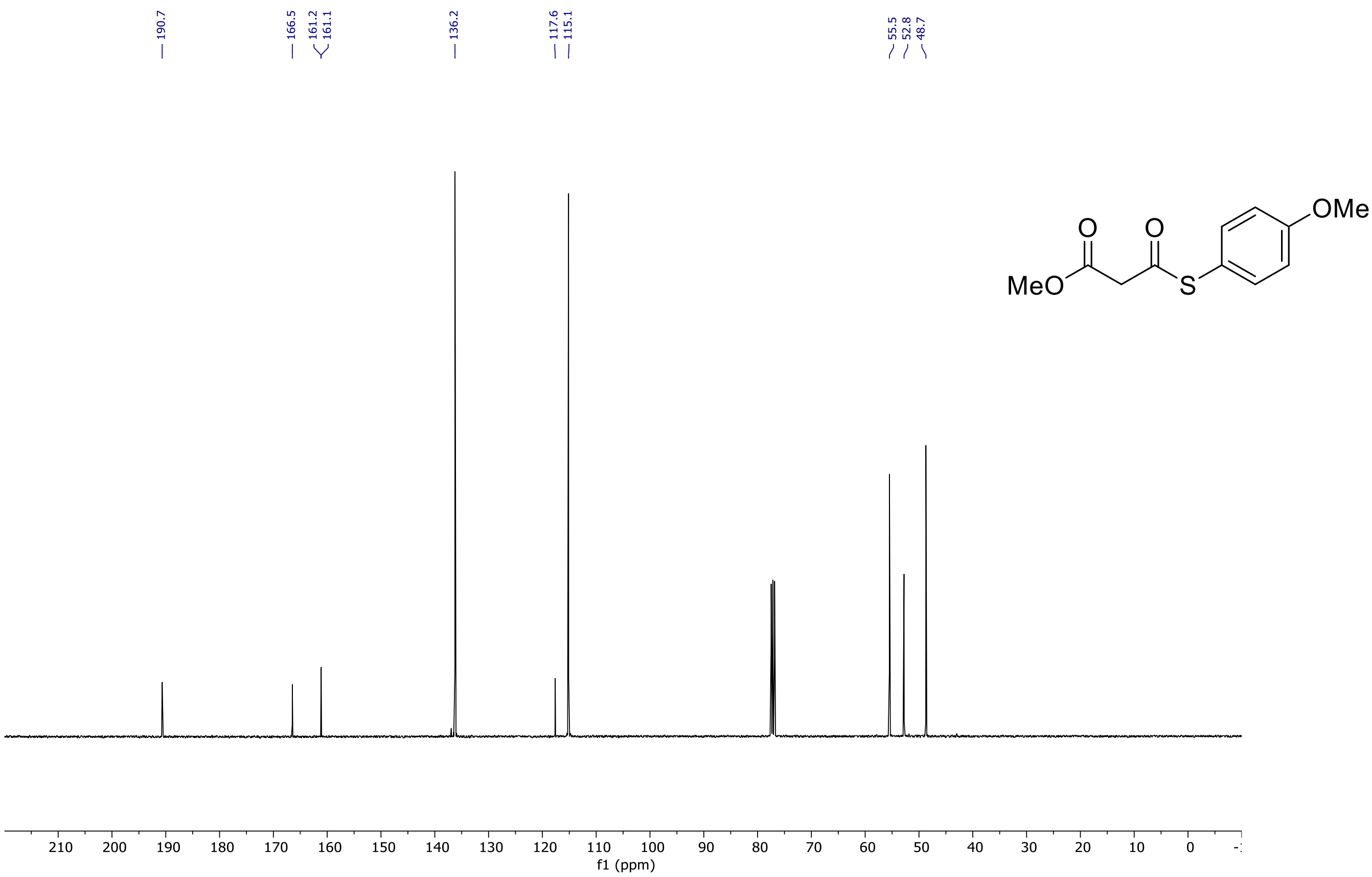

^{1}H NMR (400 MHz, $CDCl_3$) of **SI-2b**:

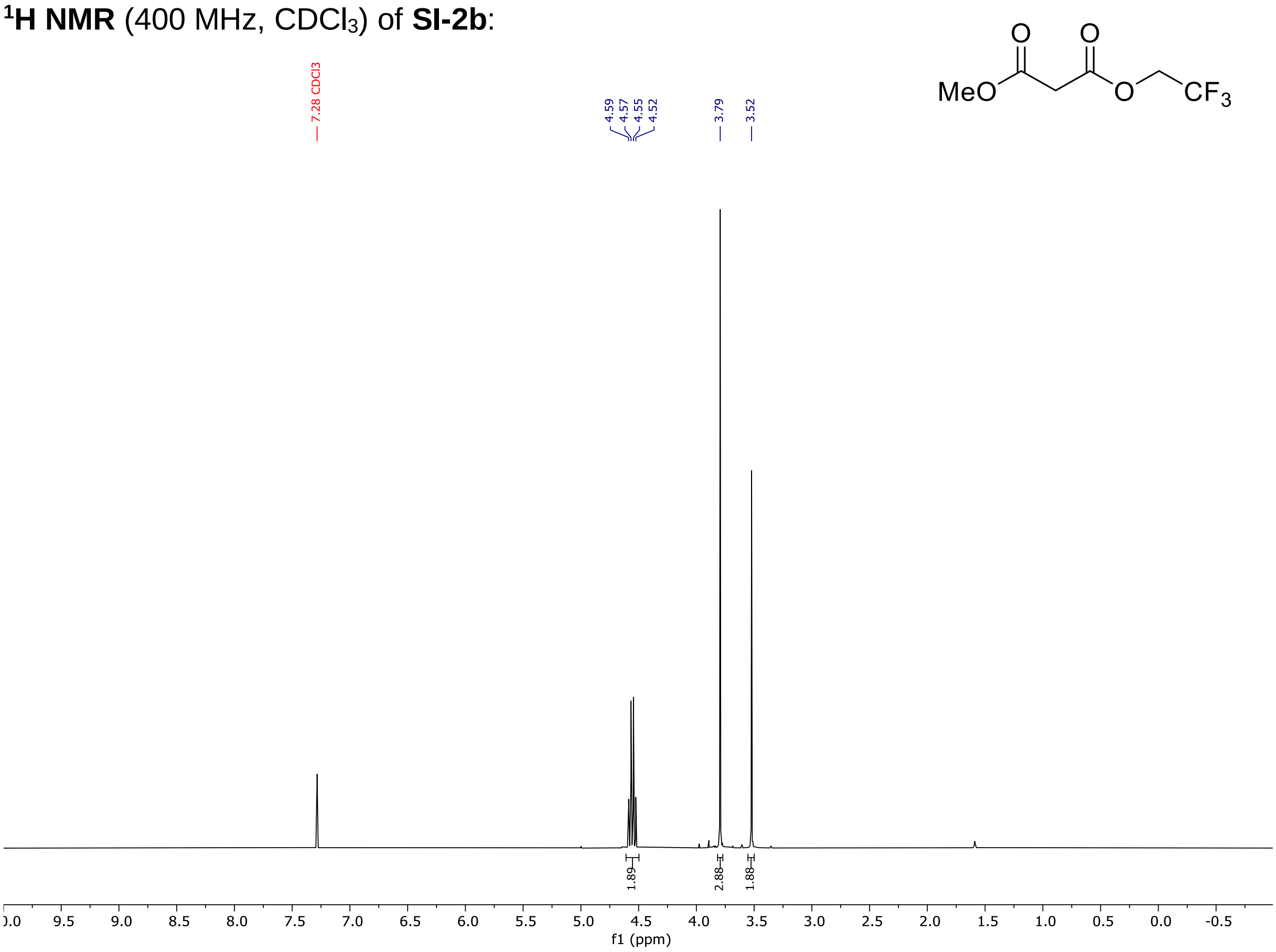

^{19}F NMR {^{1}H} (377 MHz, $CDCl_3$) of **SI-2b**:

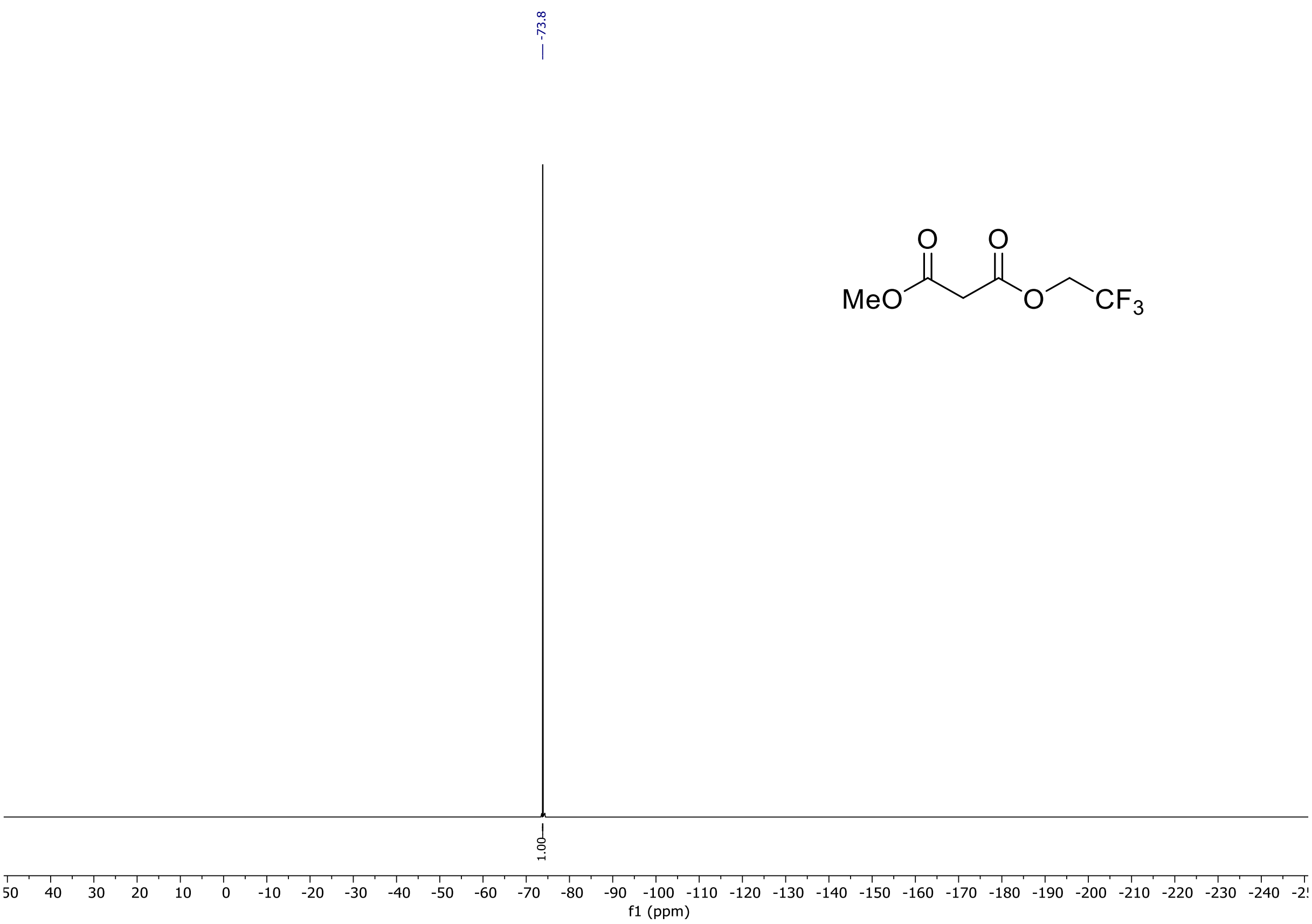

^{19}F NMR (377 MHz, $CDCl_3$) of **SI-2b**:

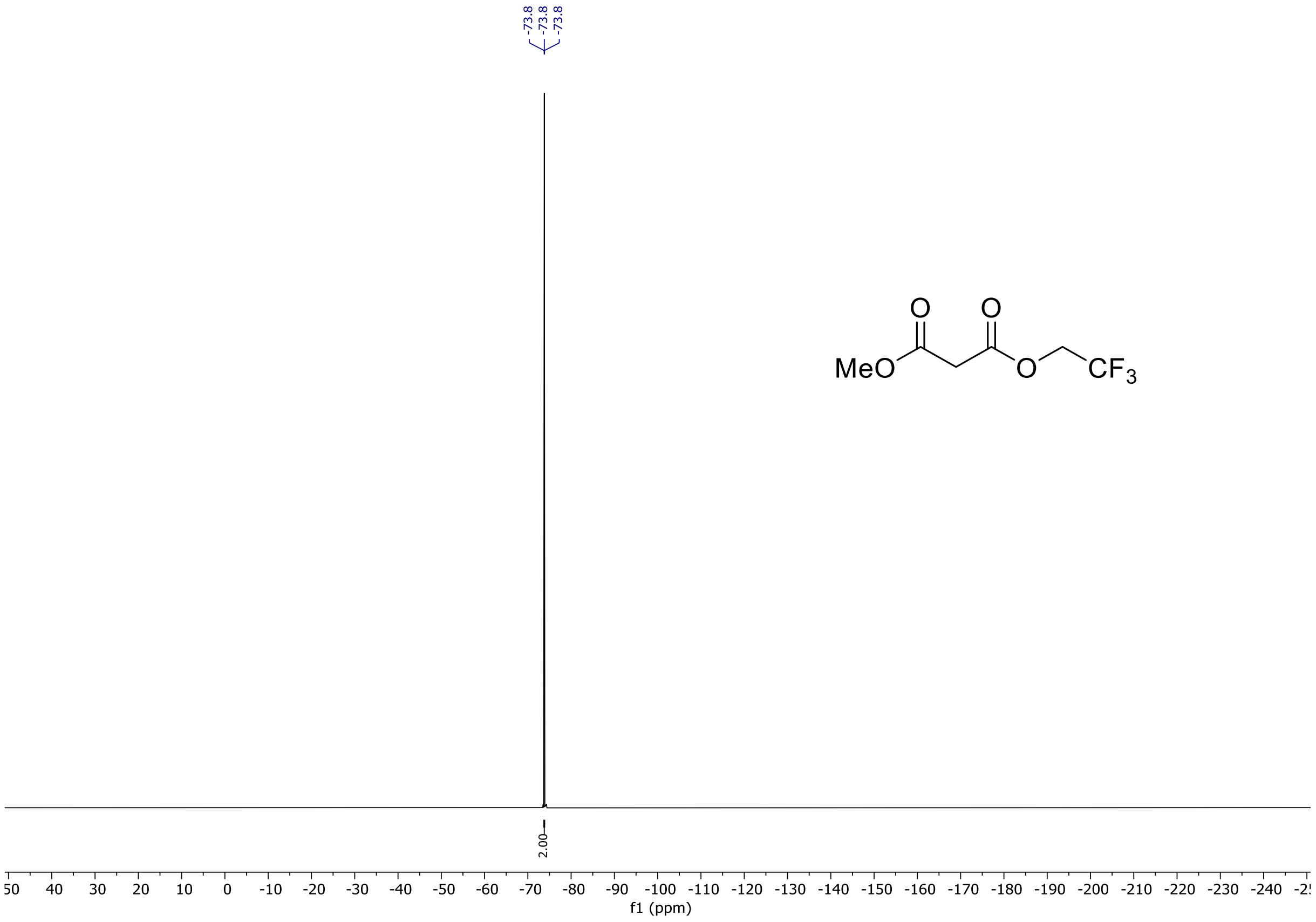

^{13}C NMR (101 MHz, $CDCl_3$) of **SI-2b**:

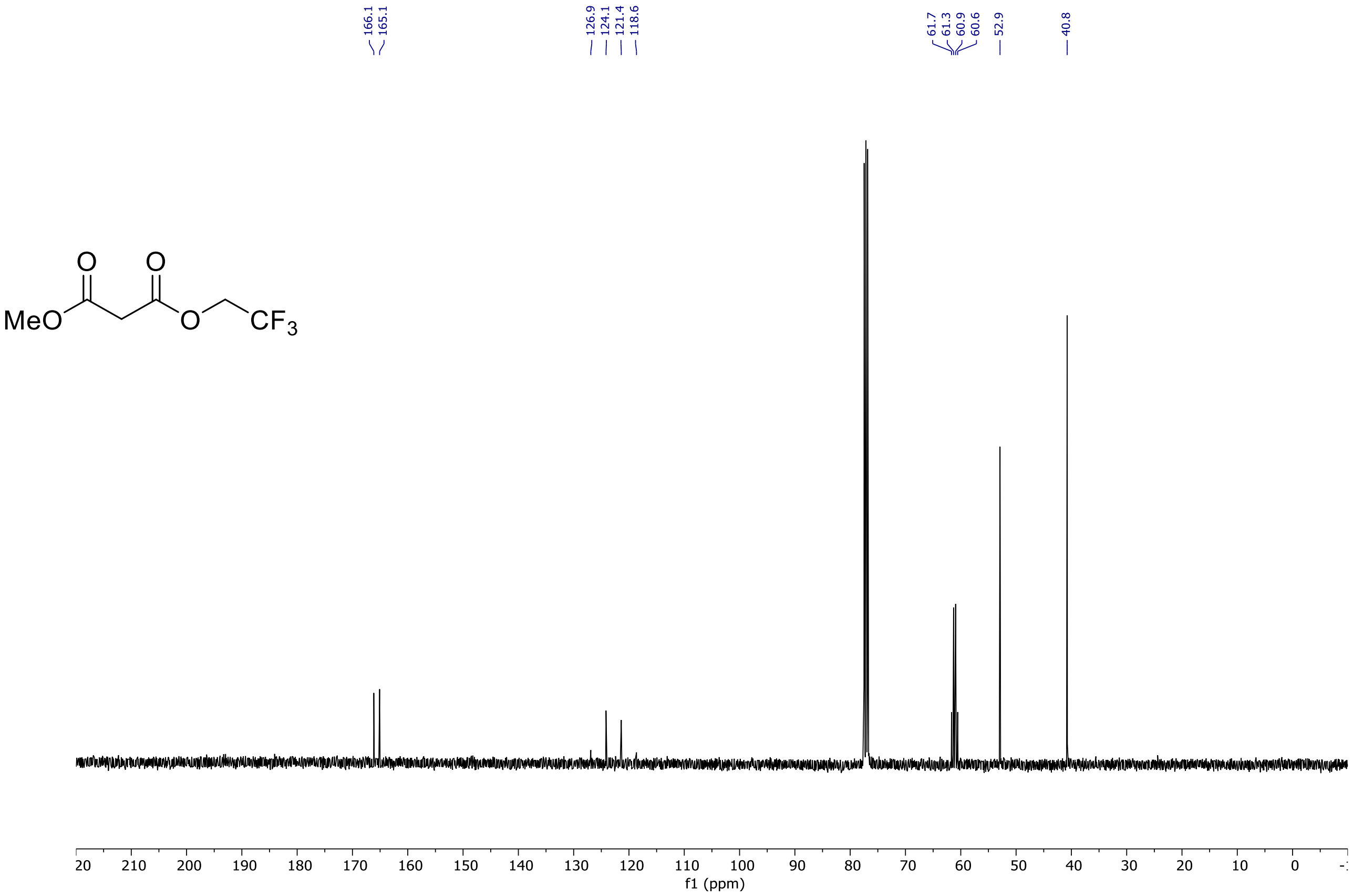

[1]**H NMR** (400 MHz, $CDCl_3$) of **SI-2c**:

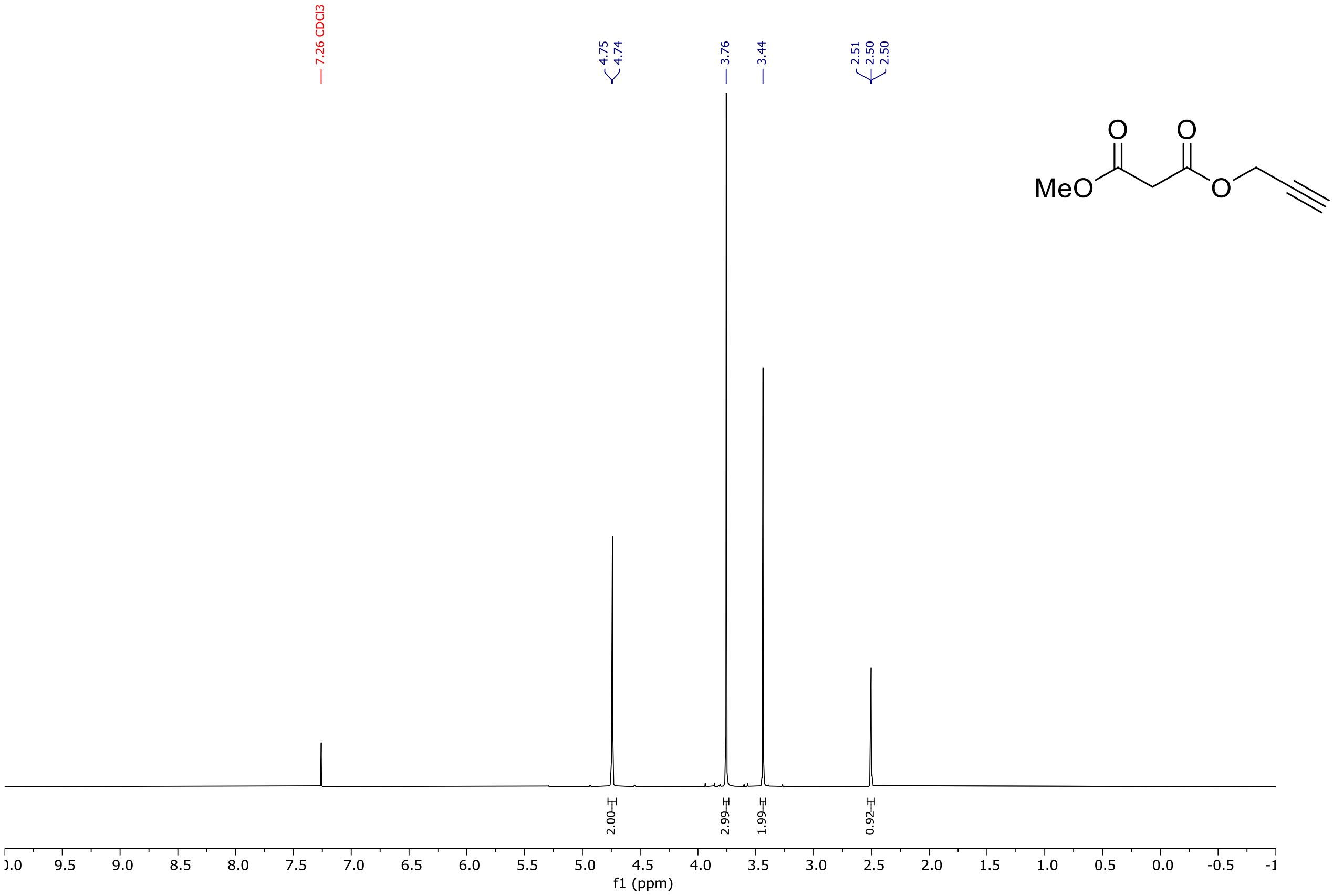

[13]**C NMR** (101 MHz, $CDCl_3$) of **SI-2c**:

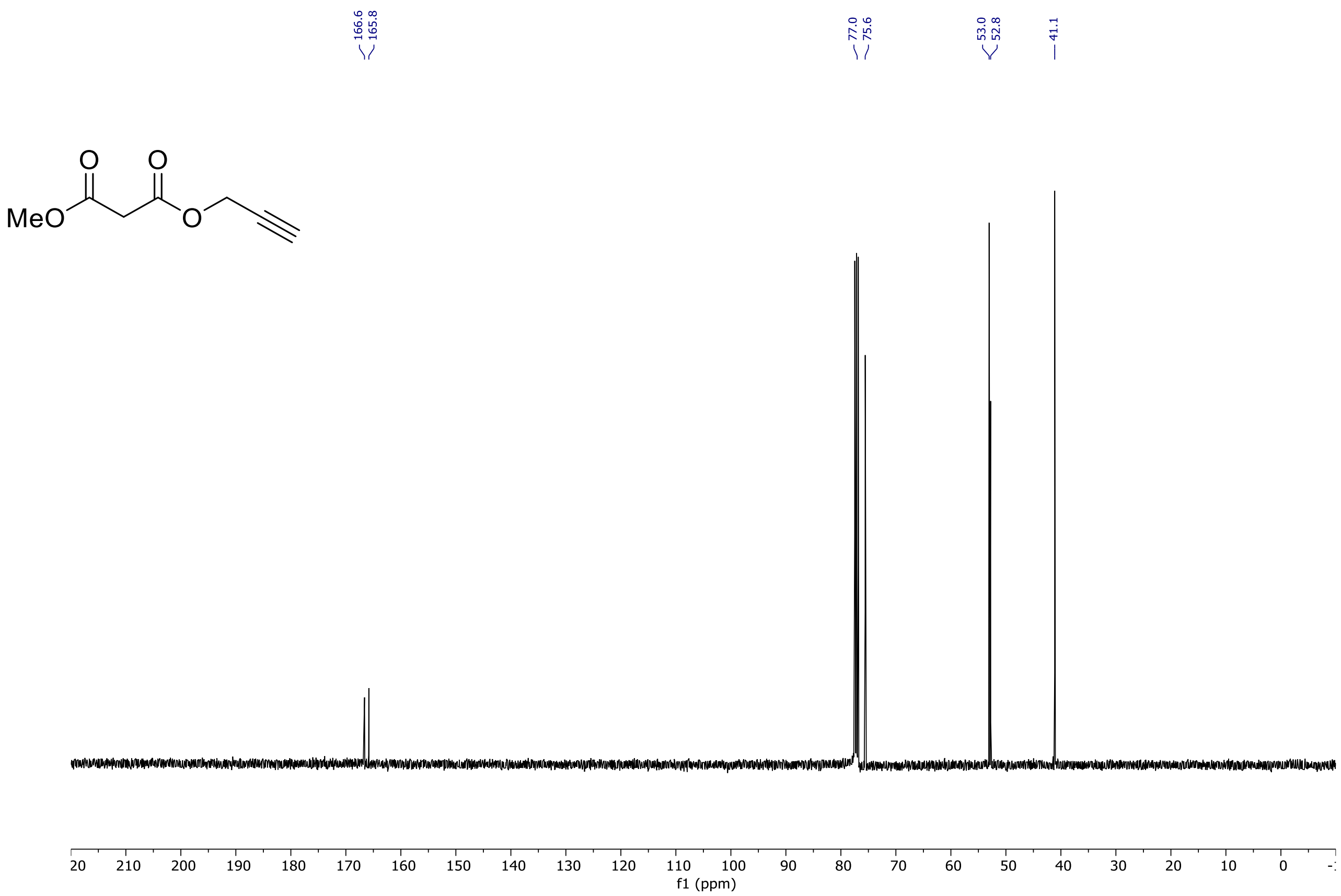

^{1}H NMR (400 MHz, $CDCl_3$) of **SI-2d**:

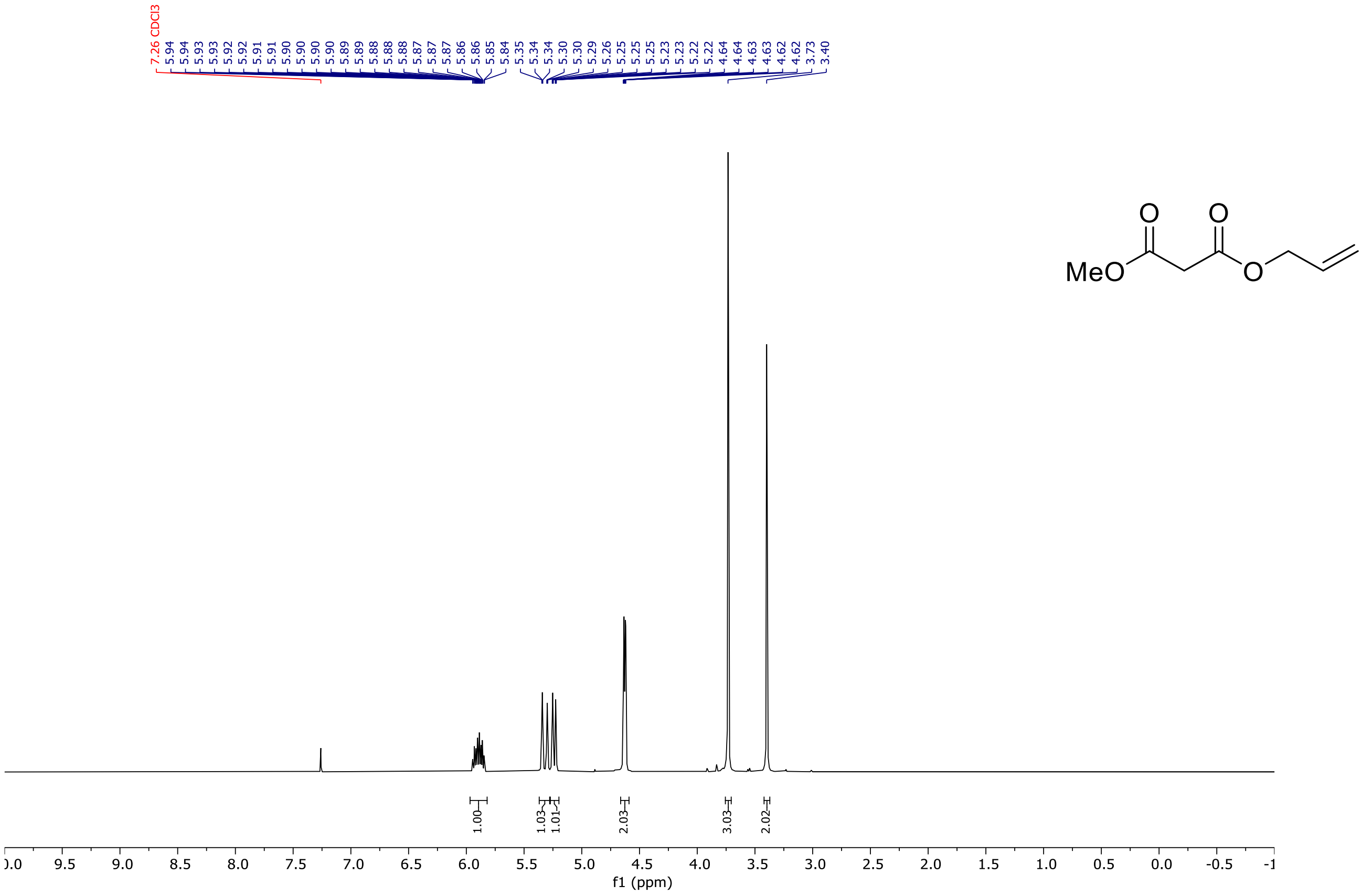

^{13}C NMR (101 MHz, $CDCl_3$) of **SI-2d**:

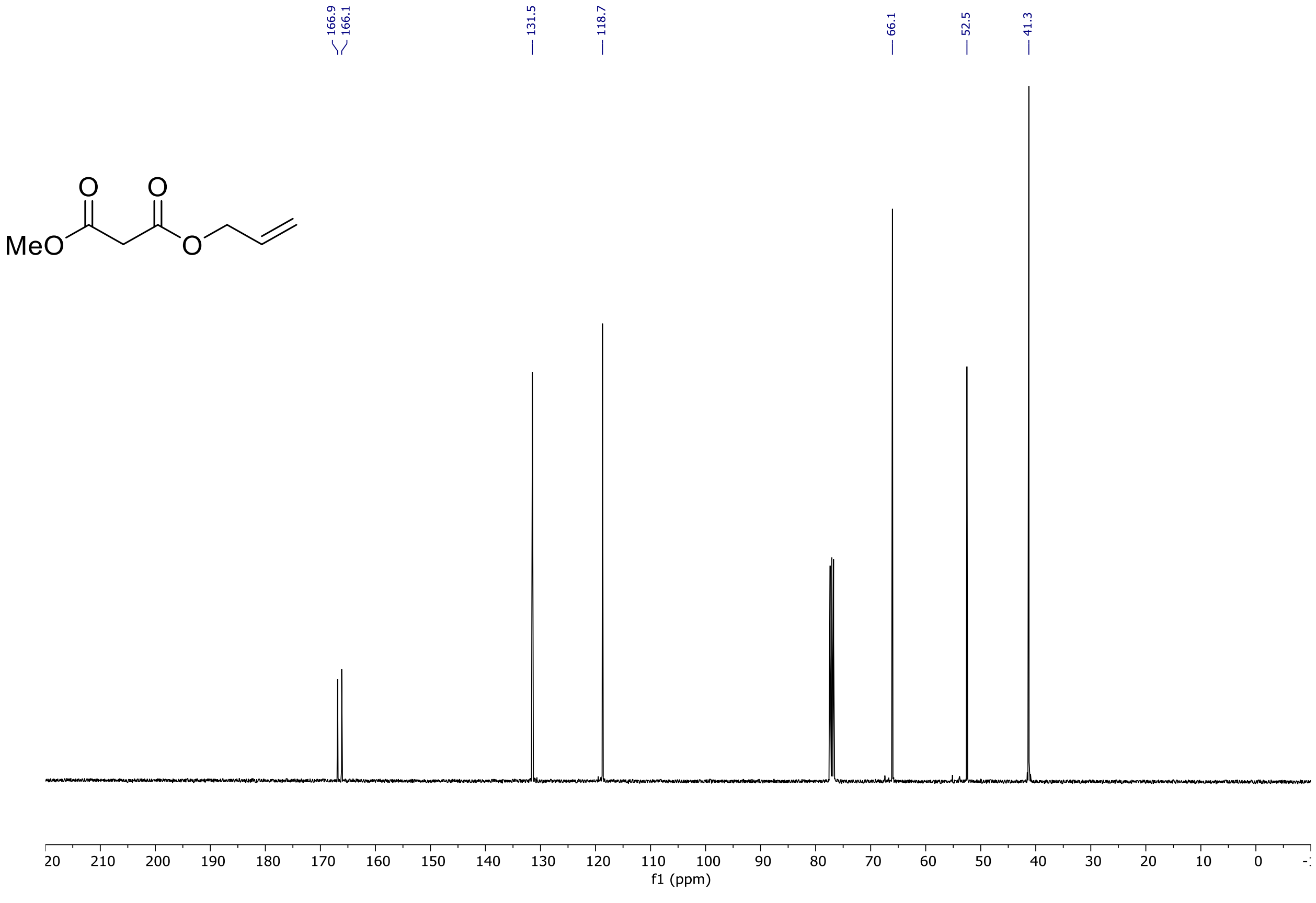

9 NMR Spectra and Chiral Stationary Phase HPLC Chromatograms of Products

9.1 NMR and chiral stationary phase of 5-6-5 products

^{1}H NMR (500 MHz, $CDCl_3$) of **3a**:

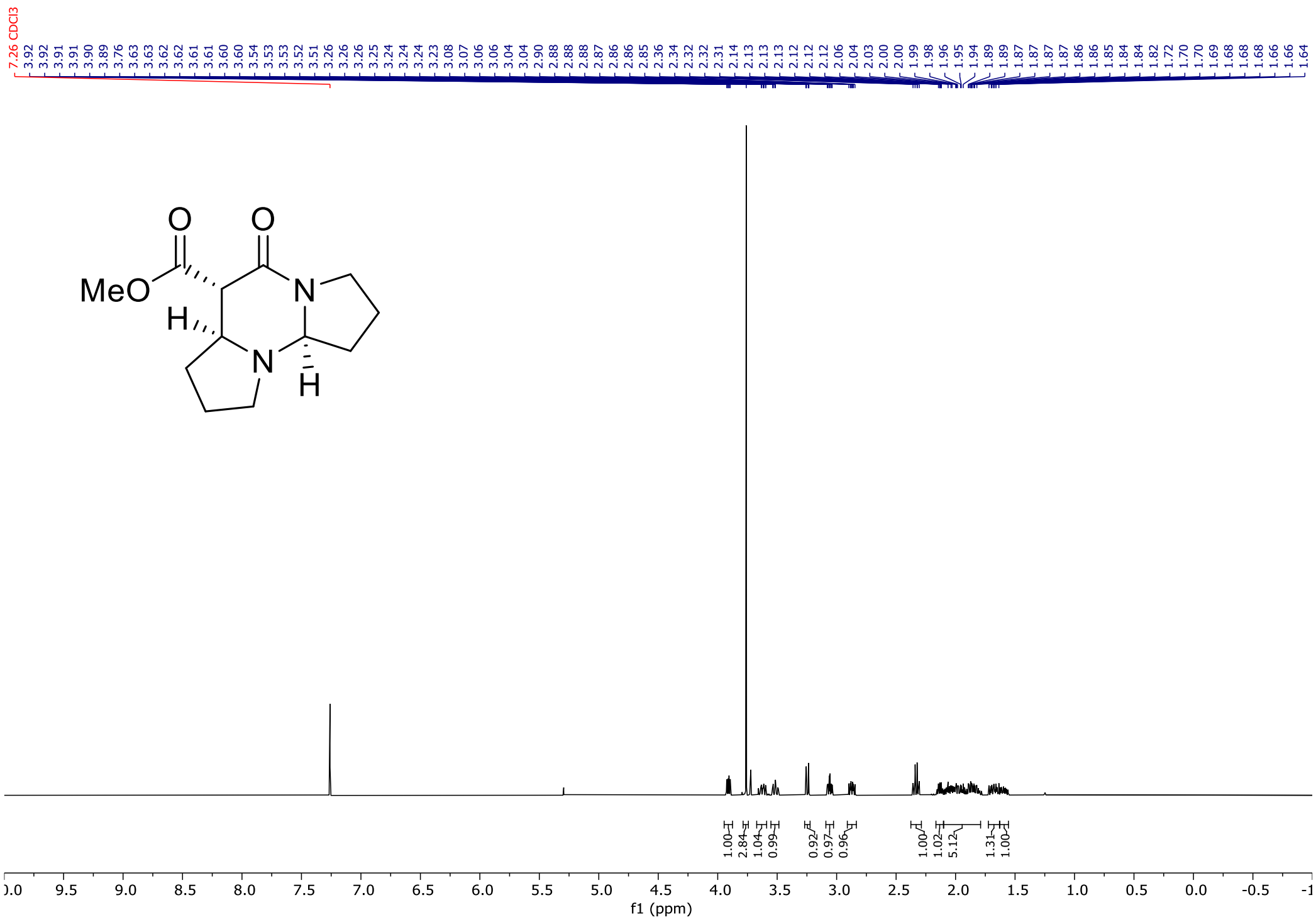

^{13}C NMR {^{1}H} (101 MHz, $CDCl_3$) of **3a**:

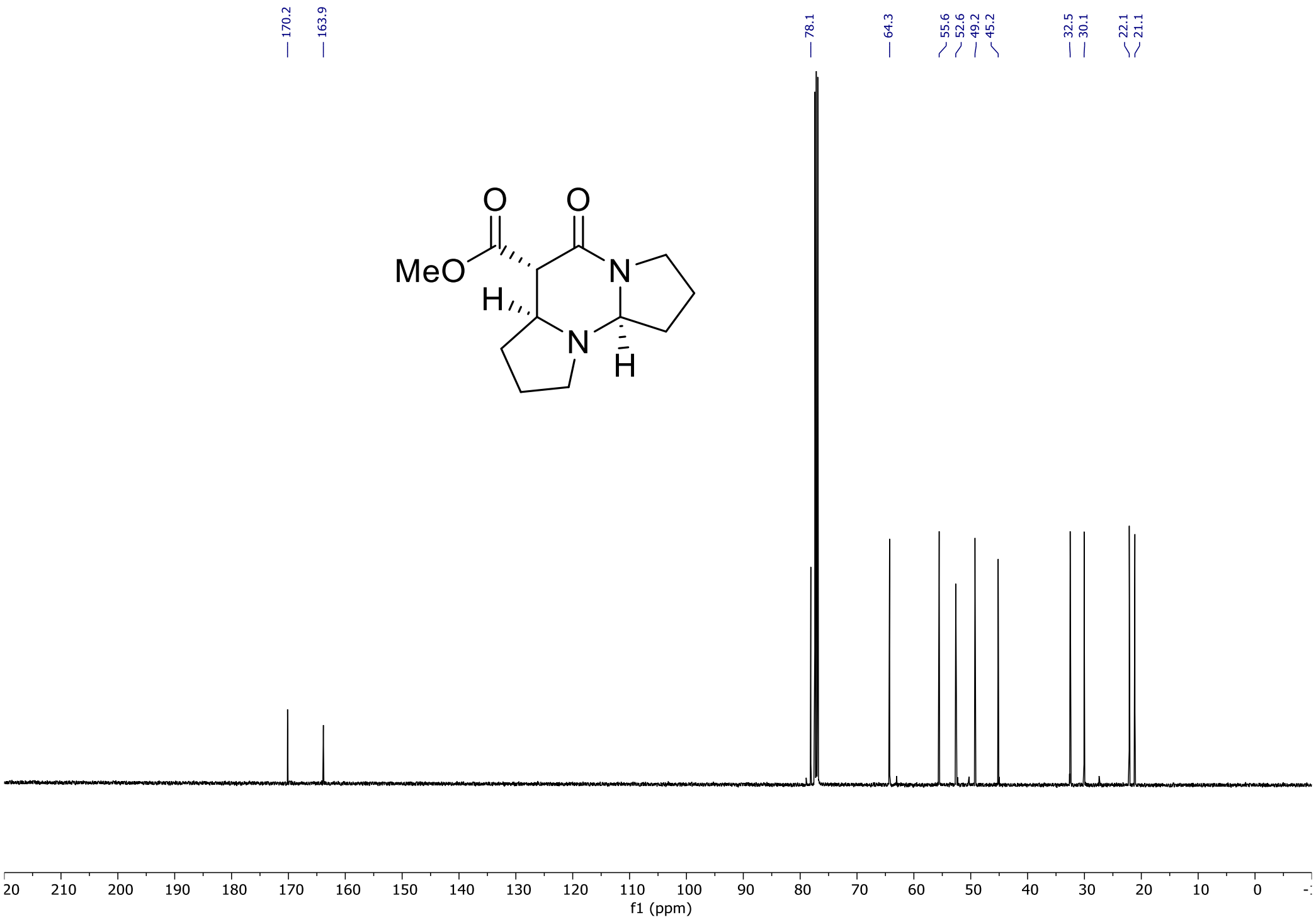

The enantiomeric access of **3a** was determined by chiral stationary phase HPLC: IBN-5 column, hexane/*i*-PrOH 70:30, 0.5 mL/min, 25 °C, 210 nm; 18.3 min (minor), 22.2 min (major).

racemate

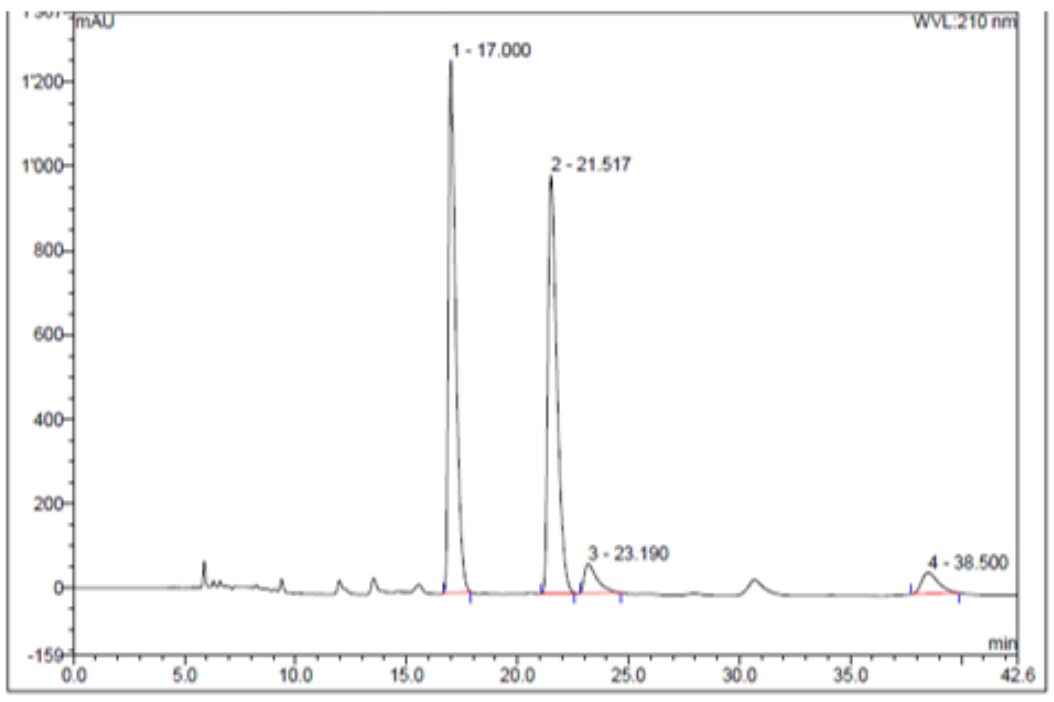

No.	Ret.Time min	Peak Name	Height mAU	Area mAU*min	Rel.Area %	Amount	Type
1	17.00	n.a.	1264.128	476.856	45.97	n.a.	BMB*
2	21.52	n.a.	994.732	469.144	45.23	n.a.	BM *
3	23.19	n.a.	69.810	46.859	4.52	n.a.	BMB*
4	38.50	n.a.	50.401	44.477	4.29	n.a.	BMB*
Total:			2379.071	1037.336	100.00	0.000	

Isolated product

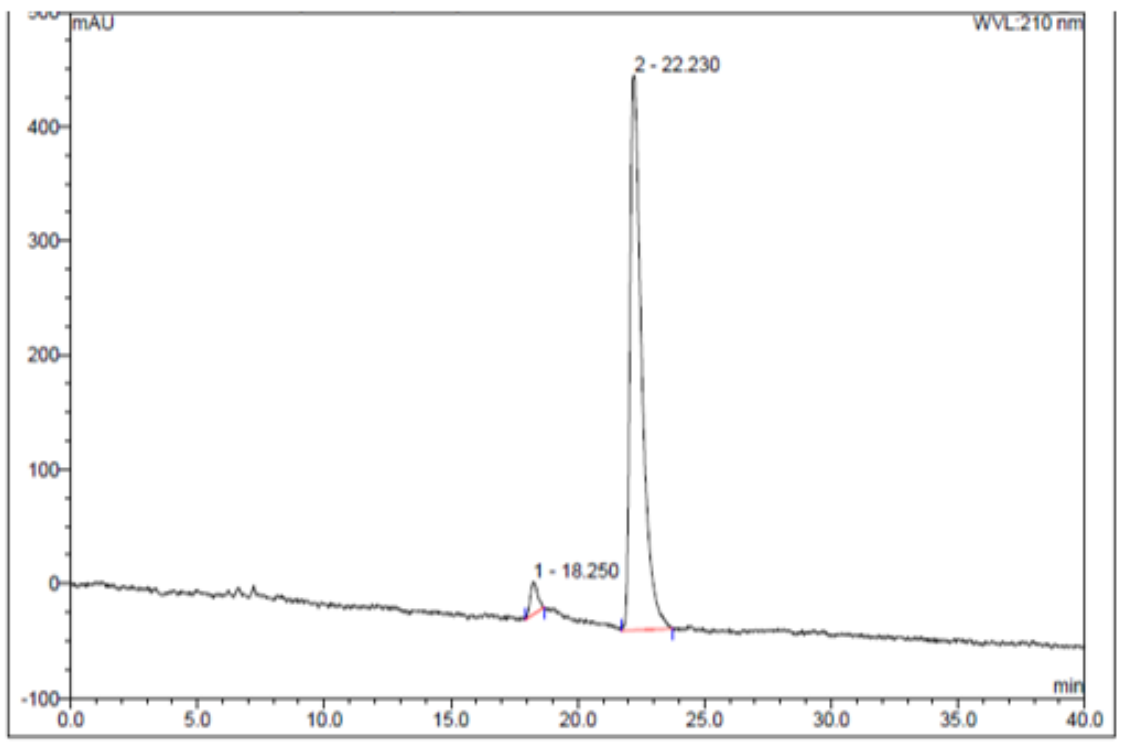

No.	Ret.Time min	Peak Name	Height mAU	Area mAU*min	Rel.Area %	Amount	Type
1	18.25	n.a.	28.398	9.464	3.49	n.a.	BMB*
2	22.23	n.a.	485.029	261.829	96.51	n.a.	BMB*
Total:			513.427	271.293	100.00	0.000	

^{1}H NMR (400 MHz, $CDCl_3$) of *ent*-**3a**:

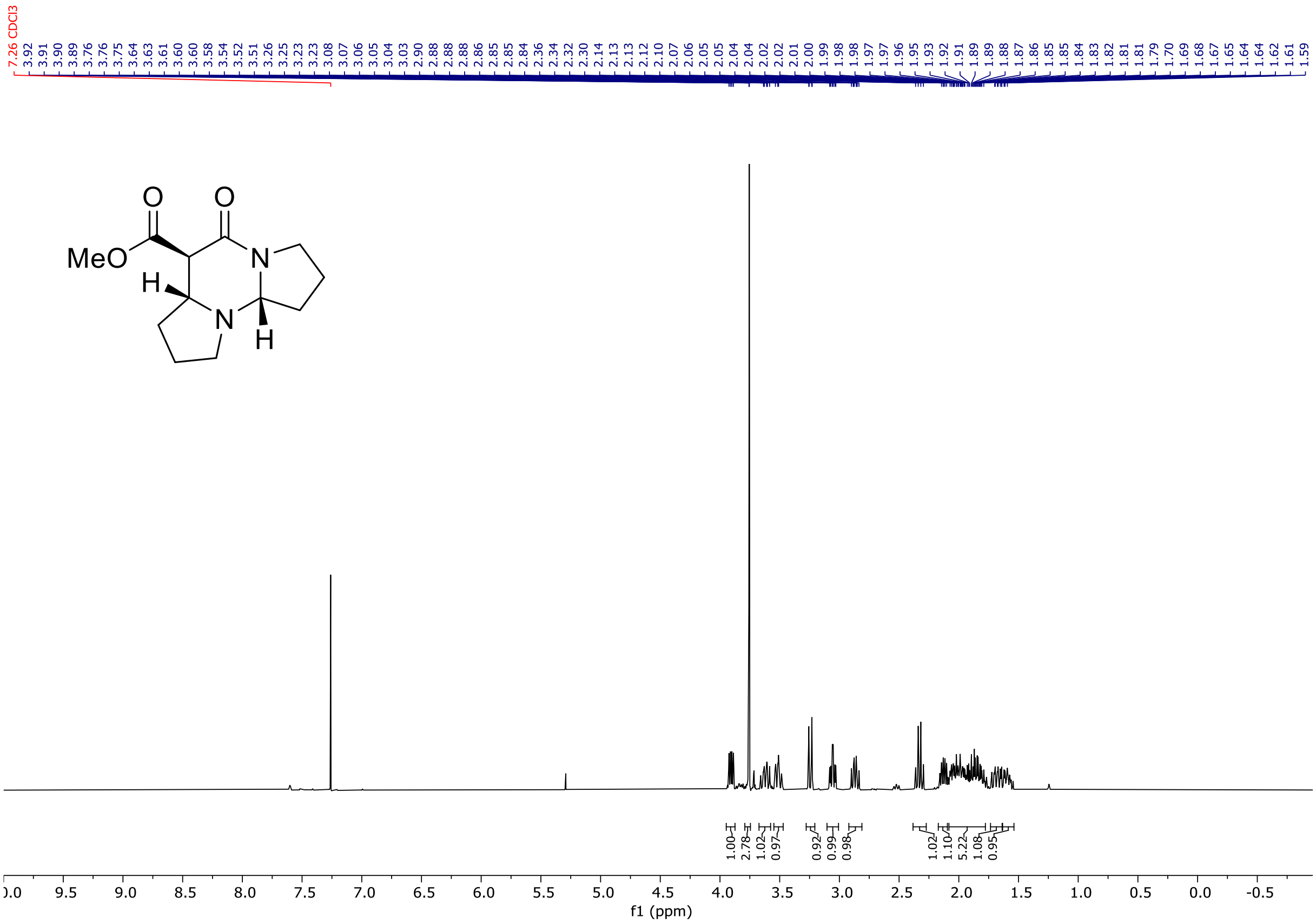

The enantiomeric access of *ent*-**3a** was determined by chiral stationary phase HPLC: IBN-5 column, hexane/*i*-PrOH 70:30, 0.5 mL/min, 25 °C, 210 nm; 17.1 min (major), 21.8 min (minor).

racemate

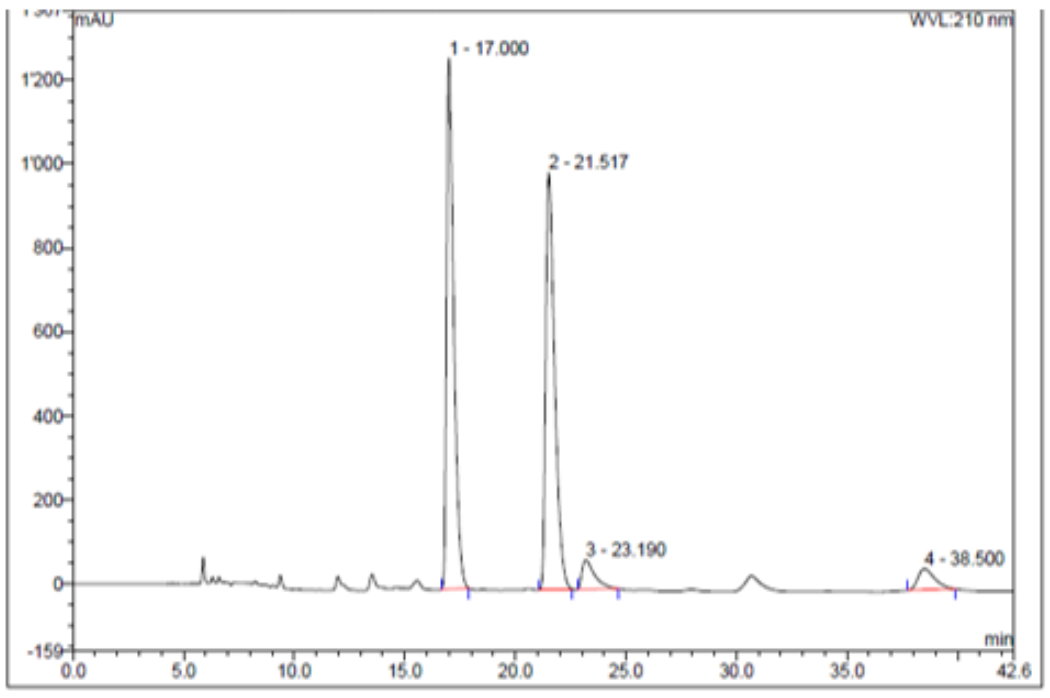

No.	Ret.Time min	Peak Name	Height mAU	Area mAU*min	Rel.Area %	Amount	Type
1	17.00	n.a.	1264.128	476.856	45.97	n.a.	BMB*
2	21.52	n.a.	994.732	469.144	45.23	n.a.	BM *
3	23.19	n.a.	69.810	46.859	4.52	n.a.	BMB*
4	38.50	n.a.	50.401	44.477	4.29	n.a.	BMB*
Total:			2379.071	1037.336	100.00	0.000	

Isolated product

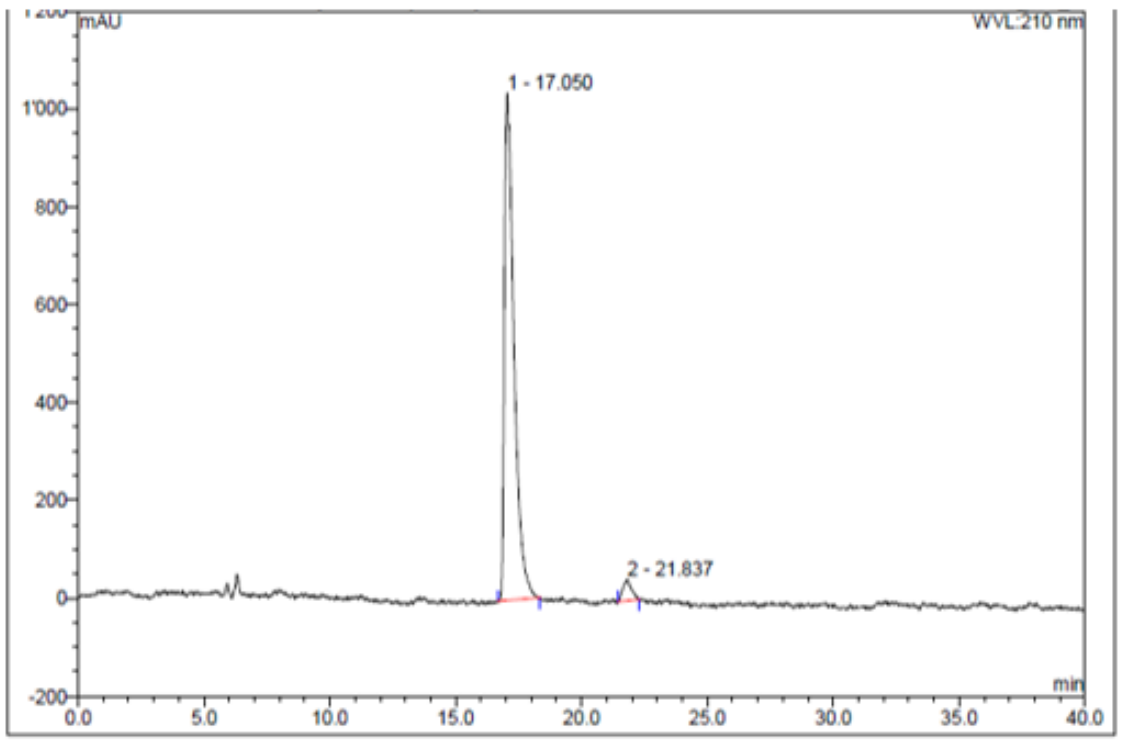

No.	Ret.Time min	Peak Name	Height mAU	Area mAU*min	Rel.Area %	Amount	Type
1	17.05	n.a.	1037.262	458.278	96.37	n.a.	BMB*
2	21.84	n.a.	43.926	17.266	3.63	n.a.	BMB*
Total:			1081.188	475.544	100.00	0.000	

^{1}H NMR (400 MHz, $CDCl_3$) of **3b**:

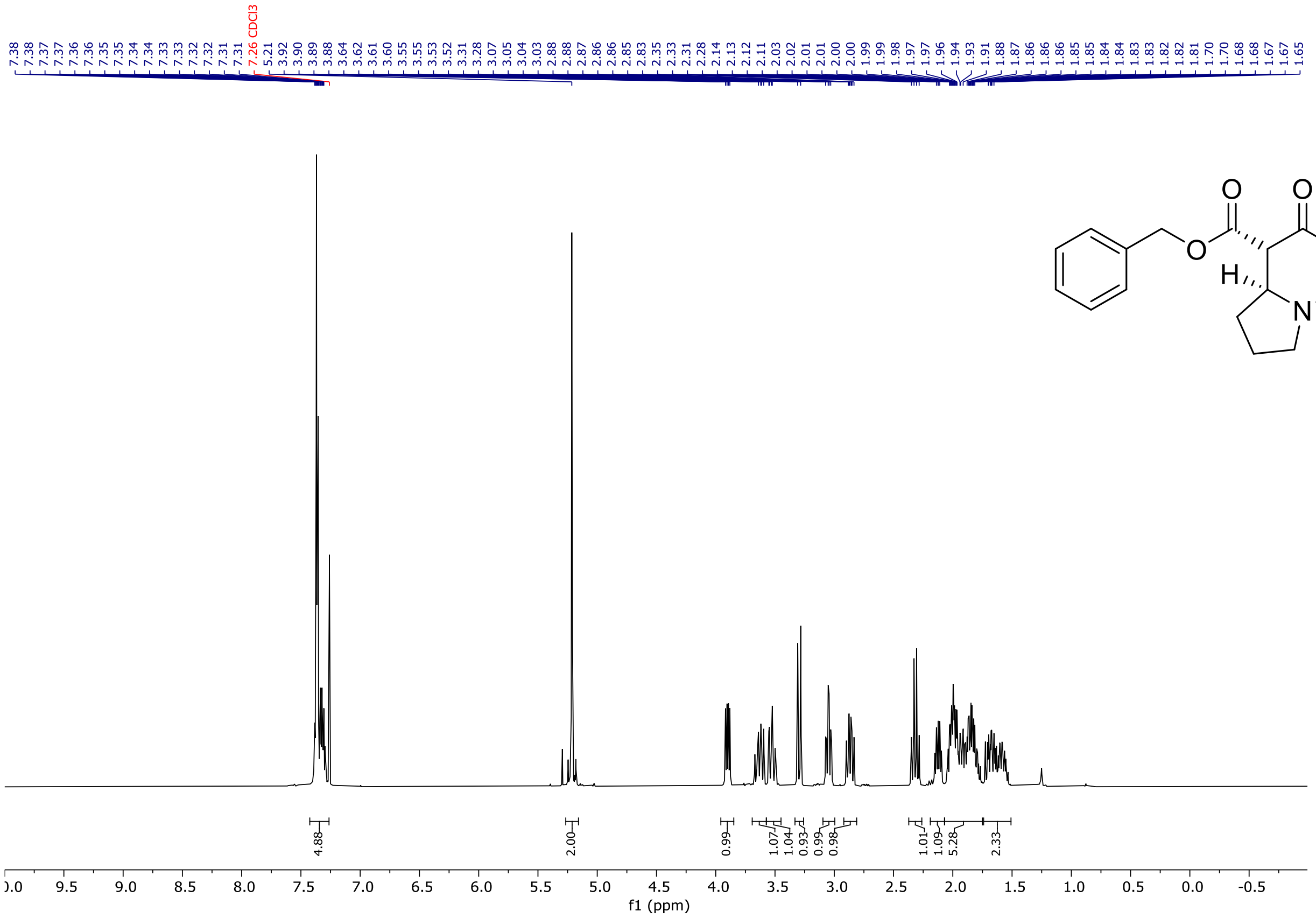

^{13}C NMR (101 MHz, $CDCl_3$) of **3b**:

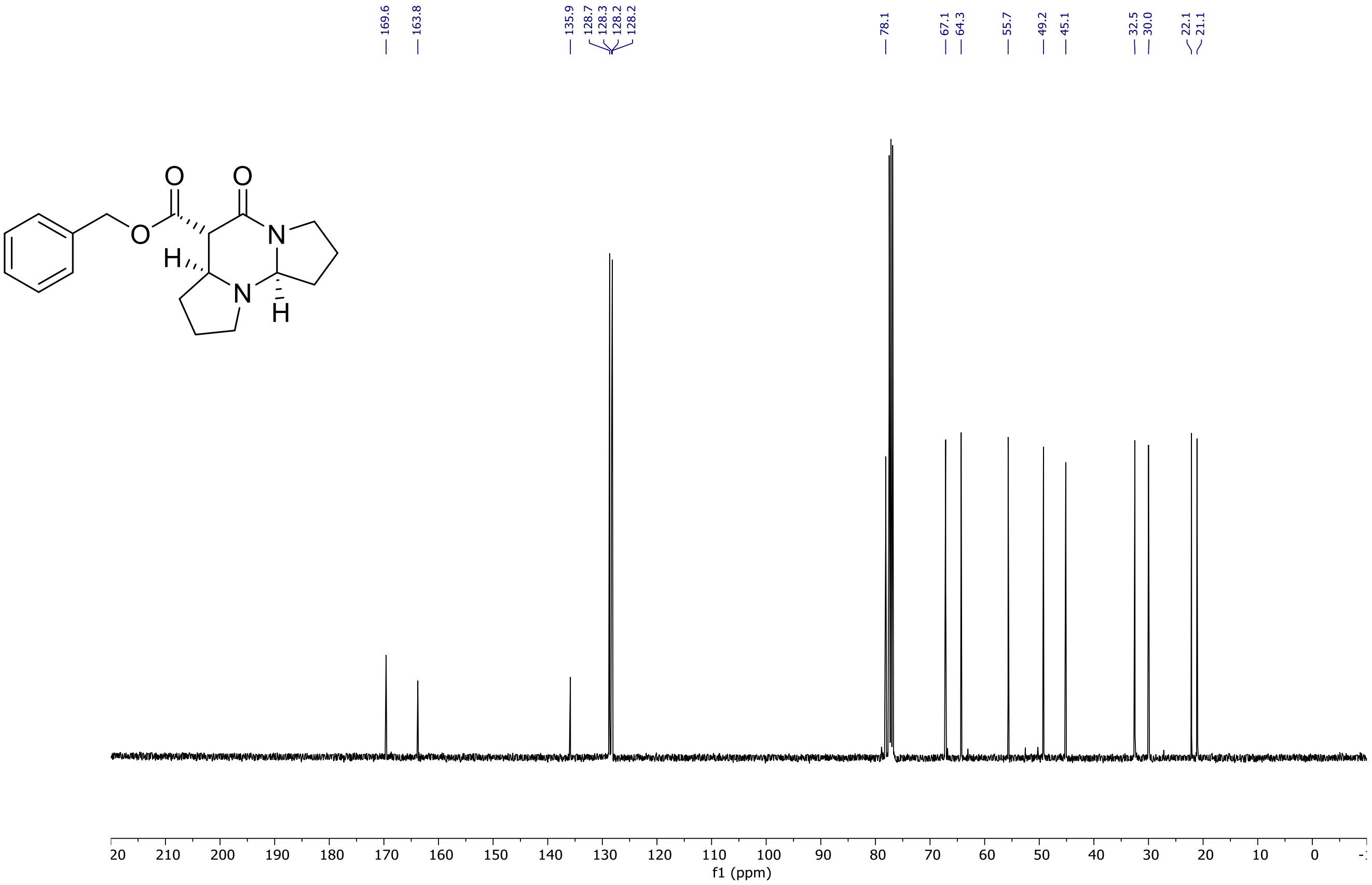

The enantiomeric access of **3b** was determined by chiral stationary phase HPLC: OD column, hexane/*i*-PrOH 85:15, 0.5 mL/min, 25 °C, 210 nm; 33.1 min (minor), 39.2 min (major).

racemate

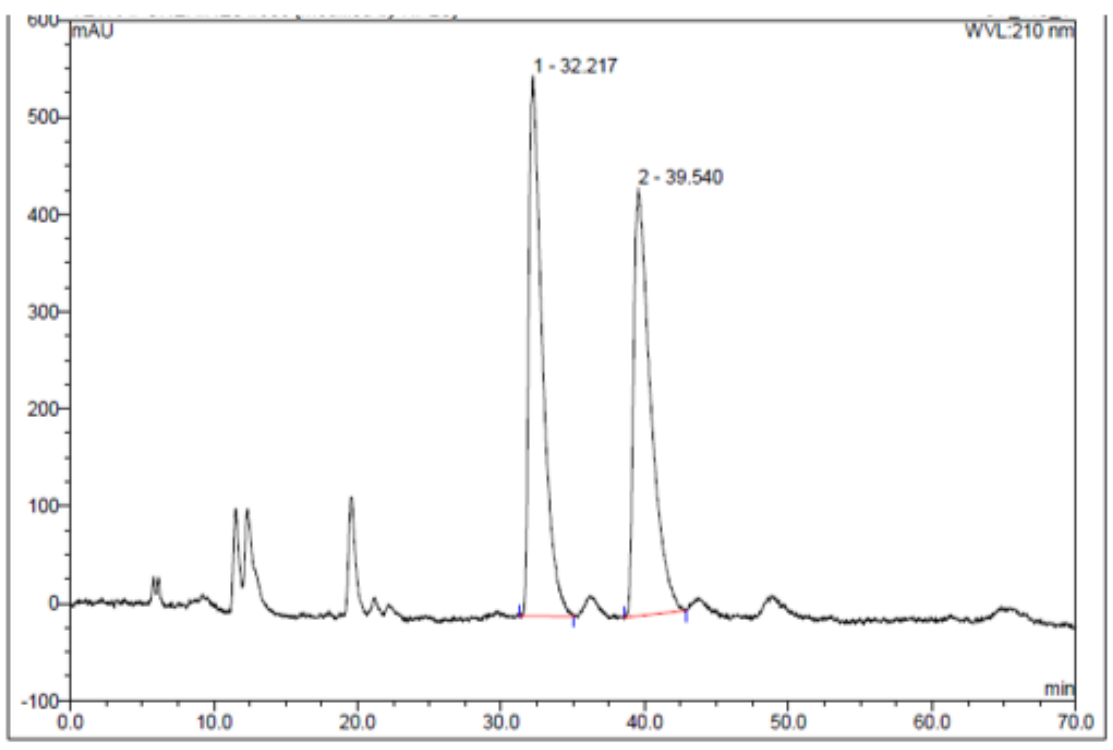

No.	Ret.Time min	Peak Name	Height mAU	Area mAU*min	Rel.Area %	Amount	Type
1	32.22	n.a.	555.825	606.892	50.08	n.a.	BMB*
2	39.54	n.a.	440.095	604.863	49.92	n.a.	BMB*
Total:			995.920	1211.755	100.00	0.000	

Isolated product

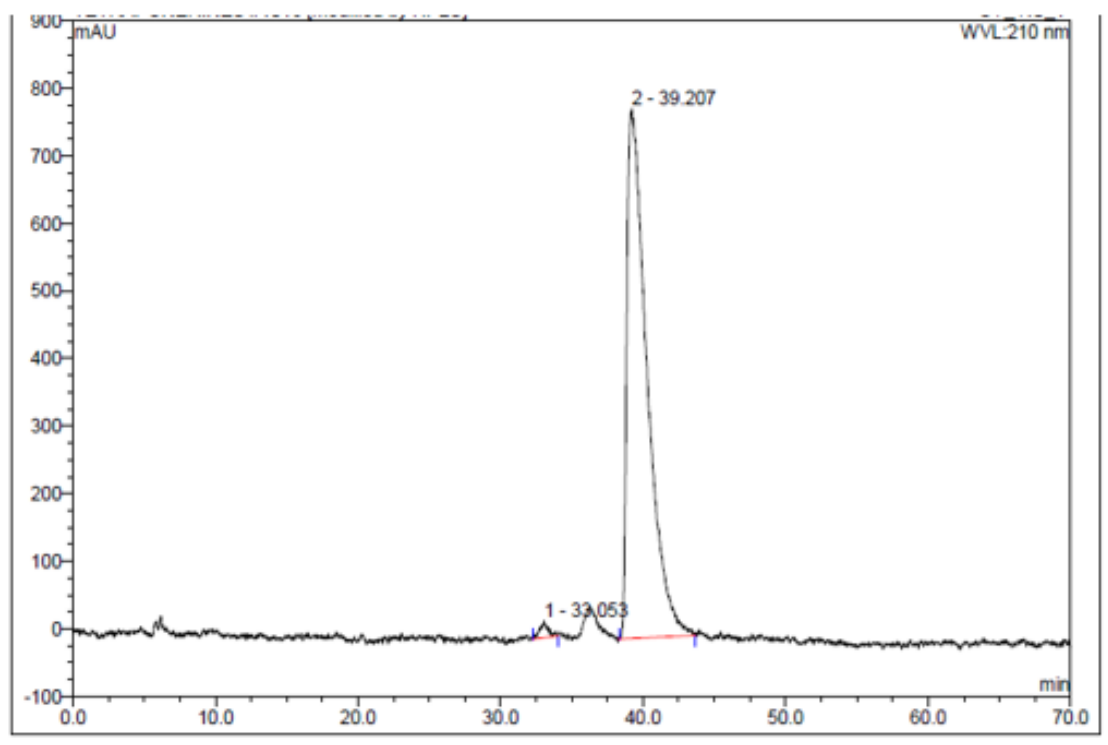

No.	Ret.Time min	Peak Name	Height mAU	Area mAU*min	Rel.Area %	Amount	Type
1	33.05	n.a.	25.119	16.227	1.25	n.a.	BMB*
2	39.21	n.a.	785.592	1282.873	98.75	n.a.	BMB*
Total:			810.711	1299.100	100.00	0.000	

^{1}H NMR (500 MHz, $CDCl_3$) of **3c**:

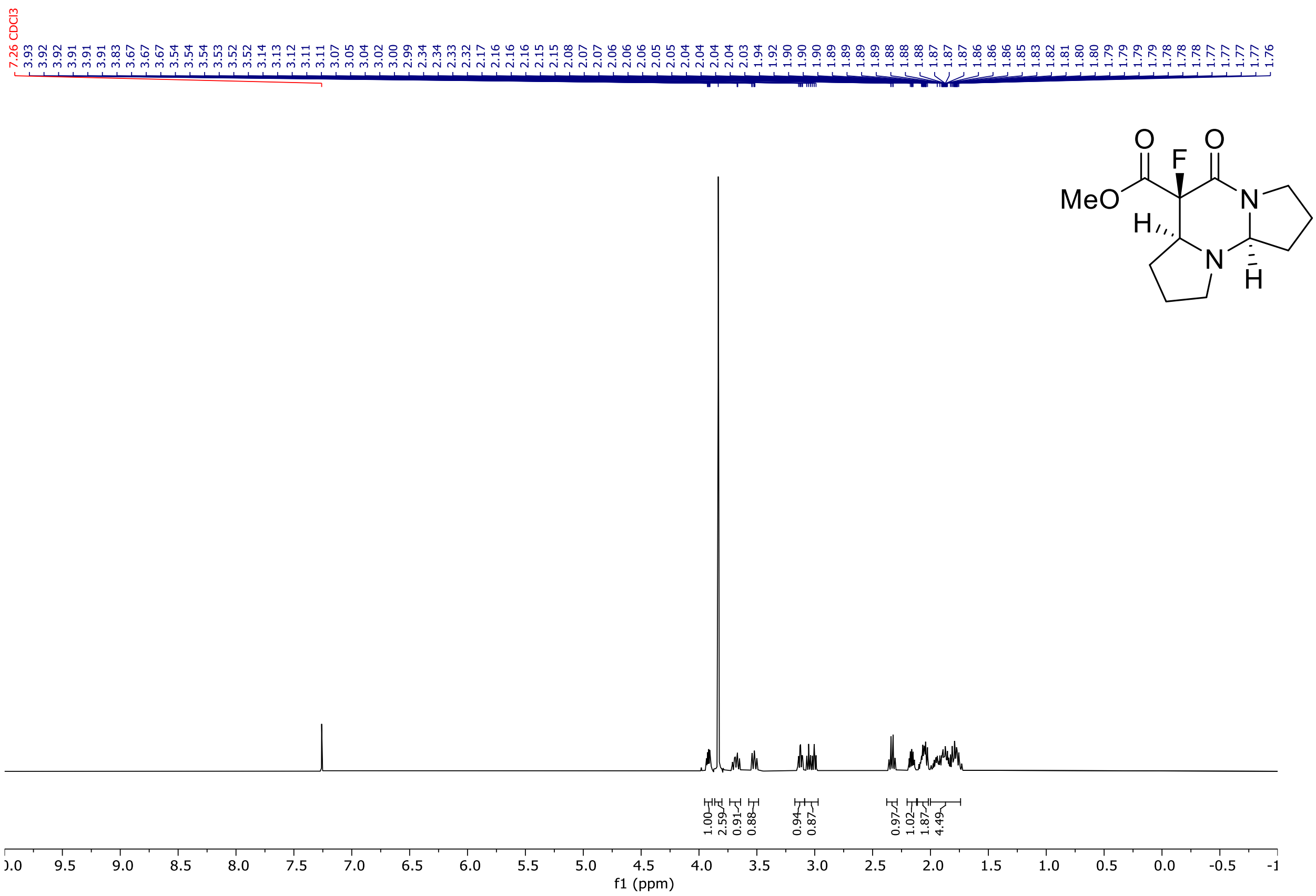

^{19}F NMR {^{1}H} (471 MHz, $CDCl_3$) of **3c**:

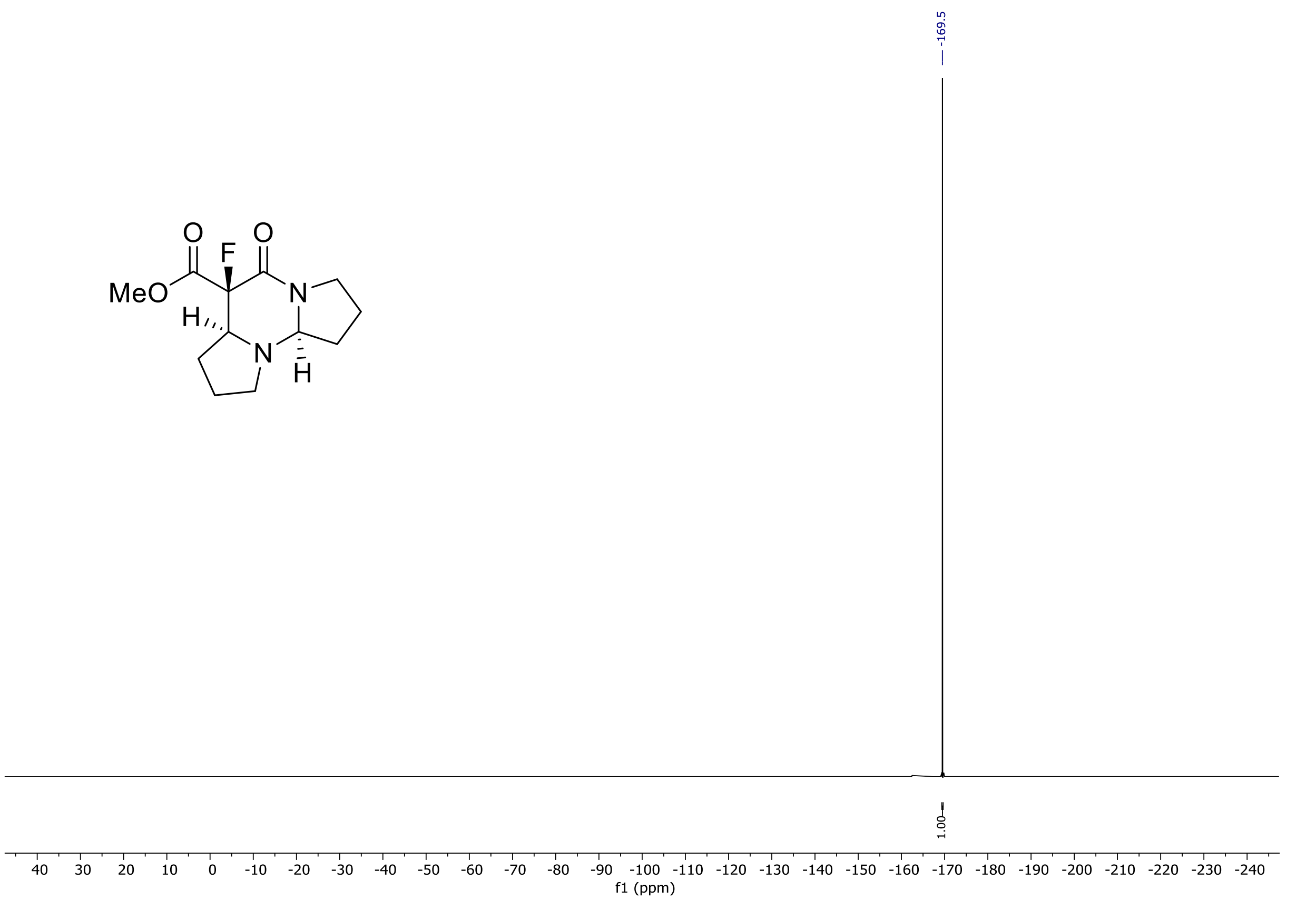

^{19}F NMR (471 MHz, $CDCl_3$) of **3c**:

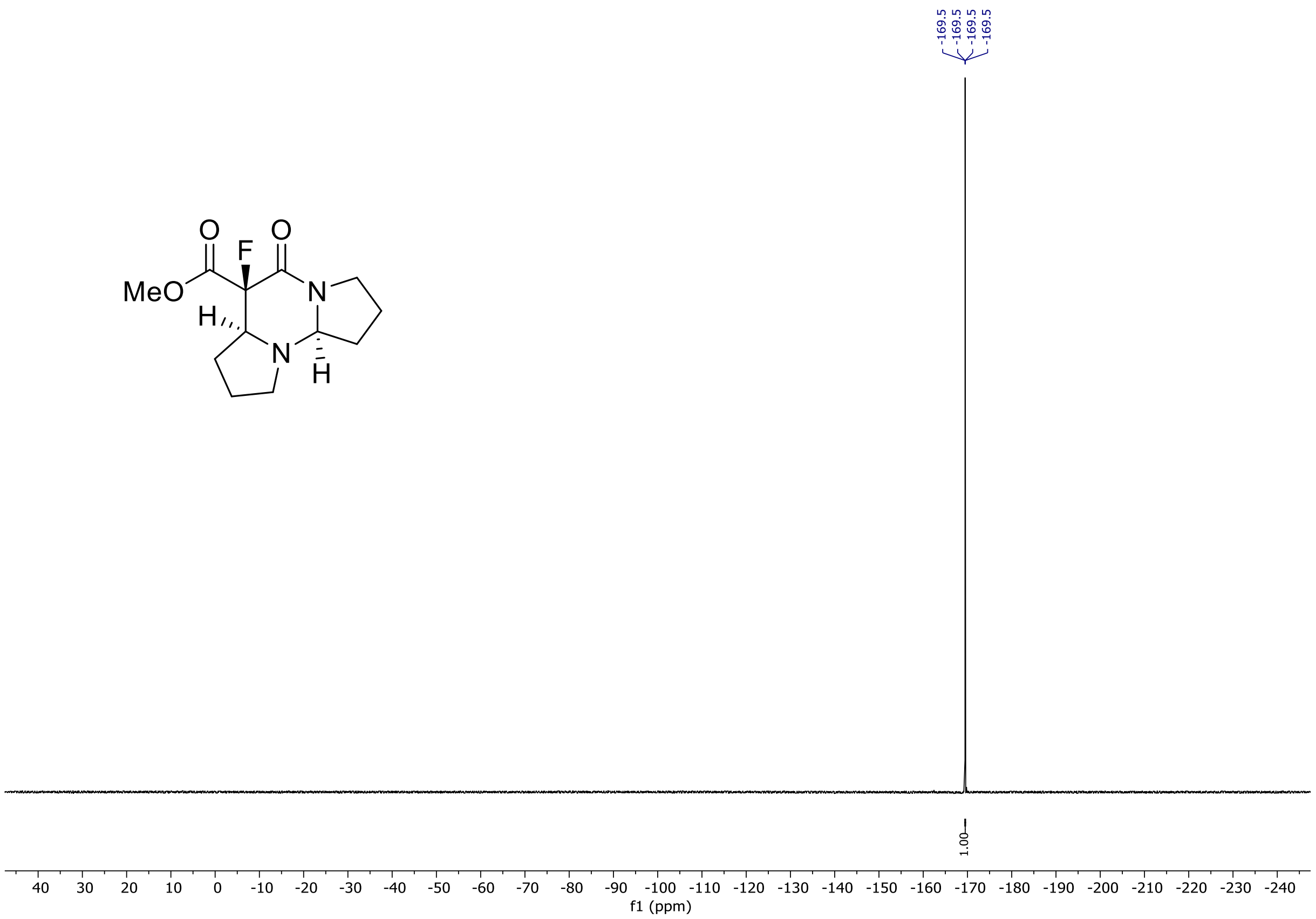

^{13}C NMR (125 MHz, $CDCl_3$) of **3c**:

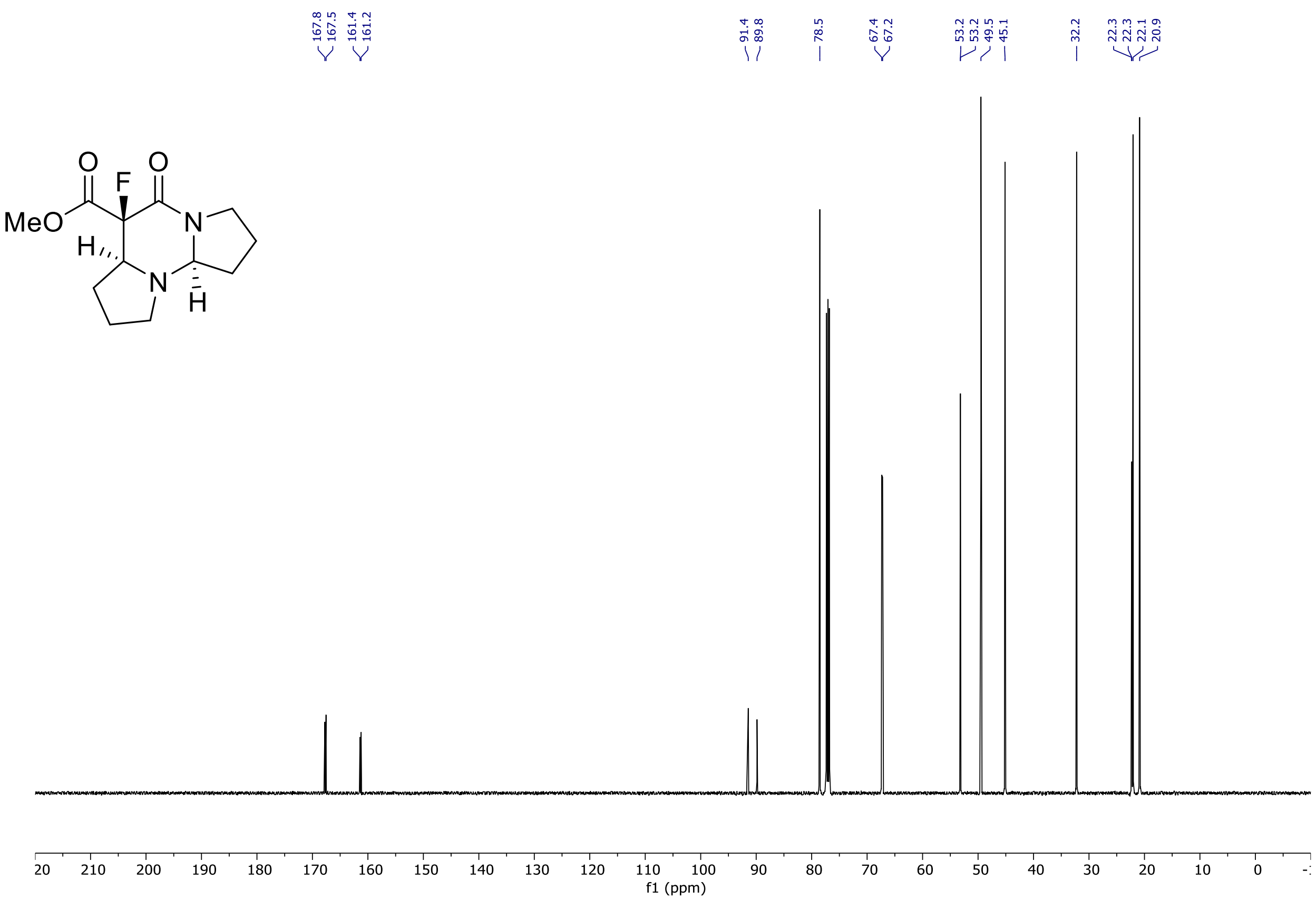

The enantiomeric access of **3c** was determined by chiral stationary phase HPLC: AD column, hexane/*i*-PrOH 85:15, 0.5 mL/min, 25 °C, 210 nm; 20.3 min (major), 21.5 min (minor).

racemate

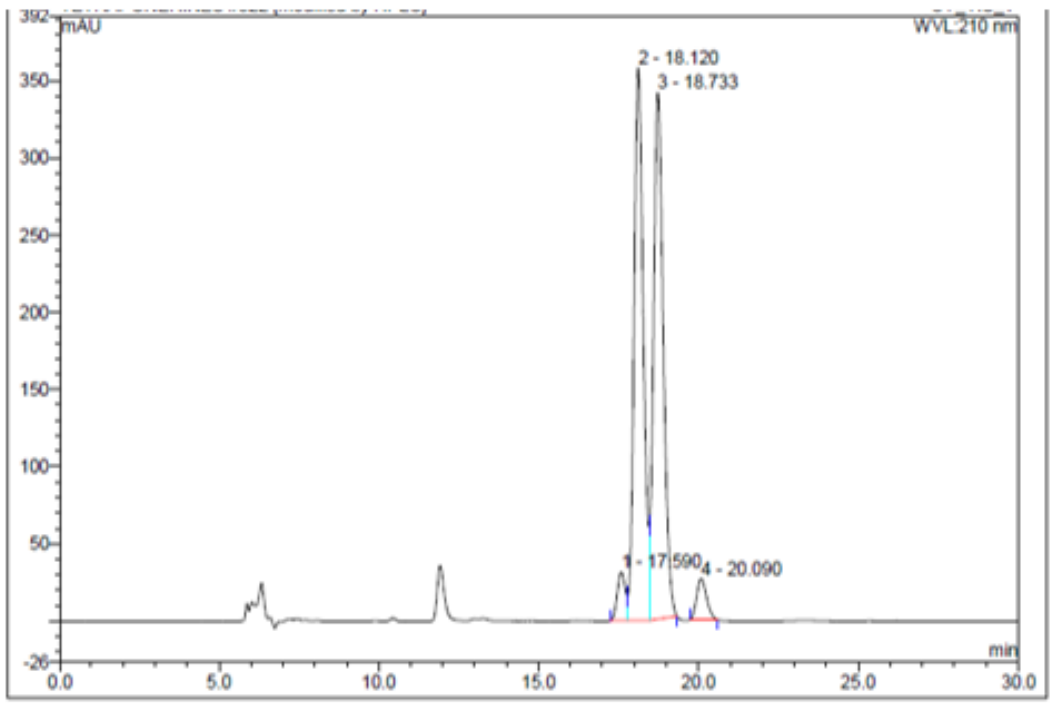

No.	Ret.Time min	Peak Name	Height mAU	Area mAU*min	Rel.Area %	Amount	Type
1	17.59	n.a.	31.424	9.100	3.50	n.a.	BM *
2	18.12	n.a.	357.996	119.842	46.06	n.a.	M *
3	18.73	n.a.	341.113	121.777	46.81	n.a.	MB*
4	20.09	n.a.	26.480	9.444	3.63	n.a.	BMB*
Total:			757.013	260.162	100.00	0.000	

Isolated product

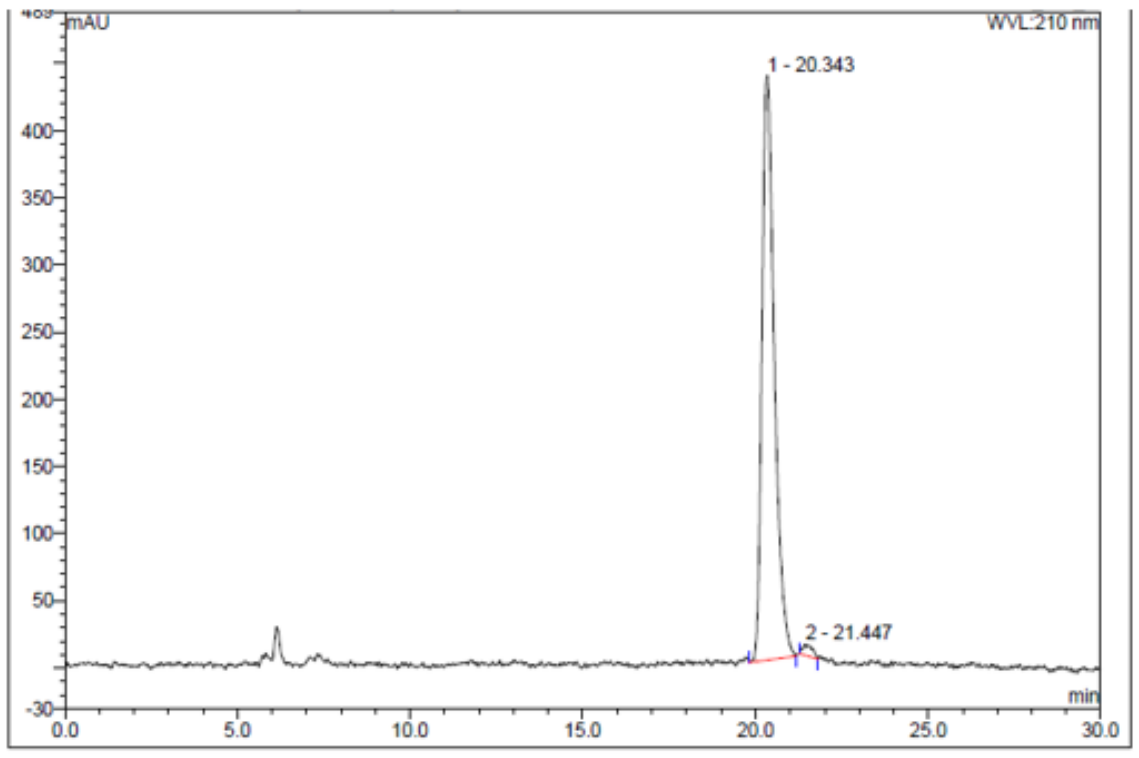

No.	Ret.Time min	Peak Name	Height mAU	Area mAU*min	Rel.Area %	Amount	Type
1	20.34	n.a.	434.865	181.031	98.44	n.a.	BMB*
2	21.45	n.a.	8.372	2.868	1.56	n.a.	BMB*
Total:			443.236	183.899	100.00	0.000	

^{1}H NMR (500 MHz, $CDCl_3$) of *ent*-**3c**:

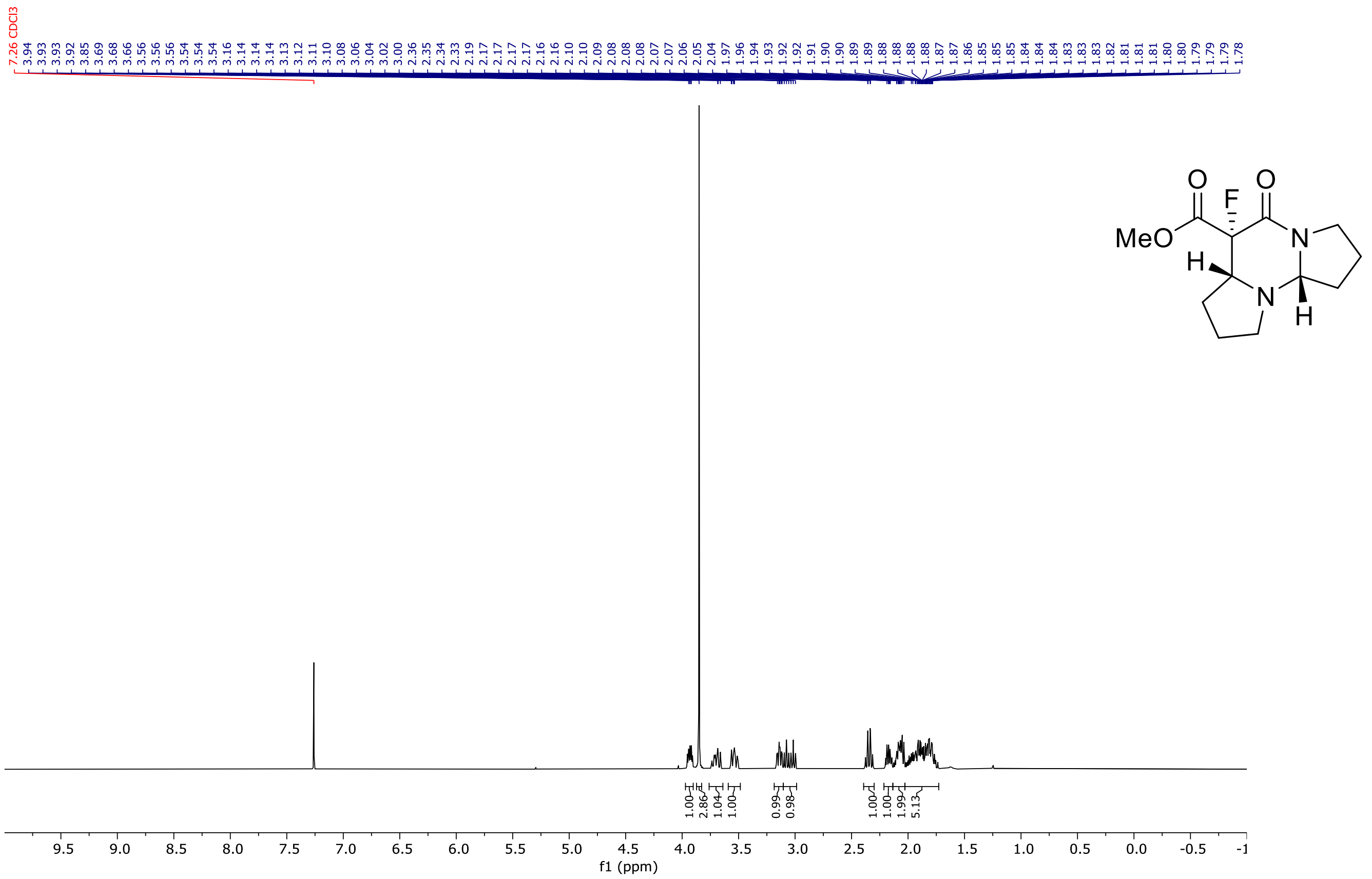

^{19}F NMR (471 MHz, $CDCl_3$) of *ent*-**3c**:

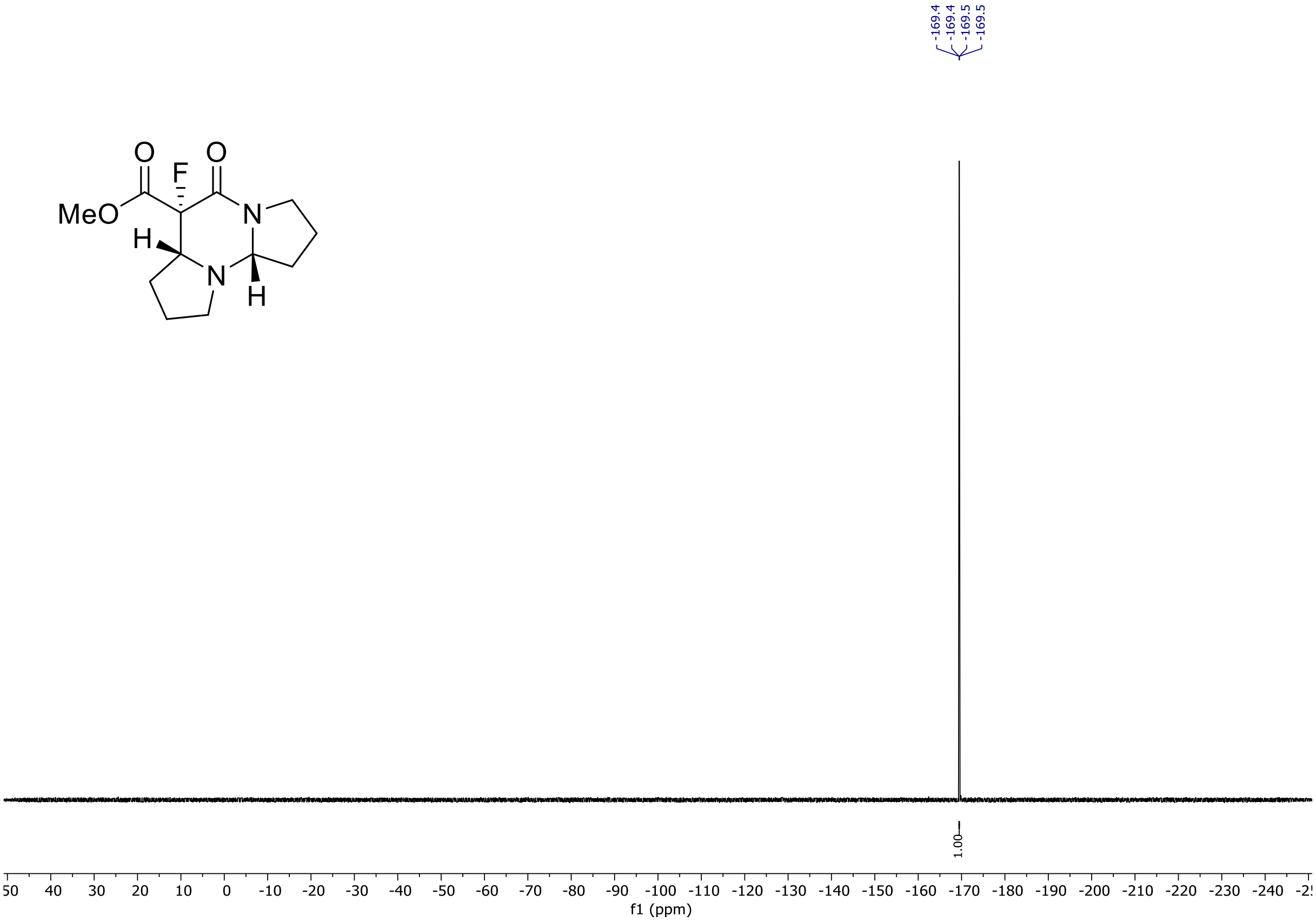

The enantiomeric access of *ent*-**3c** was determined by chiral stationary phase HPLC: AD column, hexane/*i*-PrOH 85:15, 0.5 mL/min, 25 °C, 210 nm; 20.8 min (minor), 21.7 min (major).

racemate

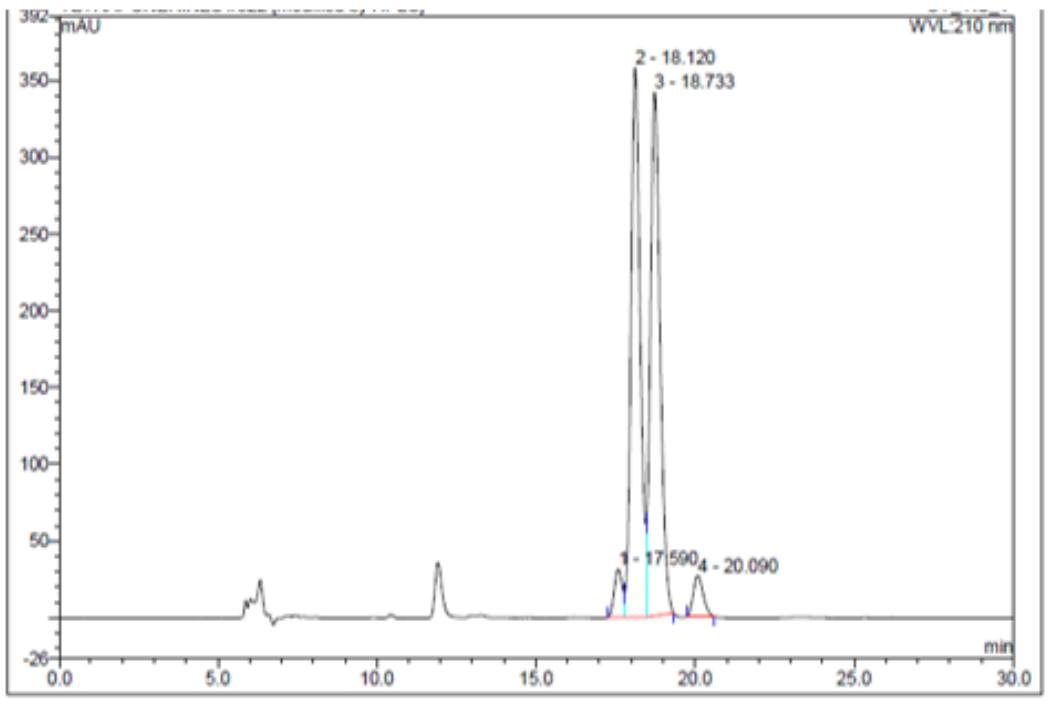

No.	Ret.Time min	Peak Name	Height mAU	Area mAU*min	Rel.Area %	Amount	Type
1	17.59	n.a.	31.424	9.100	3.50	n.a.	BM *
2	18.12	n.a.	357.996	119.842	46.06	n.a.	M *
3	18.73	n.a.	341.113	121.777	46.81	n.a.	MB*
4	20.09	n.a.	26.480	9.444	3.63	n.a.	BMB*
Total:			757.013	260.162	100.00	0.000	

Isolated product

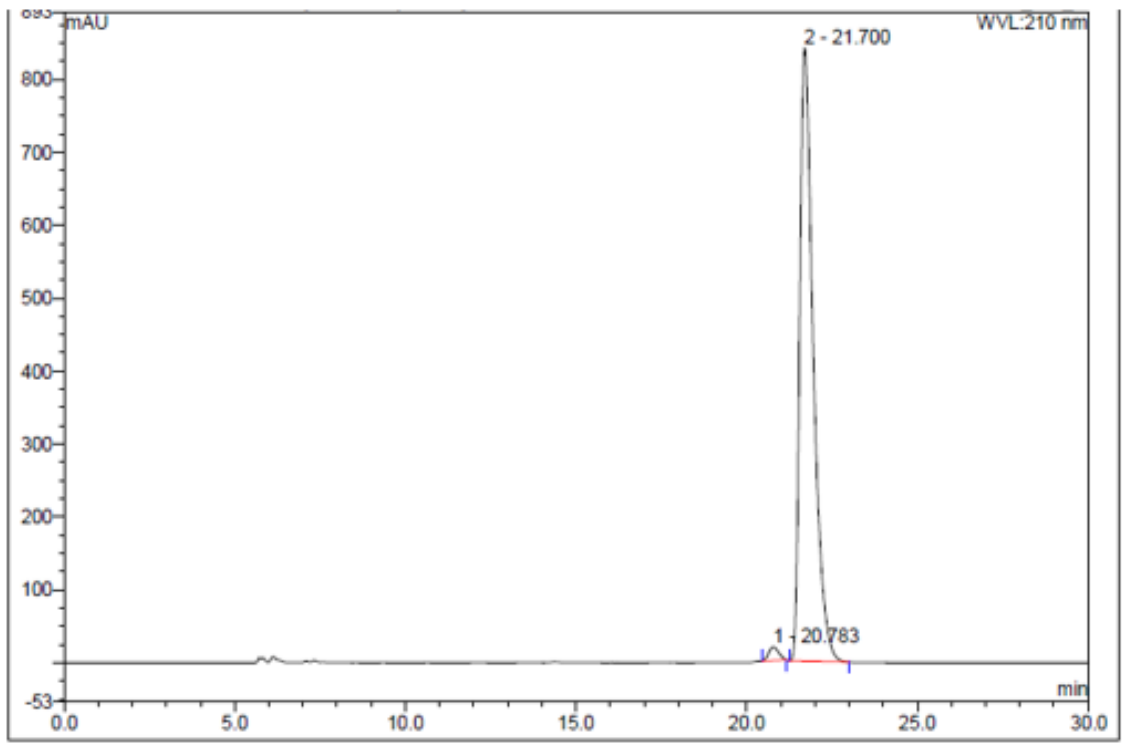

No.	Ret.Time min	Peak Name	Height mAU	Area mAU*min	Rel.Area %	Amount	Type
1	20.78	n.a.	18.294	6.174	1.58	n.a.	BMB*
2	21.70	n.a.	841.541	383.653	98.42	n.a.	BMB*
Total:			859.835	389.827	100.00	0.000	

^{1}**H NMR** (400 MHz, $CDCl_3$) of **3d**:

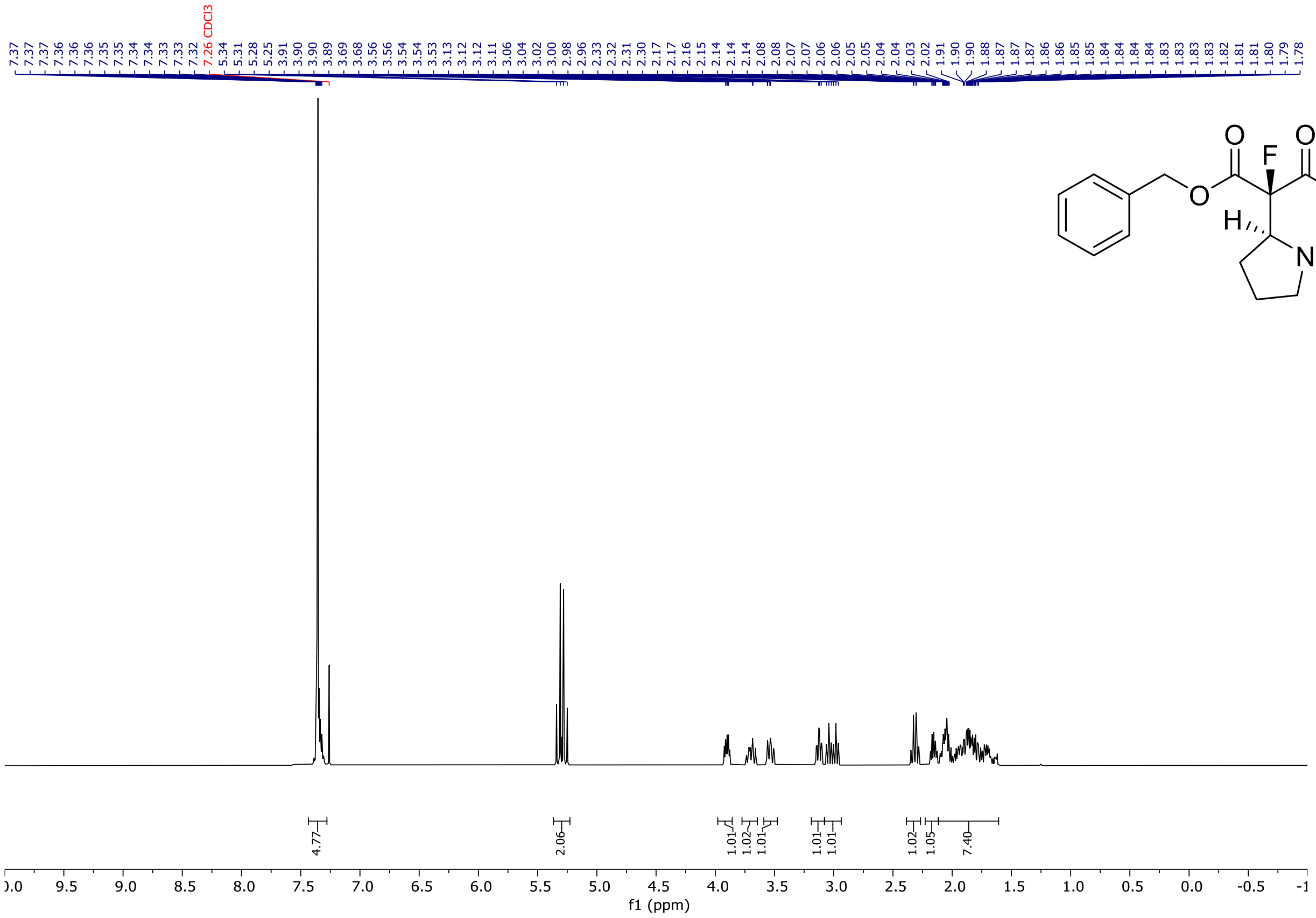

^{19}**F NMR** {^{1}H} (377 MHz, $CDCl_3$) of **3d**:

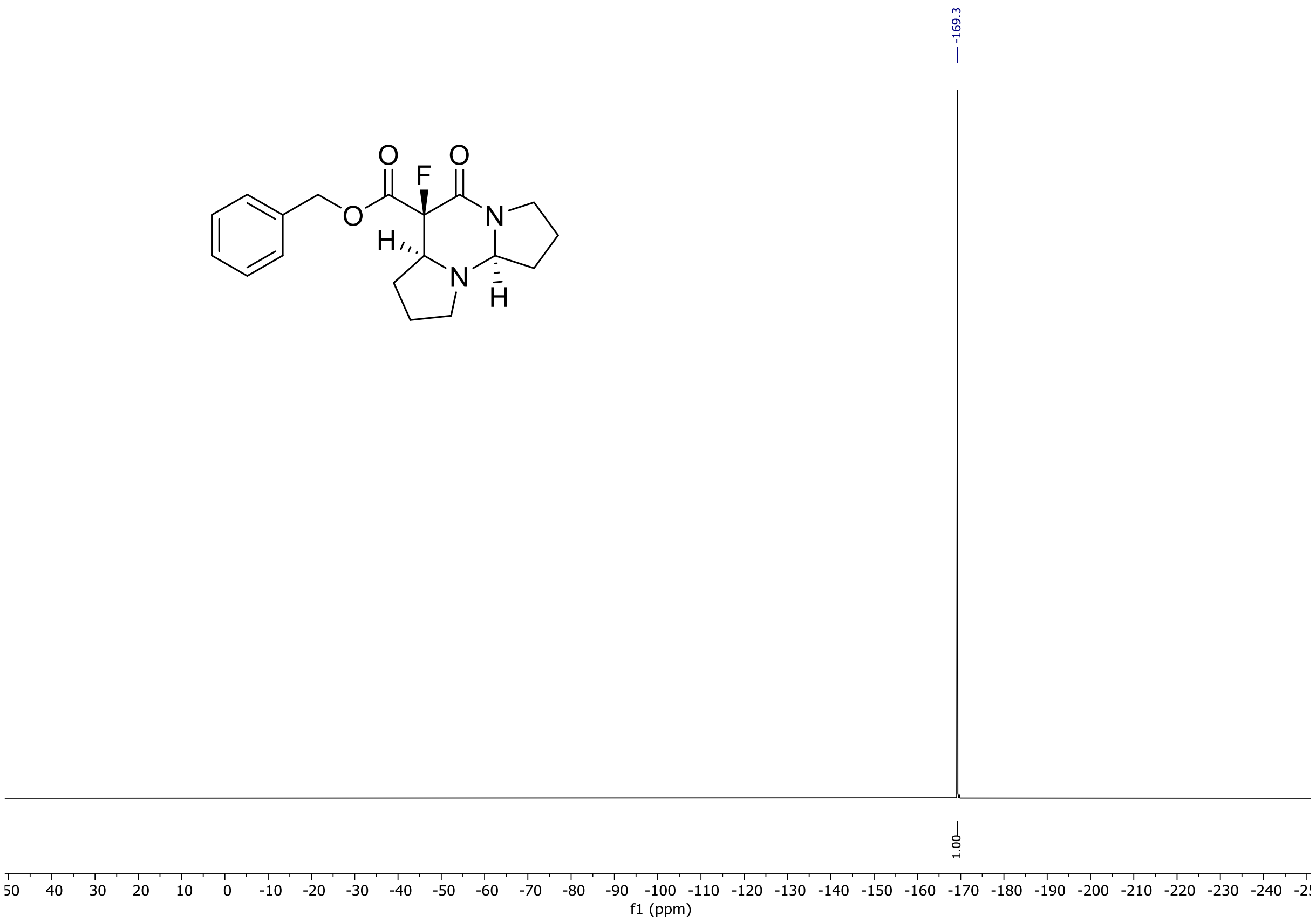

^{19}**F NMR** (377 MHz, $CDCl_3$) of **3d**:

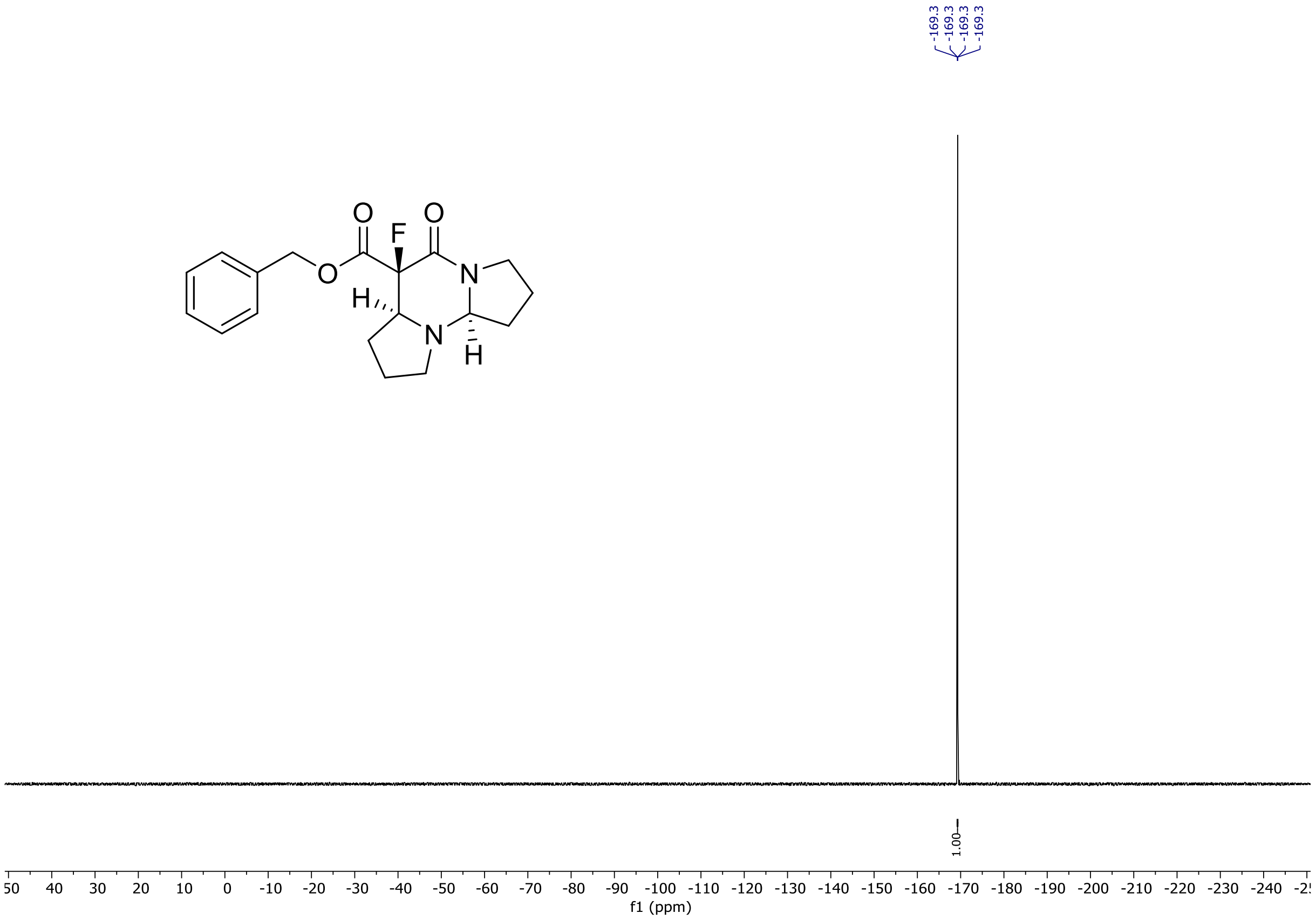

^{13}**C NMR** (101 MHz, $CDCl_3$) of **3d**:

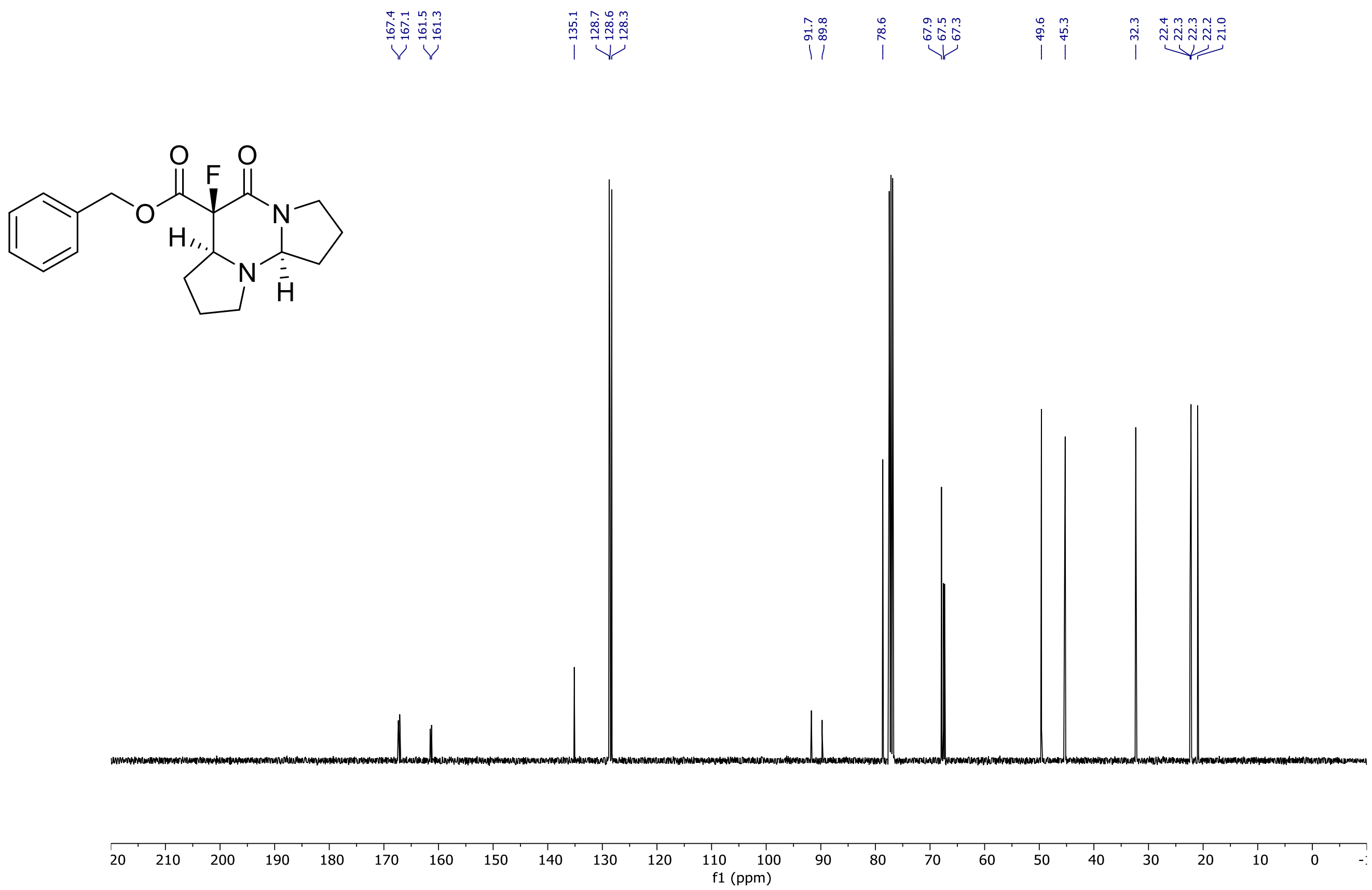

The enantiomeric access of **3d** was determined by chiral stationary phase HPLC: AD column, hexane/*i*-PrOH 80:20, 0.5 mL/min, 25 °C, 210 nm; 23.1 min (major), 28.8 min (minor).

racemate

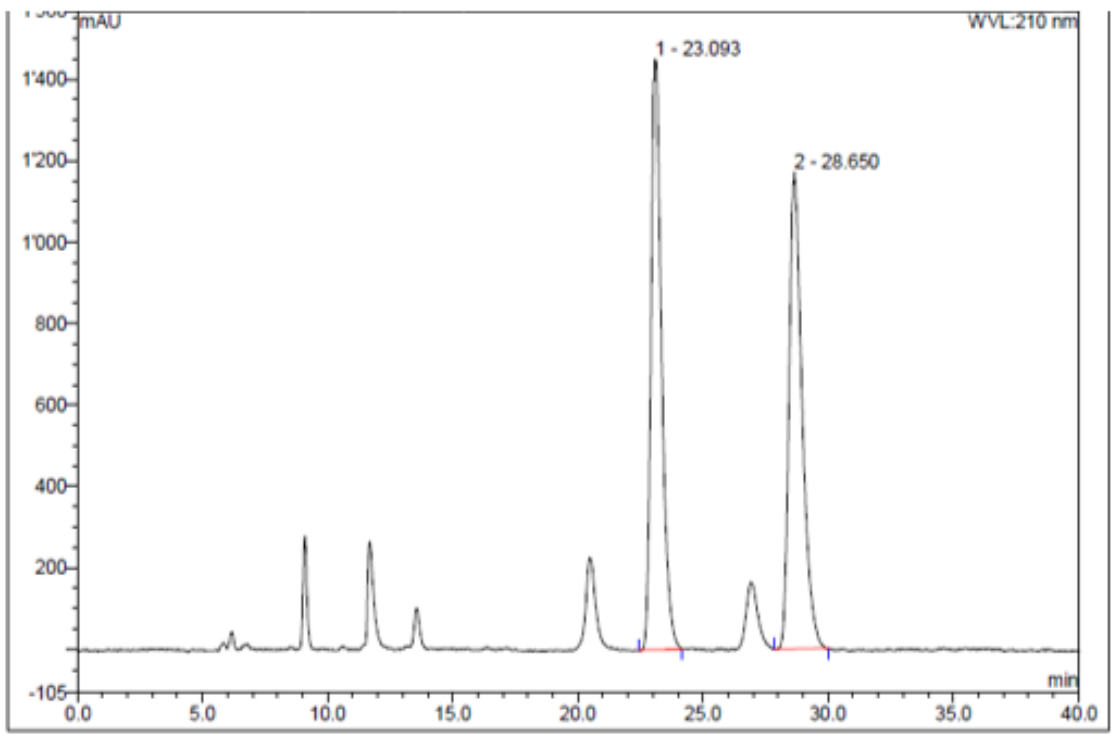

No.	Ret.Time min	Peak Name	Height mAU	Area mAU*min	Rel.Area %	Amount	Type
1	23.09	n.a.	1451.440	709.370	50.13	n.a.	BMB*
2	28.65	n.a.	1171.538	705.603	49.87	n.a.	BMB*
Total:			2622.978	1414.973	100.00	0.000	

Isolated product

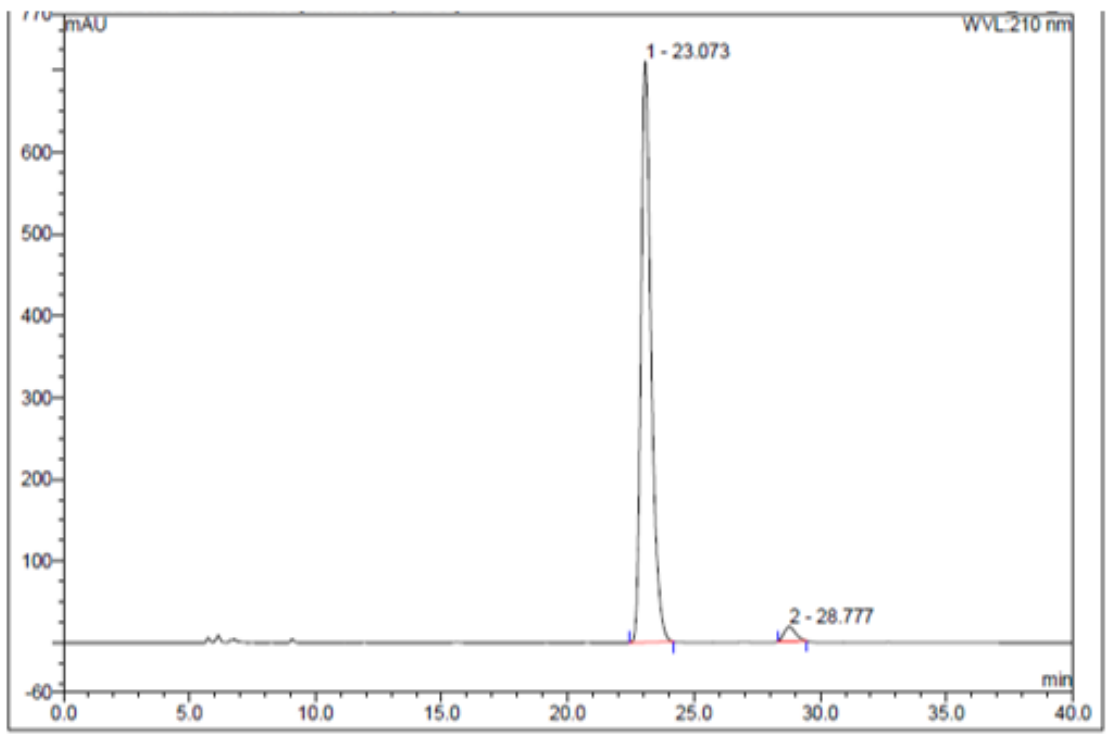

No.	Ret.Time min	Peak Name	Height mAU	Area mAU*min	Rel.Area %	Amount	Type
1	23.07	n.a.	710.477	332.523	97.23	n.a.	BMB*
2	28.78	n.a.	17.767	9.486	2.77	n.a.	BMB*
Total:			728.245	342.010	100.00	0.000	

^{1}**H NMR** (500 MHz, $CDCl_3$) of **3e**:

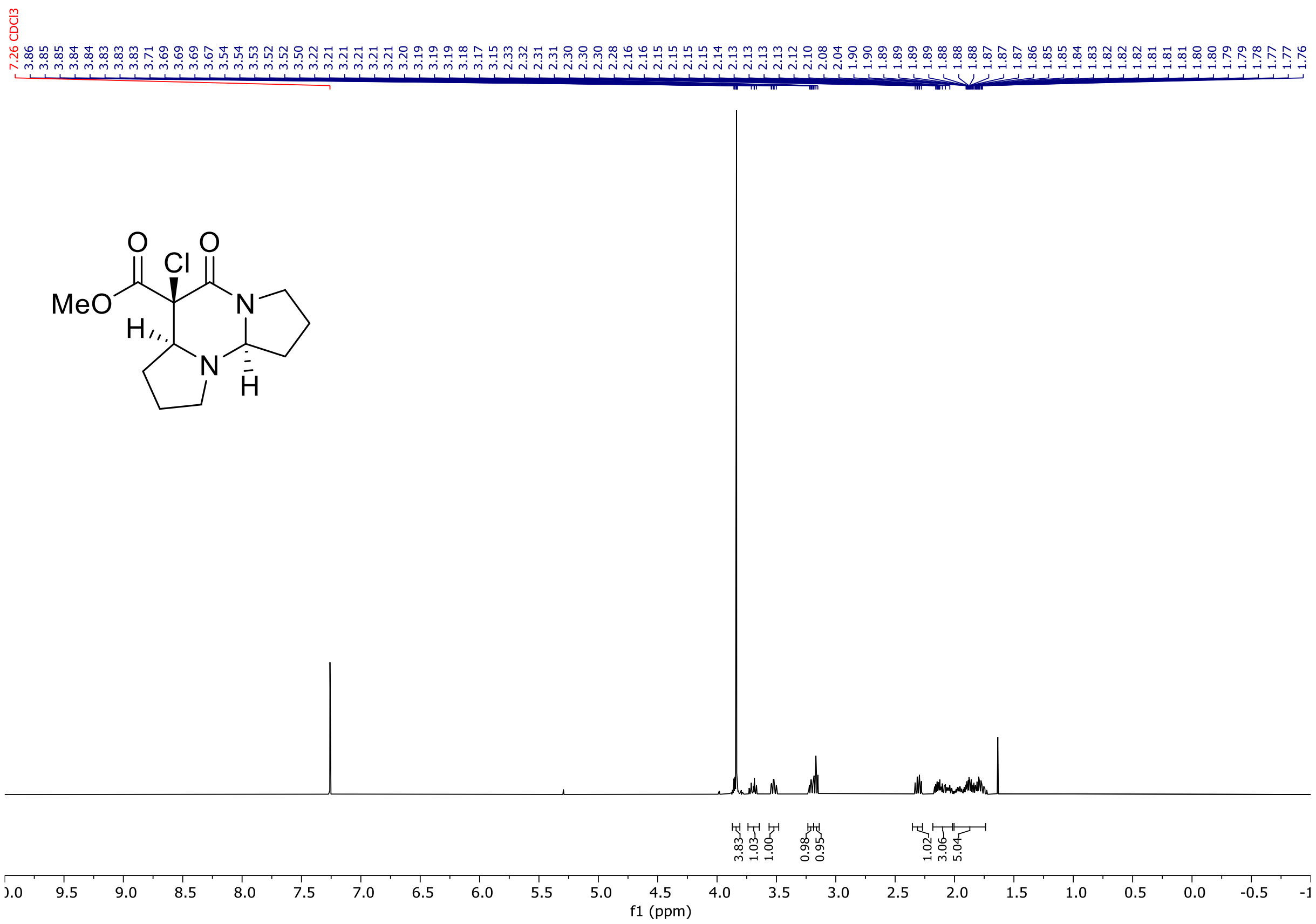

^{13}**C NMR** (126 MHz, $CDCl_3$) of **3e**:

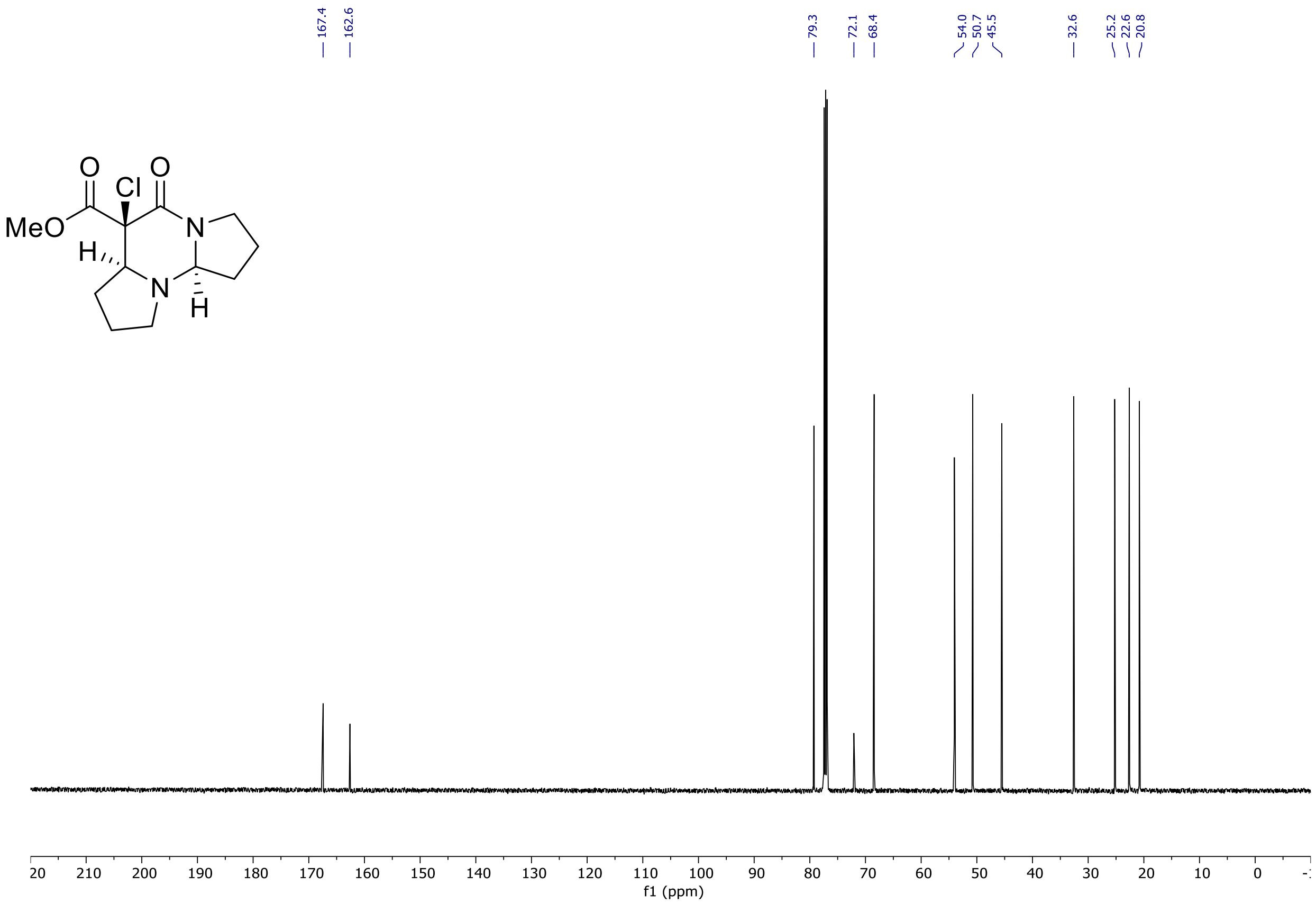

The enantiomeric access of **3e** was determined by chiral stationary phase HPLC: AD column, hexane/*i*-PrOH 80:20, 0.5 mL/min, 25 °C, 210 nm; 14.8 min (major), 16.0 min (minor).

racemate

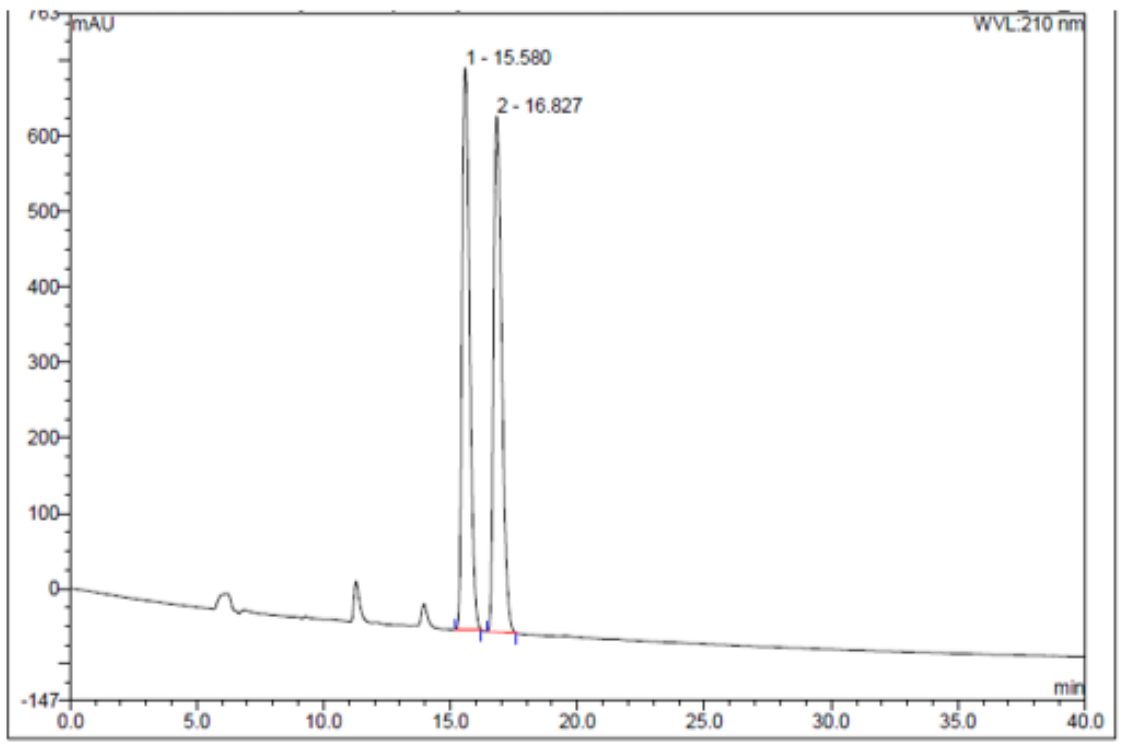

No.	Ret.Time min	Peak Name	Height mAU	Area mAU*min	Rel.Area %	Amount	Type
1	15.58	n.a.	745.674	257.166	50.09	n.a.	BMB*
2	16.83	n.a.	683.445	256.241	49.91	n.a.	BMB*
Total:			1429.119	513.407	100.00	0.000	

Isolated product

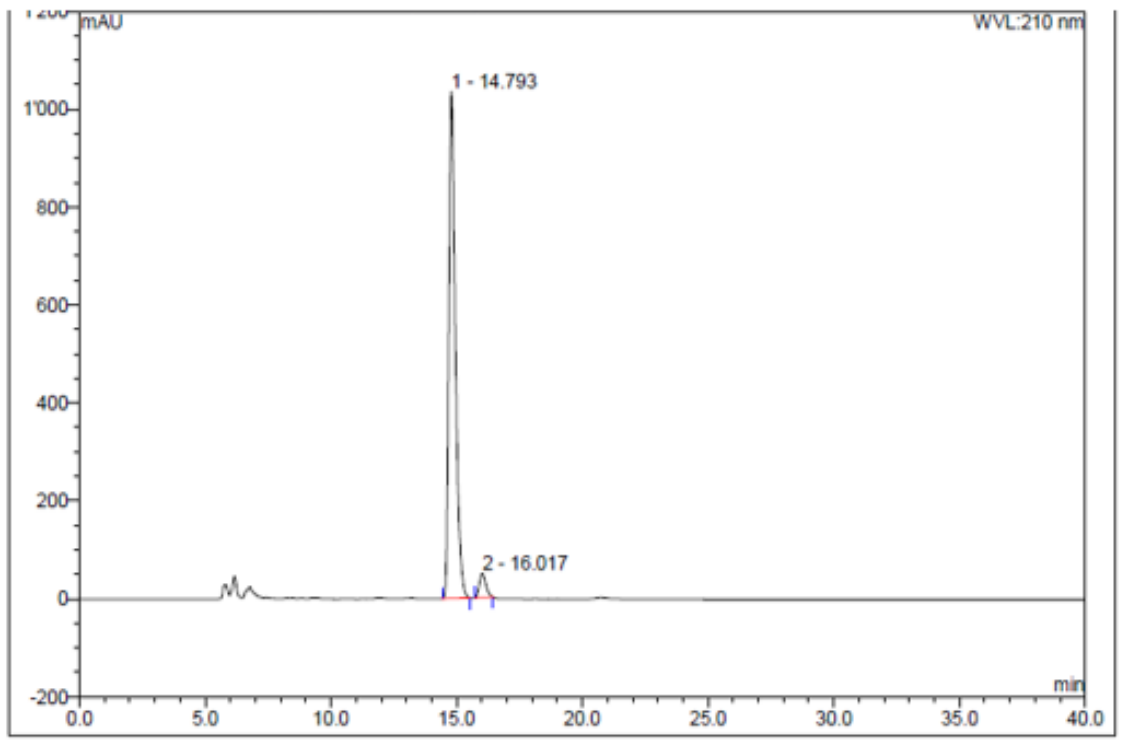

No.	Ret.Time min	Peak Name	Height mAU	Area mAU*min	Rel.Area %	Amount	Type
1	14.79	n.a.	1034.659	311.487	95.45	n.a.	BMB*
2	16.02	n.a.	49.430	14.849	4.55	n.a.	BMB*
Total:			1084.089	326.336	100.00	0.000	

^{1}**H NMR** (400 MHz, $CDCl_3$) of **3f**:

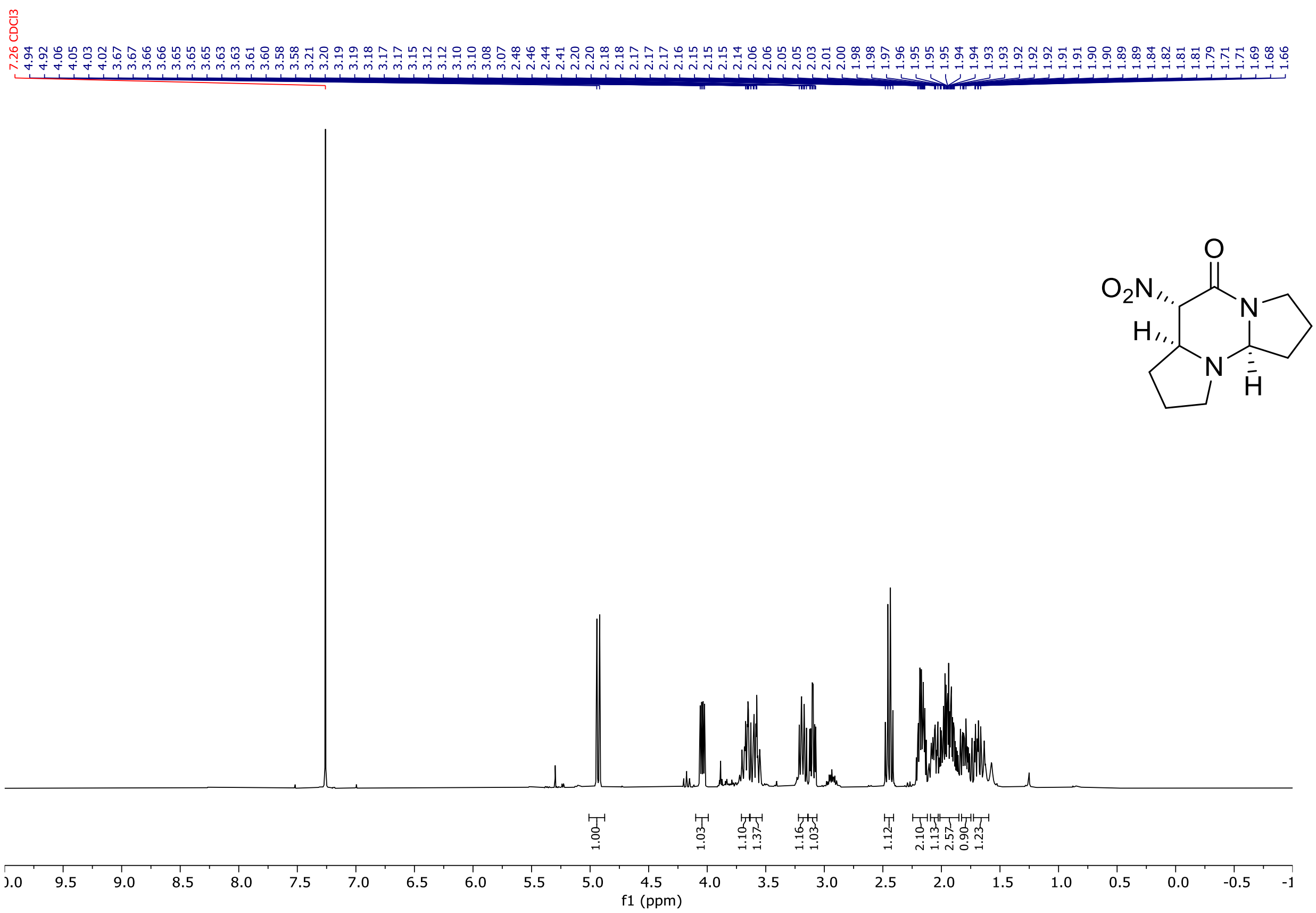

^{13}**C NMR** (101 MHz, $CDCl_3$) of **3f**:

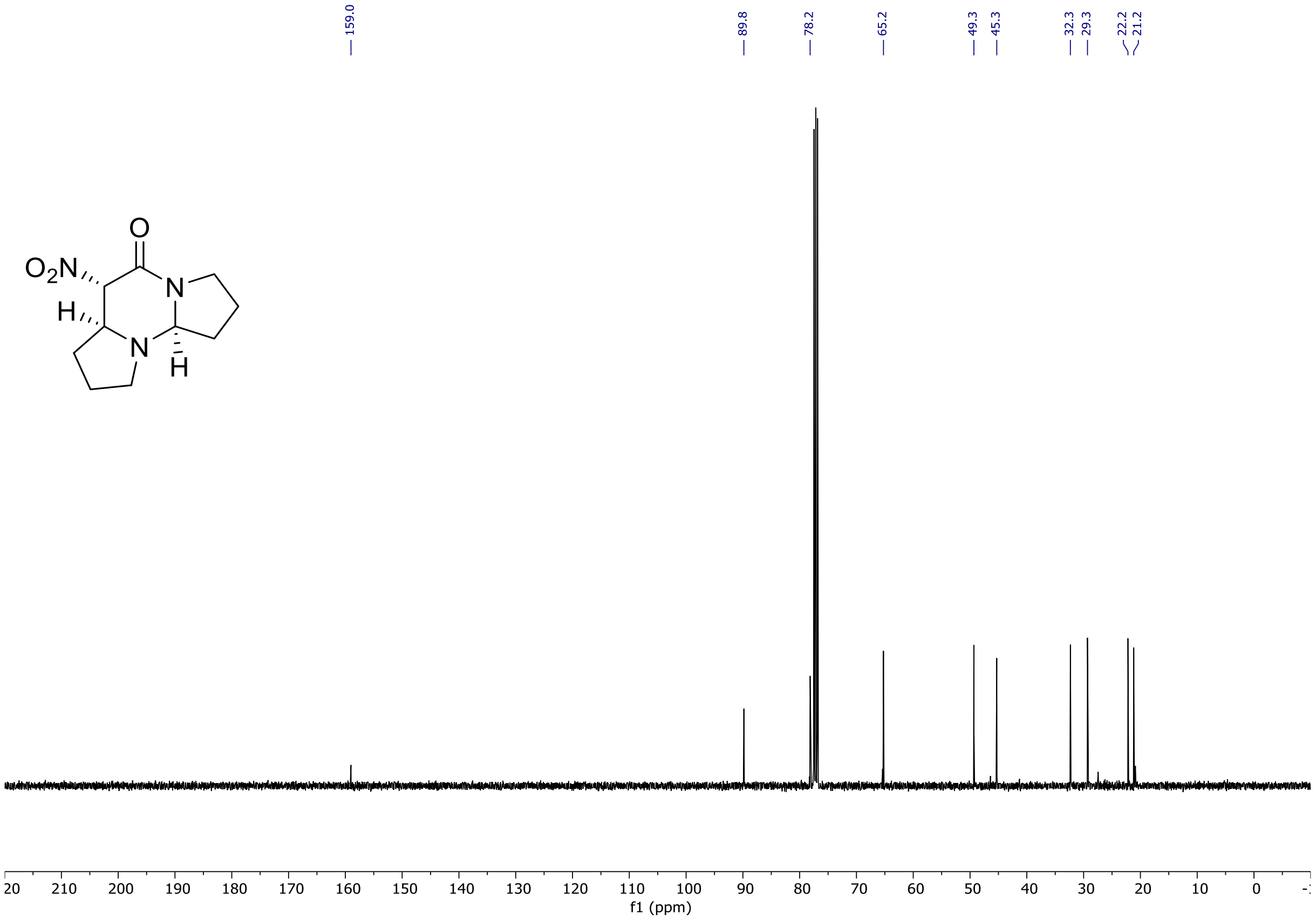

The enantiomeric access of **3f** was determined by chiral stationary phase SFC: AS3 column, CO_2/MeOH 90:10, 2.0 mL/min, 214 nm; 1.4 min (major), 1.7 min (minor).

racemate

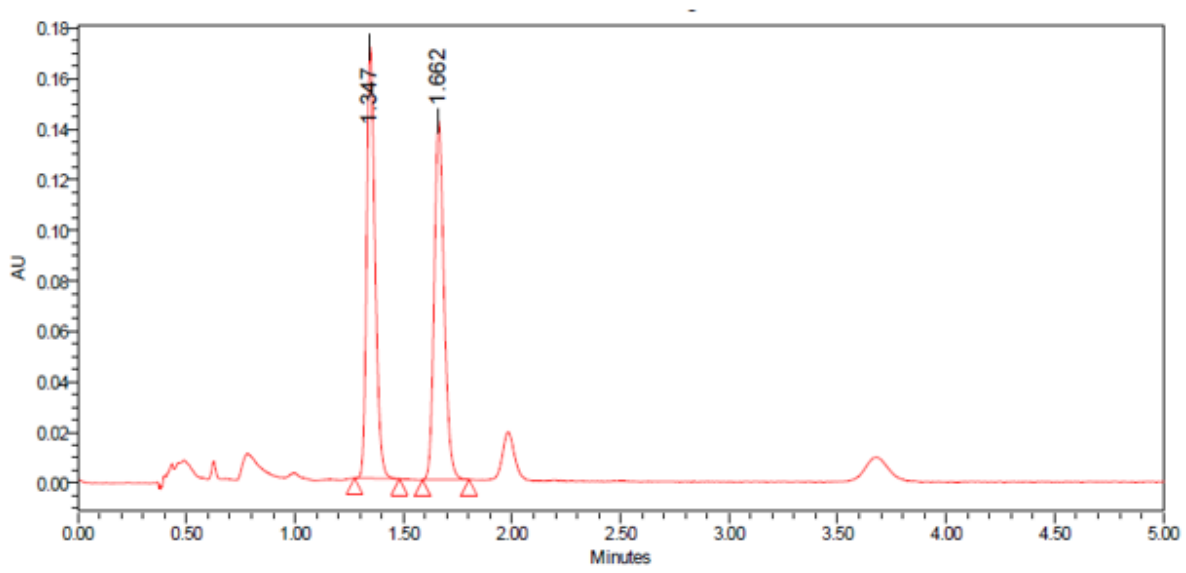

Peak Results

	Name	RT	Area	Height	% Area	Start Time (min)	End Time (min)
1		1.347	458160	170688	50.36	1.275	1.482
2		1.662	451696	142100	49.64	1.588	1.803

Isolated product

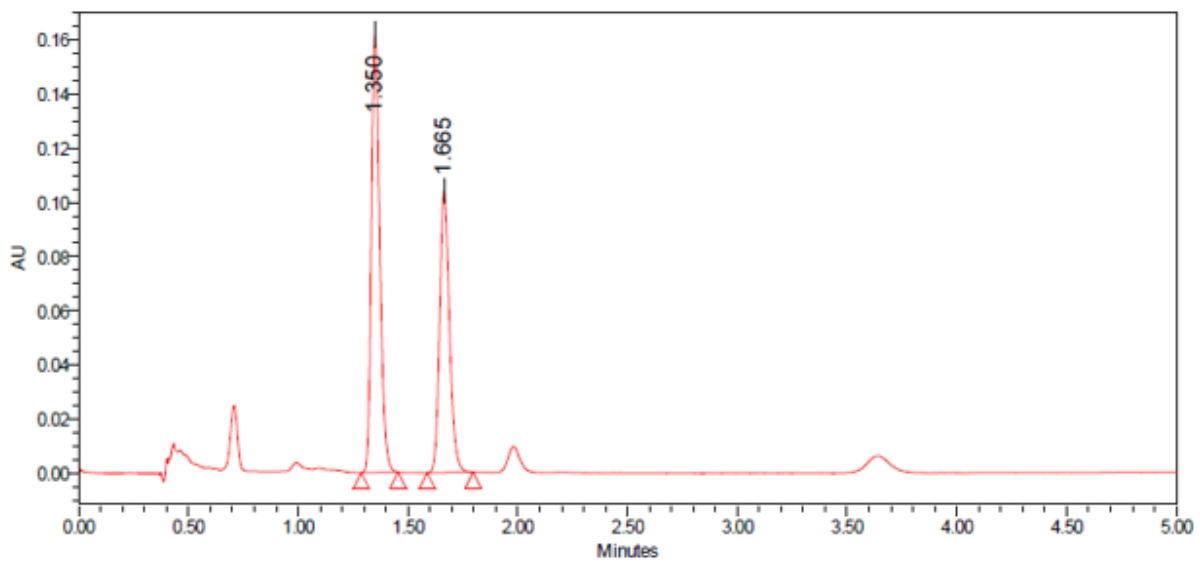

Peak Results

	Name	RT	Area	Height	% Area	Start Time (min)	End Time (min)
1		1.350	429634	161849	57.04	1.288	1.455
2		1.665	323551	104170	42.96	1.588	1.798

¹H NMR (400 MHz, $CDCl_3$) of **3g**:

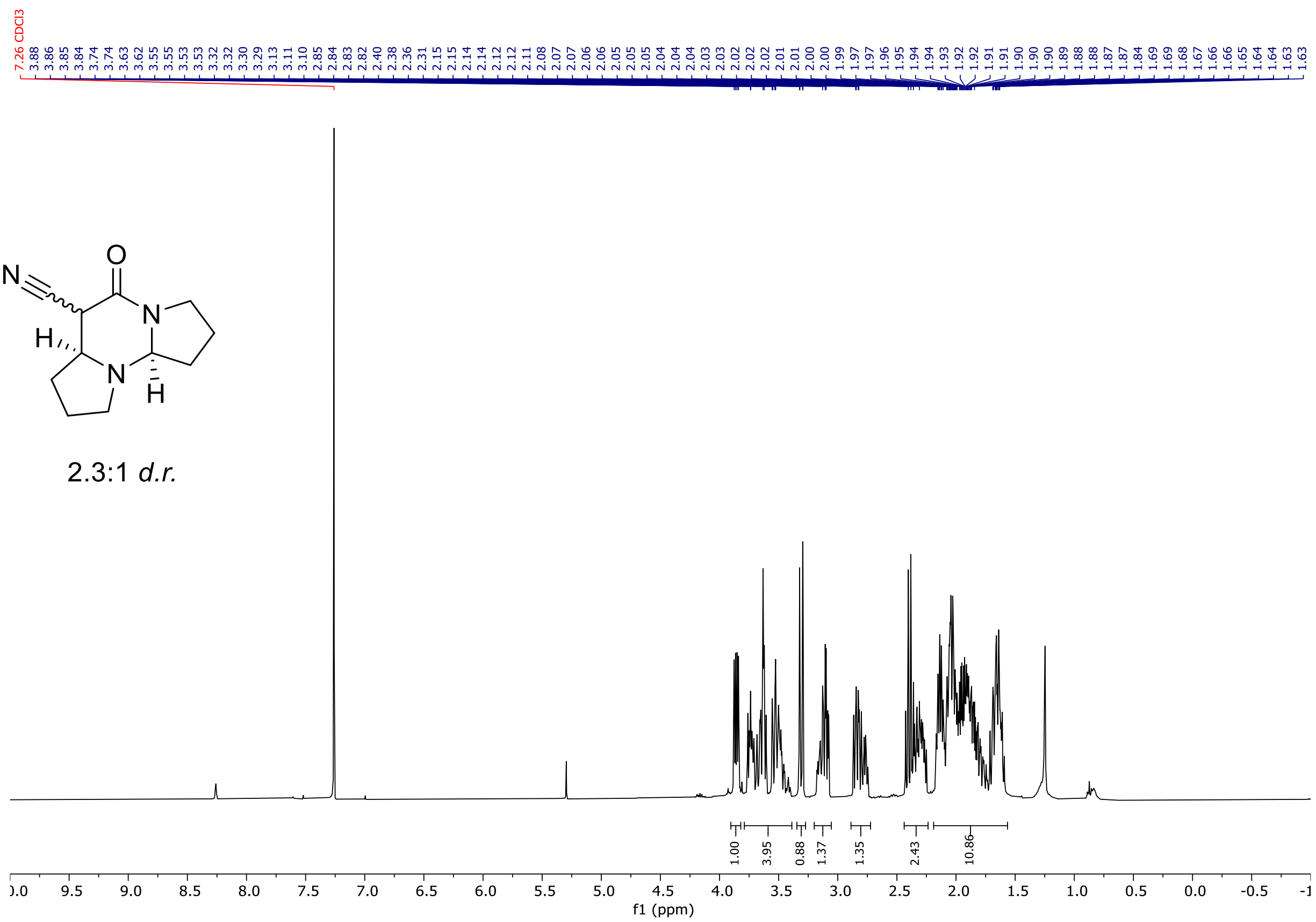

¹³C NMR (101 MHz, $CDCl_3$) of **3g**:

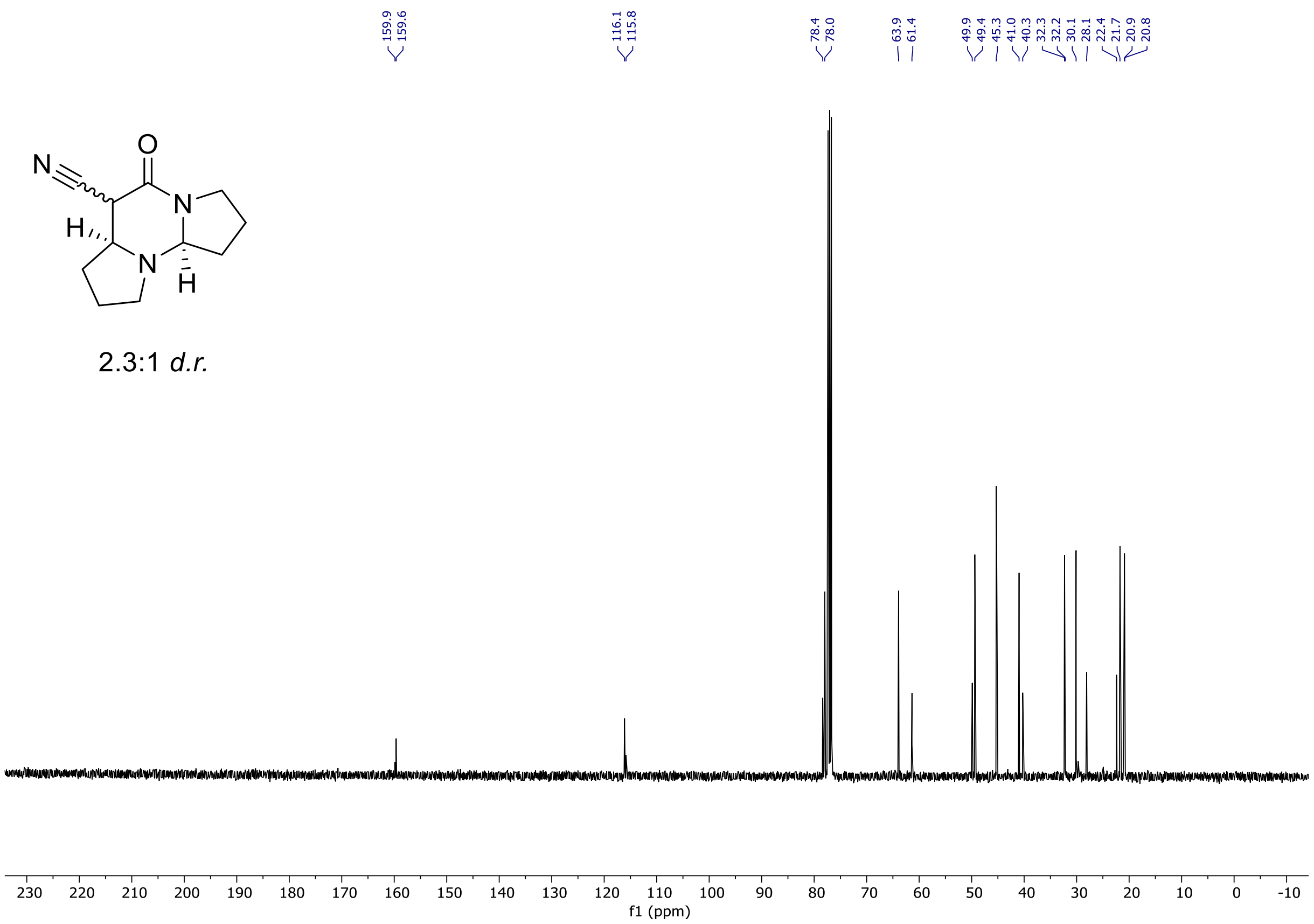

The enantiomeric access of **3g** was determined by chiral stationary phase SFC: AS3 column, CO_2/MeOH 90:10, 2.0 mL/min, 214 nm; major diastereoisomer: 1.1 min (major), 1.8 min (minor); minor diastereoisomer: 1.5 min (minor), 2.9 min (major).

racemate

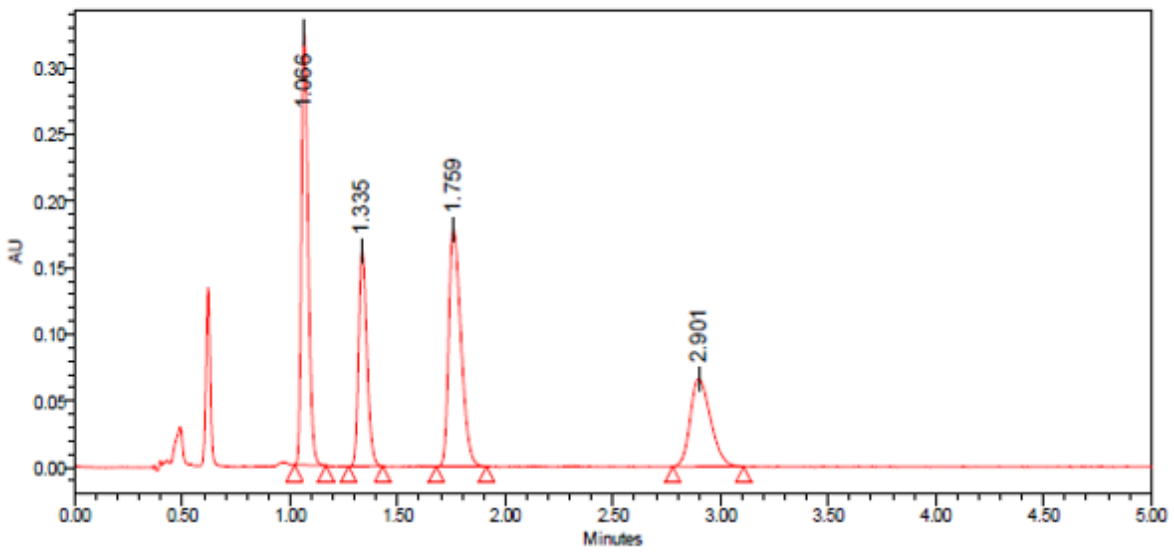

Peak Results

	Name	RT	Area	Height	% Area	Start Time (min)	End Time (min)
1		1.066	705291	324847	30.97	1.022	1.168
2		1.335	433755	161987	19.05	1.273	1.430
3		1.759	704699	178300	30.95	1.680	1.913
4		2.901	433375	65958	19.03	2.778	3.107

Isolated product

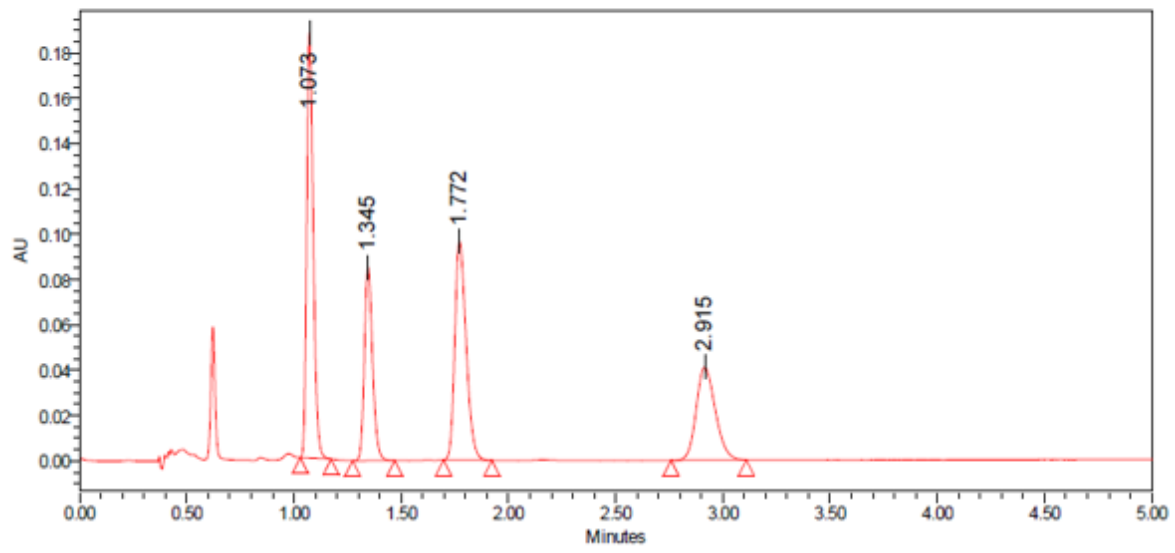

Peak Results

	Name	RT	Area	Height	% Area	Start Time (min)	End Time (min)
1		1.073	398517	187807	32.36	1.029	1.173
2		1.345	225596	85379	18.32	1.273	1.470
3		1.772	353514	96504	28.70	1.698	1.923
4		2.915	253917	41037	20.62	2.758	3.110

^{1}H NMR (500 MHz, $CDCl_3$) of **3h**:

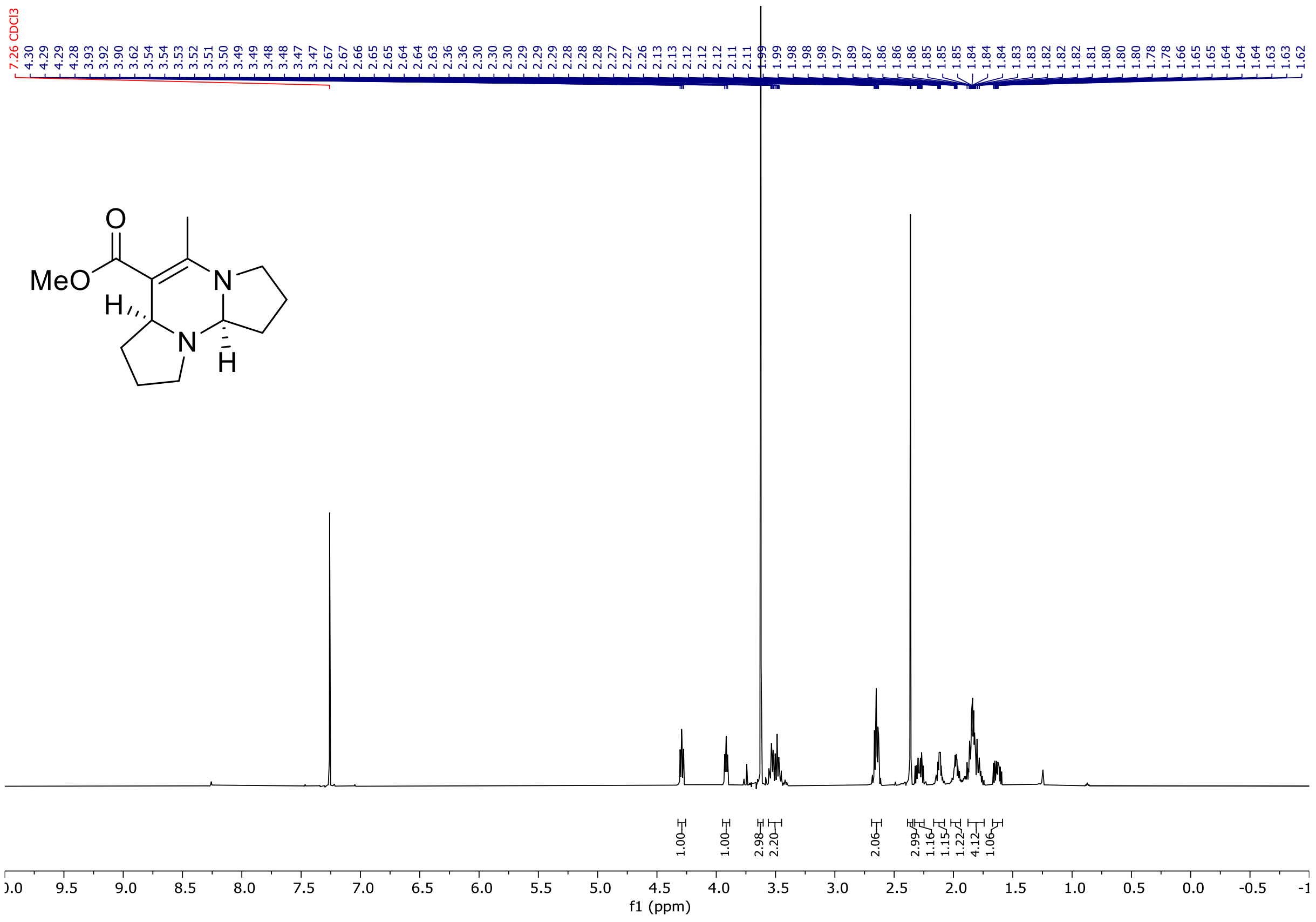

^{13}C NMR (101 MHz, $CDCl_3$) of **3h**:

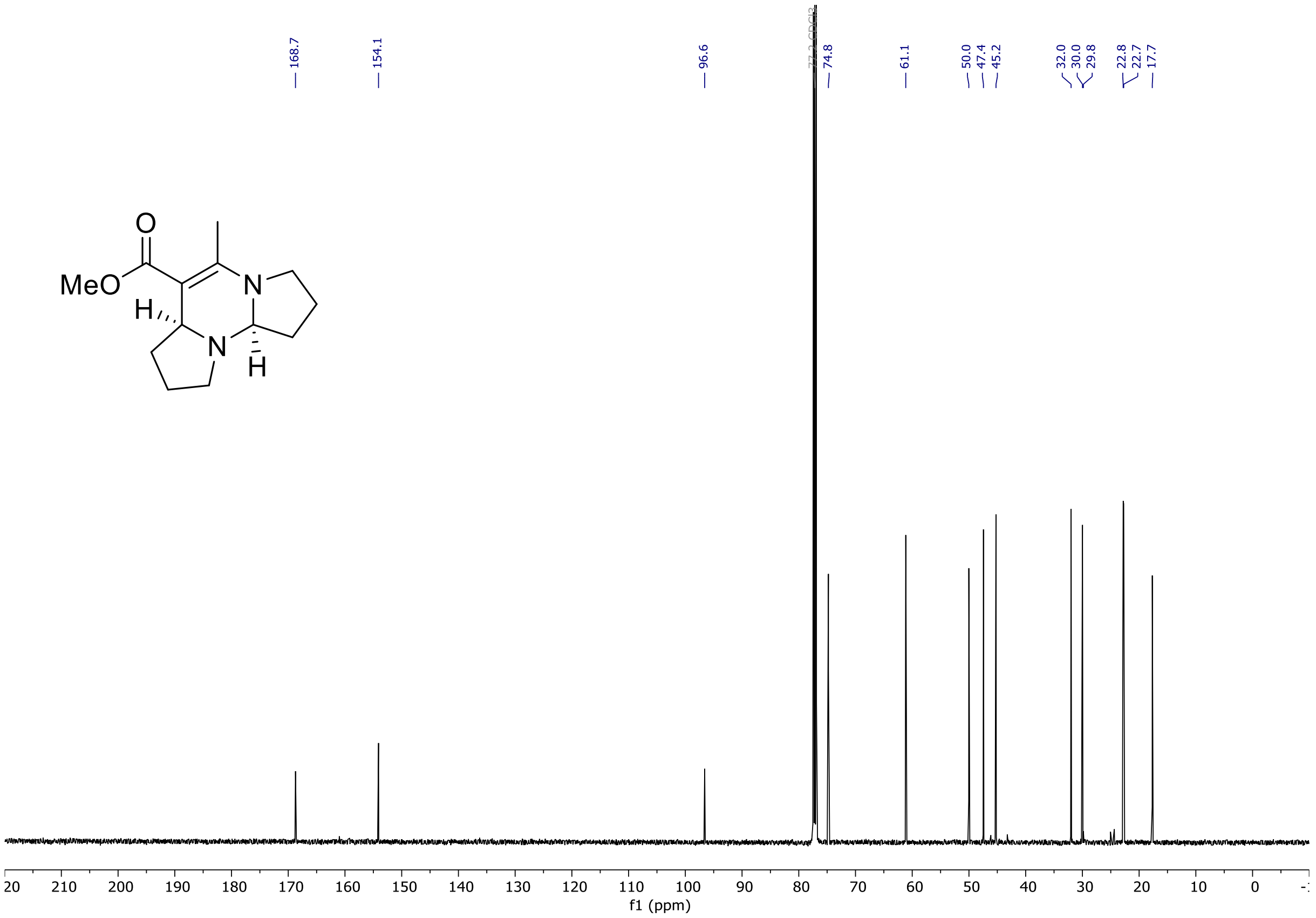

The enantiomeric access of **3h** was determined by chiral stationary phase SFC: Trefoil column, CO_2/MeOH 85:15, 2.0 mL/min, 214 nm; 1.4 min (minor), 2.4 min (major).

racemate

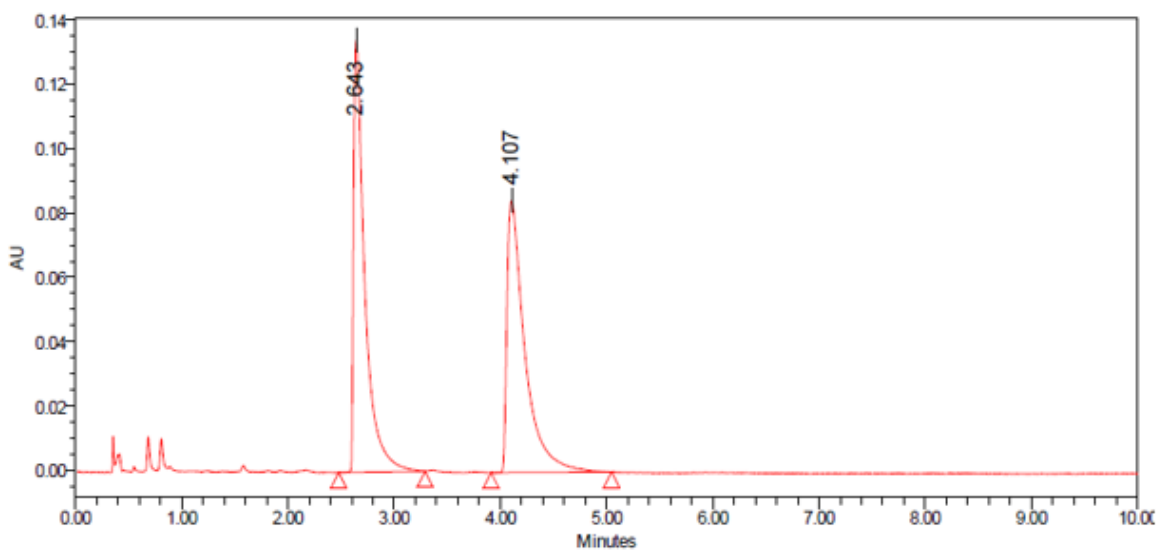

Peak Results

	Name	RT	Area	Height	% Area	Start Time (min)	End Time (min)
1		2.643	982666	134247	49.92	2.478	3.291
2		4.107	985772	84445	50.08	3.913	5.048

Isolated product

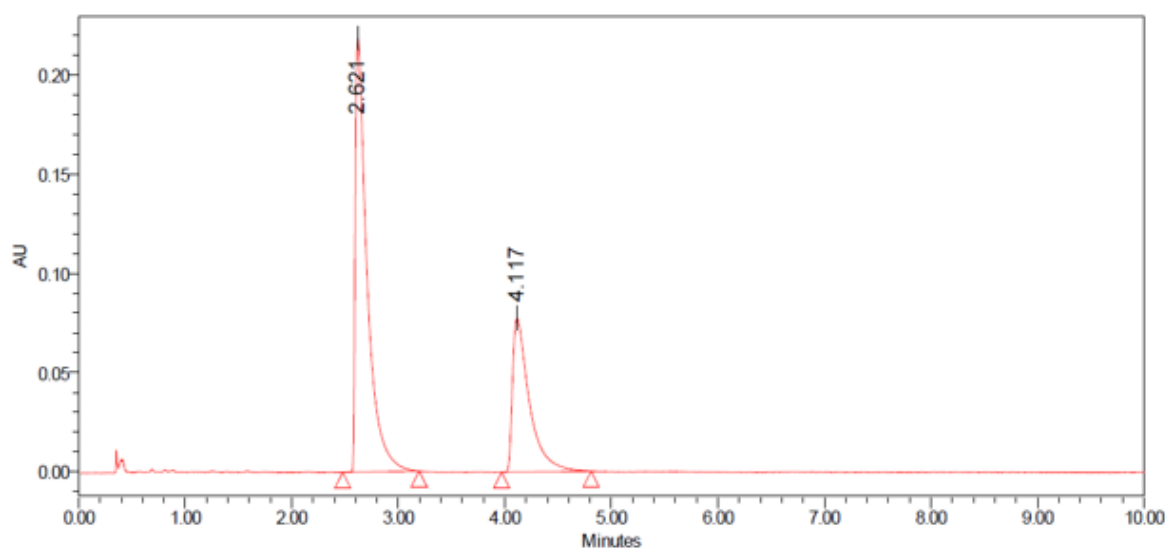

Peak Results

	Name	RT	Area	Height	% Area	Start Time (min)	End Time (min)
1		2.621	1679145	218617	66.37	2.478	3.196
2		4.117	850906	77491	33.63	3.970	4.810

^{1}H NMR (400 MHz, $CDCl_3$) of **3i**:

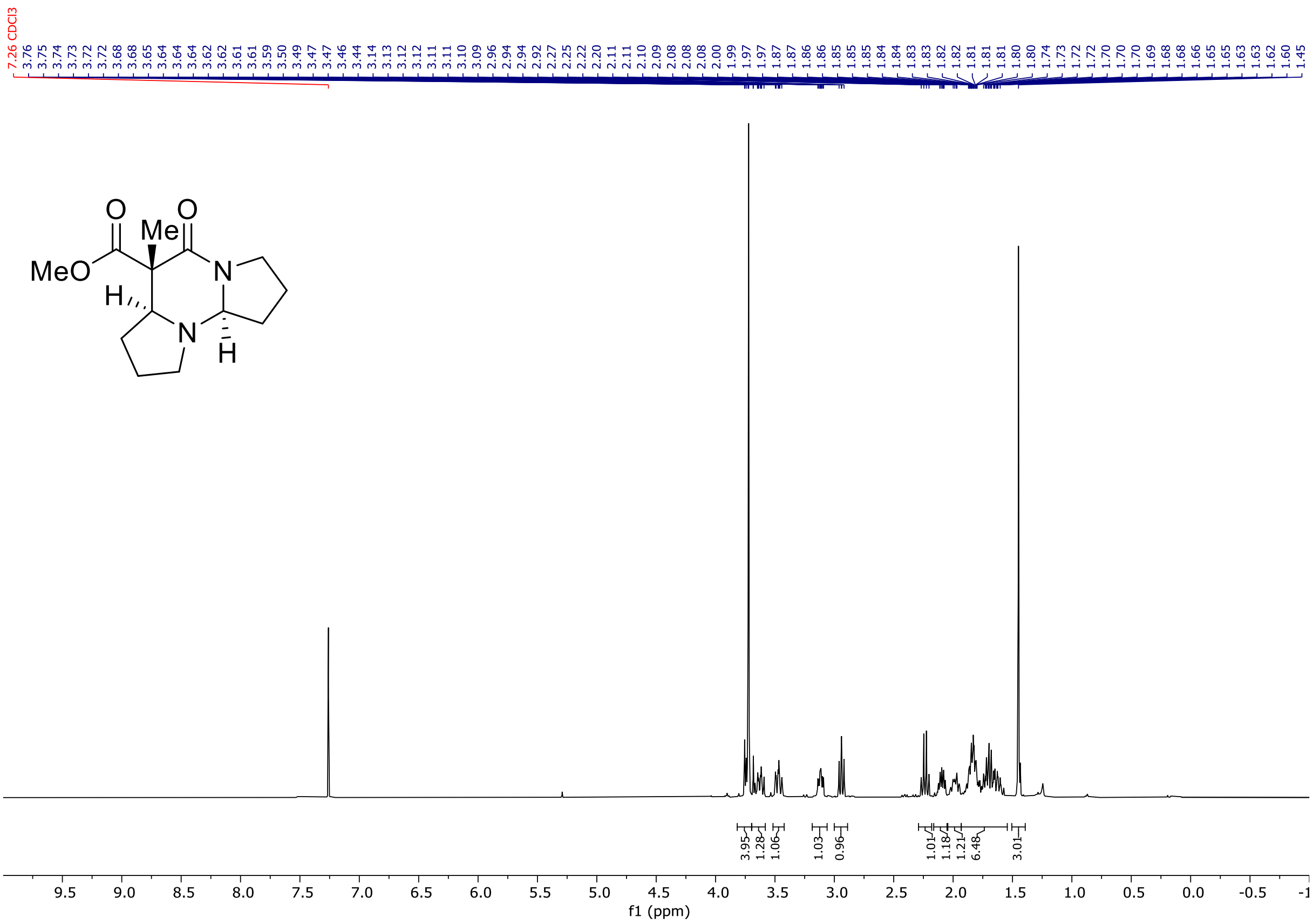

^{13}C NMR (101 MHz, $CDCl_3$) of **3i**:

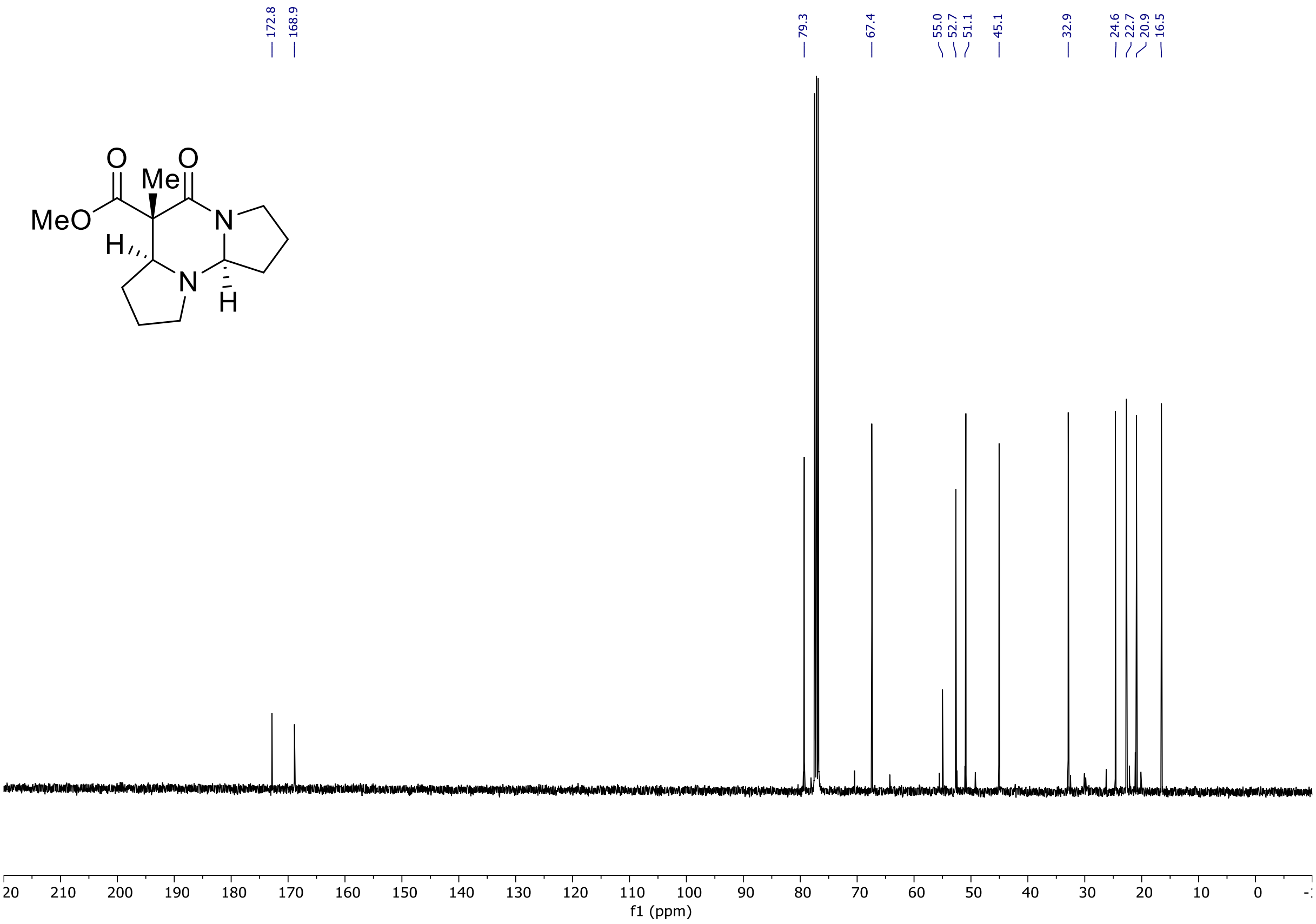

The enantiomeric access of **3i** was determined by chiral stationary phase SFC: Trefoil column, CO_2/MeOH 90:10, 2.0 mL/min, 254 nm; 2.6 min (major), 4.1 min (minor).

racemate

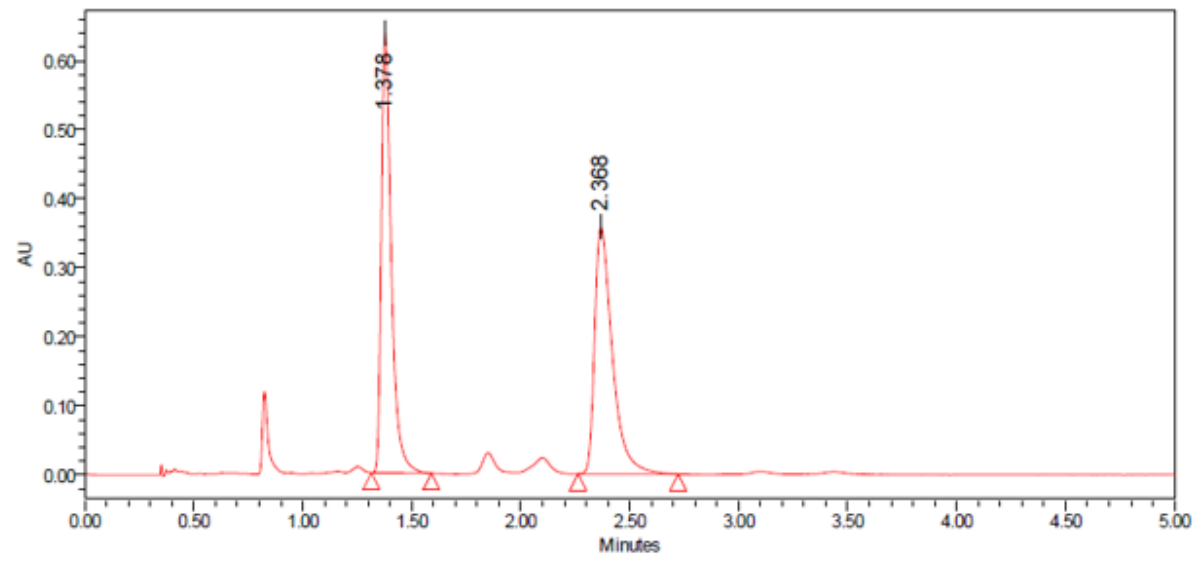

Peak Results

	Name	RT	Area	Height	% Area	Start Time (min)	End Time (min)
1		1.378	2029913	638115	50.14	1.315	1.590
2		2.368	2018187	357606	49.86	2.263	2.723

Isolated product

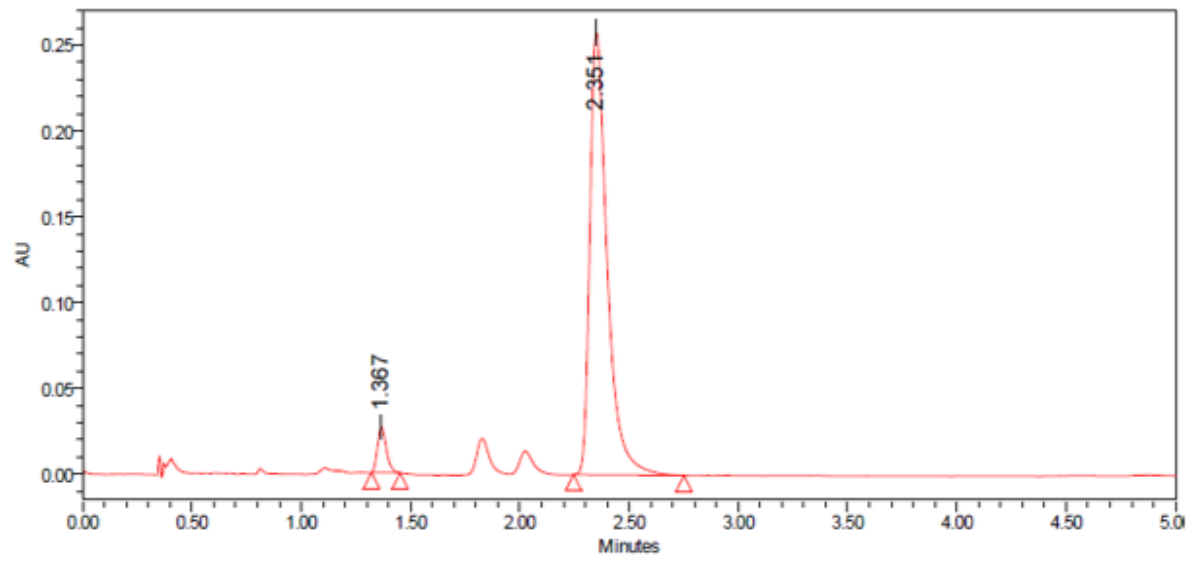

Peak Results

	Name	RT	Area	Height	% Area	Start Time (min)	End Time (min)
1		1.367	75112	26713	5.02	1.322	1.453
2		2.351	1422327	257705	94.98	2.246	2.752

9.2 NMR and Chiral Stationary Phase HPLC Chromatograms 6-6-5 ring systems

^{1}H NMR (500 MHz, $CDCl_3$) of **4a**:

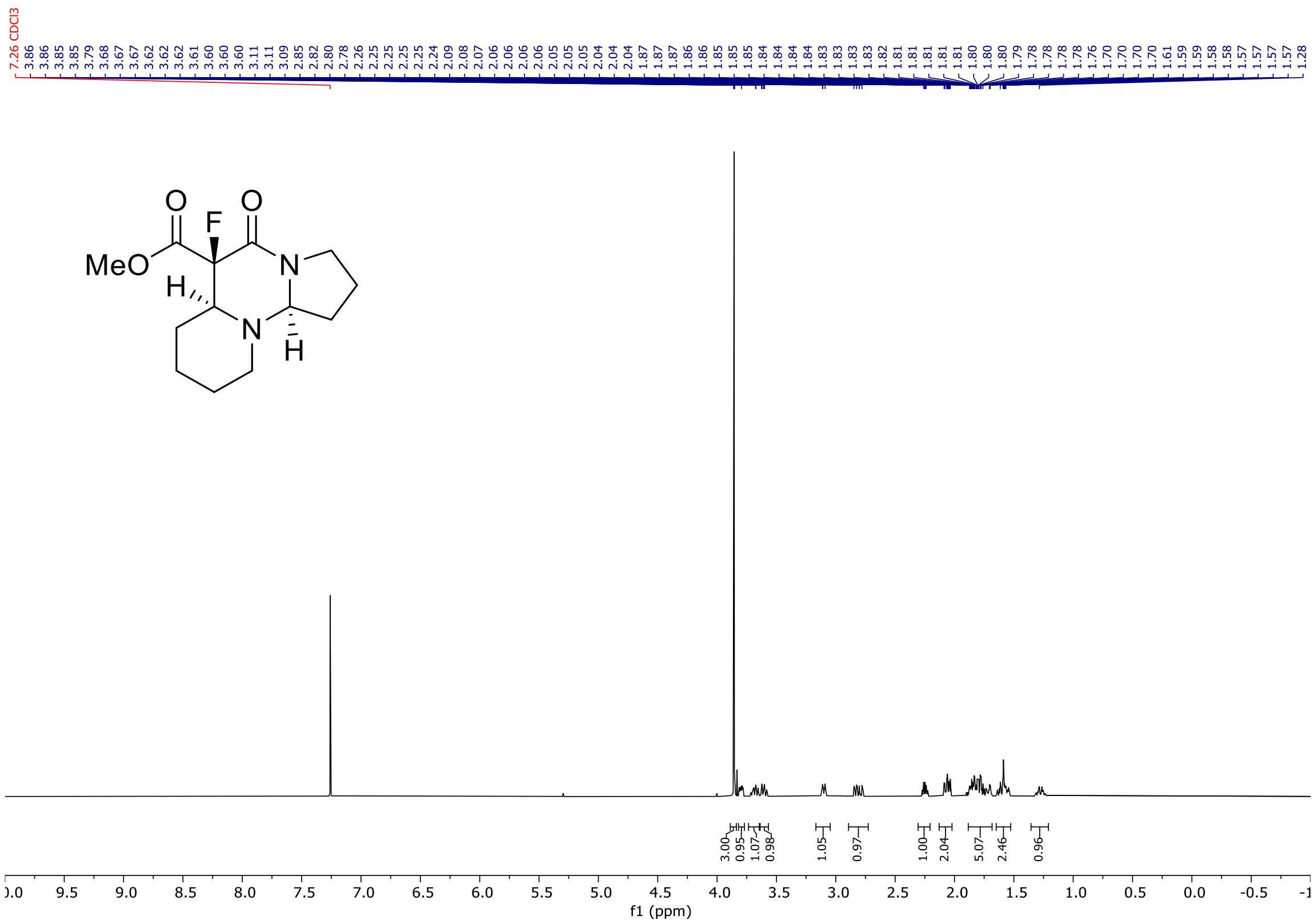

^{19}F NMR {^{1}H} (471 MHz, $CDCl_3$) of **4a**:

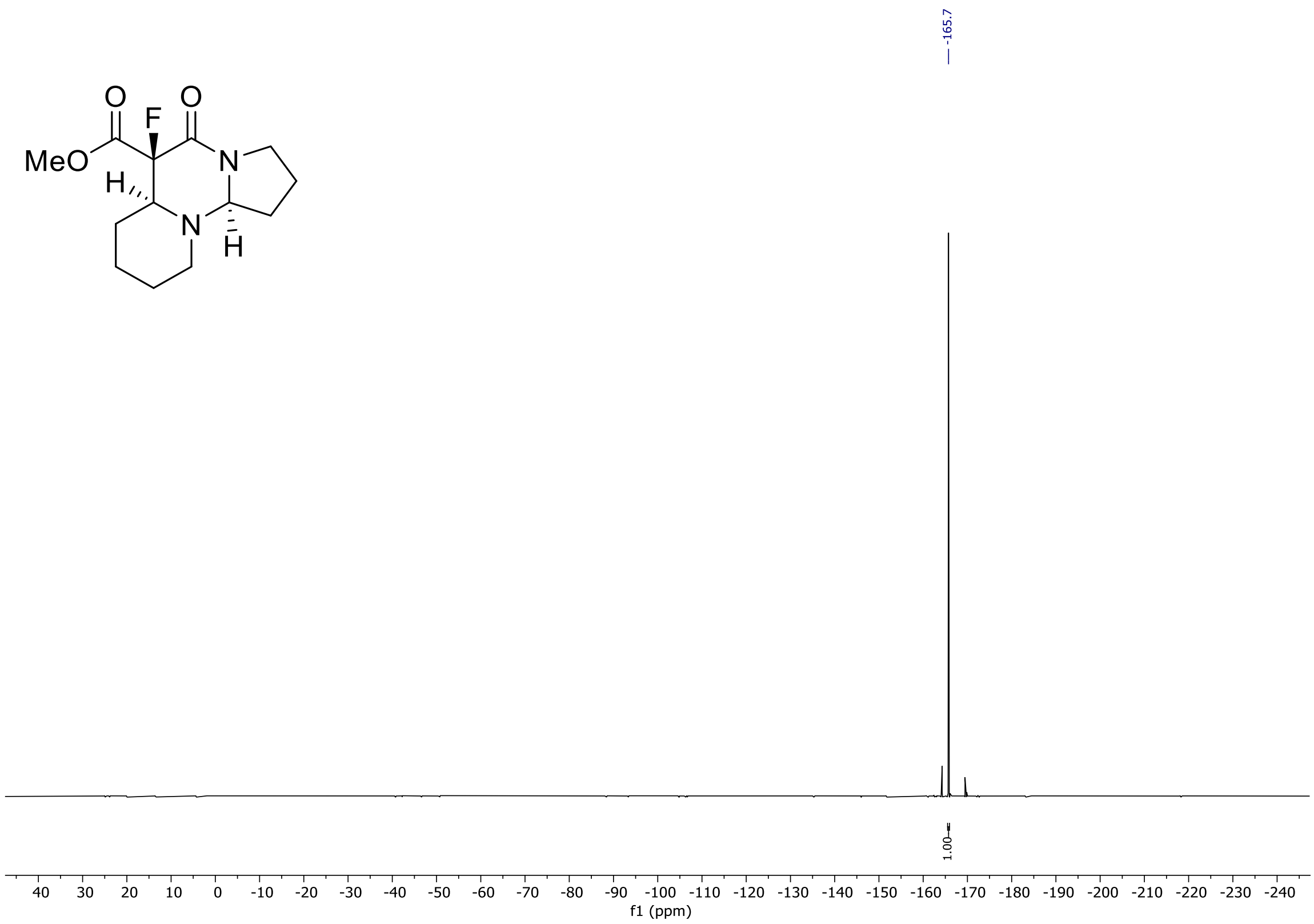

^{19}F NMR (471 MHz, $CDCl_3$) of **4a**:

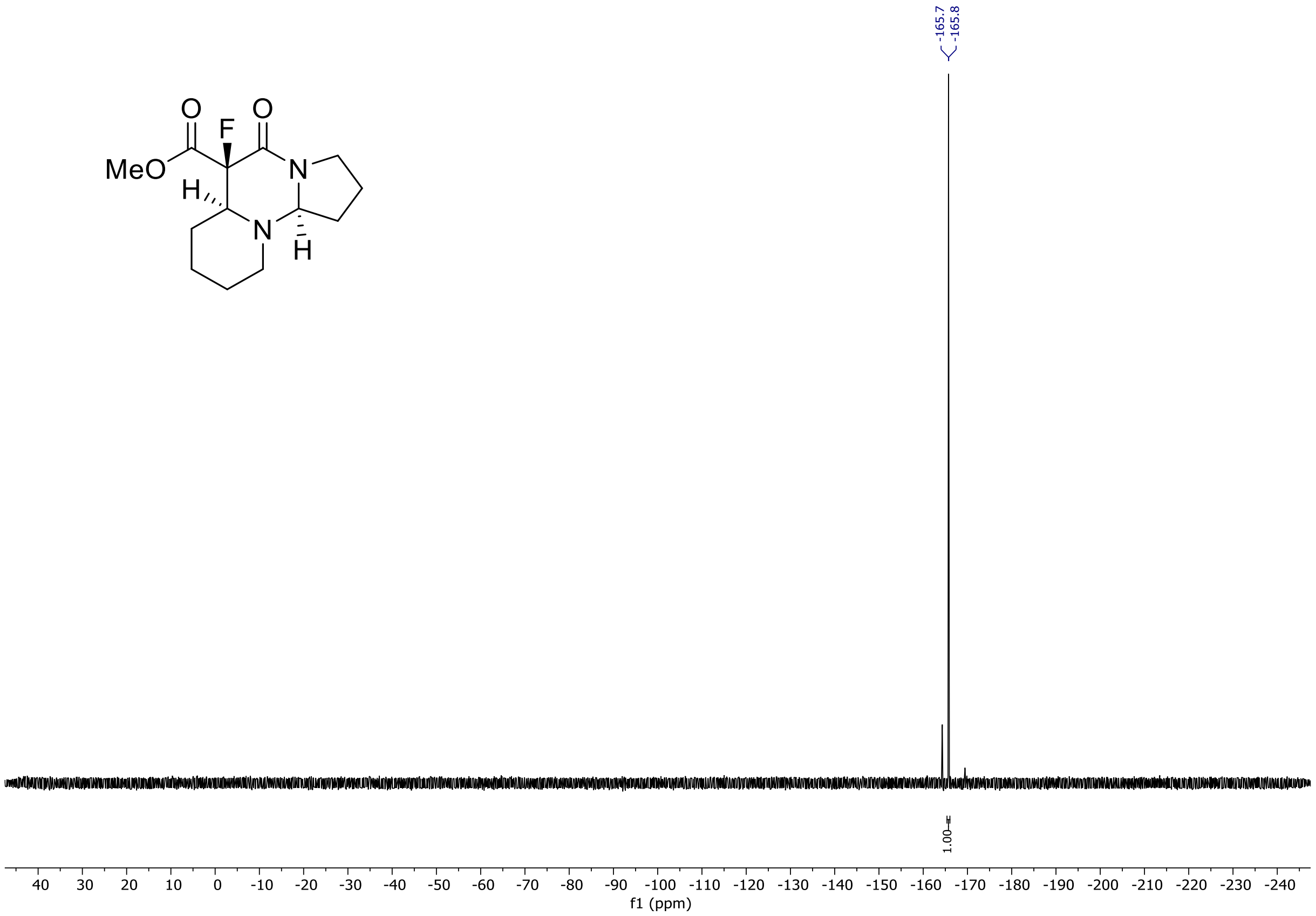

^{13}C NMR (125 MHz, $CDCl_3$) of **4a**:

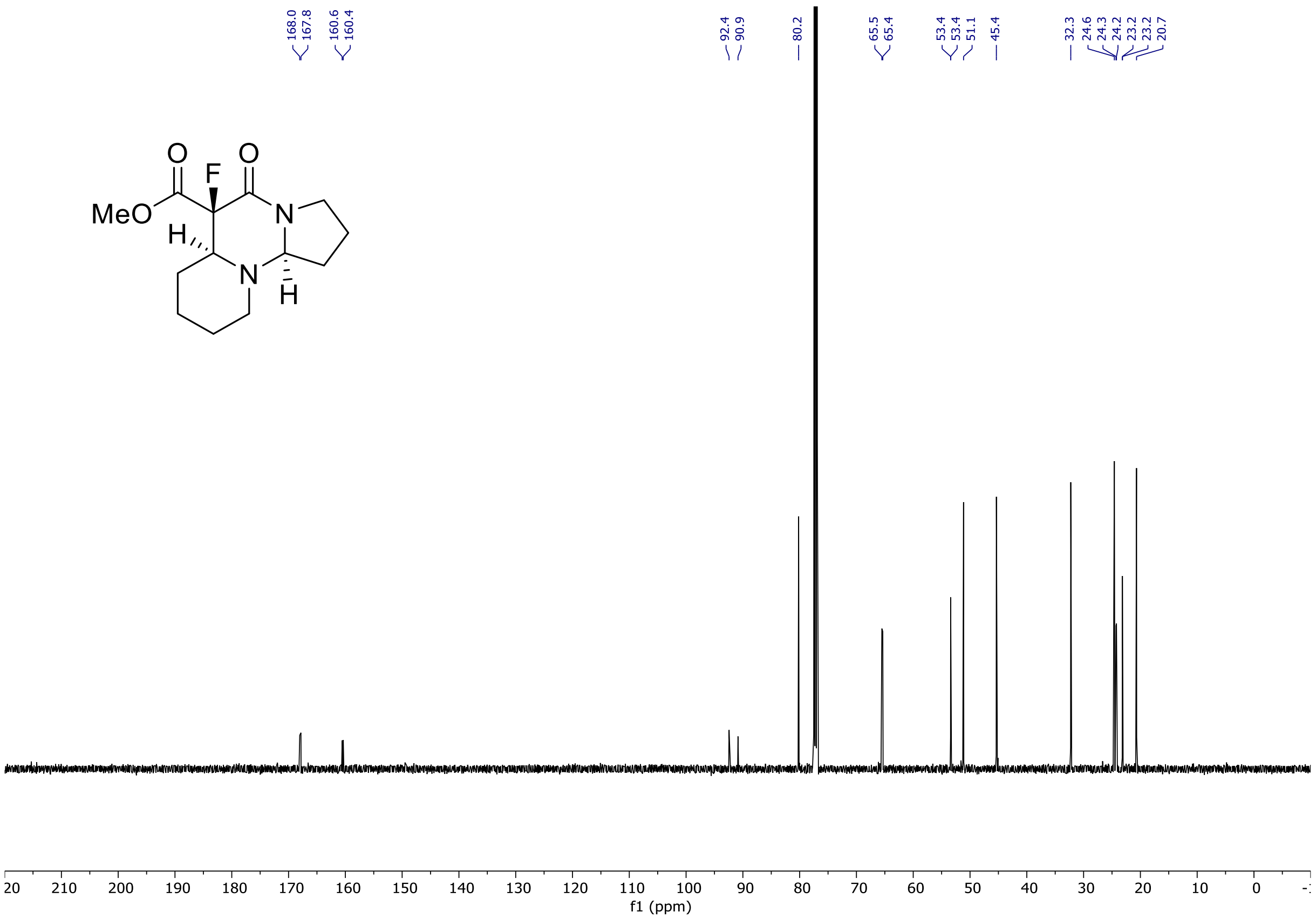

The enantiomeric access of **4a** was determined by chiral stationary phase SFC: IC3 column, CO_2/MeOH 85:15, 2.0 mL/min, 214 nm; 4.2 min (major), 6.2 min (minor).

racemate

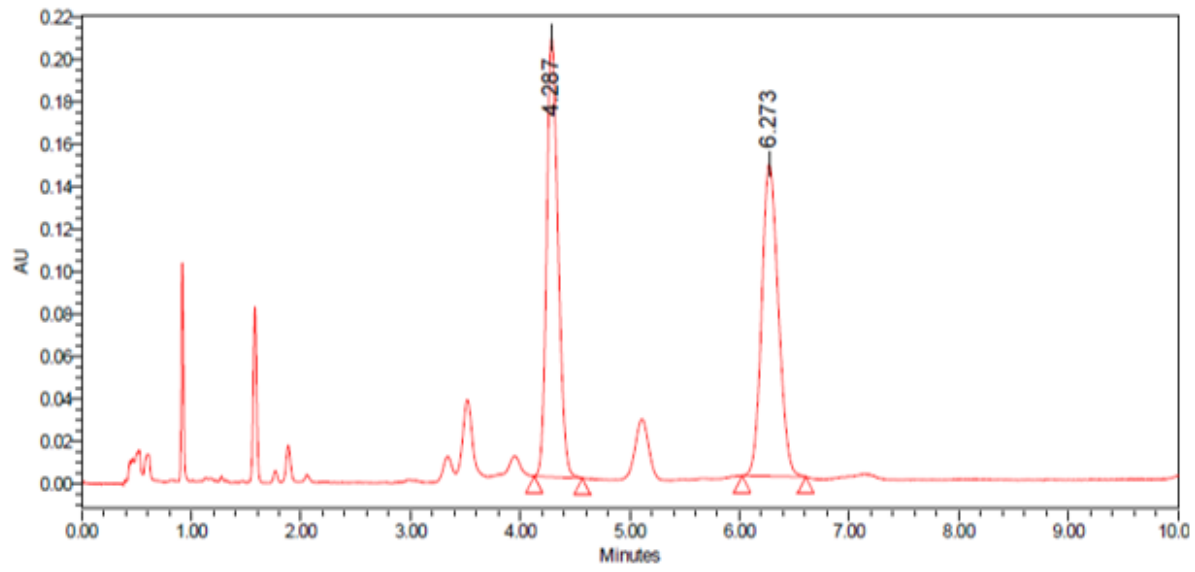

Peak Results

	Name	RT	Area	Height	% Area	Start Time (min)	End Time (min)
1		4.287	1499466	207196	49.17	4.128	4.567
2		6.273	1549987	147301	50.83	6.025	6.606

Isolated product

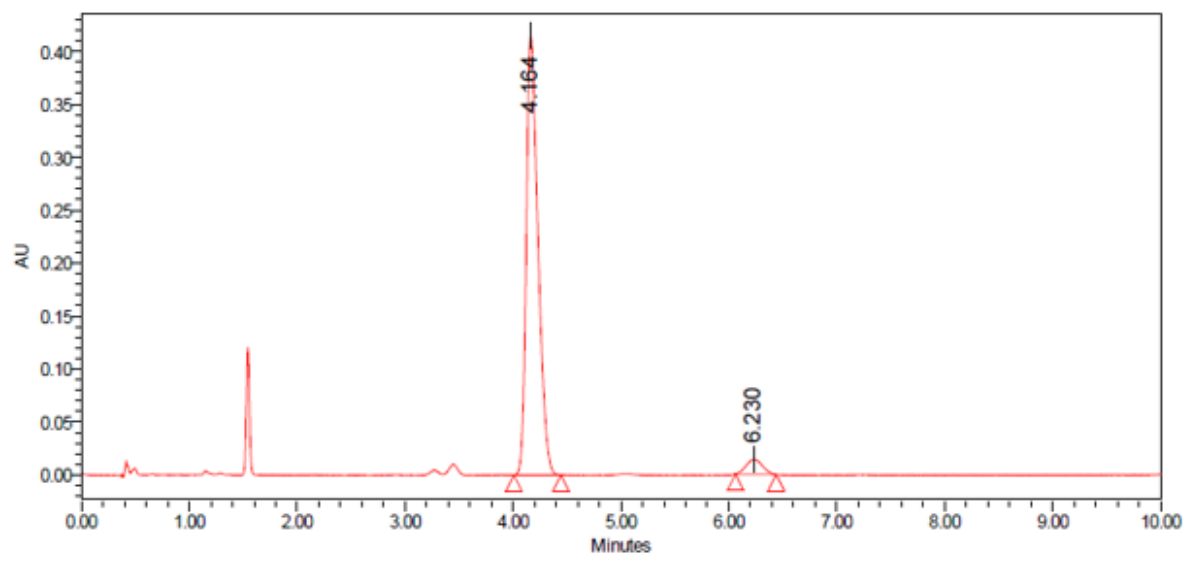

Peak Results

	Name	RT	Area	Height	% Area	Start Time (min)	End Time (min)
1		4.164	3117593	414938	95.40	4.008	4.444
2		6.230	150322	14268	4.60	6.061	6.440

^{1}H NMR (400 MHz, $CDCl_3$) of *ent*-**4a**:

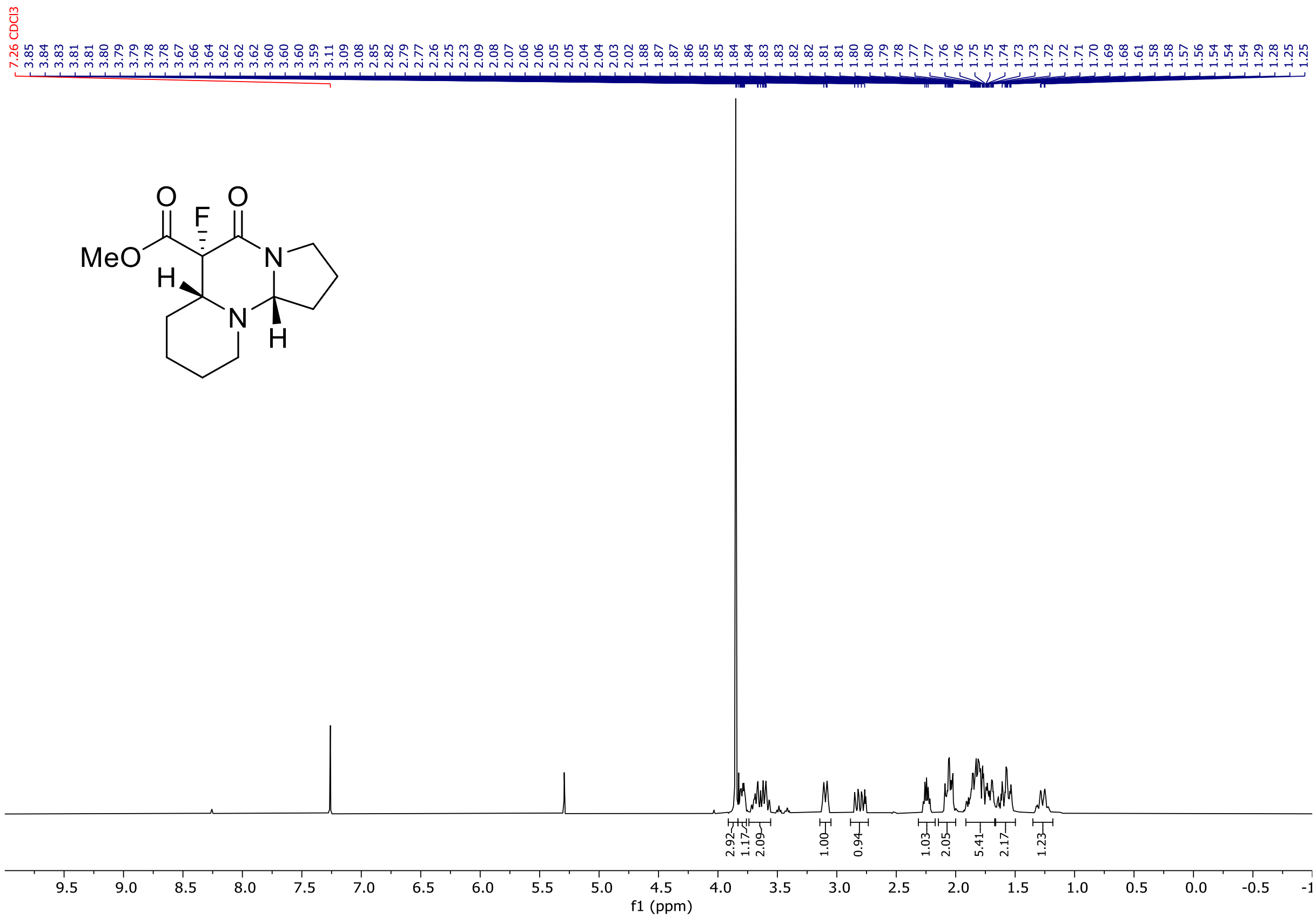

^{19}F NMR {^{1}H} (377 MHz, $CDCl_3$) of *ent*-**4a**:

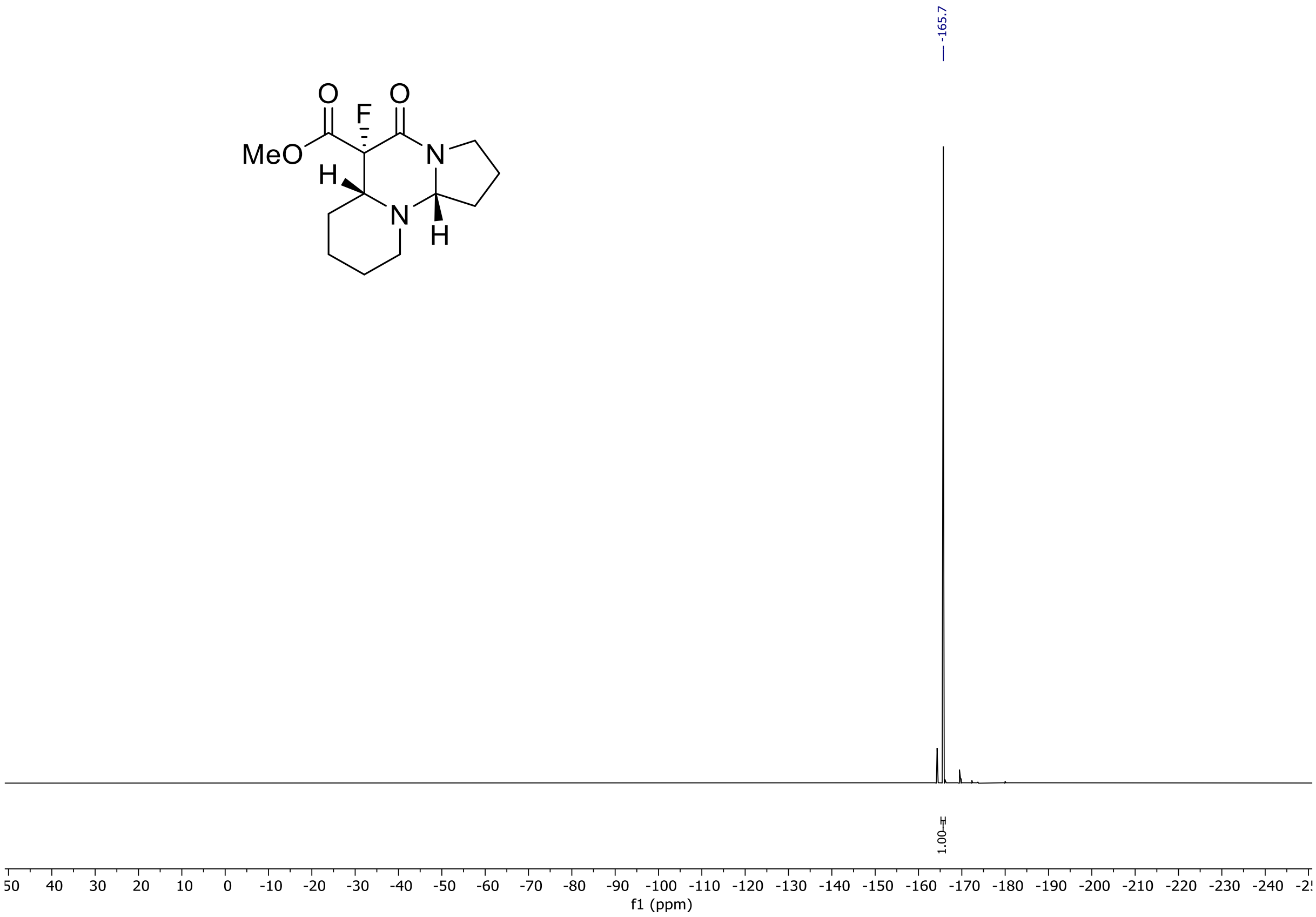

The enantiomeric access of *ent*-**4a** was determined by chiral stationary phase SFC: IC3 column, CO_2/MeOH 85:15, 2.0 mL/min, 214 nm; 4.3 min (minor), 6.3 min (major).

racemate

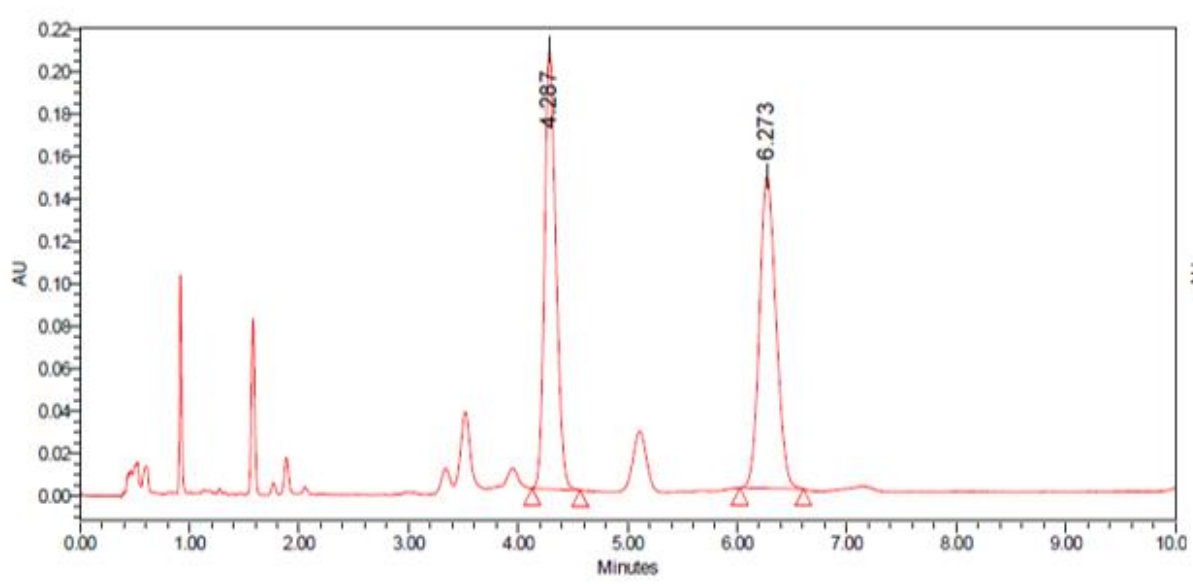

Peak Results

	Name	RT	Area	Height	% Area	Start Time (min)	End Time (min)
1		4.287	1499466	207196	49.17	4.128	4.567
2		6.273	1549987	147301	50.83	6.025	6.606

Isolated product

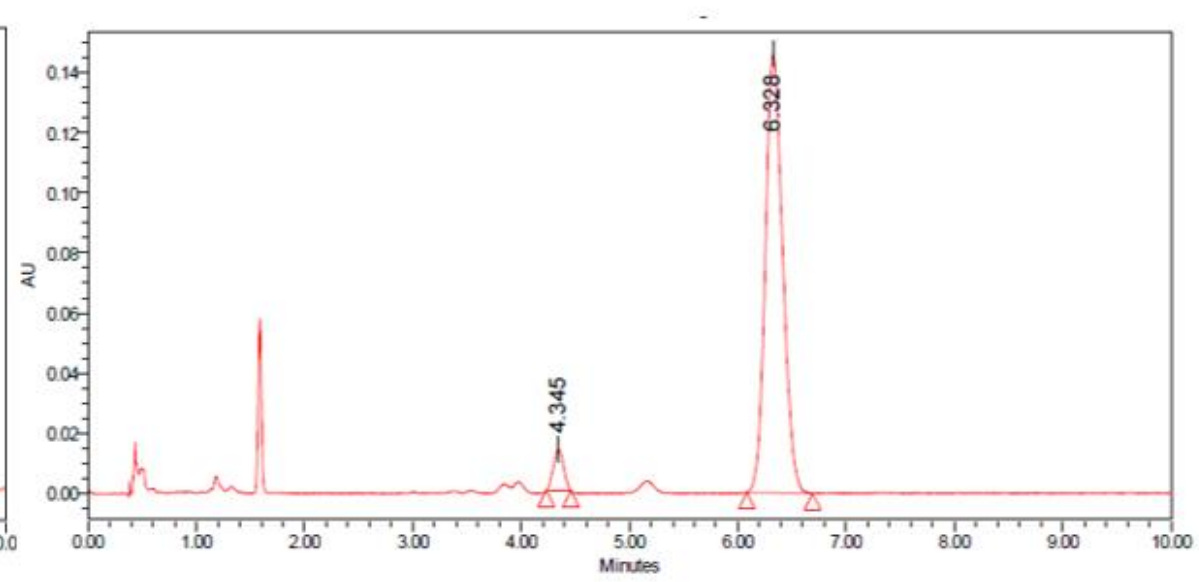

Peak Results

	Name	RT	Area	Height	% Area	Start Time (min)	End Time (min)
1		4.345	89246	13714	5.20	4.233	4.458
2		6.328	1626581	146027	94.80	6.084	6.691

^{1}H NMR (500 MHz, $CDCl_3$) of **4b**:

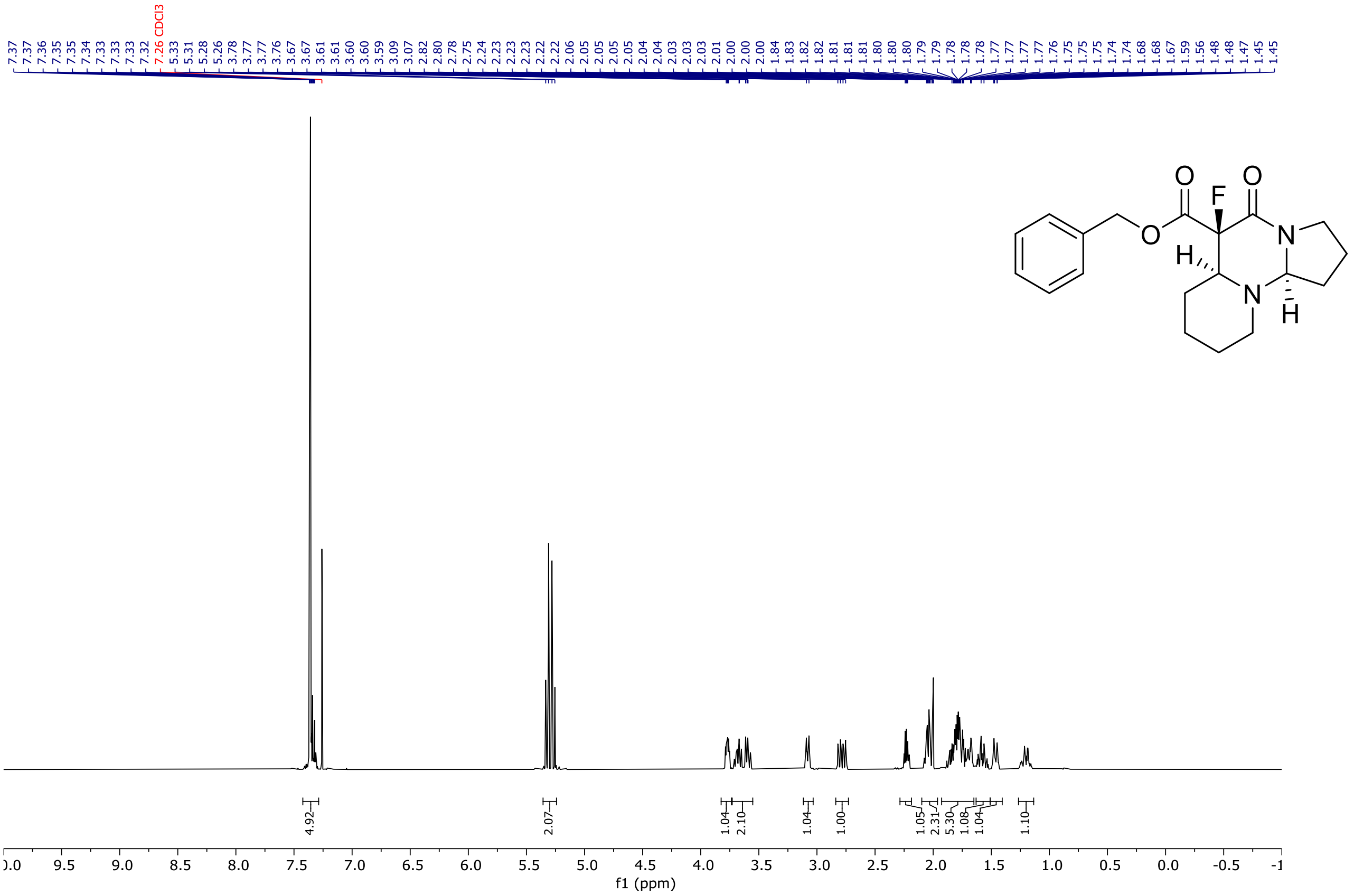

^{19}F NMR {^{1}H} (471 MHz, $CDCl_3$) of **4b**:

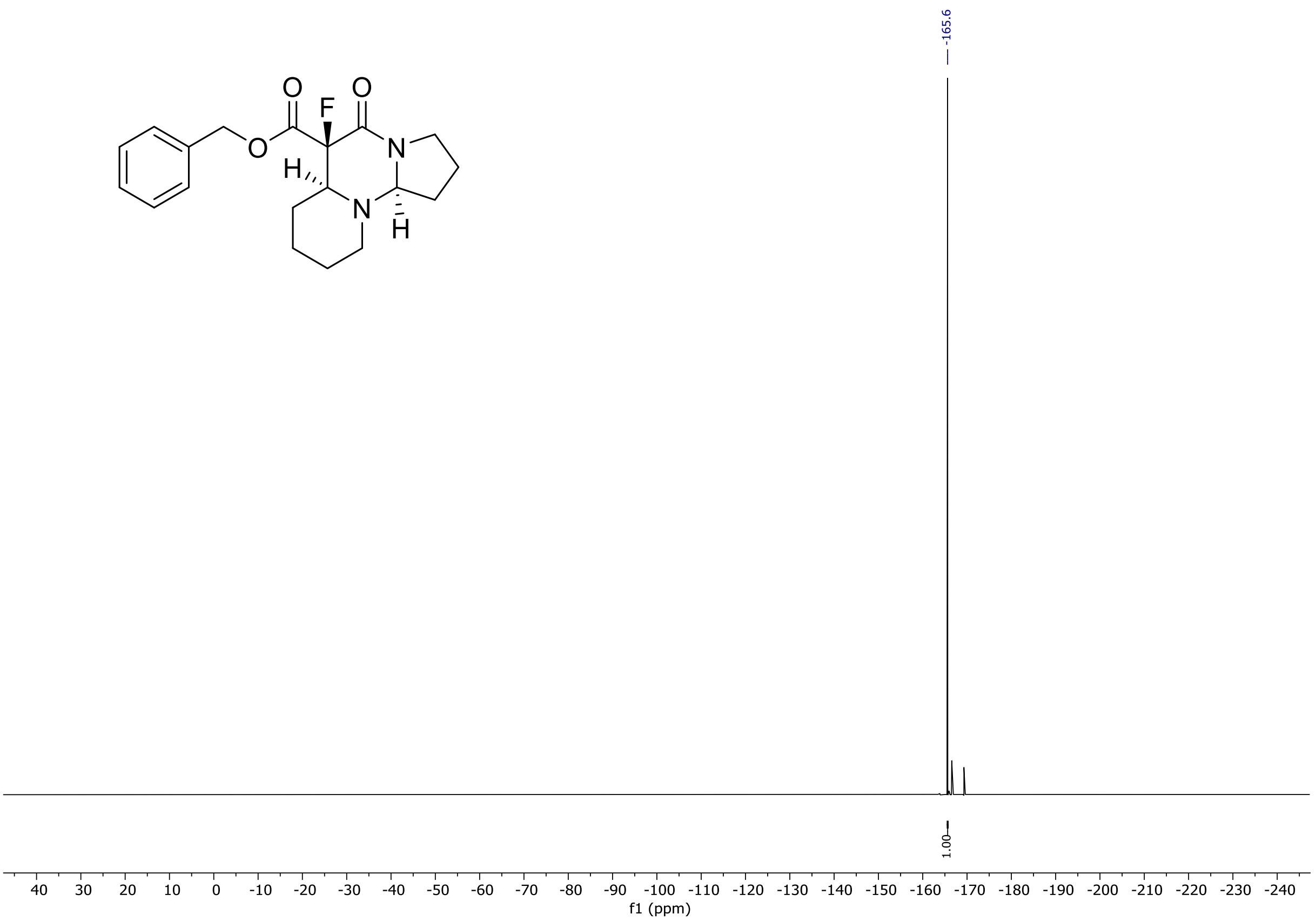

^{19}F NMR (471 MHz, $CDCl_3$) of **4b**:

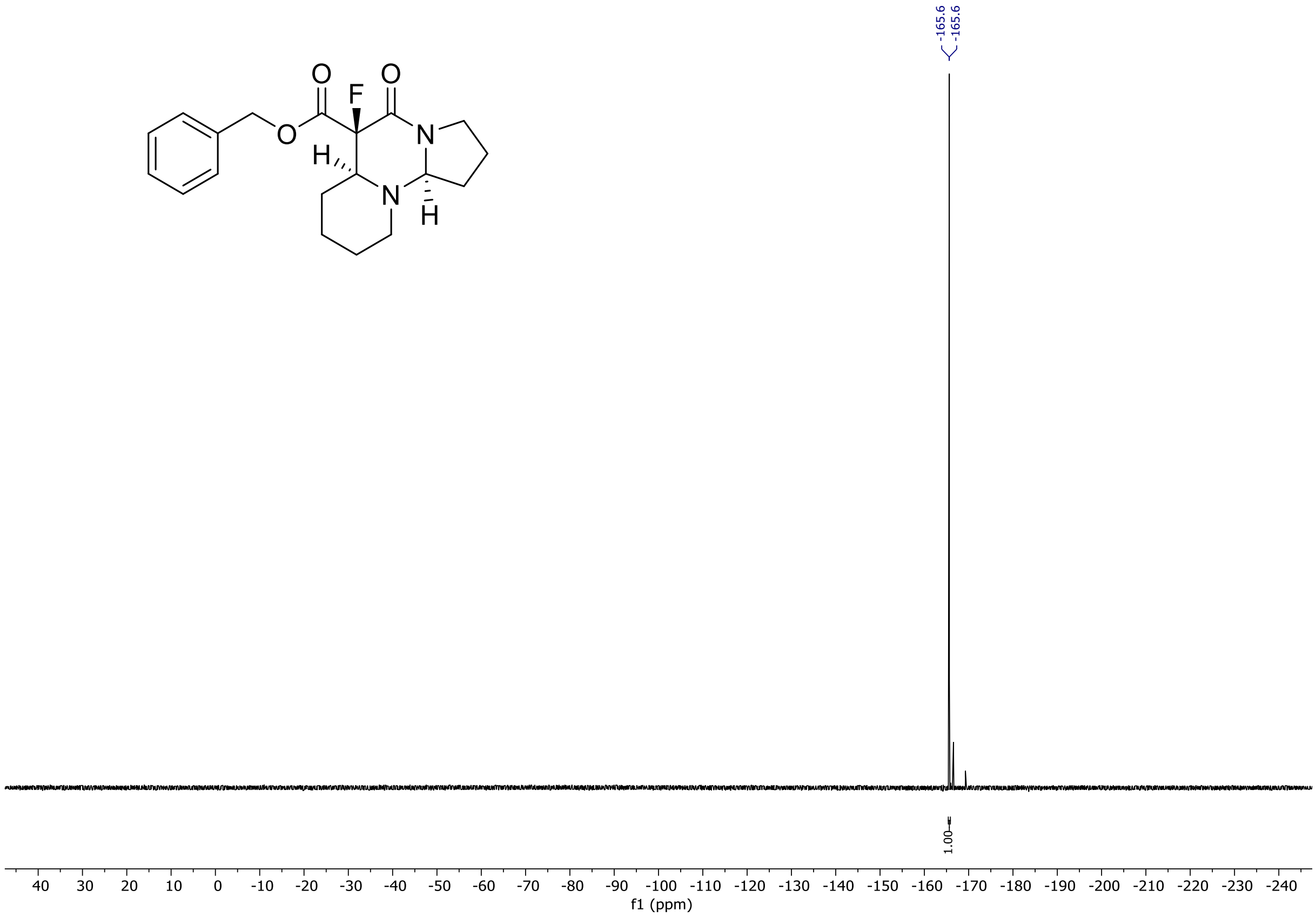

^{13}C NMR (101 MHz, $CDCl_3$) of **2a**:

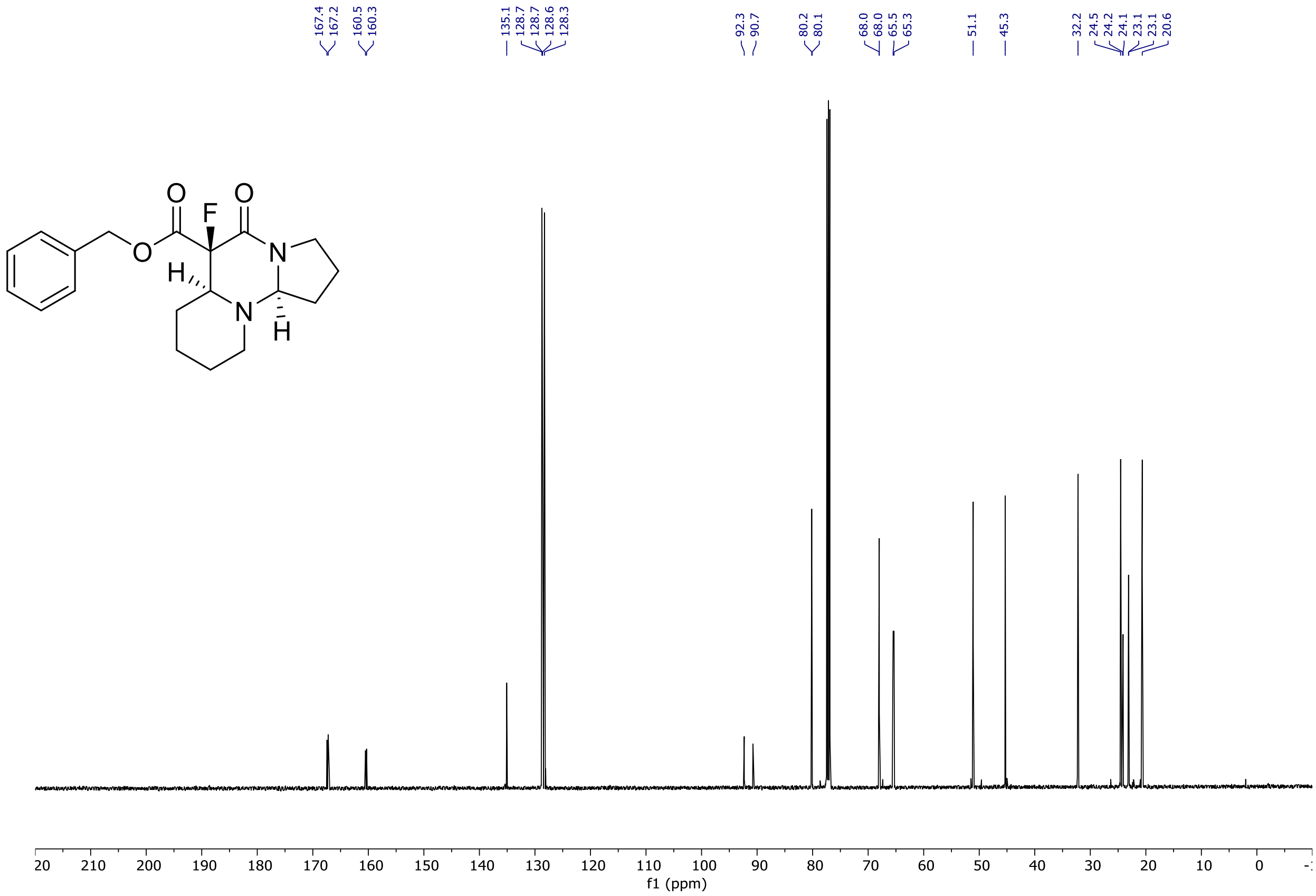

The enantiomeric access of **4b** was determined by chiral stationary phase SFC: Whelk column, CO_2/MeOH 85:15, 2.0 mL/min, 214 nm; 10.3 min (major), 12.9 min (minor).

racemate

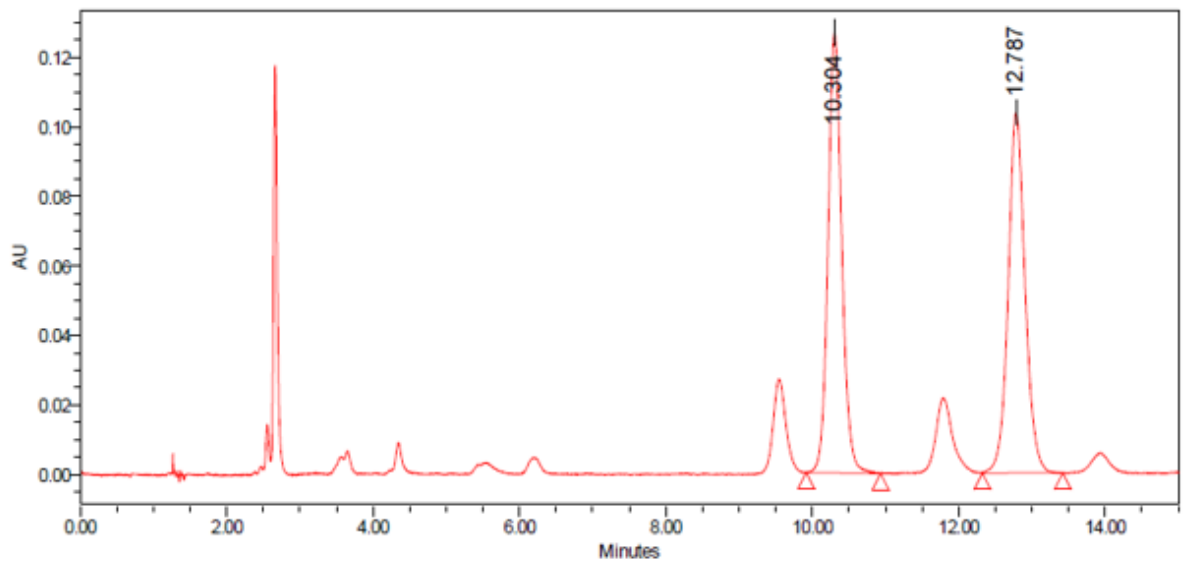

Peak Results

	Name	RT	Area	Height	% Area	Start Time (min)	End Time (min)
1		10.304	1651751	126578	49.82	9.917	10.933
2		12.787	1663404	103664	50.18	12.323	13.423

Isolated product

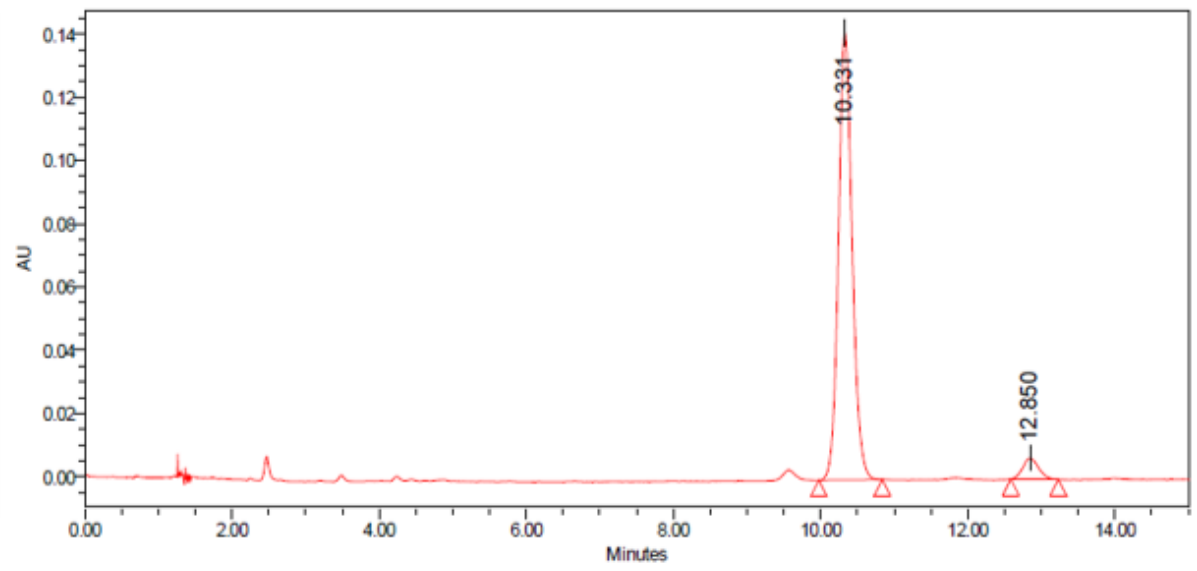

Peak Results

	Name	RT	Area	Height	% Area	Start Time (min)	End Time (min)
1		10.331	1826983	141270	94.84	9.977	10.838
2		12.850	99452	6609	5.16	12.586	13.233

9.3 NMR Spectra and Chiral Stationary Phase HPLC Chromatograms of mono addition products

^{1}H NMR (400 MHz, $CDCl_3$) of **5a**:

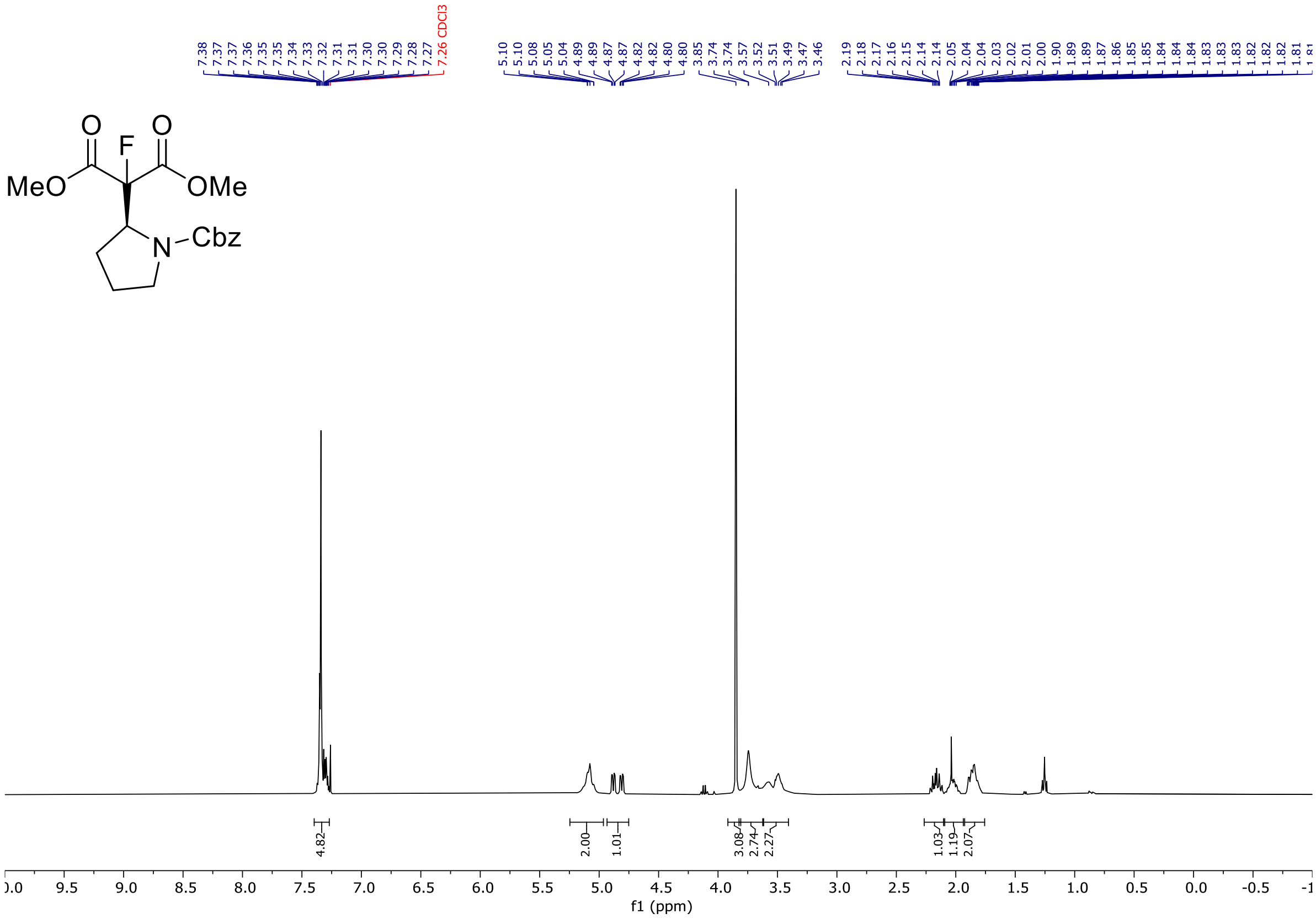

^{19}F NMR {^{1}H} (377 MHz, $CDCl_3$) of **5a**:

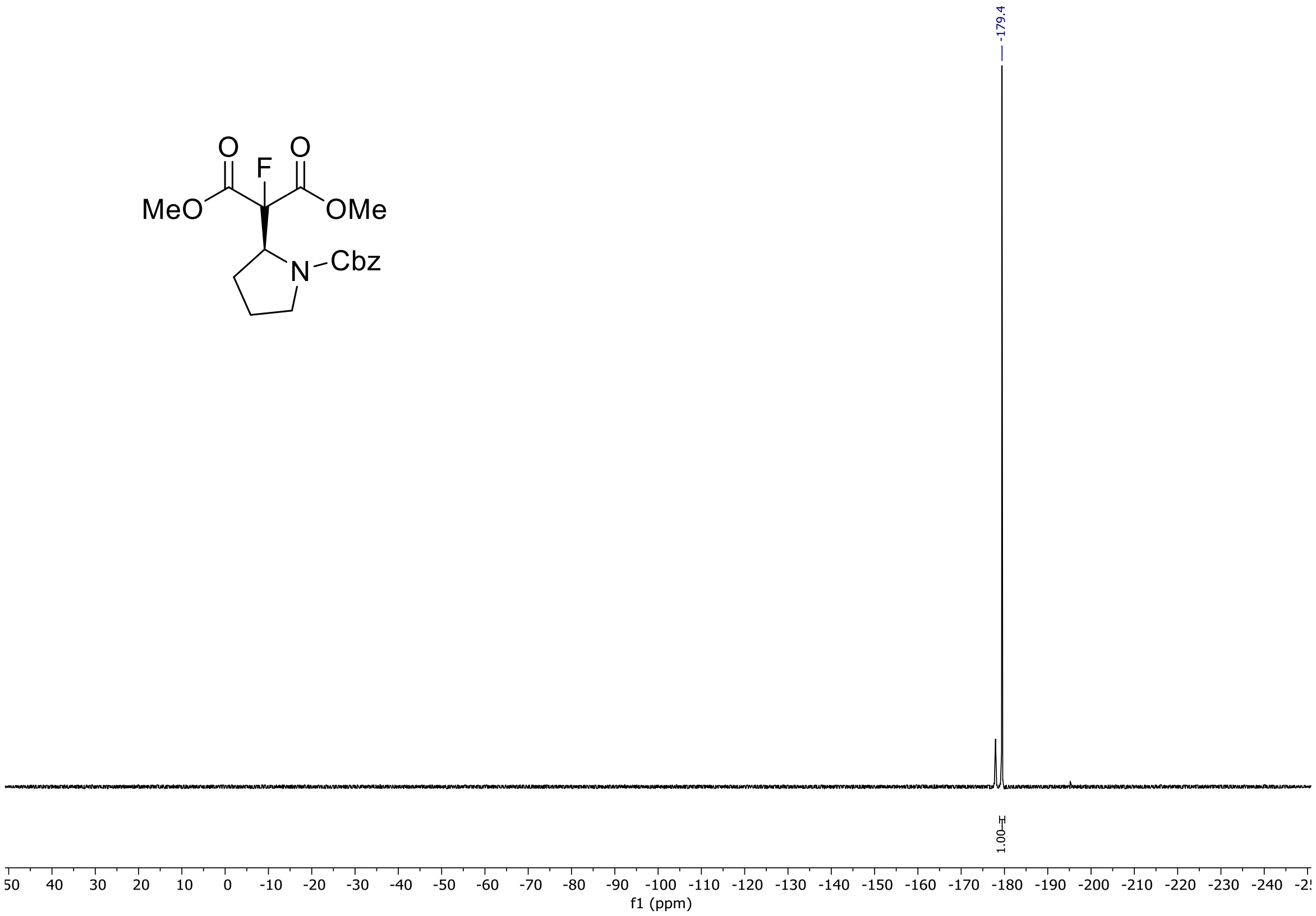

^{19}F NMR (377 MHz, CDCl$_3$) of **5a**:

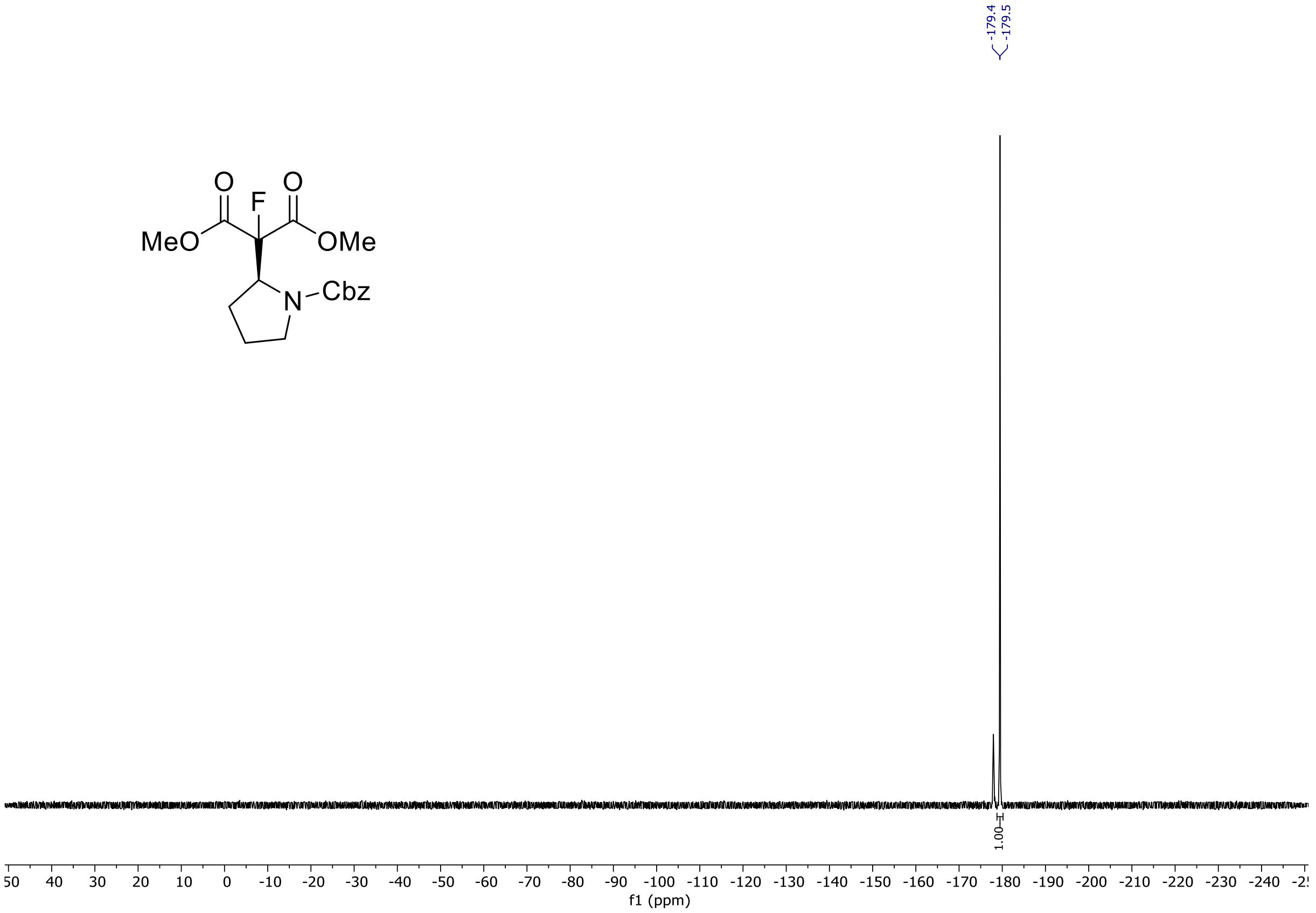

^{13}C NMR (101 MHz, CDCl$_3$) of **5a**:

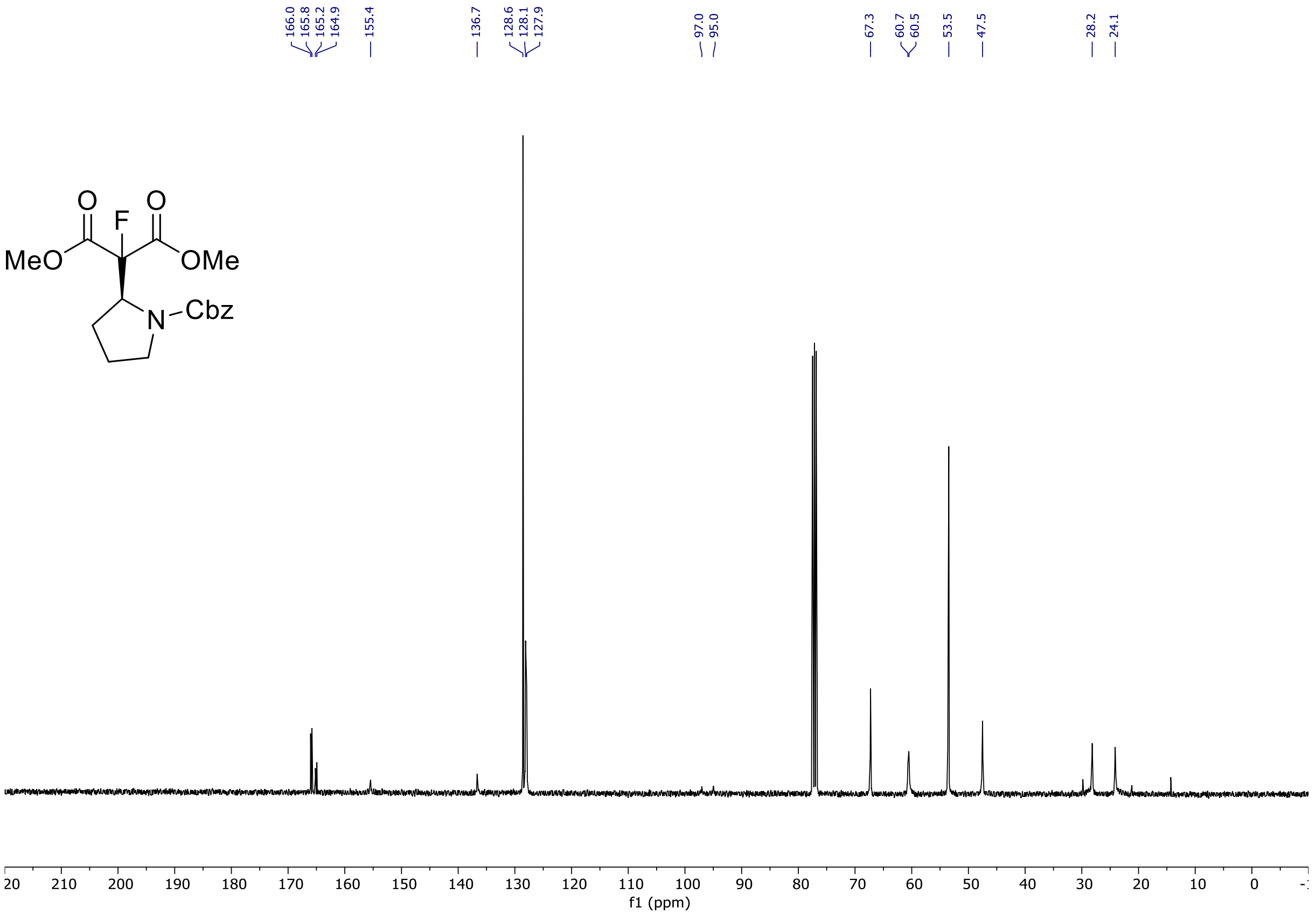

The enantiomeric access of **5a** was determined by chiral stationary phase SFC: IC3 column, CO_2/MeOH 93:7, 2.0 mL/min, 214 nm; 1.9 min (minor), 2.2 min (major).

racemate

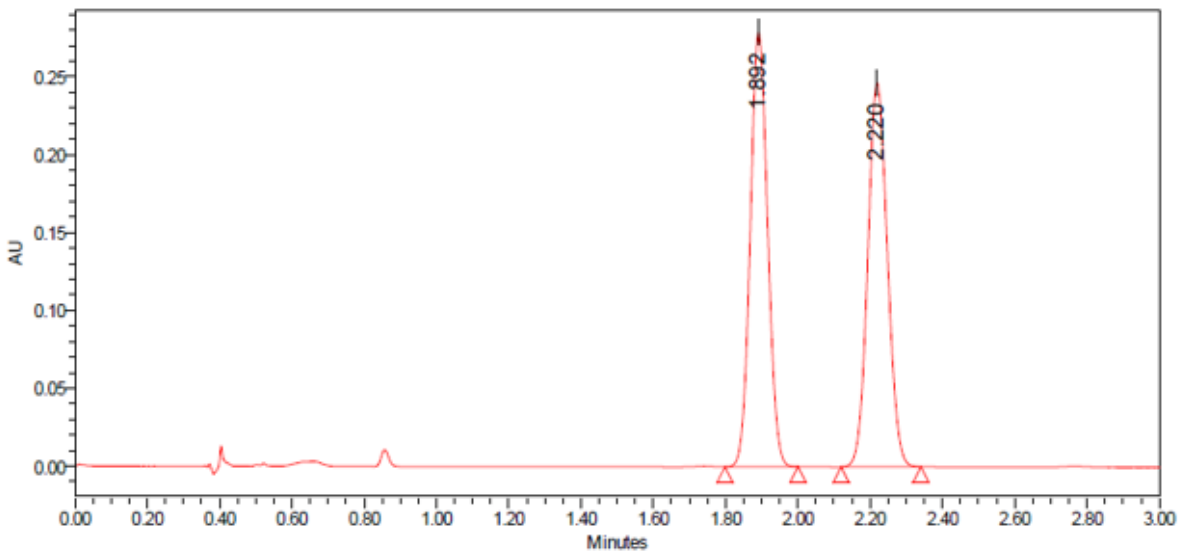

Peak Results

	Name	RT	Area	Height	% Area	Start Time (min)	End Time (min)
1		1.892	931177	279167	50.00	1.798	2.001
2		2.220	931075	246578	50.00	2.120	2.341

Isolated product

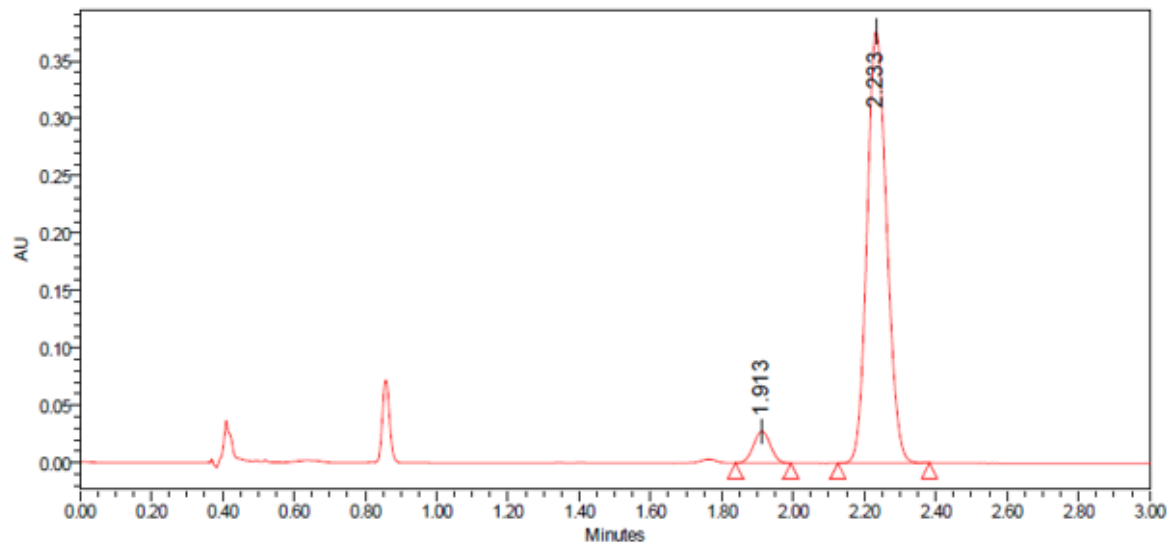

Peak Results

	Name	RT	Area	Height	% Area	Start Time (min)	End Time (min)
1		1.913	91969	27724	6.01	1.838	1.994
2		2.233	1439138	375869	93.99	2.126	2.383

^{1}H NMR (500 MHz, $CDCl_3$) of **5b**:

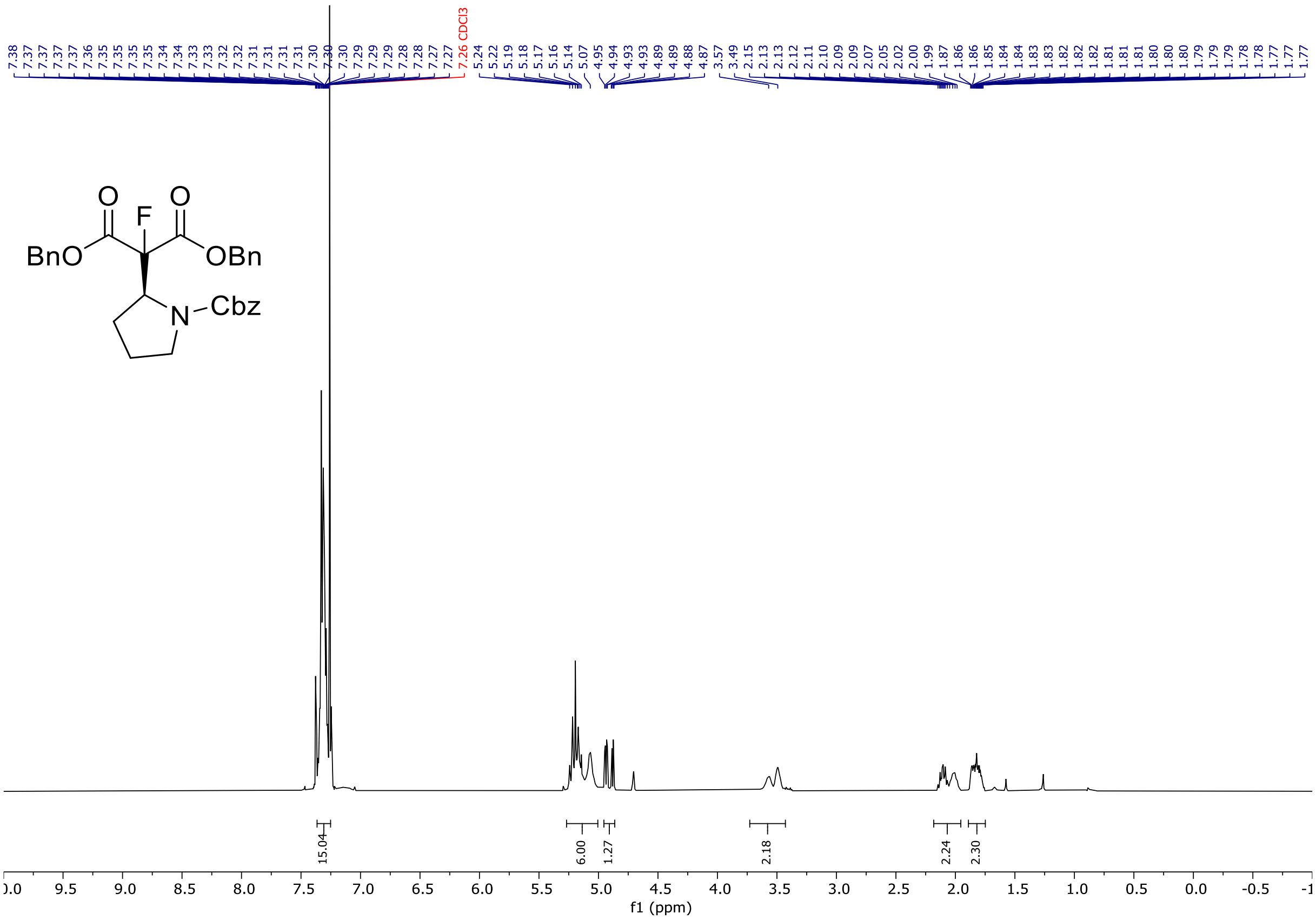

^{19}F NMR {^{1}H} (471 MHz, $CDCl_3$) of **5b**:

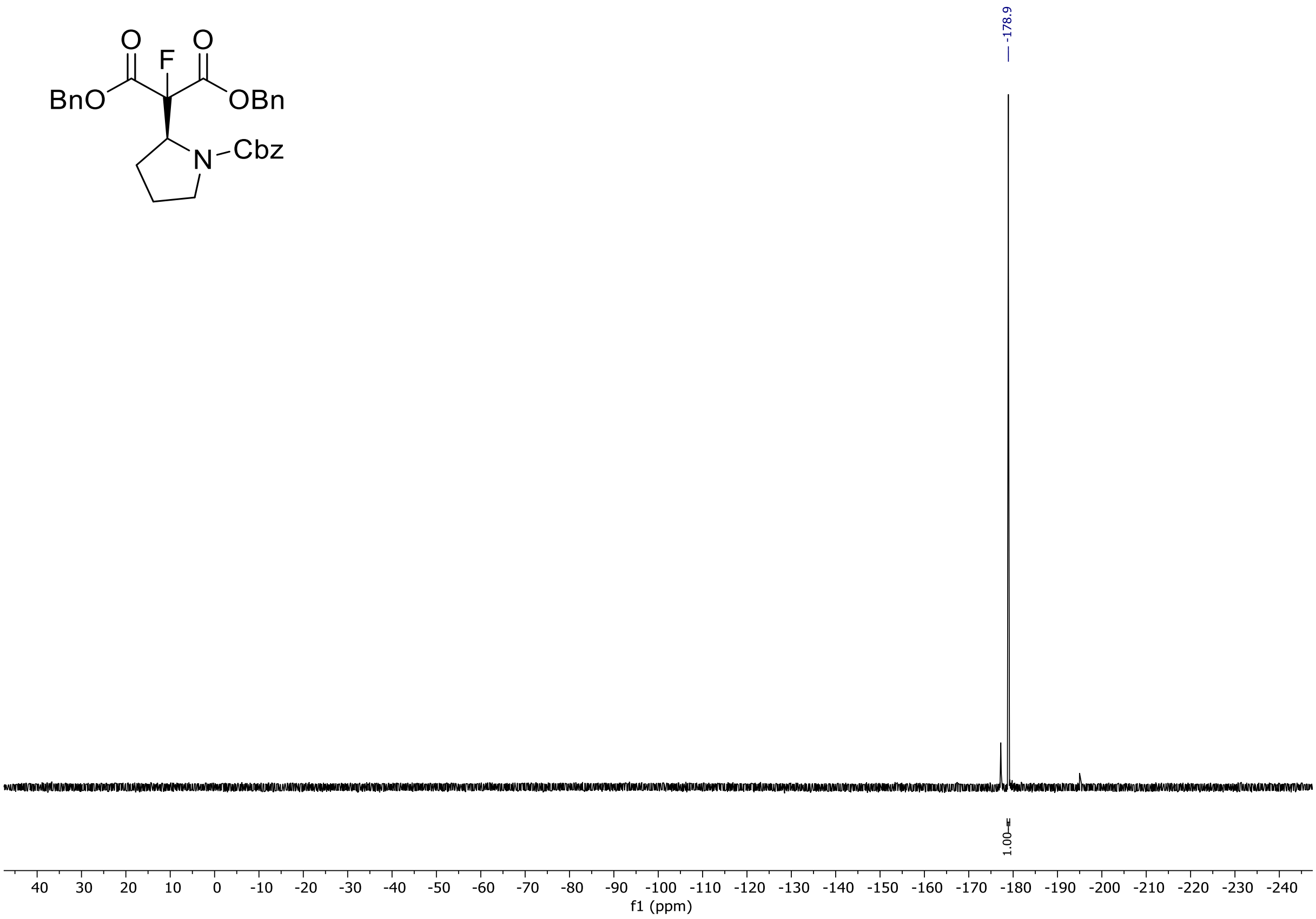

^{19}F NMR (471 MHz, $CDCl_3$) of **5b**:

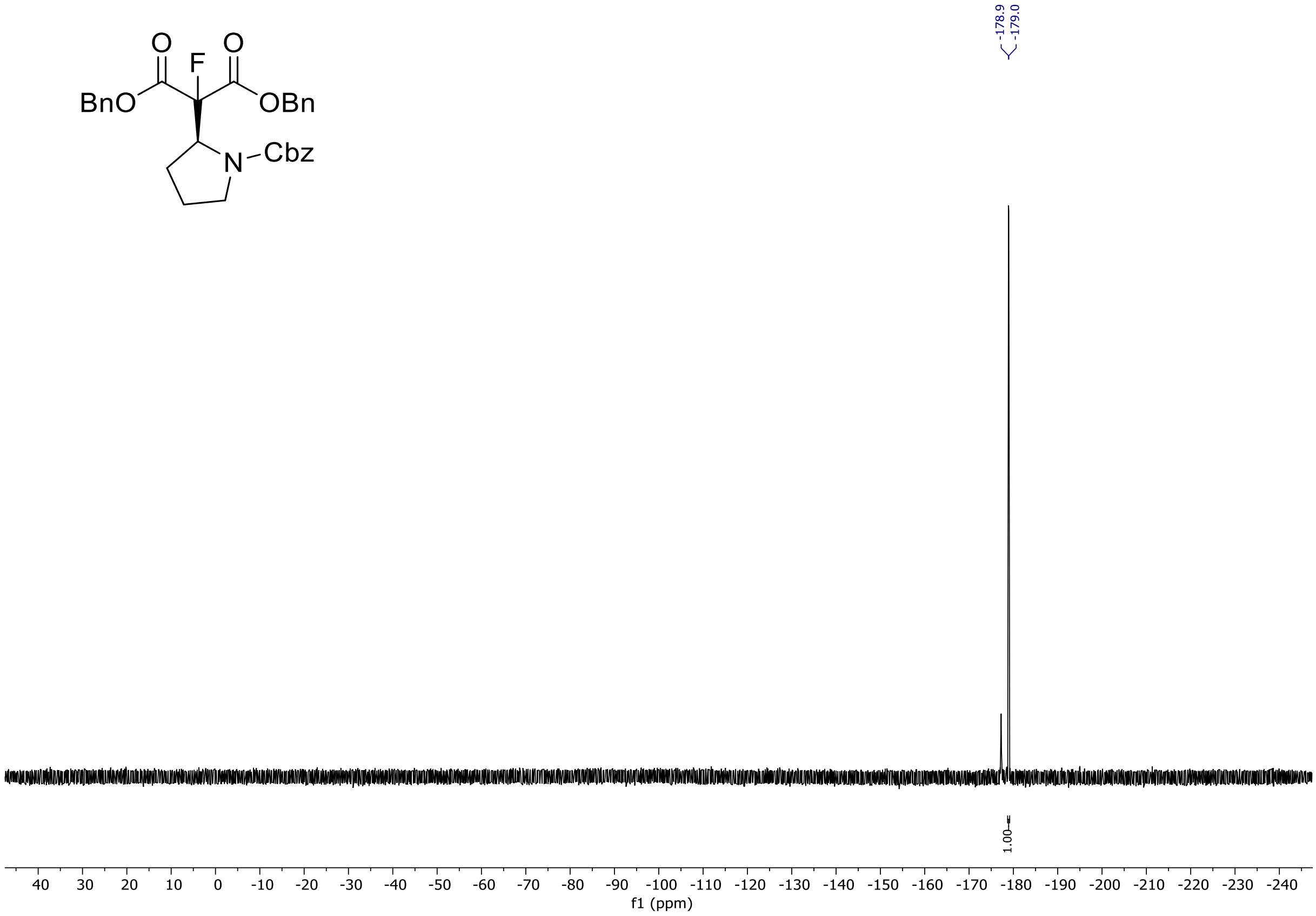

^{13}C NMR (126 MHz, $CDCl_3$) of **5b**:

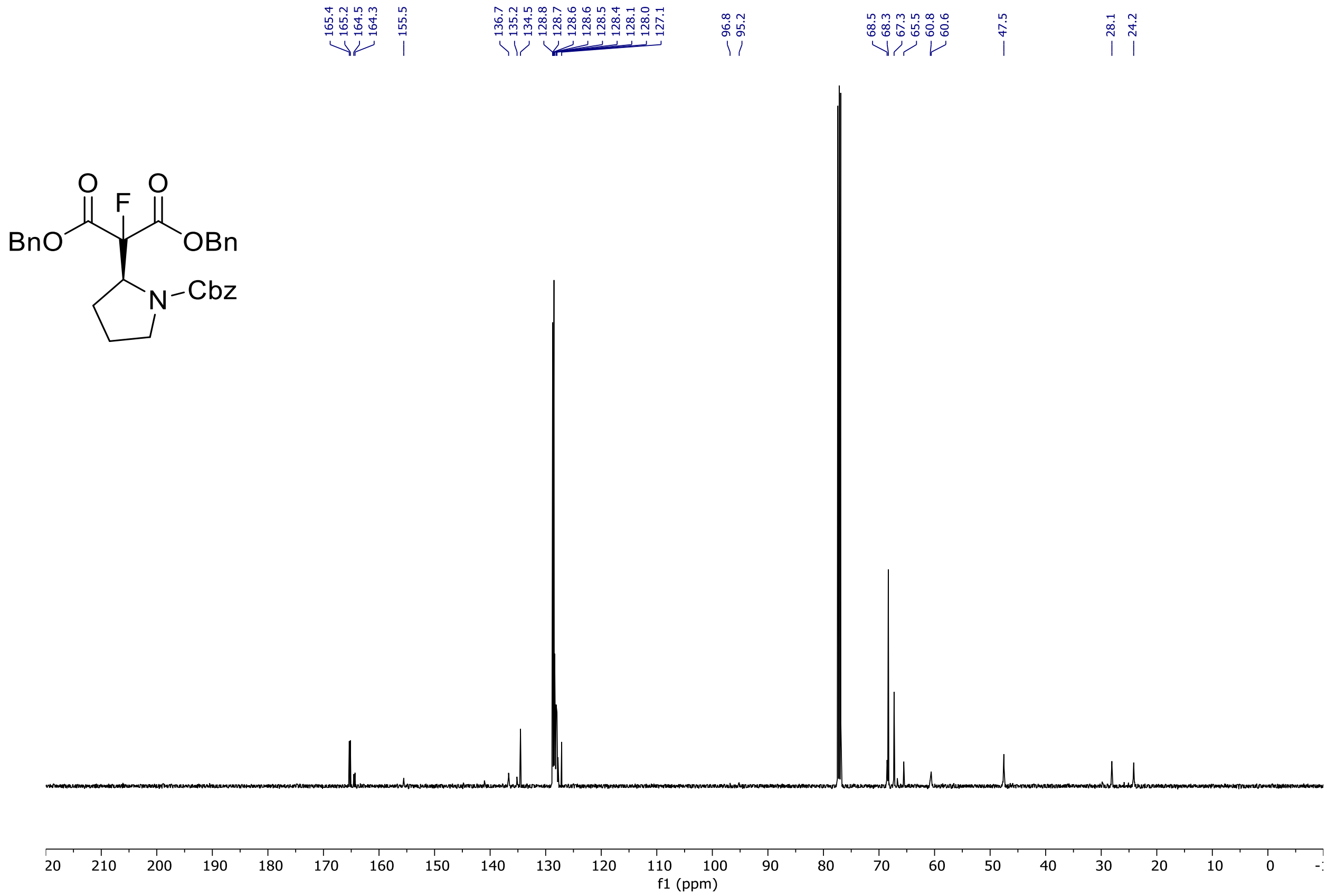

The enantiomeric access of **5b** was determined by chiral stationary phase SFC: IC3 column, CO_2/MeOH 85:15, 2.0 mL/min, 214 nm; 2.0 min (major), 2.3 min (minor).

racemate

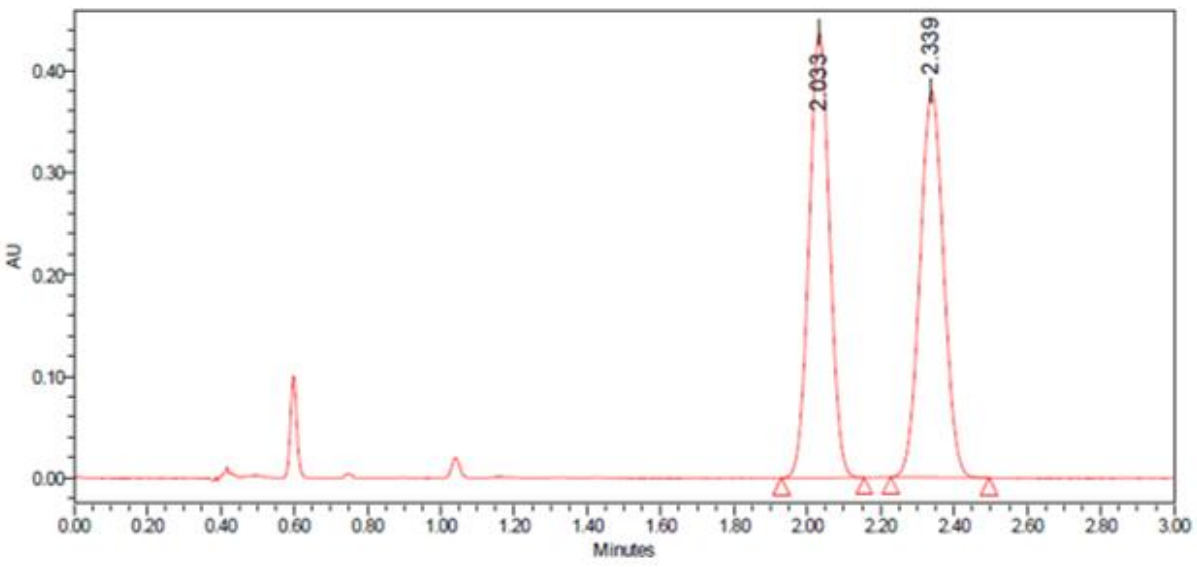

Peak Results

	Name	RT	Area	Height	% Area	Start Time (min)	End Time (min)
1		2.033	1697862	436677	50.03	1.929	2.156
2		2.339	1695527	379030	49.97	2.228	2.496

Isolated product

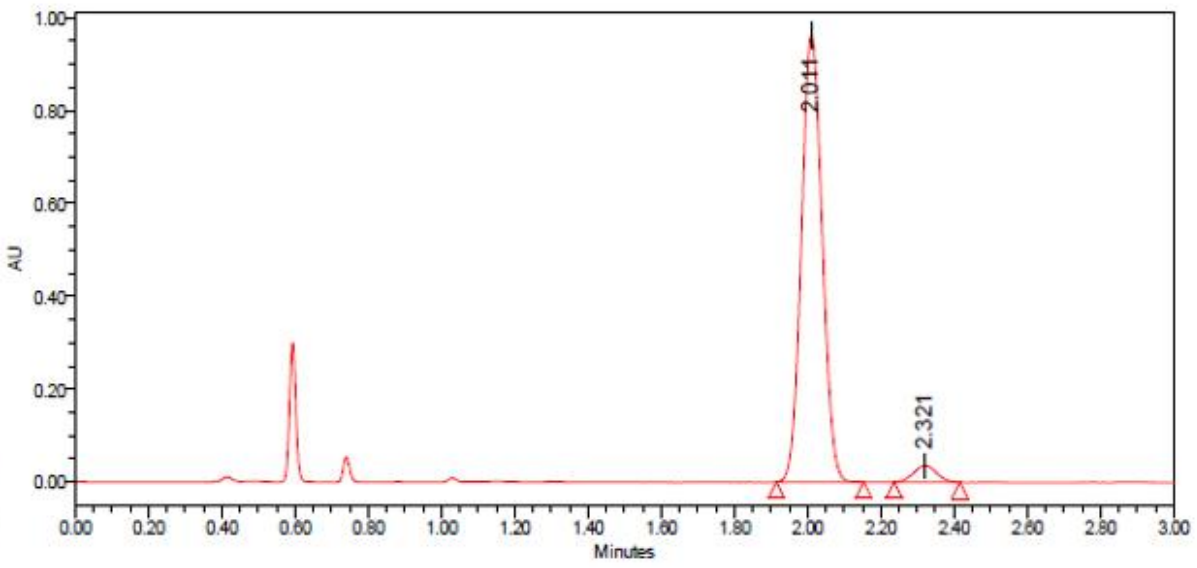

Peak Results

	Name	RT	Area	Height	% Area	Start Time (min)	End Time (min)
1		2.011	3790378	961627	96.07	1.915	2.153
2		2.321	155176	35593	3.93	2.237	2.416

^{1}H NMR (500 MHz, $CDCl_3$) of **5c**:

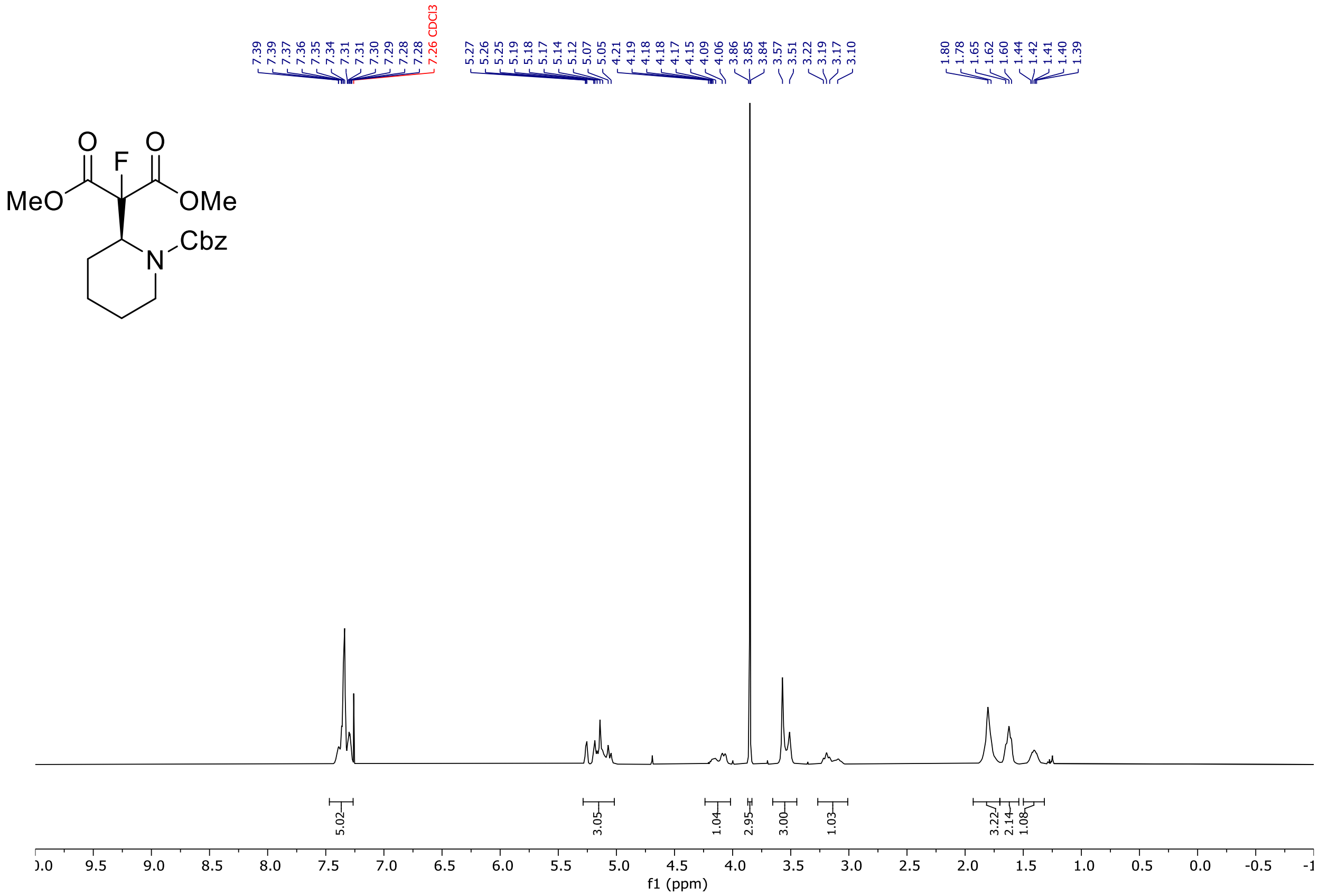

^{19}F NMR {^{1}H} (471 MHz, $CDCl_3$) of **5c**:

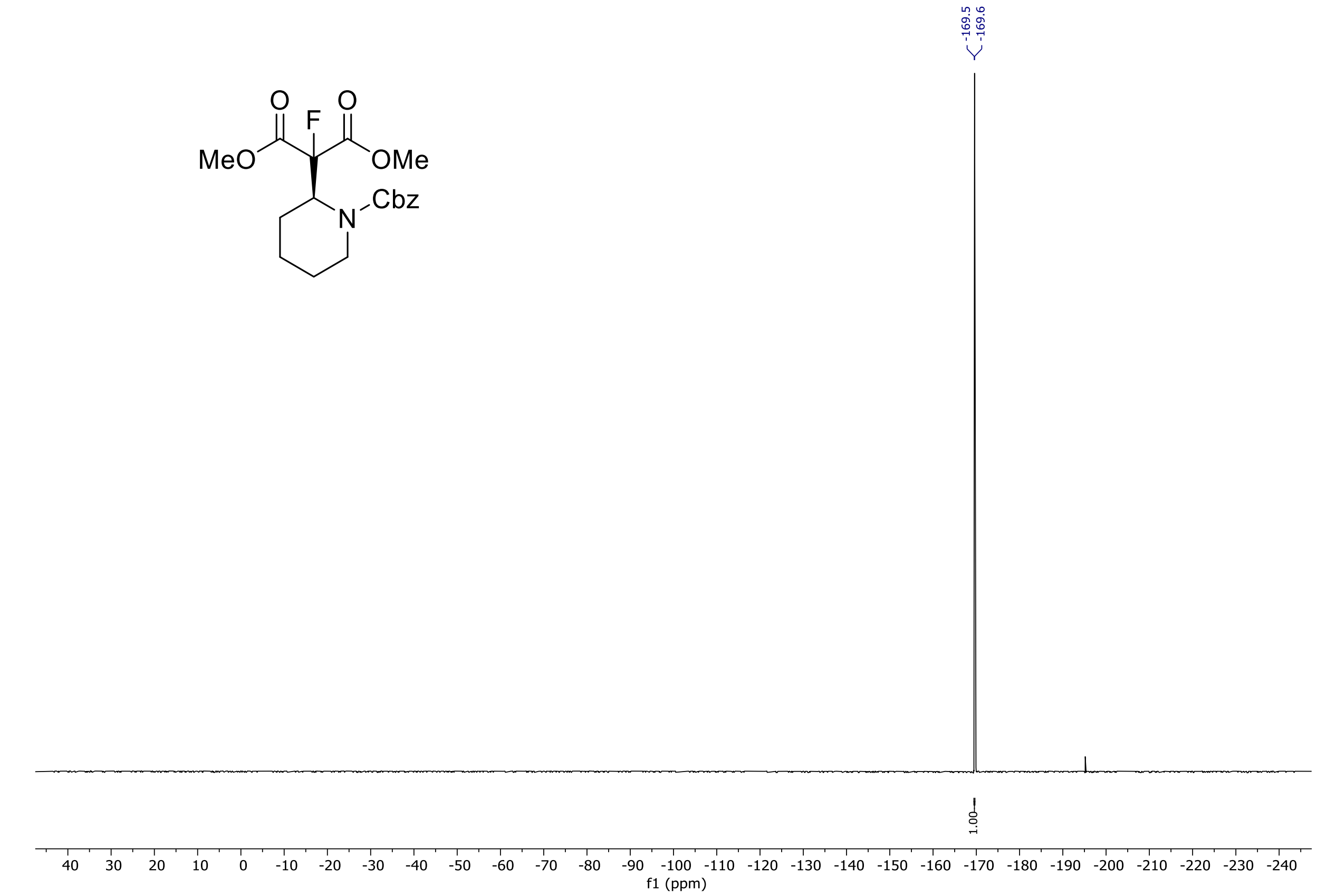

^{19}F NMR (471 MHz, $CDCl_3$) of **5c**:

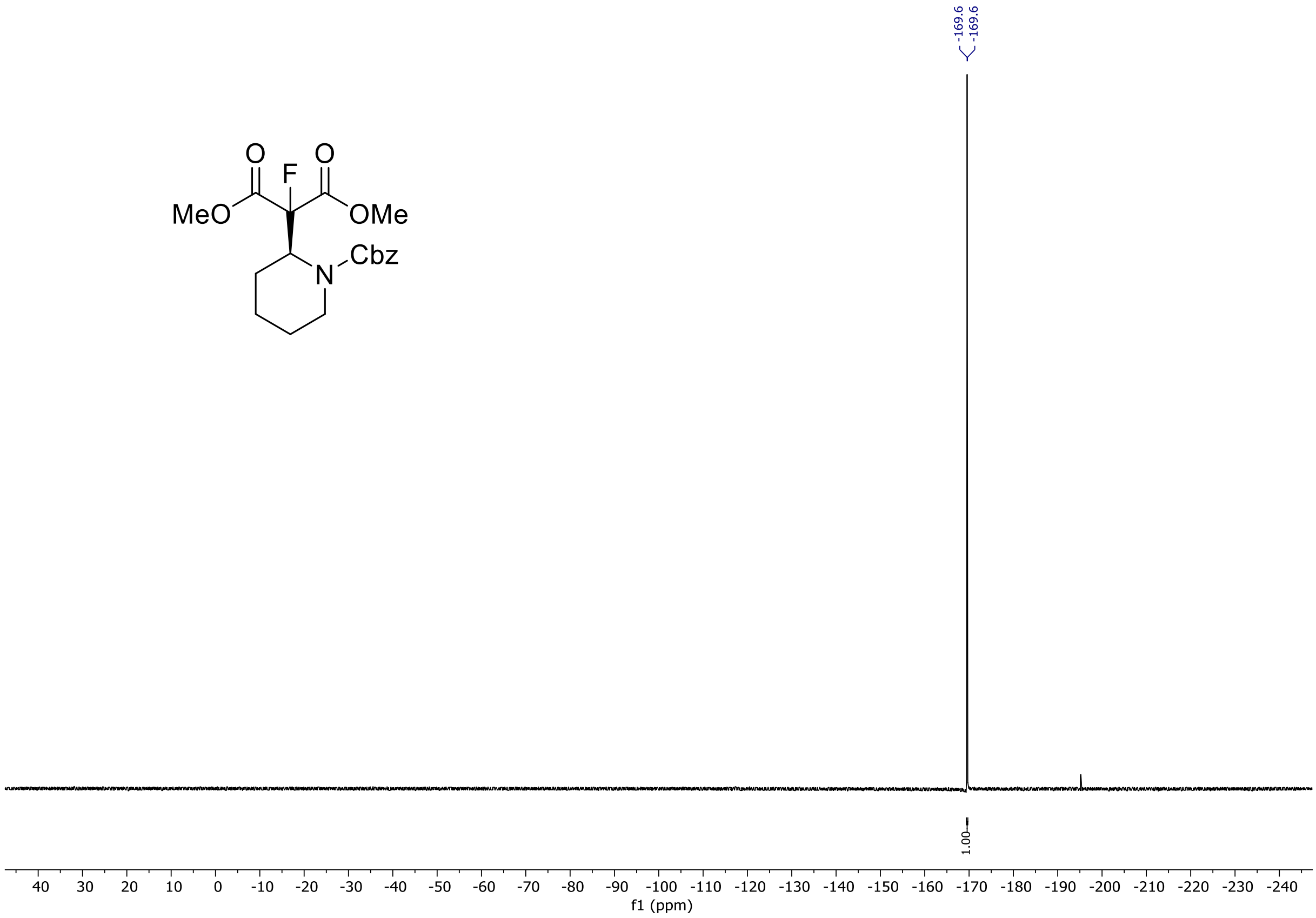

^{13}C NMR (126 MHz, $CDCl_3$) of **5c**:

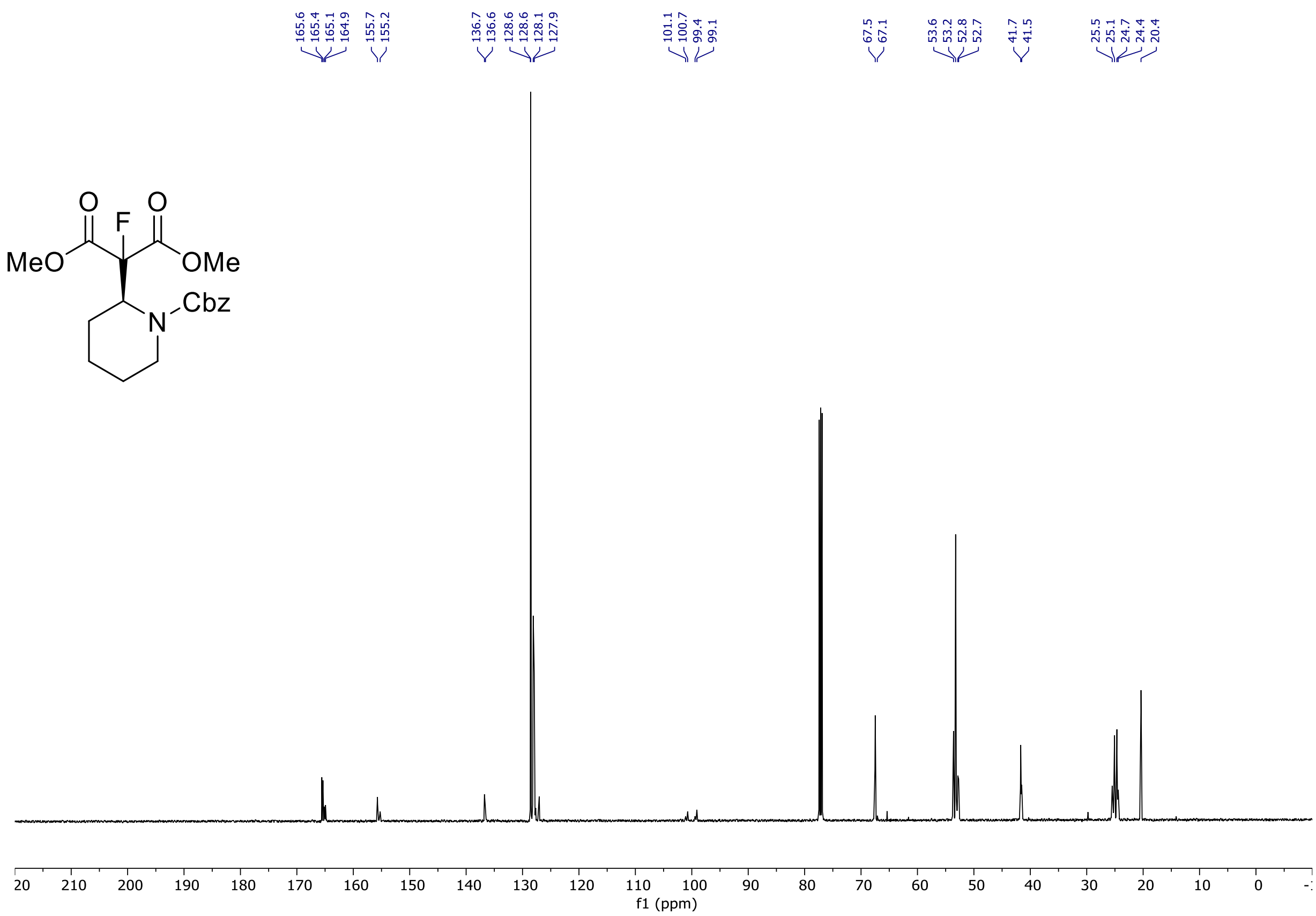

The enantiomeric access of **5c** was determined by chiral stationary phase HPLC: IA column, hexane/*i*-PrOH 80:20, 0.5 mL/min, 25 °C, 210 nm; 19.5 min (major), 21.9 min (minor).

racemate

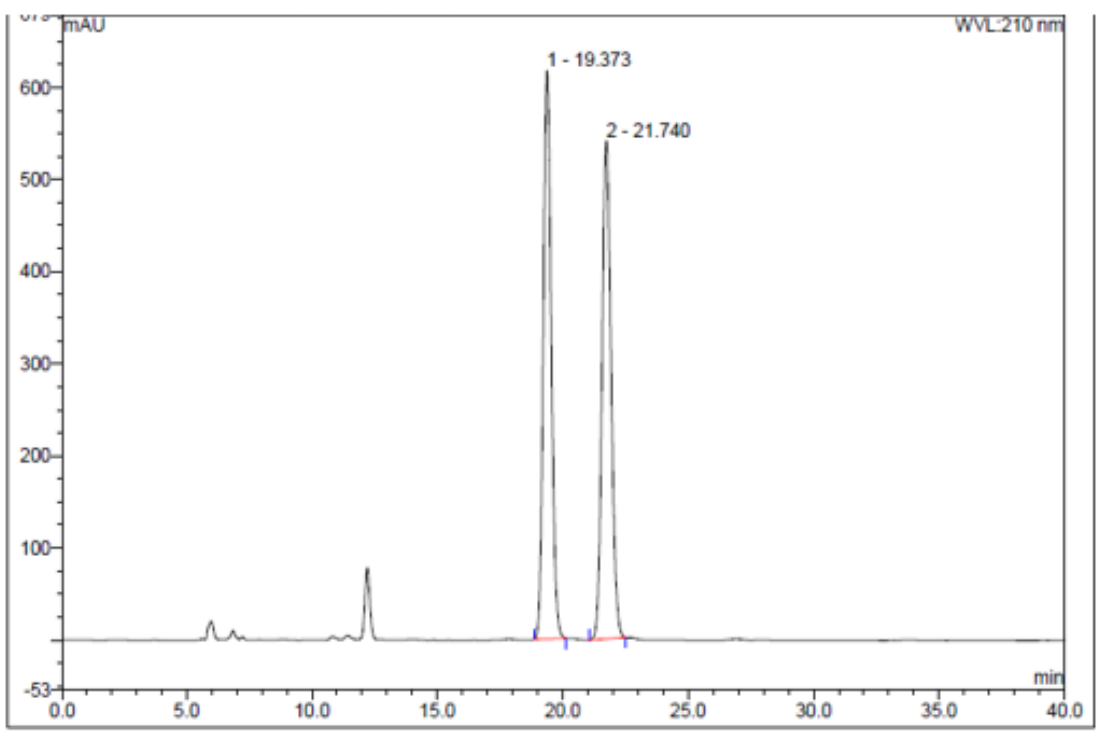

No.	Ret.Time min	Peak Name	Height mAU	Area mAU*min	Rel.Area %	Amount	Type
1	19.37	n.a.	616.861	226.230	50.02	n.a.	BMB*
2	21.74	n.a.	540.496	226.091	49.98	n.a.	BMB*
Total:			1157.358	452.321	100.00	0.000	

Isolated product

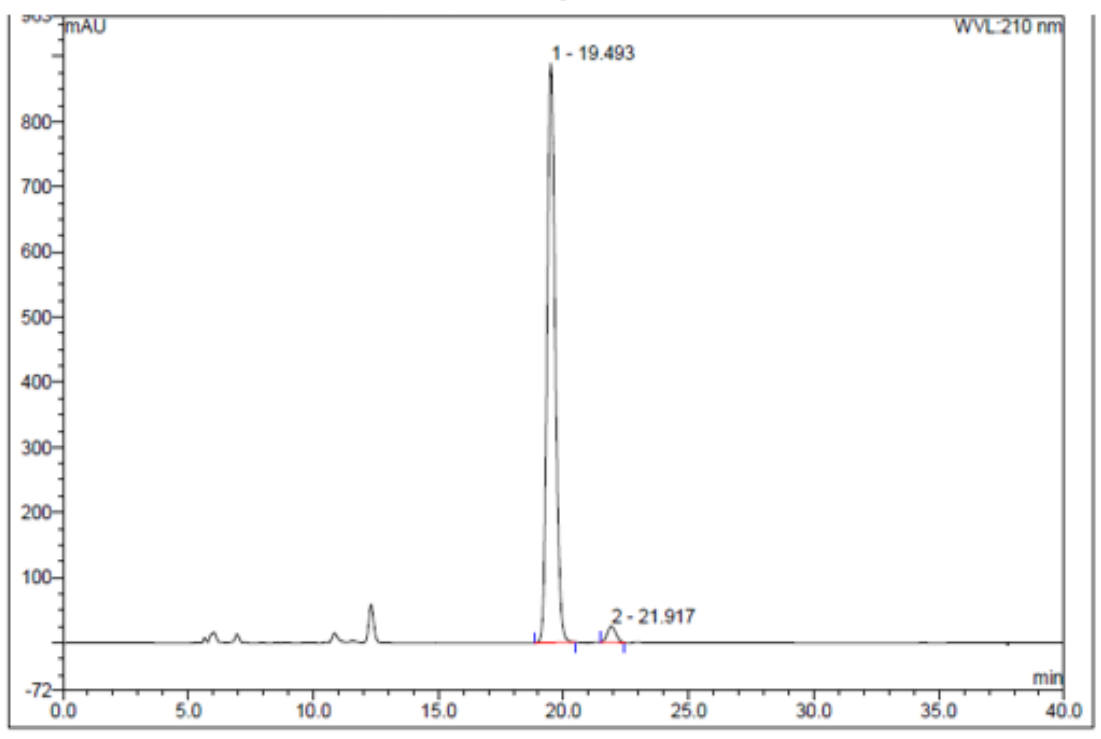

No.	Ret.Time min	Peak Name	Height mAU	Area mAU*min	Rel.Area %	Amount	Type
1	19.49	n.a.	888.993	334.557	97.14	n.a.	BMB*
2	21.92	n.a.	24.619	9.840	2.86	n.a.	BMB*
Total:			913.612	344.397	100.00	0.000	

^{1}H NMR (400 MHz, $CDCl_3$) of **6**:

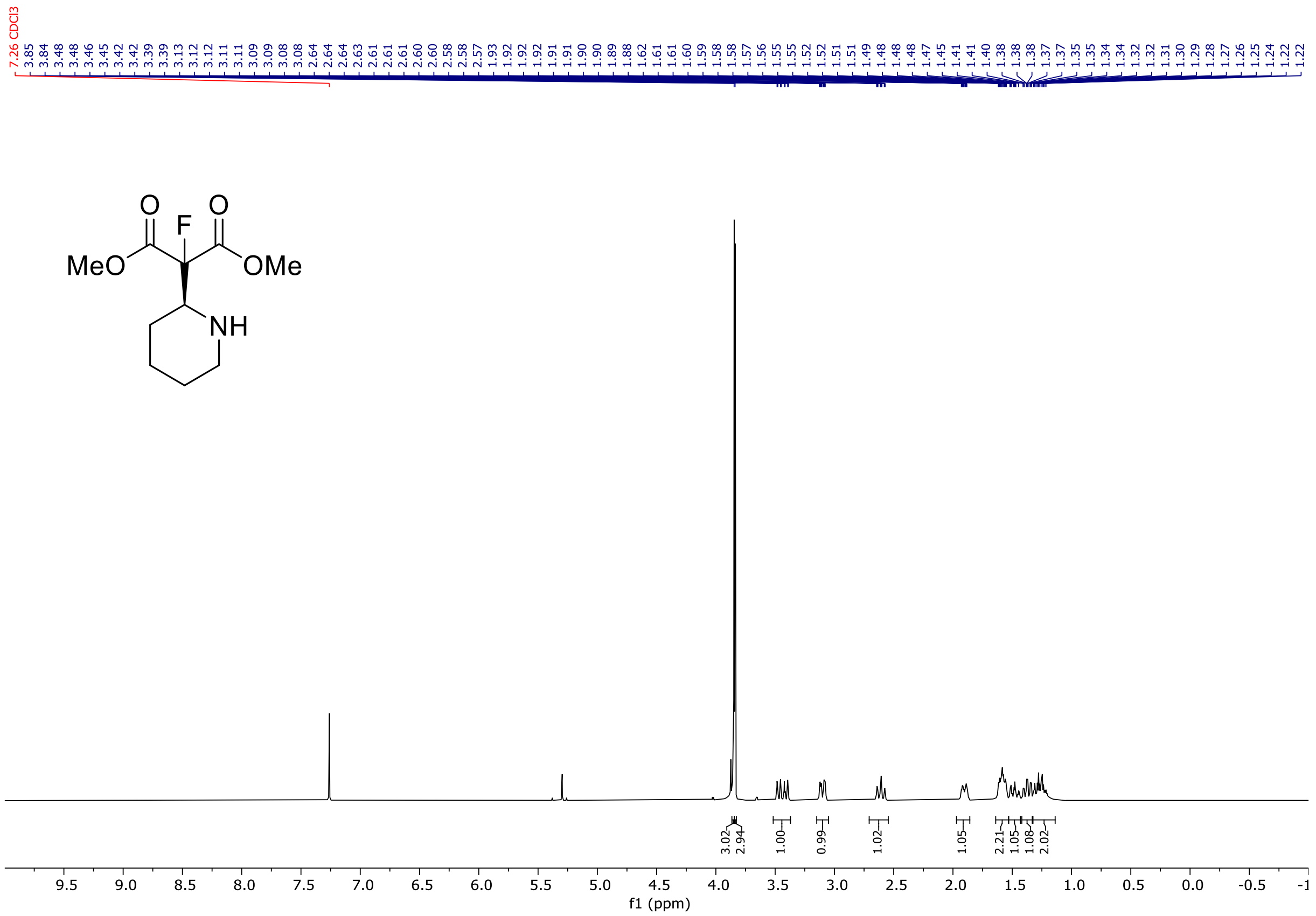

^{19}F NMR {^{1}H} (377 MHz, $CDCl_3$) of **6**:

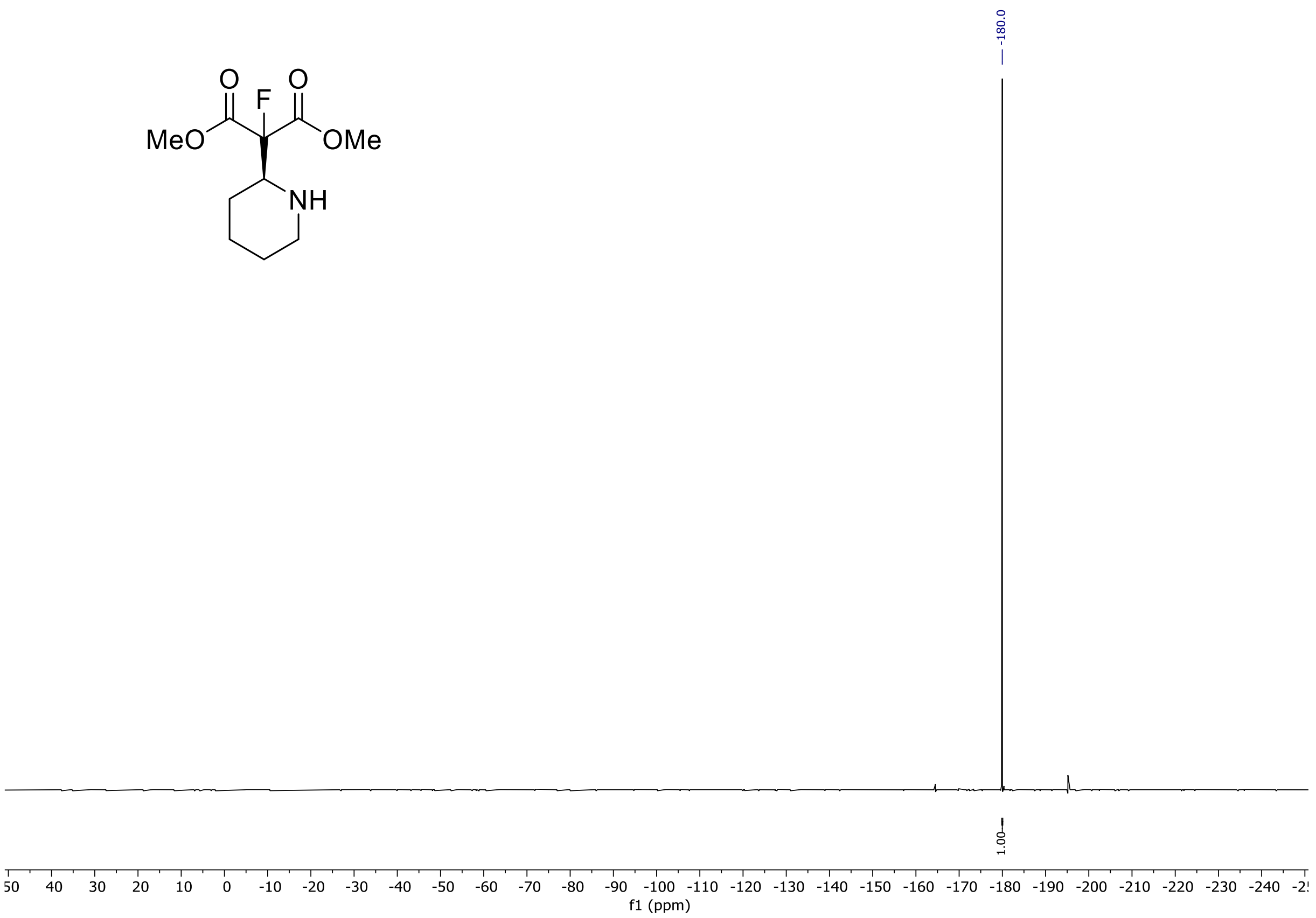

^{19}F NMR (377 MHz, $CDCl_3$) of **6**:

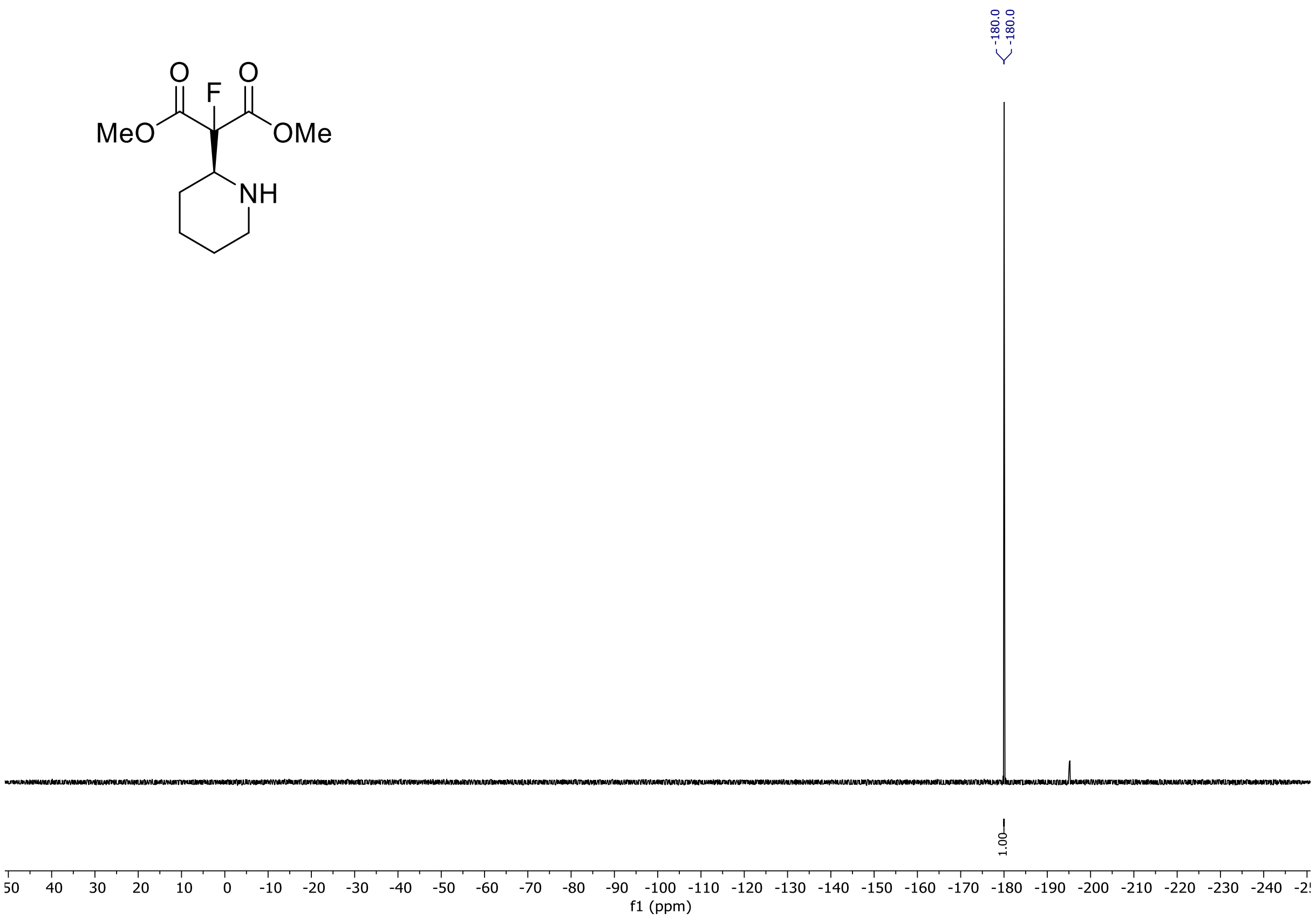

^{13}C NMR (101 MHz, $CDCl_3$) of **6**:

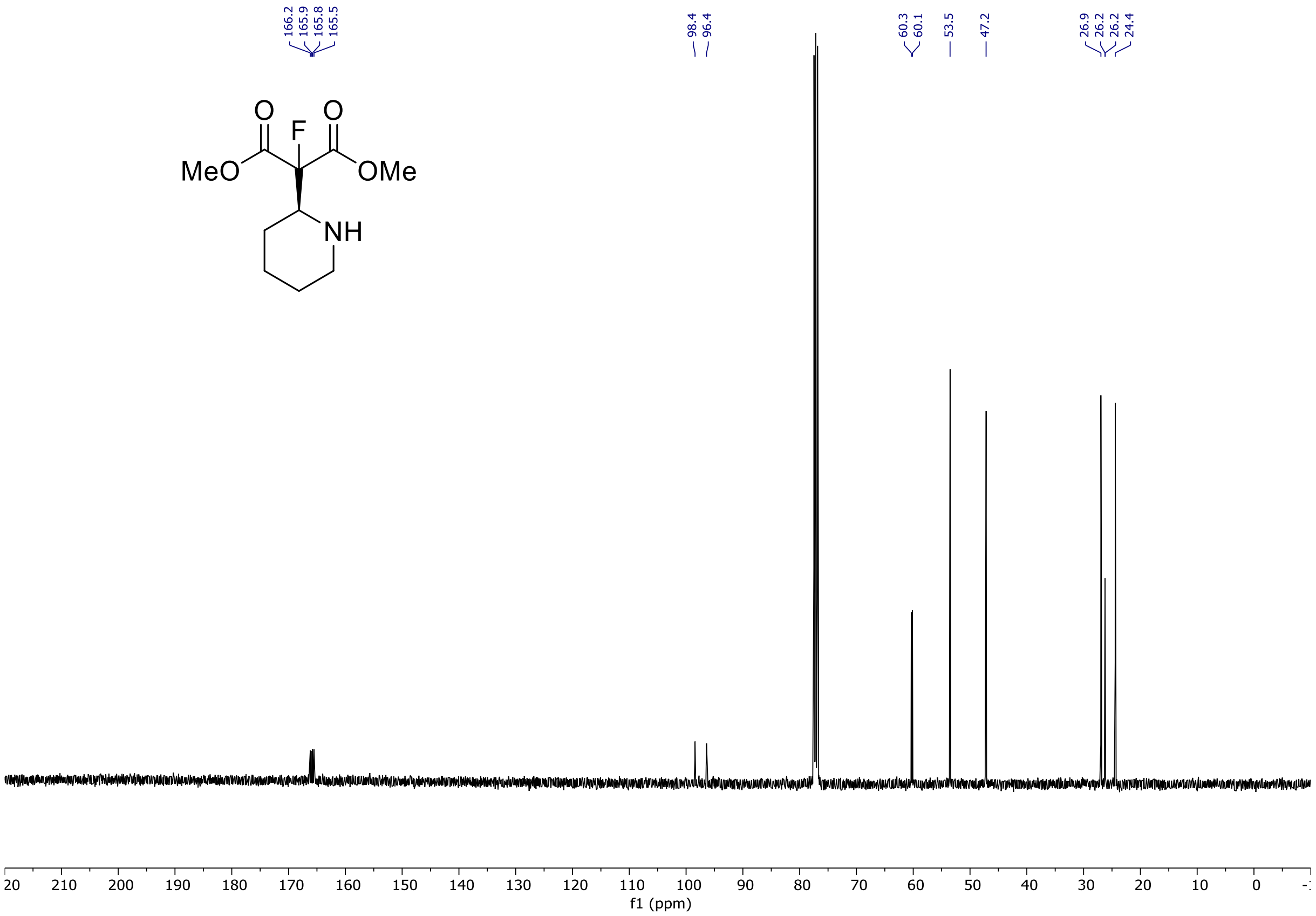

The enantiomeric access of **6** was determined by chiral stationary phase SFC: Trefoil column, CO_2/MeOH 95:5, 2.0 mL/min, 214 nm; 0.9 min (major), 1.6 min (minor).

racemate

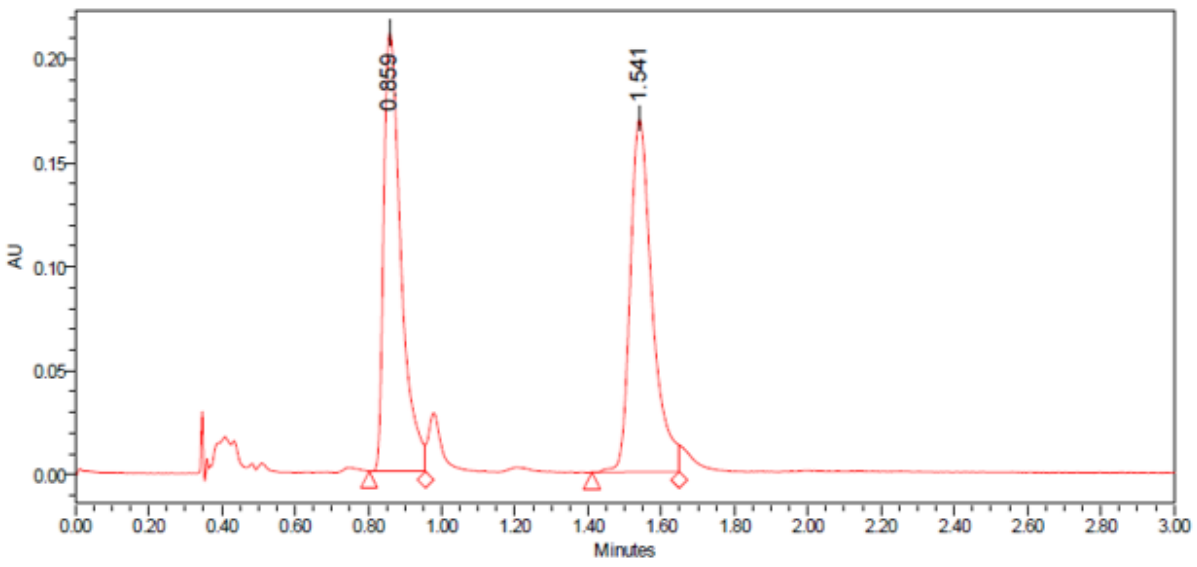

Peak Results

	Name	RT	Area	Height	% Area	Start Time (min)	End Time (min)
1		0.859	691272	21115	49.34	0.803	0.957
2		1.541	709747	170074	50.66	1.411	1.649

Isolated product

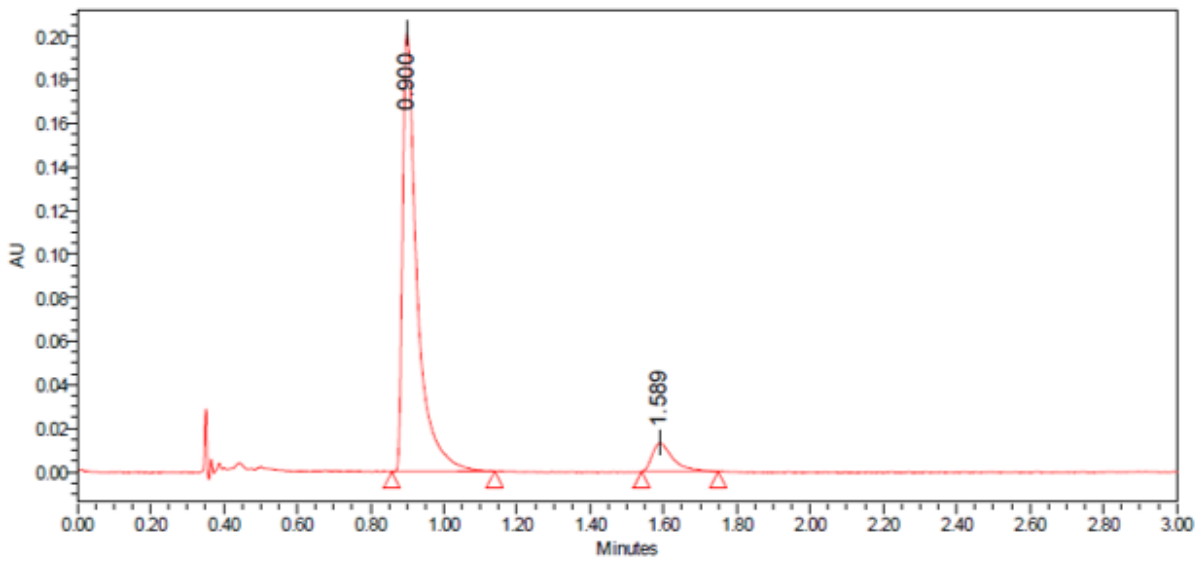

Peak Results

	Name	RT	Area	Height	% Area	Start Time (min)	End Time (min)
1		0.900	552667	201632	91.52	0.858	1.139
2		1.589	51234	13070	8.48	1.540	1.749

10 NMR Spectra and Chiral Stationary Phase HPLC Chromatograms of Compounds in Tetraponerine total synthesis

^{1}H NMR (400 MHz, $CDCl_3$) of **7**:

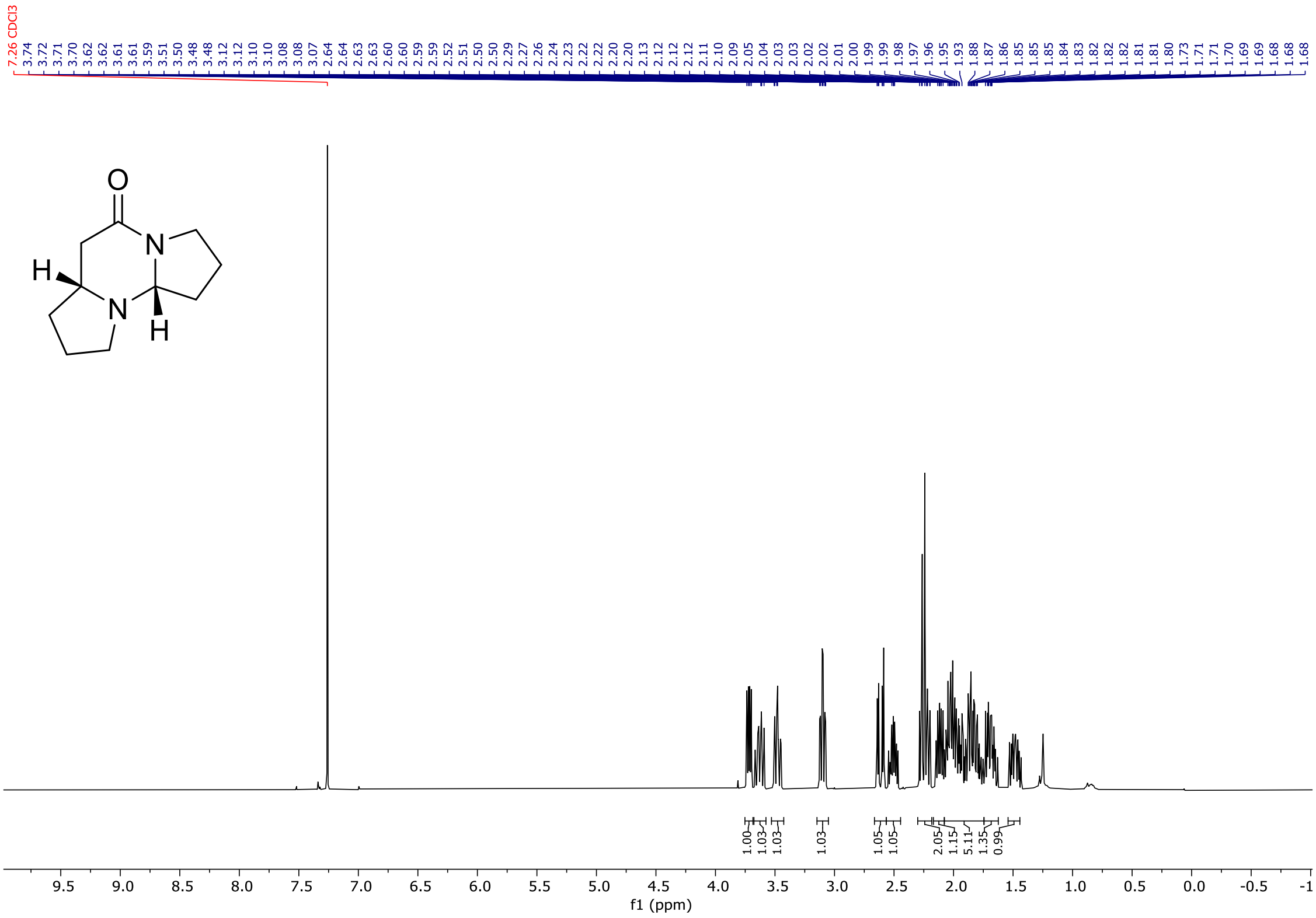

^{13}C NMR (101 MHz, $CDCl_3$) of **7**:

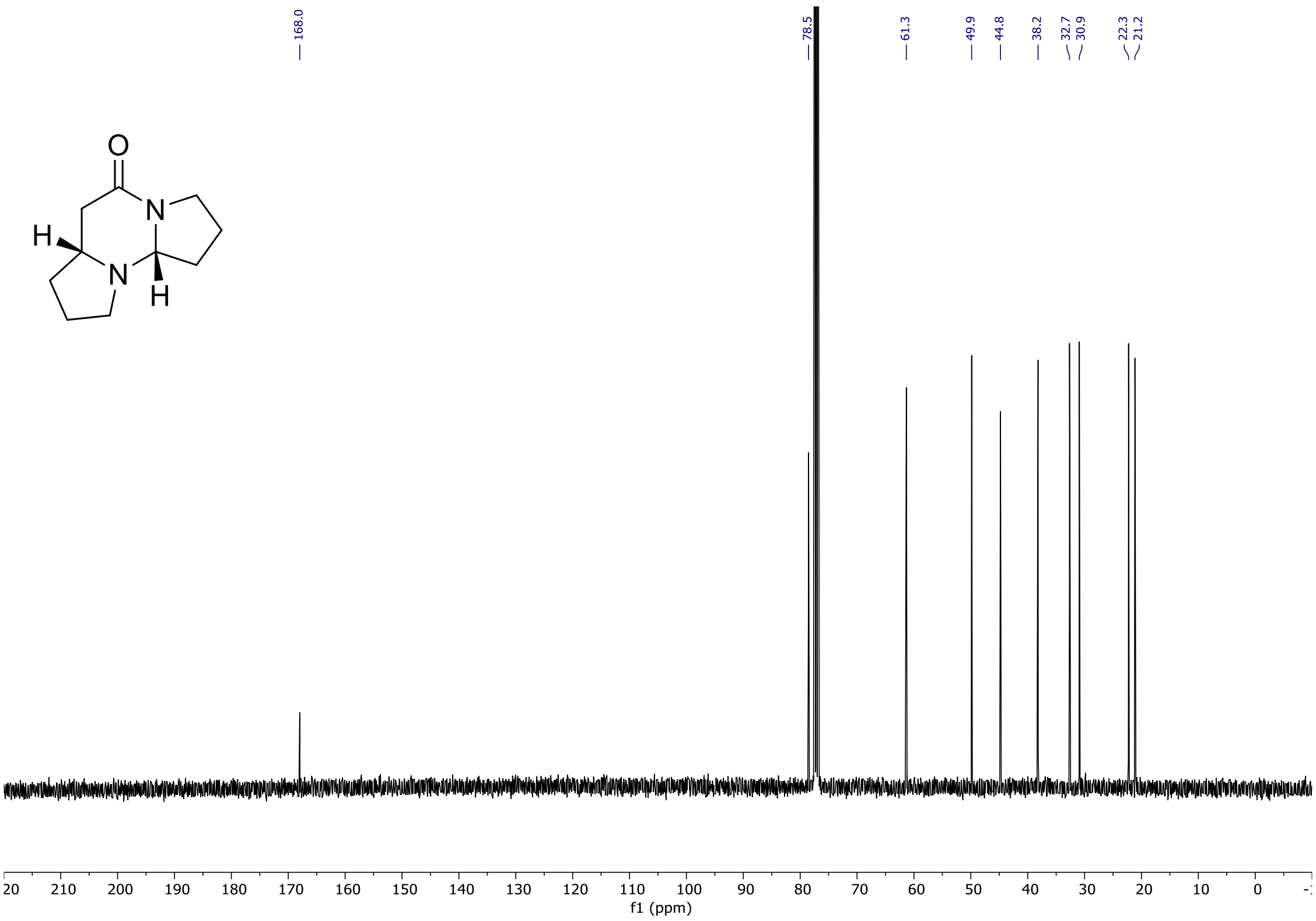

The enantiomeric access of **7** was determined by chiral stationary phase HPLC: OD column, hexane/*i*-PrOH 85:15, 0.5 mL/min, 25 °C, 210 nm; 15.2 min (major), 17.3 min (minor).

racemate

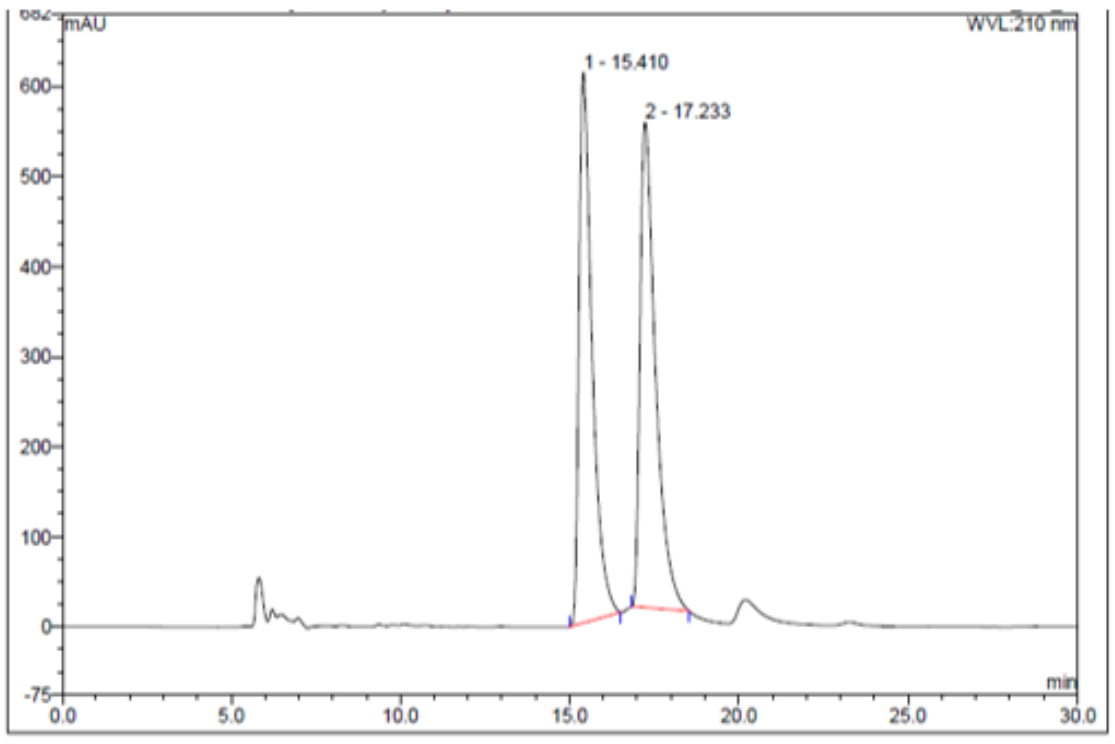

No.	Ret.Time min	Peak Name	Height mAU	Area mAU*min	Rel.Area %	Amount	Type
1	15.41	n.a.	611.642	275.275	49.04	n.a.	BMB*
2	17.23	n.a.	539.004	286.085	50.96	n.a.	BMB*
Total:			1150.646	561.360	100.00	0.000	

Isolated product

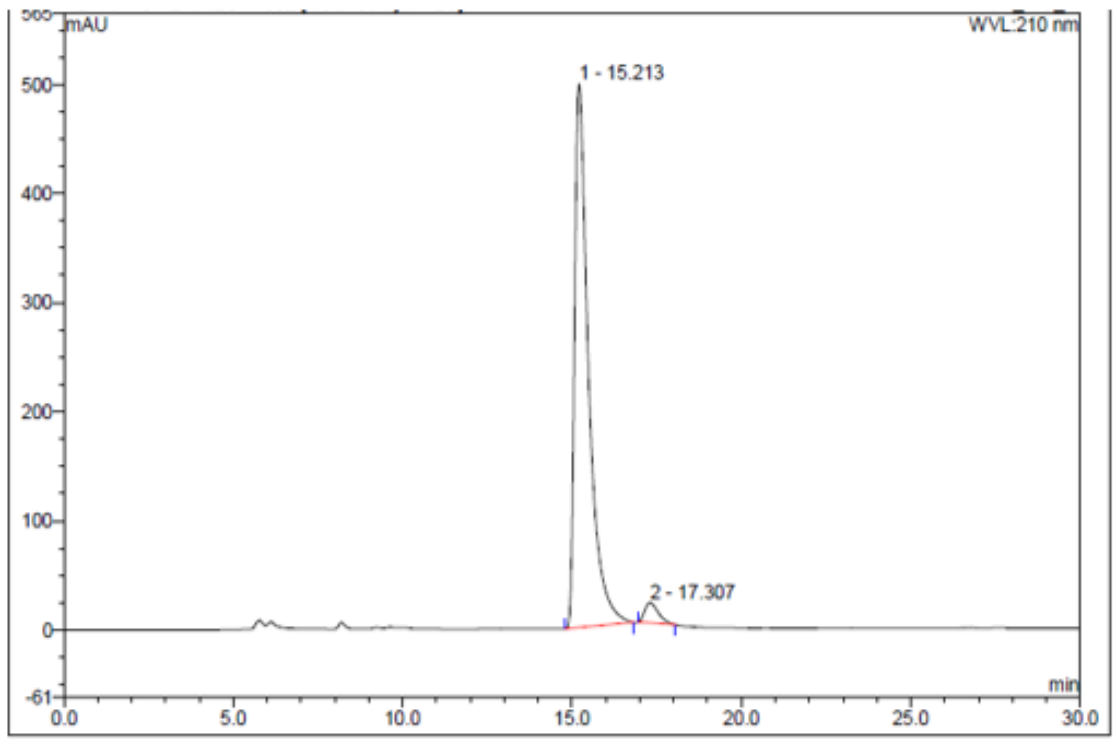

No.	Ret.Time min	Peak Name	Height mAU	Area mAU*min	Rel.Area %	Amount	Type
1	15.21	n.a.	498.402	238.114	96.48	n.a.	BMB*
2	17.31	n.a.	18.543	8.697	3.52	n.a.	BMB*
Total:			516.944	246.811	100.00	0.000	

^{1}H NMR (500 MHz, $CDCl_3$) of *ent*-**7**:

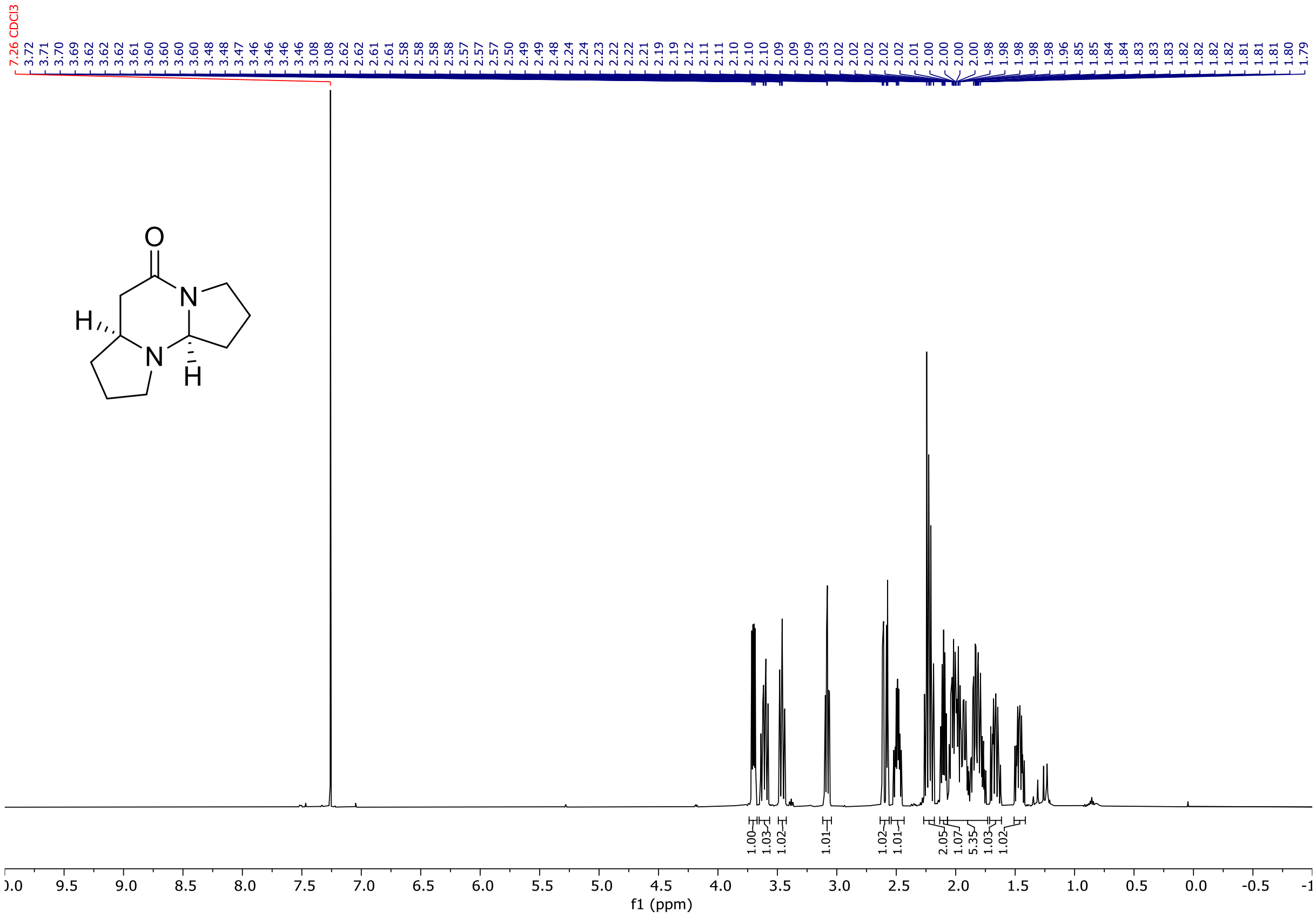

^{13}C NMR (126 MHz, $CDCl_3$) of ent-**7**:

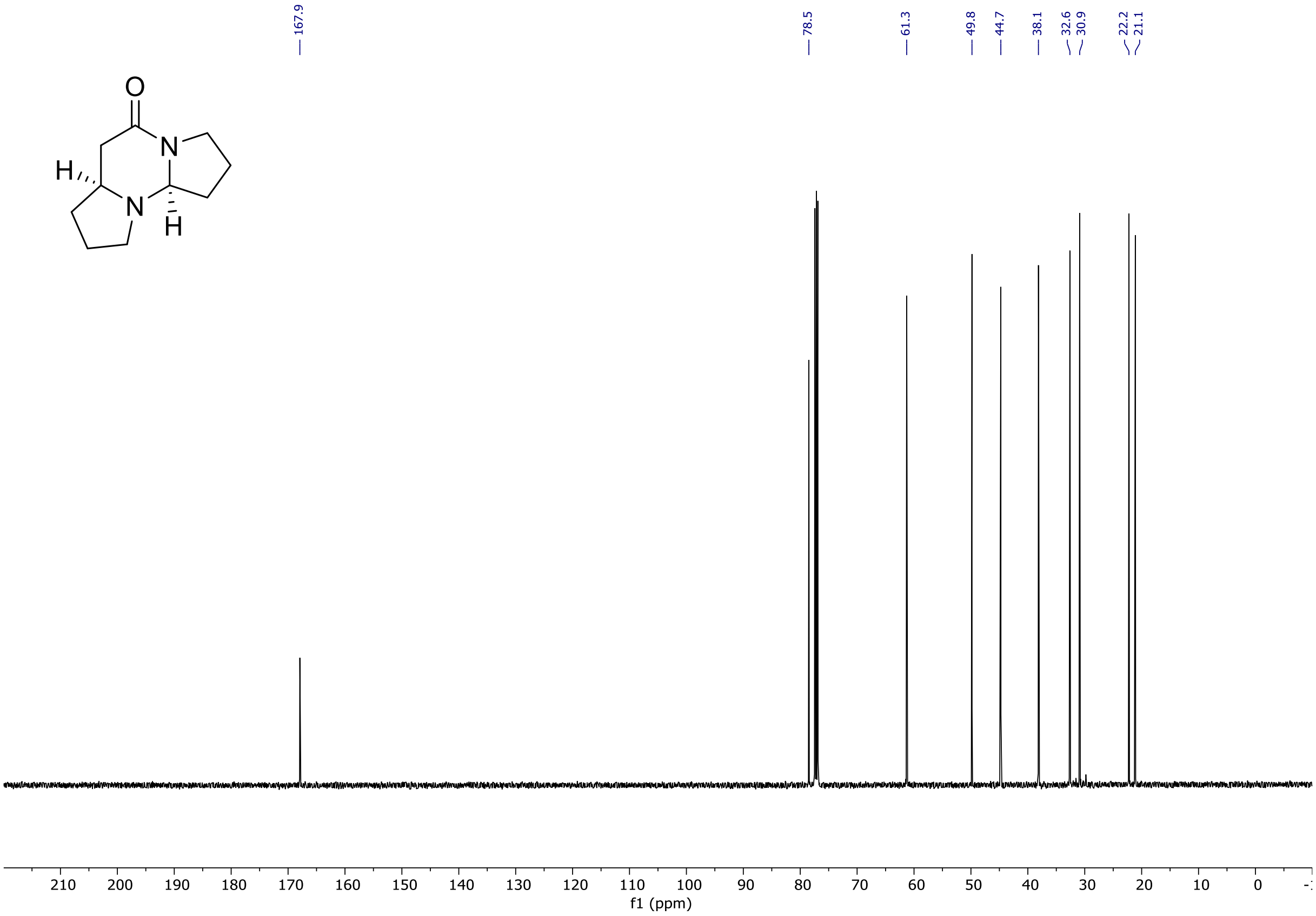

The enantiomeric access of *ent*-**7** was determined by chiral stationary phase HPLC: OD column, hexane/*i*-PrOH 85:15, 0.5 mL/min, 25 °C, 210 nm; 15.3 min (minor), 17.1 min (major).

racemate

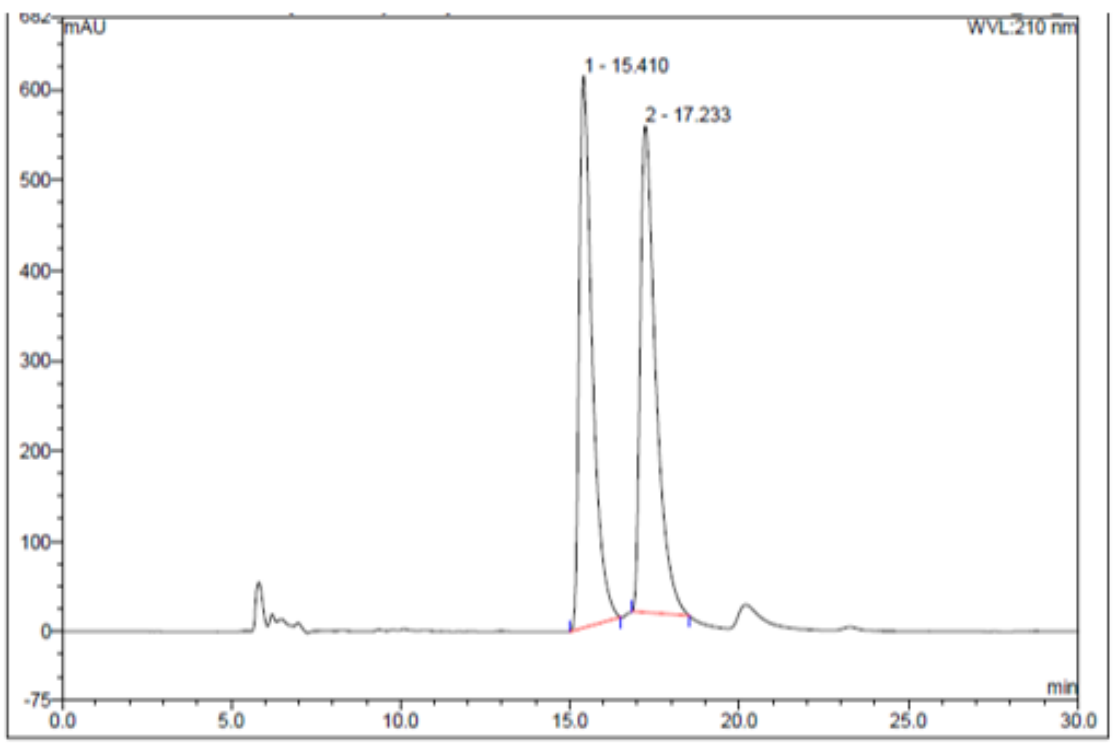

No.	Ret.Time min	Peak Name	Height mAU	Area mAU*min	Rel.Area %	Amount	Type
1	15.41	n.a.	611.642	275.275	49.04	n.a.	BMB*
2	17.23	n.a.	539.004	286.085	50.96	n.a.	BMB*
Total:			1150.646	561.360	100.00	0.000	

Isolated product

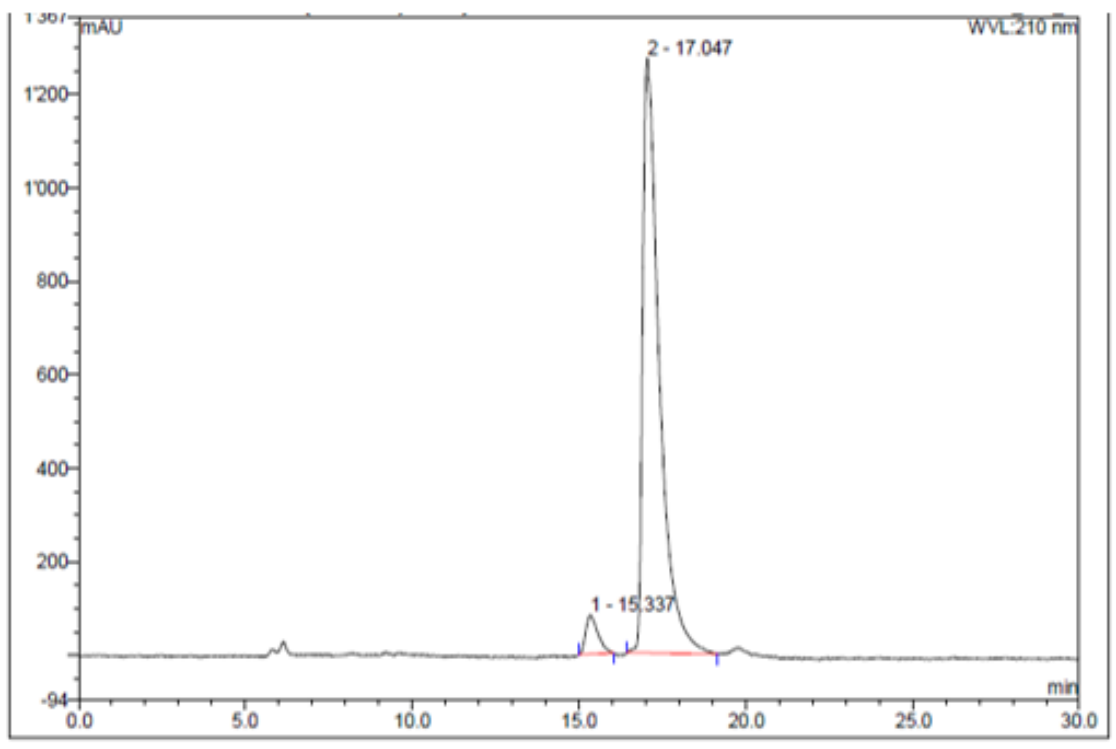

No.	Ret.Time min	Peak Name	Height mAU	Area mAU*min	Rel.Area %	Amount	Type
1	15.34	n.a.	83.366	34.956	4.55	n.a.	BMB*
2	17.05	n.a.	1273.028	733.475	95.45	n.a.	BMB*
Total:			1356.394	768.431	100.00	0.000	

^{1}H NMR (500 MHz, $CDCl_3$) of Tetraponerine **T-1**: The molecule adopts two conformations in $CDCl_3$.[26]

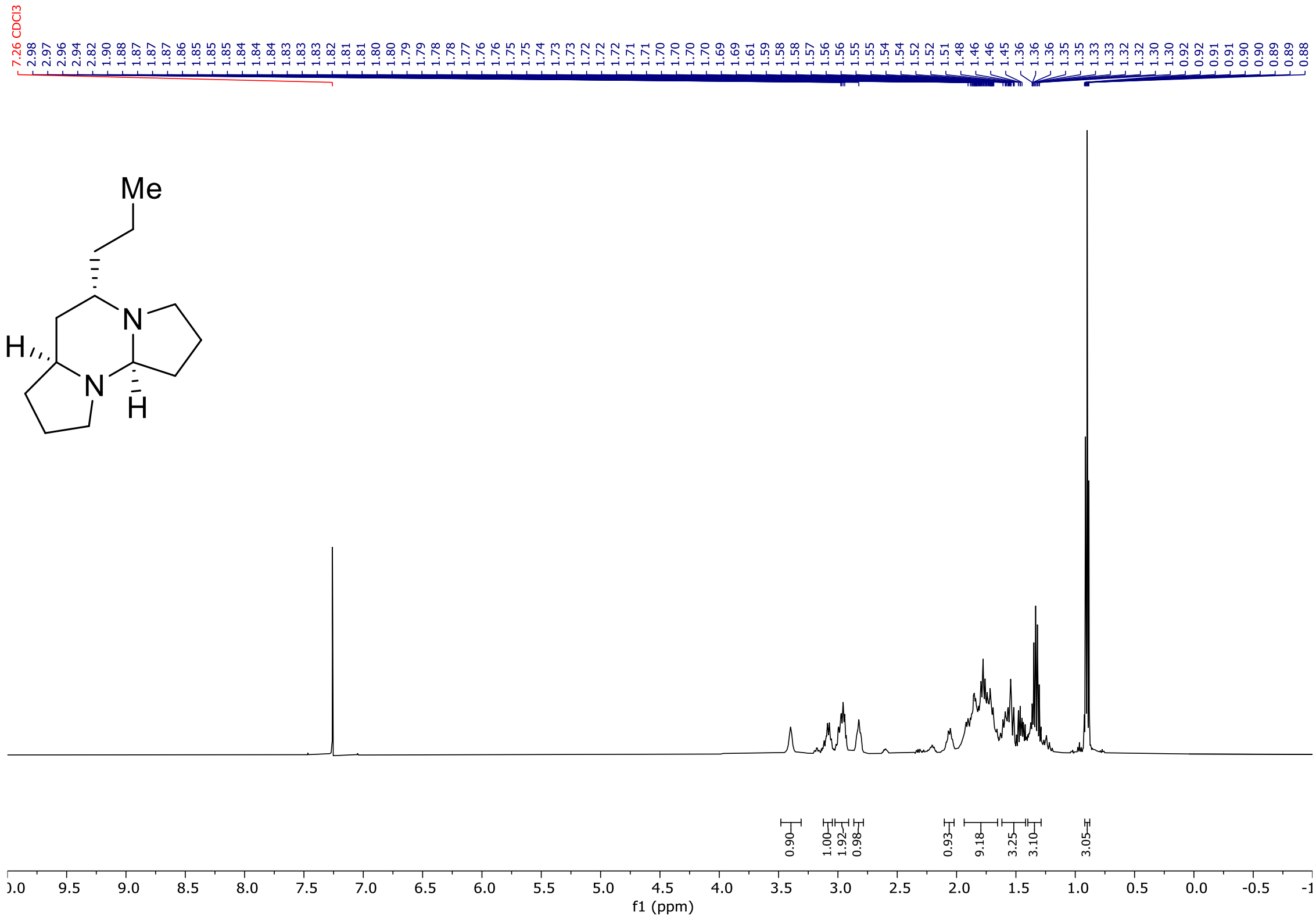

^{13}C NMR (126 MHz, $CDCl_3$) of Tetraponerine **T-1**: The molecule adopts two conformations in $CDCl_3$.[26]

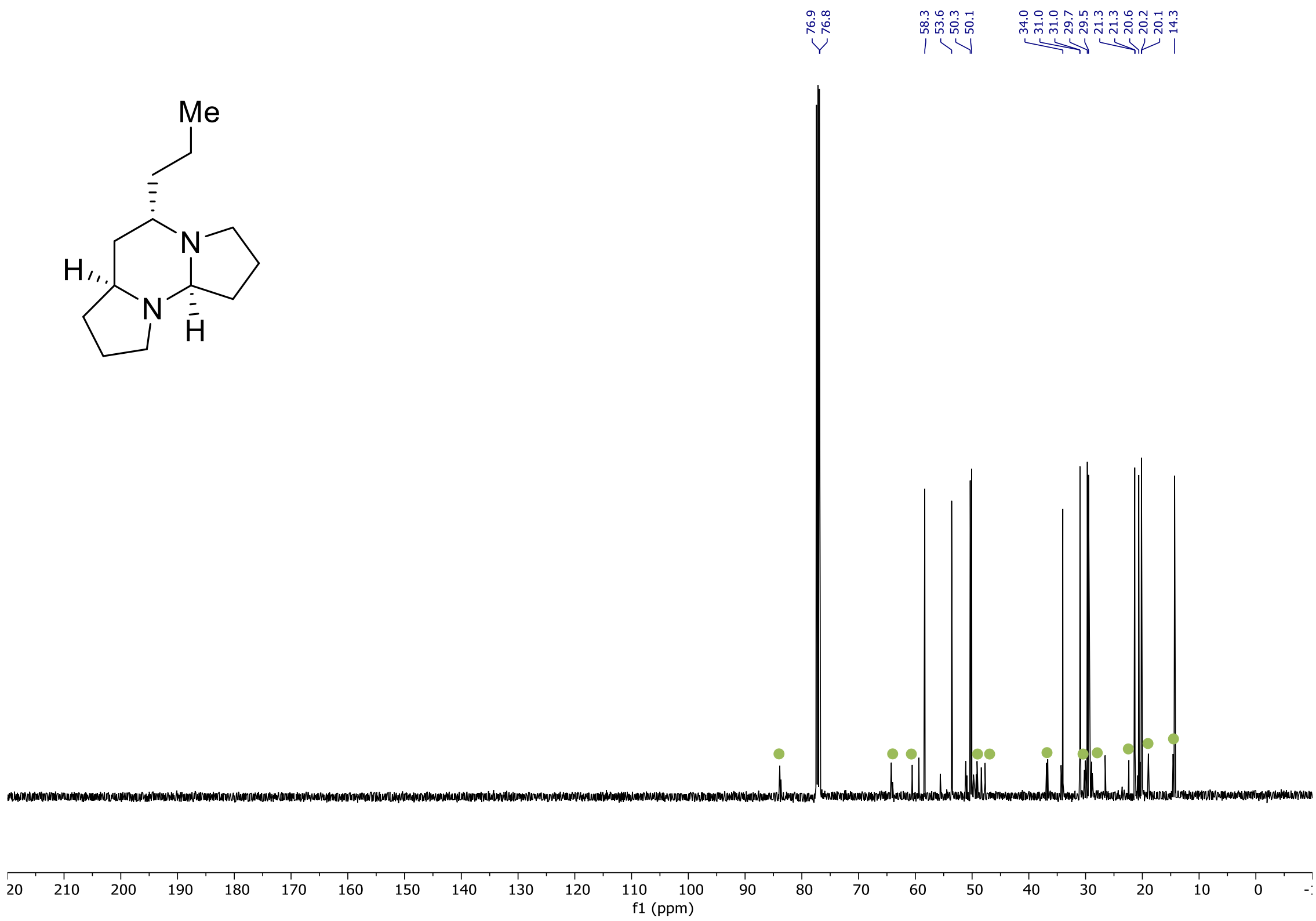

● = signals of Tetraponerine **T-2**

¹H NMR (400 MHz, $CDCl_3$) of *ent*-Tetraponerine **T-1**: The molecule adopts two conformations in $CDCl_3$.[26

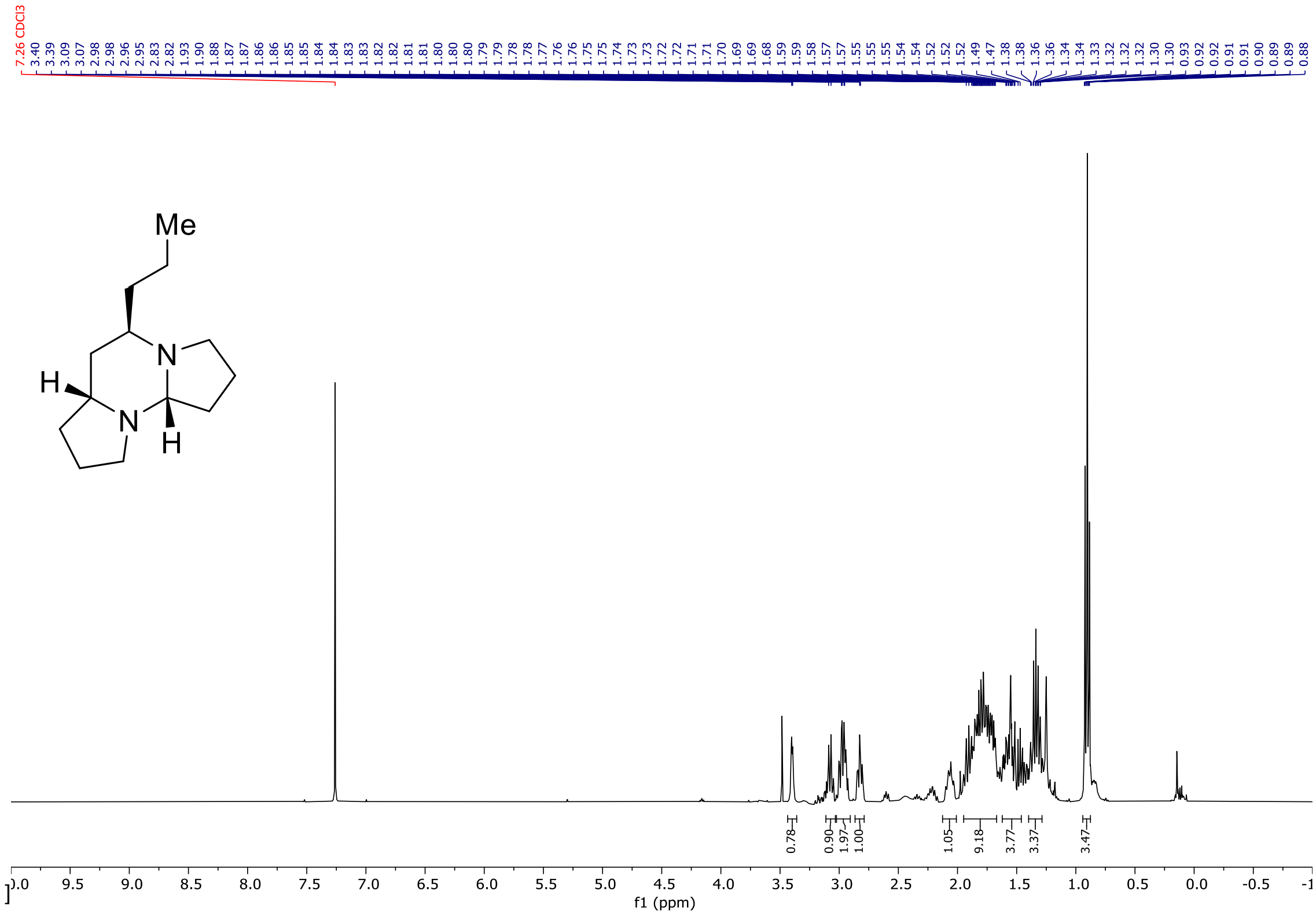

¹³C NMR {¹H} (101 MHz, $CDCl_3$) of *ent*-Tetraponerine **T-1**: The molecule adopts two conformations in $CDCl_3$.[26]

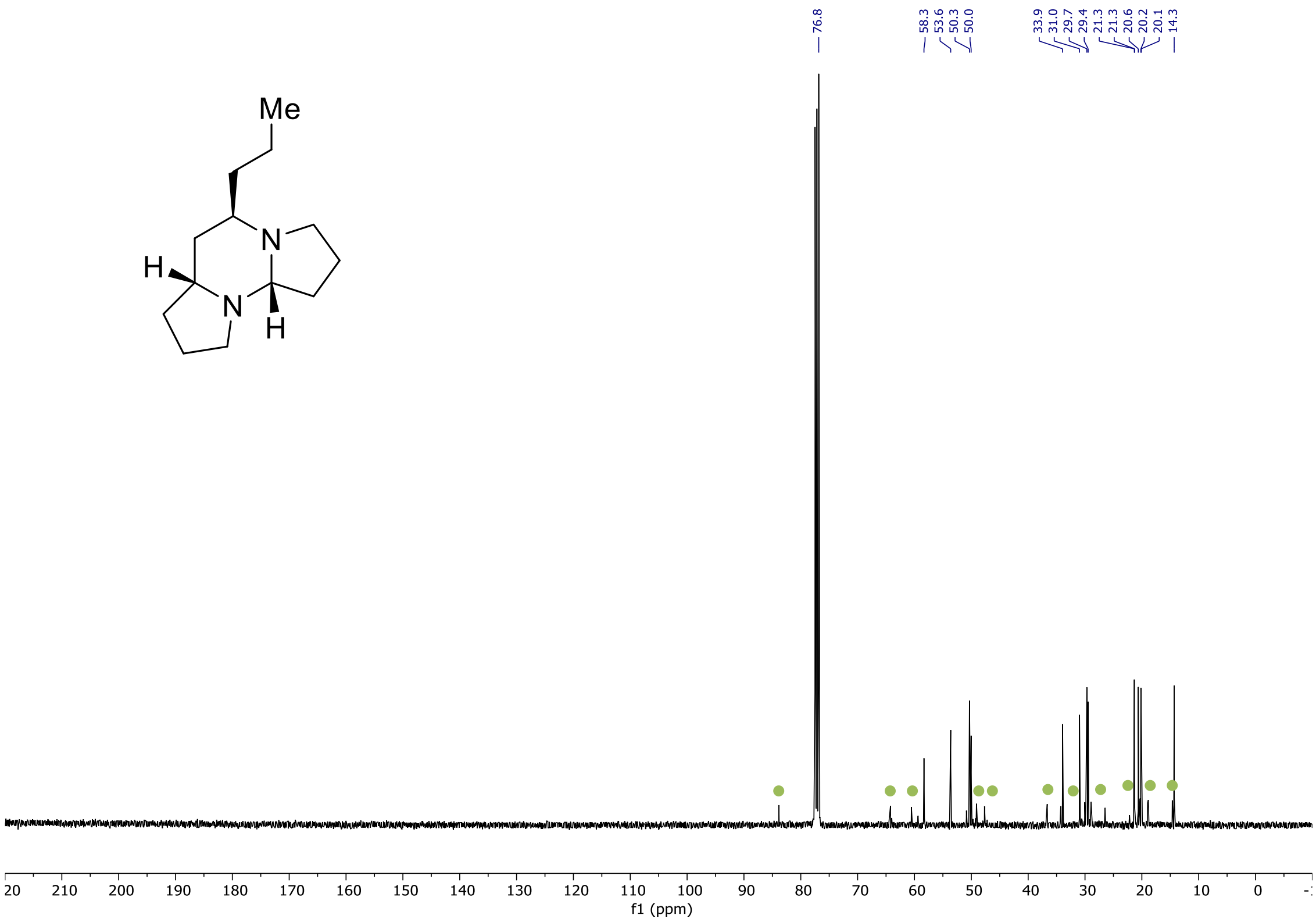

• = signals of *ent*-Tetraponerine **T-2**
